# *Liaisons dangereuses*: Intrinsic Disorder in Cellular Proteins Recruited to Viral Infection-Related Biocondensates

**DOI:** 10.3390/ijms24032151

**Published:** 2023-01-21

**Authors:** Greta Bianchi, Stefania Brocca, Sonia Longhi, Vladimir N. Uversky

**Affiliations:** 1 Department of Biotechnology and Biosciences, University of Milano-Bicocca, 20126 Milano, Italy; 2 Laboratoire Architecture et Fonction des Macromolécules Biologiques (AFMB), UMR 7257, Aix Marseille University and CNRS, 13288 Marseille, France; 3 Department of Molecular Medicine, Byrd Alzheimer’s Research Institute, Morsani College of Medicine, University of South Florida, Tampa, FL 33601, USA

**Keywords:** liquid–liquid phase separation, membrane-less organelles, intrinsically disordered proteins, intrinsically disordered regions, viral factories, viral inclusion bodies, viral infection-related MLOs, protein–protein interactions, post-translational modifications

## Abstract

Liquid–liquid phase separation (LLPS) is responsible for the formation of so-called membrane-less organelles (MLOs) that are essential for the spatio-temporal organization of the cell. Intrinsically disordered proteins (IDPs) or regions (IDRs), either alone or in conjunction with nucleic acids, are involved in the formation of these intracellular condensates. Notably, viruses exploit LLPS at their own benefit to form viral replication compartments. Beyond giving rise to biomolecular condensates, viral proteins are also known to partition into cellular MLOs, thus raising the question as to whether these cellular phase-separating proteins are *drivers* of LLPS or behave as *clients/regulators*. Here, we focus on a set of eukaryotic proteins that are either sequestered in viral factories or colocalize with viral proteins within cellular MLOs, with the primary goal of gathering organized, predicted, and experimental information on these proteins, which constitute promising targets for innovative antiviral strategies. Using various computational approaches, we thoroughly investigated their disorder content and inherent propensity to undergo LLPS, along with their biological functions and interactivity networks. Results show that these proteins are on average, though to varying degrees, enriched in disorder, with their propensity for phase separation being correlated, as expected, with their disorder content. A trend, which awaits further validation, tends to emerge whereby the most disordered proteins serve as *drivers*, while more ordered cellular proteins tend instead to be *clients* of viral factories. In light of their high disorder content and their annotated LLPS behavior, most proteins in our data set are *drivers* or *co-drivers* of molecular condensation, foreshadowing a key role of these cellular proteins in the scaffolding of viral infection-related MLOs.

## 1. Introduction

Liquid–liquid phase separation (LLPS) is a physico-chemical process by which a homogeneous solution demixes to form a dense and a light phase, with the solute being more concentrated in the dense phase than in the light phase [1]. LLPS sensitivity to variations in temperature, ionic strength, pH, and solute concentration makes this process an ideally suited mechanism for the spatio-temporal organization of macromolecular components in living cells [2,3,4,5,6,7]. In living cells, molecular condensation involves proteins and often nucleic acids, producing a peculiar kind of biomolecular condensate referred to as membrane-less organelles (MLOs). Cells in all kingdoms of life might have hundreds of different MLOs [8], with the most known being stress granules (SGs), nucleoli, processing (P) granules, and Cajal bodies. MLOs respond not only to environmental stimuli, but also to changes in the concentration and chemical composition of solutes, i.e., they can be modulated by post-translational modifications (PTMs) of proteins undergoing phase separation [9]. Thus, by endowing the cell with the ability to transiently compartmentalize its components, LLPS enables (in)activating specific functions, responding rapidly to a range of factors, integrating them and buffering the cellular noise [10].

An analysis of the molecular features of intracellular condensation has led to a distinction between elements that actively trigger demixing, which act as LLPS *drivers* and eventually form the coacervate scaffold, and those that implant into MLOs secondarily, the so-called *clients* (or *passengers*) which are not able to trigger condensation on their own [11,12]. An additional category is provided by so-called *co-drivers*, i.e., macromolecules (proteins, RNA, or DNA) that strictly require another macromolecule to undergo phase separation. Depending on the circumstances in which demixing takes place, a particular specific component may act as either a scaffold or a client. Although *regulators*, like clients, do not physically participate in the formation of the scaffold, they can determine both the formation of an MLO and its functional and morphological properties. For instance, a typical regulator is an enzyme that induces key PTM(s) that may define the localization and/or binding properties of scaffold proteins [13]. Among the intrinsic characteristics of a protein that endow it with the scaffolding ability is undoubtedly its multivalency, i.e., the multiplicity of binding motifs. Multivalency is typical of, although not strictly restricted to, intrinsically disordered proteins (IDPs) and regions (IDRs). Therefore, it is not surprising that many MLOs and biomolecular condensates are specifically enriched in IDPs or proteins with IDRs [14]. Although the “grammar” of LLPS has only started to be deciphered, a few rules are beginning to emerge [15]. For instance, Arg/Lys-containing IDRs were shown to serve as cryptic nucleic-acid-binding domains that may phase separate upon binding nucleic acids [16]. The gathered knowledge so far has contributed to making possible the generation of synthetic MLOs endowed with controllable phase separation and cargo recruitment abilities [17].

The sequence degeneracy of IDPs/IDRs, favoring low complexity, encodes residue types and/or short motifs that favor three-dimensional networking of protein chains, and thus behave as *stickers* [18]. This ability is further amplified by their structural flexibility, conformational dynamics [19], and the general accessibility of IDRs to the enzymes catalyzing various PTMs [20] that ultimately impact their charge and hydrophobicity. Further modulation of LLPS is offered by the distribution of *stickers*. Molecular dynamics simulation studies unveiled that uniformly interspersed stickers consisting of aromatic residues promote LLPS, while their clustering leads to aggregation [21]. Sequence features influence IDP/IDR recruitment as clients as well. Indeed, electrostatic and cation-π interactions favor IDP/IDR client recruitment into numerous protein condensates [22].

Viruses broadly exploit LLPS to form viral factories, also known as inclusion bodies (IBs). Viral factories are sites where transcription and replication take place. They can be either membrane-delimited (which is typically the case in +ssRNA viruses, such as *Flaviviridae* and *Coronaviridae*) or devoid of membranes. In the latter case, they are referred to as “membrane-less replication compartments”. Viruses exploit LLPS to form not only viral factories but also assembly compartments, i.e., compartments where trafficking and assembly of viral components take place. LLPS provides an excellent solution to the problem of physical and functional separation of viral macromolecules from those endogenous to the host cell in its cytosol. Accordingly, LLPS has emerged as a new promising target for antiviral approaches [23,24,25].

One of the best-known examples of viral factories resulting from LLPS is represented by the so-called Negri bodies (NBs) in pyramidal cells of the hippocampus [26,27]. NBs have been long considered a hallmark of infection by rabies virus (RABV), a member of the *Mononegavirales* order that embraces non-segmented, negative-sense single-strand RNA (-ssRNA) viruses [28,29,30]. NBs are micrometer-sized liquid condensates containing viral RNA, together with nucleoprotein (N), phosphoprotein (P), and large protein (L), which build up the replicative complex [30,31,32]. Recent works have led to the identification of several viruses whose life cycle is mediated by the formation of molecular condensates, the liquid-like nature of which was demonstrated based on their sphericity, fluidity, and ability to coalesce (for reviews see [25,32,33,34,35,36,37,38,39,40,41,42,43,44,45,46]). Other well-known examples of viral factories resulting from LLPS pertain to other members of the *Mononegavirales* order, such as vesicular stomatitis virus (VSV) [47], measles virus (MeV) [48,49], respiratory syncytial virus (RSV) [50], human metapneumovirus (hMPV) [51], and Borna disease virus (BDV) [52]. A proposed mechanism for viral factory formation relies on the attainment of high concentrations of N and P proteins, together with RNA molecules [44]. Indeed, when critical concentrations are reached, phase separation occurs, thus bringing virion components into proximity and facilitating their proper assembly, while at the same time conferring the ability to the virus to evade the cell defensive mechanisms. 

Beyond members of the *Mononegavirales* order, a growing number of studies provide evidence for the liquid nature of viral factories from an expanding number of viruses, including members of positive-stranded RNA (+ssRNA) virus families (i.e., Zika and Dengue viruses, ZIKV and DENV, [53] and SARS-CoV-2 [54,55]), double-stranded RNA (dsRNA) virus families (i.e., Rotaviruses [56] and Birnaviruses [57]), and segmented, single-stranded negative-sense RNA virus families (i.e., influenza A virus, IAV, [58]), as well as members of the *Retroviridae* family (i.e., Human immunodeficiency virus 1 (HIV-1) [59,60]) and some DNA viruses (i.e., Herpes simplex virus 1 (HSV-1) [61,62] and adenovirus [41]). In line with the well-documented relationship between intrinsic disorder and LLPS propensity, in the majority of examples cited above, the viral proteins engaged in the formation of these liquid replication compartments were shown to encompass IDRs [35,43], and in a few cases, a clear link between intrinsic disorder and LLPS was established (for examples see [31,49,50,63]). 

Viruses can either give rise de novo to biomolecular condensates, using parallel strategies to cellular systems [34], or interfere with existing ones, especially SGs and P bodies, which are involved in stress signaling and cellular defense mechanisms (for a recent review see [64]). An even more devious use of condensation mechanisms can be hypothesized, namely the exploitation of the intrinsic condensation properties of host proteins to form viral factories. This is suggested by the observation that host cell proteins are also found in viral factories and that, for some of these, intrinsic scaffolding capacity is known. In this work, we have focused on a set of eukaryotic proteins found either within viral factories or within cellular MLOs into which viral proteins partition. We will refer to both types of compartments as “viral infection-related MLOs” (vir-MLOs). The main goal of this work is to gather predicted and experimental information on cellular proteins recruited to vir-MLOs as a first step towards the future development of a dedicated database. The identification of specific features, such as conformational disorder content, LLPS propensity, and degree of interactivity may enable, in the future, predicting if a cellular protein is recruited to vir-MLOs, as well as its functional role as potential *(co)driver* or *client/regulator*. 

## 2. Results

### 2.1. Data Set Generation and Global Disorder Analysis of the Selected Eukaryotic Proteins

The data set of cellular vir-MLOs proteins was generated by selecting cellular proteins that are either recruited to virus-specific condensates or found in cellular MLOs into which viral proteins colocalize. The use of these strict criteria for selecting target proteins for our analysis led to a relatively small data set, encompassing 19 proteins (Table 1). The small size of the data set reflects the hitherto limited number of published studies reporting examples of interactions between cellular and viral proteins within phase-separated compartments. 

One should, however, keep in mind that there are a number of additional potential interactors, including essentially all proteins found in SGs and/or proteins of the NF-κB pathway shown to interact with various viral proteins known to form liquid condensates (as, for instance, the SARS-CoV-2 nucleocapsid, NC, protein) [65,66]. However, if the interaction and the functional outcome have been documented, no data are available supporting the evidence that the interaction occurs within liquid compartments. Likewise, kinases and phosphatases are expected to be client proteins of IBs formed by MeV N and P proteins or by the SARS-CoV-2 NC protein, as phosphorylation events are known to impact the material properties of the liquid condensates formed by these viral proteins [48,67]. Unfortunately, the specific kinases and phosphatases involved in these PTMs and co-localizing within these IBs have not been identified yet. Since we were interested in analyzing cell proteins genuinely interacting with viral proteins within phase-separated condensates, we chose not to incorporate these proteins in the data set. Indeed, although incorporation of the latter would have resulted in an enlarged data set, it would also have jeopardized the analysis.

**Table 1:** Eukaryotic proteins recruited to MLOs related to viral infection.

Cellular Protein	Organism UniProt ID	Type of Condensate	Virus	Known to Phase Separate	PPIDR (PONDR^®^ VSL2)	IDRs (PONDR^®^ VSL2)	MoRFs	FuzDrop p_LLPS_ Status DPRs	PSP Score	DisProt Entry # (IDRs)	Ref.
FUS ^1^	*Homo sapiens* P35637	Cellular MLO	SARS-CoV-2	YES	90.68	1–286 314–315 330–347 356–526	1–19 33–61 75–83 111–165 175–196 205–212 231–240 257–268 285–312 347–375 423–428 432–446 478–486 489–512	0.9999 Driver 1–294 360–437 443–526	0.99	DP01102 (1–507)	[66]
MAVS ^2^	*Homo sapiens* Q7Z434	Viral IBs	RSV	NO	79.26	1–6 91–508 537–540	123–148 156–268 277–290 294–325 338–377 397–410 416–449 463–479	0.9996 Driver 81–456 470–513	0.58	none	[68]
hnRNPA2 ^3^	*Homo sapiens* P22626	Cellular MLO	SARS-CoV-2	YES	69.97	1–21 57–62 72–108 120–126 148–152 183–353	65–71 155–160 168–177 205–212 238–244	0.9808 Driver 1–12 187–353	0.99	DP01109 (190–341)	[66]
p53 ^4^	*Homo sapiens* P04637	Viral replication foci	HPV (human papilloma virus)	YES	68.19	1–107 165–166 168–189 222–224 260–393	11–57 106–115 132–141 232–239 251–258 265–277 322–355 363–387	0.9848 Driver 1–24 28–108 277–337 341–393	0.94	DP00086 (1–93, 291–312, 361–393)	[69]
G3BP2 ^5^	*Homo sapiens* Q9UN86	Cellular MLO	SARS-CoV-2	YES	64.94	1–7 43–53 100–106 127–331 400–482	90–97 109–115 119–145 168–192 207–224 232–281 323–338 348–358 371–379 387–399 436–448 456–482	0.9976 Driver 130–325 399–482	0.94	none	[70]
p65 ^6^	*Homo sapiens* Q04206	Viral IBs	RSV	NO	64.61	1–4 14–92 169–175 257–457 487–551	1–11 31–41 62–76 98–103 110–118 285–290 305–317 350–380 398–414 433–483 492–504 523–551	0.9487 Driver 37–71 77–96 309–355 367–441 503–526	0.83	DP00085 (428–551)	[71]
G3BP1 ^7^	*Homo sapiens* Q13283	Cellular MLO	SARS-CoV-2	YES	63.95	1–7 37–52 139–342 352–356 401–466	123–143 165–193 206–280 304–311 337–348 355–369 376–386 395–406 435–466	0.9937 Driver 135–339 405–466	0.49	none	[70]
TDP-43 ^8^	*Homo sapiens* Q13148	Cellular MLO	SARS-CoV-2	YES	57.25	1–23 79–98 137–143 176–195 197–197 199–207 258–414	28–35 245–255 311–342 380–387 397–402	0.8981 Driver 251–414	0.98	DP01108 (263–414)	[66]
NTF2 ^9^	*Nicotiana benthamiana* Q84JH2 This is a homolog from *Arabidopsis thaliana*	Cellular MLO	Pea enation mosaic virus 2 (PEMV2)	YES	52.18	1–8 82–87 141–144 160–174 177–315 392–458	173–179 197–202 233–257 276–284 292–299 318–334 360–365 424–429 450–458	0.7408 Driver 184–200 213–312 396–451	0.96	none	[72]
MDA5 ^10^	*Homo sapiens* Q9BYX4	Viral IBs	RSV	NO	37.27	1–8 98–110 153–164 192–311 347–356 425–430 466–477 493–500 524–553 568–568 570–576 585–598 631–718 757–774 824–825 865–896 995–999 1022–1025	233–239 243–276 324–329 503–515	0.6164 Driver 239–308 489–499 566–595 641–661	0.11	none	[68]
Fib2 ^11^	*Nicotiana benthamiana* B7VCB9	Cellular MLO	PEMV2	YES	35.99	1–83 116–119 277–292 305–314	1–16 29–44 72–90	0.3248 Client 1–86	0.99	none	[72]
FAK1 ^12^	*Homo sapiens* Q05397	Negri bodies (viral IBs)	RABV	YES	34.32	1–32 107–113 188–194 306–313 363–418 576–580 638–647 660–751 771 785–926 941–949 1014–1018 1046–1052	1–7 36–42 341–359 652–660 672–681 698–705 726–769 792–808 830–845 848–867 882–905 922–938 958–965	0.6417 Driver 1–35 683–736 743–767 812–922	0.07	DP03144 (565–583)	[31,73]
TIAR1 ^13^	*Homo sapiens* Q01085	Viral IBs	Ebola Virus (EBOV)	YES	32.27	1–8 85–94 132–138 174–201 280–284 303–309 320–375	140–148 205–213 339–345	0.5857 Client 1–16 174–185 311–375	0.36	none	[74]
HSP70-1A ^14^	*Homo sapiens* P0DMV8	Negri bodies (viral IBs)	RABV	NO	29.64	1–5 100–106 153–158 230–230 243–286 361–363 491–572 588–598 611–641	476–486 541–550 573–584 602–614	0.3828 Client 548–569 606–641	0.12	DP02353 (229–306)	[31]
RAB11 ^15^	*Homo sapiens* P62491	Viral assembly compartment	IAV	NO	24.54	1–6 24–25 35–40 178–216	166–176 210–216	0.1679 Client 176–209	0.02	none	[58]
PP1 ^16^	*Homo sapiens* P62136	Viral IBs	RSV	NO	19.70	1–10 14–14 18–30 179–183 213–216 299–330	Not found	0.1692 Client 300–330	0.04	none	[75]
CAD ^17^	*Homo sapiens* P27708	IBs (viral factories)	EBOV	NO	19.51	1–5 118–155 222–232 239–243 319–320 338–343 360–402 405–406 408–412 525–544 567–573 689–696 797–804 852–860 1041–1042 1117–1119 1156–1159 1287–1289 1325–1326 1401–1403 1540–1545 1649–1665 1690–1708 1711–1711 1713–1727 1807–1923 1972–1986 1988–2009 2122–2135 2177–2177 2190–2196 2223–2225	327–333 346–357 1675–1684 1709–1718 1768–1805 1841–1864 1874–1891 1903–1930 2161–2166	0.2011 Client 379–392 1812–1923 2043–2053	0.11	DP01024 (1822–1846)	[76]
MAPK14 ^18^	*Homo sapiens* Q16539	Viral IBs	RSV	NO	13.89	1–7 176–178 247–256 313–327 342–345 350–360	Not found	0.1119 Not related to LLPS	0.00	None	[77]
OGT ^19^	*Homo sapiens* O15294	Viral IBs	RSV	NO	12.83	1–17 34–36 106–106 305–308 377–377 405–405 435–445 499–512 541–553 564–579 683–693 759–770 814–819 908–911 1031–1034 1039–1046	Not found	0.1567 Client 1–15 758–772	0.10	None	[77]

^1^ RNA-binding protein fused in sarcoma (FUS). SARS-CoV-2 N protein partitions as a client in cellular MLOs containing TDP-43, FUS, and hnRNPA2. ^2^ Mitochondrial antiviral signaling protein (MAVS). RSV N protein likely interacts with MDA5, is in close proximity to MAVS, and sequesters these proteins within IBs, which results in the attenuation of the interferon (IFN) response. ^3^ Heterogeneous nuclear ribonucleoprotein A2/B1 (hnRNPA2). SARS-CoV-2 N protein partitions as a client in cellular MLOs containing TDP-43, FUS, and hnRNPA2. ^4^ p53. HPV E2 protein partitions as client in chromatin-associated foci containing p53. Co-condensation of p53 and E2 in the nucleus results in modulation of HPV gene function. ^5^ Ras-GAP SH3 domain-binding protein 2 (G3BP2). SARS-CoV-2 N protein associates with the host SG-nucleating proteins G3BP1 and G3BP2. ^6^ NF-κB complex p65 subunit (p65). In both human and bovine RSV-infected cells, the p65 subunit of NF-κB is rerouted to perinuclear puncta in the cytoplasm, which correspond to viral IBs where viral RNA replication occurs. Captured p65 is unable to translocate to the nucleus or transactivate a NF-κB reporter following tumor necrosis factor alpha (TNF-α) stimulation, confirming the immune-antagonistic nature of this sequestration. ^7^ Ras-GAP SH3 domain-binding protein 1 (G3BP1). SARS-CoV-2 N protein associates with the host SG-nucleating proteins G3BP1 and G3BP2. ^8^ TAR DNA-binding protein 43 (TDP-43). SARS-CoV-2 N protein partitions as a client in cellular MLOs containing TDP-43, FUS, and hnRNPA2. ^9^ Nuclear transport factor 2 (NTF2) family protein (a plant G3BP-like activator of SGs). The p26 movement protein from PEMV2 partitions with cellular proteins fibrillarin (Fib2) and G3BP. p26 partitions as a client protein in the nucleolus and in SGs. ^10^ Melanoma differentiation-associated protein 5 (MDA5). MDA5 is sequestered into IBs, likely through interaction with the RSV N protein. ^11^ Fibrillarin (Fib2). p26 movement protein from PEMV2 partitions with cellular proteins fibrillarin (Fib2) and G3BP. Viral p26 protein partitions as client protein in the nucleolus and in SGs. ^12^ Focal Adhesion Kinase 1 (FAK1). FAK1 is recruited to NBs through interaction with the RABV P protein.^13^ Nucleolysin TIAR1. TIAR1 is a SG marker. During infection, it co-localizes with EBOV VP35 in cytoplasmic aggregates, which are likely to be viral IBs. ^14^ Heat shock 70 kDa protein 1A (HSP70-1A). HSP70-1A is recruited to NBs through interaction with the RABV P protein.^15^ GTPase RAB11 (RAB11). In uninfected cells, RAB11 is the master regulator of the endocytic recycling compartment (ERC), a system used for delivering endocytosed material and specific cargos from the trans-Golgi network (TGN) to the cell surface. RAB11 is redistributed during infection, changing from discrete to enlarged puncta that match sites of clustered vesicles positive for RAB11 and vRNPs (viral ribonucleoproteins), constituting vRNP hotspots. ^16^ Phosphatase PP1 (PP1). PP1 is recruited to IBs through interaction with the RSV P protein. The P protein recruits the viral transcription factor M2-1 to viral IBs (independently of its phosphorylation state). M2-1 is dephosphorylated by the P-PP1 complex. M2-1 needs to be dephosphorylated in order to be recruited into IBAGs, which are a substructure of IBs where viral transcription mainly takes place. ^17^ CAD is a key component in the pathway of de novo synthesis of pyrimidines. CAD directly interacts with the EBOV N protein, with N being sufficient to recruit CAD into IBs via the glutaminase (GLN) domain of the latter. ^18^ Mitogen-activated protein kinase 14 (MAPK14) or p38MAPKα is a key regulator of cellular inflammatory and stress responses. RSV induces the sequestration of p38MAPKα in IBs resulting in the accumulation of a downstream signaling substrate, MAPK-activated protein kinase 2 (MAPK2). ^19^ O-GlcNAc transferase (OGT). OGT catalyzes the addition of OGN to target proteins to regulate cellular processes, including signal transduction, transcription, translation, and stress response. During RSV infection, OGT is sequestered in RSV IBs, causing the impairment of SGs formation, thus triggering suppression of the antiviral cellular response. Detailed information on the structural organization and main biological functions of the proteins of the data set can be found in Section 2.5.

As shown in Table 1, many, though not all, proteins within our data set are characterized by high levels of predicted disorder. Proteins in Table 1 are arranged by their intrinsic disorder status in the form of the predicted percentage of intrinsically disordered residues (PPIDR, i.e., percent of residues with disorder scores exceeding 0.5), evaluated by PONDR^®^ VSL2 [78], a highly accurate disorder predictor [79]. It is noteworthy that for some of the proteins (i.e., FUS, hnRNPA2, p53, p65, TDP-43, FAK1, HSP701A, and CAD), disorder has already been experimentally validated, and for them we reported the amino acid boundaries as annotated in the DisProt database of IDPs [80,81,82]. For most of the proteins, including the most disordered ones such as FUS, MAVS, hnRNPA2, and p53, at least partial structural information is available (see Supplementary Table S1). Note, however, that the availability of structural data for a portion of the polypeptide chain is not, per se, proof that the region is ordered, as structural data for the proteins with the highest predicted disorder (i.e., FUS, MAVS, hnRNPA2, p53, and G3BP1) are often based either on crystal structures of complexes with partners/ligands or on NMR solution structures that feature a high structural heterogeneity (see Supplementary Table S1). Table 1 also features information on their experimentally documented ability to undergo LLPS whenever available. As shown in Table 1, a number of these cellular proteins have intrinsic phase-separating abilities, thus raising the question as to whether they may serve as *(co-)drivers* in the condensation processes that give rise to viral factories. Such a mechanism would reflect a long co-evolutionary pathway between the host cell and the virus and would identify new promising targets for antiviral strategies. 

**Figure 1:**
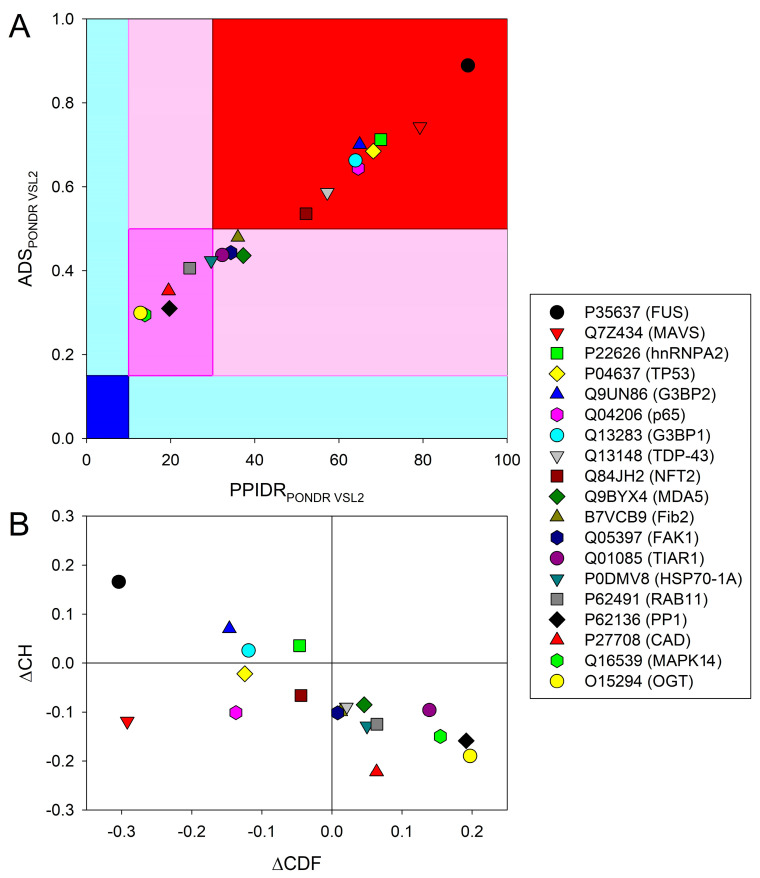
Global intrinsic disorder predisposition of 19 eukaryotic proteins recruited to the viral infection-related MLOs. (**A**). Analysis of query proteins based on the average disorder score (ADS) and percent of predicted disordered residues (PPIDR) as evaluated by PONDR^®^ VSL2. High values of each parameter correspond to high disorder propensities. Different color blocks indicate regions containing proteins with different levels of order, where mostly ordered, moderately disordered, and mostly disordered proteins are located within blue, pink, and red blocks, respectively. If the two parameters (ADS and PPIDR) agree, the corresponding part of the background is shown by a dark color (blue or pink), whereas light blue and light pink reflect areas in which only one of these criteria applies. (**B**). CH–CDF plot for 19 eukaryotic proteins recruited to the viral infection-related MLOs.

It is commonly recognized that PPIDR can be used for intrinsic disorder status classification of query proteins, where proteins with PPIDR < 10%, 10% ≤ PPIDR < 30%, and PPIDR ≥ 30% are considered highly ordered, moderately disordered, and highly disordered, respectively [83]. Based on these criteria, 13 proteins analyzed here are highly disordered and 6 are moderately disordered (see Table 1). In addition to classifying proteins based on their PPIDR values, the average disorder score (ADS, calculated as the sum of disorder scores divided by the number of protein residues) in query proteins can be used for their classification as well. Here, proteins with ADS < 0.15, 0.15 ≤ ADS < 0.5, and ADS ≥ 0.5 are considered highly ordered, moderately disordered and highly disordered, respectively. Figure 1A shows the correlation between ADS and PPIDR values for the 19 eukaryotic proteins analyzed in this study and supports the notion that all these proteins are at least moderately disordered. The most disordered proteins (i.e., those located within the red region) are human FUS, MAVS, hnRNPA2, p53, G3BP2, NF-κB complex p65, G3BP1, TDP-43, and G3BP from *Nicotiana benthamiana* (see also Table 1).

Another approach to look at the global intrinsic disorder predisposition of query proteins is based on the analysis of their distribution within the charge-hydropathy (CH)–cumulative distribution function (CDF) phase space, where ordered proteins, molten globular/hybrid proteins containing sizable levels of order and disorder, and native coils or native pre-molten globular proteins are located within the lower right, lower left, and upper left quadrant, respectively [84]. Figure 1B shows that FUS, G3BP2, G3BP1, hnRNPA2, and p53 are predicted to be highly disordered, whereas MAVS, NF-κB complex p65, and G3BP-like SG activator in plants are predicted to be native molten globules or hybrid proteins with sizable ordered and disordered regions, with the remaining proteins being grouped within the lower right quadrant, confirming that these proteins are mostly ordered or contain large ordered domains. Therefore, although many proteins in our data set are disordered, some of them are not, clearly indicating that enrichment in disorder is not a strict prerequisite for a protein to be recruited to vir-MLOs.

### 2.2. Per-Residue Disorder Predictions and Interactivity Analysis of the Eukaryotic Proteins Recruited to Vir-MLOs

**Figure 2:**
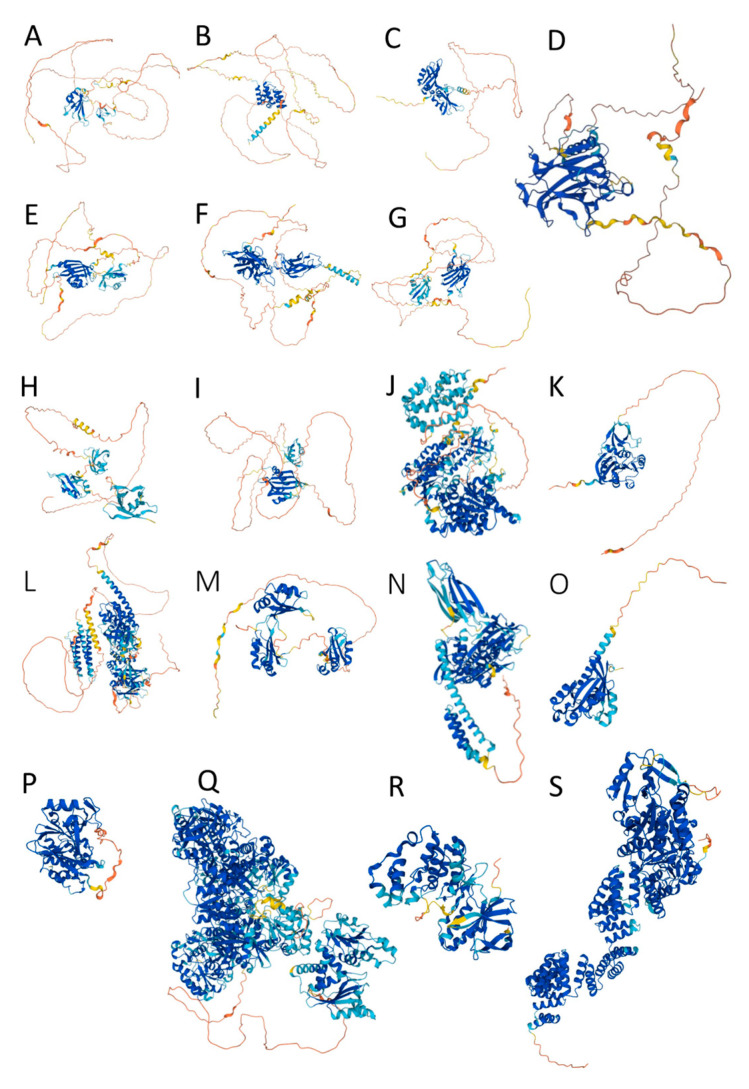
Three-dimensional structures modeled for query proteins by AlphaFold2 as retrieved from UniProt. (**A**). FUS (UniProt ID: P35637); (**B**). MAVS (UniProt ID: Q7Z434); (**C**). hnRNPA2 (UniProt ID: P22626); (**D**). p53 (UniProt ID: P04637); (**E**). G3BP2 (UniProt ID: Q9UN86); (**F**). p65 (UniProt ID: Q04206); (**G**). G3BP1 (UniProt ID: Q13283); (**H**). TDP-43 (UniProt ID: Q13148); (**I**). G3BP (UniProt ID: Q84JH2); (**J**). MDA5 (UniProt ID: Q9BYX4); (**K**). Fib2 (UniProt ID: B7VCB9); (**L**). FAK1 (UniProt ID: Q05397); (**M**). TIAR1 (UniProt ID: Q01085); (**N**). HSP701A (UniProt ID: P0DMV8); (**O**). RAB11 (UniProt ID: P62491); (**P**). PP1 (UniProt ID: P62136); (**Q**). CAD (UniProt ID: P27708); (**R**). MAPK14 (UniProt ID: Q16539); (**S**). OGT (UniProt ID: O15294). Note that since the predicted structure of Fib2 from *N. benthamiana* is not available in UniProt, shown is the structure of Fib2 from *Arabidopsis thaliana* (UniProt ID: Q94AH9). Structural elements are colored based on the confidence of structure prediction by AlphaFold2 (in cyan and dark blue structures predicted with high to very high confidence, in yellow and orange segments predicted with low to very low confidence, and expected to be disordered).

The structures of the proteins in our data set were predicted with AlphaFold2 and the resulting structural models are shown in Figure 2. 

**Figure 3:**
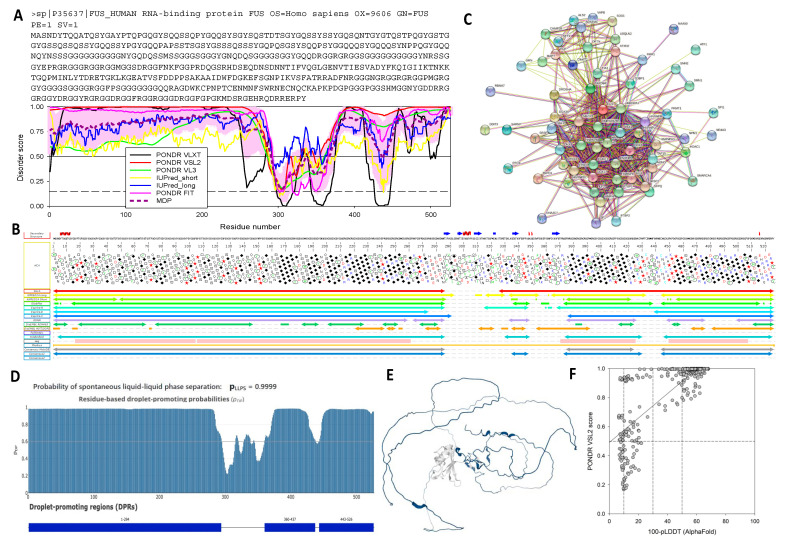
Functional disorder in RNA-binding protein FUS. (**A**). Amino acid sequence (top) and RIDAO-generated disorder profile (bottom). RIDAO aggregates the results from a number of well-known disorder predictors: PONDR^®^ VLXT (black line), PONDR^®^ VLS2 (red line), PONDR^®^ VL3 (green line), IUPred2_short (yellow line), IUPred2_long (blue line), and PONDR^®^ FIT (pink line). Mean disorder prediction (MDP), which is calculated by averaging the outputs of these six predictors, is shown by a thick dashed dark-pink line and the corresponding error distribution is shown by a light pink shadow. The outputs of the evaluation of the per residue disorder propensity by these tools are represented as real numbers between 0 (ideal prediction of order) and 1 (ideal prediction of disorder). A threshold of ≥0.5 is used to identify disordered residues and regions in query proteins. Solid and dashed horizontal lines at disorder scores 0.5 and 0.15 correspond to the disorder and flexibility thresholds. (**B**). MeDor output. MeDor aggregates the results of eight predictors implemented in MobiDB-lite 3.0 (GlobPlot, ESpritz-N, ESpritz-D, ESpritz-X, IUPRED2A Long, IUPRED2A Short, DisEMBL REM465, DisEMBL hotloops) along with those provided by DorA, MoreRONN (RONN), FoldIndex, and FoldUnfold. Regions predicted to be disordered by the various predictors are indicated by double arrows with a color code corresponding to that of the corresponding predictors as indicated on the left, while regions predicted to be ordered are shown as grey dashed lines. The MeDor output displays the MobiDB-lite consensus (Consensus MobiDB, grey double arrow) and two other types of global consensus: Consensus 1 (dark blue double arrow), which corresponds to regions predicted to be disordered by more than half of the implemented predictors, and Consensus 2, which corresponds to regions consistently predicted to be disordered by all the implemented predictors (none in this case). The MeDor output also displays low-complexity regions (pink bar) and transmembrane regions (none in this case). The HCA plot (see Appendix A) is shown below the amino acid sequence. Predicted α-helices (red) and β-strands (blue arrows) are shown above the sequence. (**C**). STRING-generated PPI network. The minimum required interaction score was set to 0.700 (high confidence). Number of nodes 67; number of edges 523; average node degree: 15.6; avg. local clustering coefficient: 0.77; expected number of edges: 97; PPI enrichment *p*-value: < 1.0 × 10^−16^. The corresponding interactive map of FUS-centered PPI network can be found at: https://string-db.org/cgi/network?taskId=bvsmgavPysJb&sessionId=bxLUgl7X6fKS (last accessed on 2 January 2023). (**D**). FuzDrop output. (**E**). AlphaFold2 structure with FuzDrop annotations. (**F**). Per residue correlation plot of disorder score (as provided by PONDR VLS2) *versus* (100-pLDDT), where pLDDT is the corresponding AlphaFold2 confidence score.

Although AlphaFold2 models can in no way be regarded as accurate descriptions of IDPs/IDRs, the latter being only describable as conformational ensembles [85], they are still useful because they provide a convenient cartoon representation that graphically enables conveying and capturing the conformational heterogeneity of IDRs. AlphaFold2 model reliability is expressed by the parameter pLDDT, which takes a value between 0 and 100 assigned to each residue. Low pLDDT scores (i.e., <50) have been shown to provide a good measure of residue-wise disorder [79,86]. For each of the protein in our data set, we generated a per-residue correlation plot between the predicted disorder score (as provided by PONDR^®^ VSL2) and the AlphaFold confidence score (see Figure 3 and Supplementary Figures S1–S17). Results indicate that the correlation between these two tools is moderate, which is not surprising, as it was already pointed out that AlphaFold is not the most accurate predictor of disorder. However, for all proteins, when looking at all data points, there is an overall positive correlation between the two predictors, indicating that, on average, residues with higher PONDR^®^ VSL2 propensity have lower AlphaFold2 confidence.

Visual analysis of AlphaFold2 structural models revealed that all proteins in this study are enriched in disorder and even the most ordered members of the data set, i.e., RAB11, PP1, CAD, P38MAPKα, and OGT, still possess noticeable levels of disorder, judged from the presence of multiple IDRs (see also Table 1). 

Next, we generated disorder profiles for all query proteins using various computational platforms, such as the MeDor disorder prediction metaserver [87], the web crawler RIDAO, which aggregates outputs of six per residue disorder predictors (i.e., PONDR^®^ VLXT [88], PONDR^®^ VL3 [89], PONDR^®^ VLS2 [90], PONDR^®^ FIT [91], IUPred2 (Short), and IUPred2 (Long) [92,93]) and generates mean disorder profiles based on the averaged outputs of individual predictors and the D^2^P^2^ database [94]. The RIDAO profile of the well-known, highly disordered, and phase-separating FUS protein is shown as an example in Figure 3A, while the RIDAO profiles of all the other proteins analyzed in this study are shown in the Supplementary Materials. Figure 3B shows the MeDor output for FUS as an example, while the MeDor profiles obtained for all the other proteins in the data set are shown in the Supplementary Materials.

**Figure 4:**
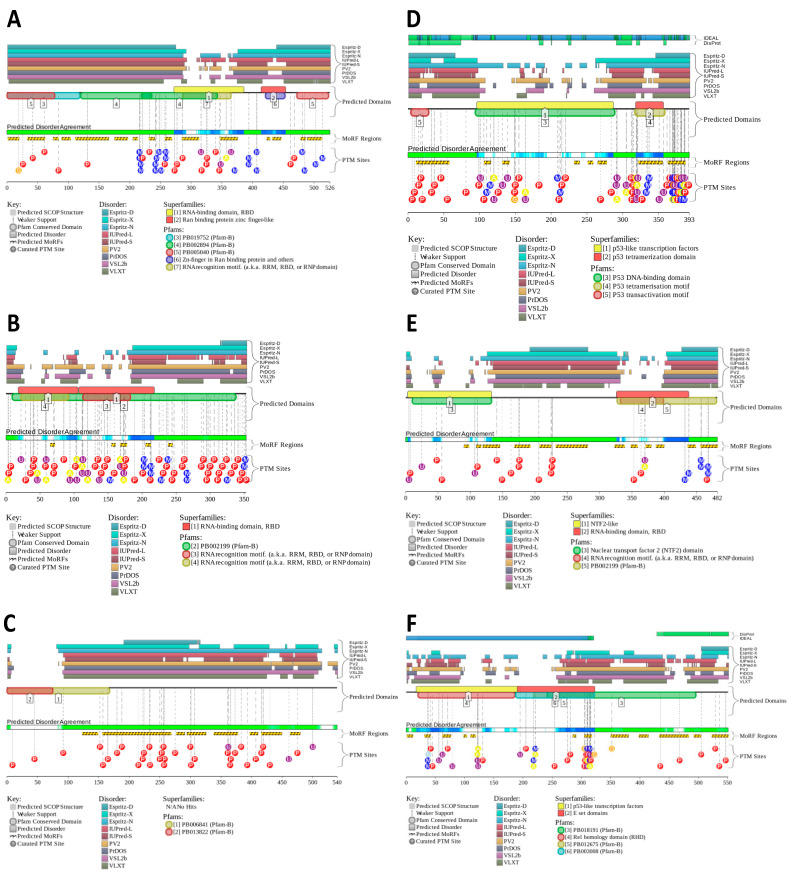
Functional disorder profiles generated for FUS (**A**), hnRNPA2 (**B**), MAVS (**C**), p53 (**D**), G3BP2 (**E**), and p65 (**F**) by the D^2^P^2^ platform, which is a database of predicted disorder for proteins from completely sequenced genomes (https://d2p2.pro/) (last accessed on 9 December 2022). Here, the outputs of IUPred, PONDR^®^ VLXT, PrDOS, PONDR^®^ VSL2, PV2, and ESpritz are used to show disorder predispositions of query proteins by differently colored bars. Consensus between these nine disorder predictors is shown by the blue-green-white bar, whereas location of various PTMs is shown by differently colored circles. D^2^P^2^ also shows positions of conserved functional SCOP domains as predicted by the SUPERFAMILY predictor. Positions of these functional domains are shown below the outputs of the nine disorder predictors. Functional disorder profile also includes information on the location of predicted disorder-based binding sites (MoRF regions) identified by the ANCHOR algorithm and various PTMs assigned using the outputs of PhosphoSitePlus.

Figure 4 shows the functional disorder profiles generated by the D^2^P^2^ platform for the six most disordered proteins, namely FUS, MAVS, hnRNPA2, p53, G3BP2, and p65. The analysis clearly shows that all these proteins make extensive use of intrinsic disorder for functional purposes, as they all contain multiple disorder-based binding sites, known as molecular recognition features (MoRFs) i.e., short disordered regions that fold upon interaction with binding partners [95,96]. Furthermore, all these proteins contain numerous PTM sites, which is a well-documented feature of IDRs [20]. Amino acid boundaries of MoRFs were obtained from the D^2^P^2^ output for all proteins analyzed in this study except for Fib2, for which D^2^P^2^ does not have data and whose MoRFs were predicted using ANCHOR2 [97]. Table 1 shows that, with the exception of PP1, P38MAPKα, and OGT, all these proteins contain multiple MoRFs, with some of these proteins (e.g., FUS) utilizing almost their entire sequence as a disorder-based binding platform (see Table 1 and Figure 4A). 

**Figure 5:**
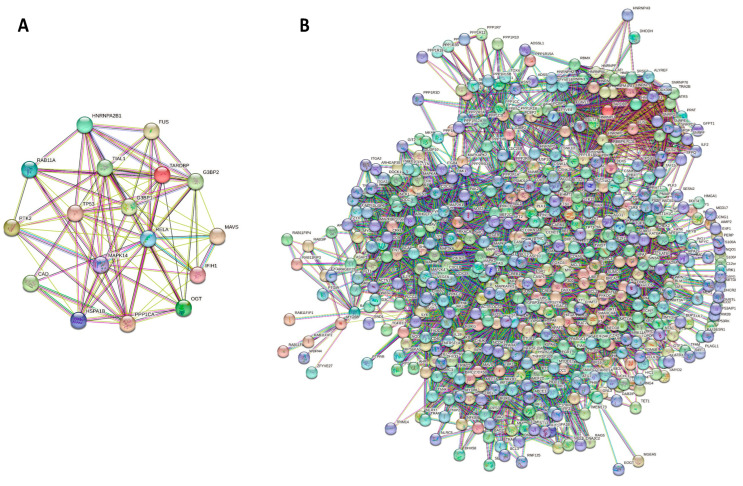
Internal (**A**) and external PPI networks (**B**) of 17 human proteins recruited to MLOs related to viral infection. Networks were generated by STRING (http://string-db.org/) (accessed on 9 December 2022), which creates a network of associations based on predicted and experimentally validated information on the interaction partners of a protein of interest. In the corresponding network, the nodes correspond to proteins, whereas the edges show predicted or known functional associations. Seven types of evidence are used to build the corresponding network and are indicated by the differently colored lines: a green line—neighborhood evidence; a red line—presence of fusion evidence; a purple line—experimental evidence; a blue line—co-occurrence evidence; a light blue line—database evidence; a yellow line—text mining evidence; and a black line—co-expression evidence. Interactive maps of internal and external PPI networks can be found at: https://string-db.org/cgi/network?taskId=bUhEOdNrRfH9&sessionId=bxLUgl7X6fKS (last accessed on 2 January 2023) and https://string-db.org/cgi/network?taskId=beMc7yqPboZj&sessionId=bxLUgl7X6fKS (last accessed on 2 January 2023).

To test the possibility of reciprocal interactions between the 17 human proteins selected in this study, we used the STRING platform to generate internal protein–protein interaction (PPI) networks in these proteins. The results of this analysis are presented in Figure 5A, which shows 86 interactions between the 17 human proteins, with an average number of interactions per protein—i.e., the average node degree—of 10.1. Gene Ontology (GO) term enrichment analysis was applied to this network and the most relevant data are shown in Table 2. Taken together, these data provide a measure of the involvement of these proteins in rather diverse metabolic and cellular signaling pathways.

Next, we generated an external PPI network for these 17 human proteins and found that they, as a group, form a very dense and highly populated network (Figure 5B), encompassing at least 517 proteins linked by 4834 interactions (with an average node degree of 18.7). This extensive interaction network was again analyzed to identify enriched GO terms, and the results are collected in Table 3. The high interactivity of the 17 human proteins with the rest of the human proteome is also confirmed by STRING-based analysis obtained for each protein (see Figure 3C and Supplementary Materials).

### 2.3. LLPS Propensities of the Eukaryotic Proteins Recruited to Vir-MLOs

**Figure 6:**
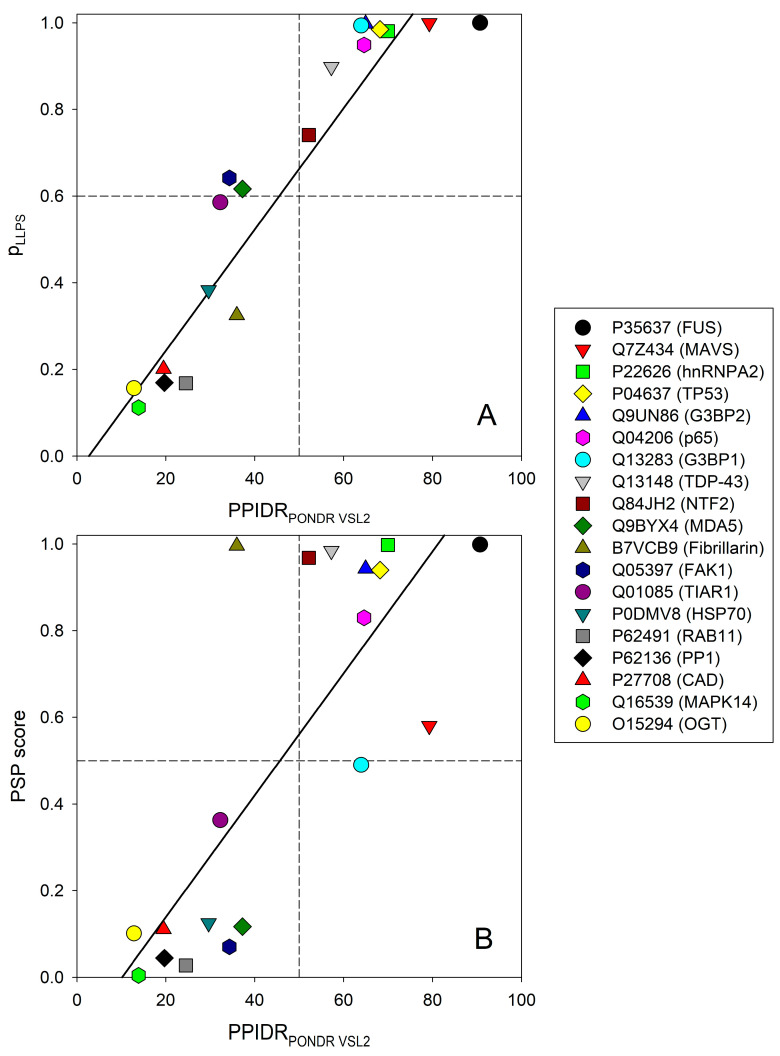
Correlations between protein intrinsic disorder content, PPIDR_PONDR VSL2_, as evaluated by PONDR^®^ VSL2, and probability of spontaneous liquid–liquid phase separation, p_LLPS_, evaluated by FuzDrop (**A**) or phase separation protein score, PSP score, evaluated by PSPredictor (**B**).

To assess the inherent propensity of the set of target proteins to undergo LLPS, we used the FuzDrop and the PSPredictor web servers. Obtained results are shown in Table 1, Figure 3D,E, and Supplementary Materials. Next, we looked at the correlation between intrinsic disorder and propensity for phase separation as evaluated by FuzDrop (Figure 6A) and PSPredictor (Figure 6B) (see also Table 1). 

This analysis revealed that the LLPS propensities of the proteins are related to their intrinsic disorder level according to the following equations:

pLLPS = --0.0372(± 0.079) + 0.0140(± 0.0015) × PPIDR_PONDR VSL2_ (R^2^ = 0.8192) (1)

PSP score = --0.14(± 0.13) + 0.0141(± 0.0025) × PPIDR_PONDR VSL2_ (R^2^ = 0.6257) (2)

Since it is commonly accepted that intrinsic disorder represents one of the key drivers of phase separation [14,15] and because LLPS predictions are also based on the structural disorder status of proteins, it might seem obvious, if not recursive, to assess the correlation between disorder and the condensation propensity of the 19 proteins in our data set. It should be noted that our set contains many proteins for which the ability to form LLPS is experimentally known (see Table 1 and Appendix A), and the comparison of the two LLPS predictors reveals a different ability to capture phase separation behavior, at least in the peculiar context of vir-MLOs. This different performance seems to be influenced by the degree of disorder. Although the pLLPS and PSP scores are both reasonably well related to the PPIDR disorder score as provided by VSL2, the correlation between these two measures of LLPS predisposition was much poorer, being formalized as follows:

pLLPS = 0.283(± 0.089) + 0.64(± 0.14) × PSP score (R^2^ = 0.5407) (3)

Figure 6 and Table 1 show that the outputs of these two LLPS predictors mostly agree for proteins with the highest and lowest propensity for phase separation, whereas agreement is less prominent for proteins with intermediate to moderate propensities for LLPS. Although eleven proteins were identified as LLPS drivers by FuzDrop, PSPredictor found nine phase-separating proteins, and only one protein, i.e., fibrillarin from *Nicotiana benthamiana* (UniProt ID: B7VCB9), was predicted to be a phase-separating protein by PSPredictor but not an LLPS driver by FuzDrop (see Table 1 and Figure 6).

### 2.4. Relationships between Disorder Content and Role in LLPS

**Figure 7:**
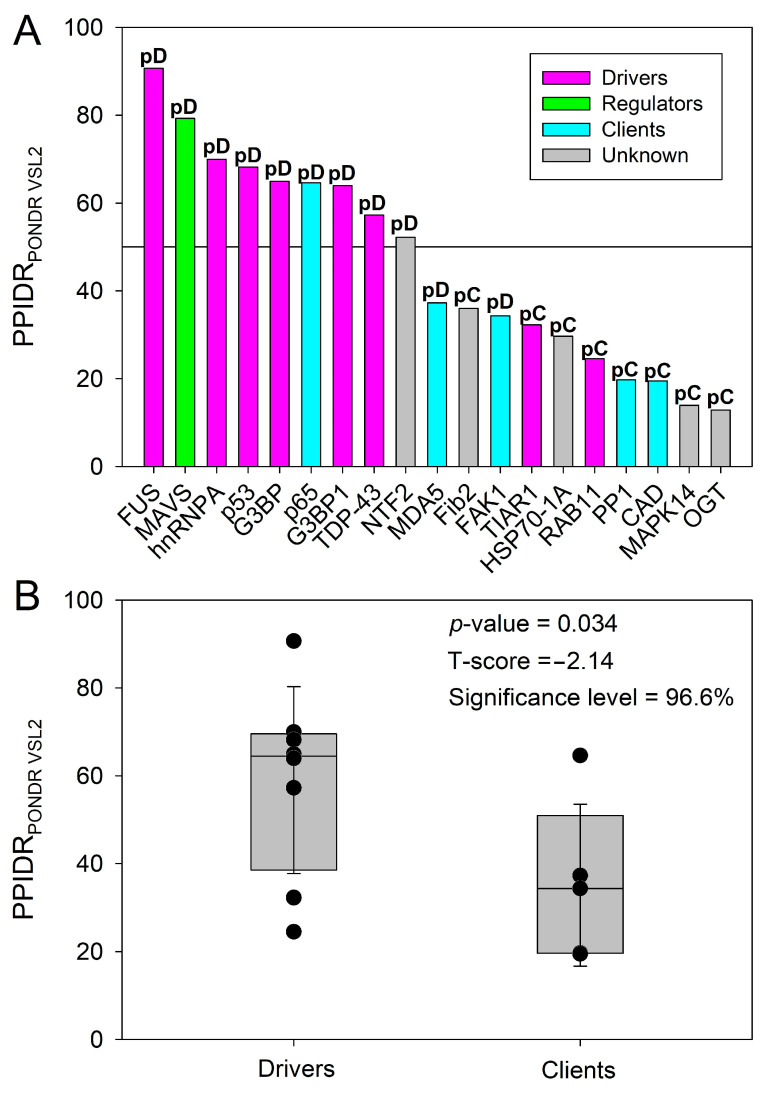
(**A**) Correlations between protein intrinsic disorder content, PPIDR_PONDR VSL2_, evaluated by PONDR^®^ VSL2, and the experimentally validated role in LLPS for each of the 19 proteins in our data set, as retrieved by browsing MLOsMetaDB (https://mlos.leloir.org.ar, accessed on 4 January 2023). The continuous horizontal line corresponds to a 50% content in disorder. Proteins predicted as *drivers* or as *clients* by FuzDrop (see Table 1) are marked as **pD** and **pC**, respectively. (**B**) Statistical analysis based on Student’s *t*-test. Note that both Normality and Equal Variance tests were successfully passed, indicating the suitability of the *t*-test to assess statistically significant differences. The analysis was conducted using the online Georgiev G.Z., “P-value Calculator” (https://www.gigacalculator.com/calculators/p-value-significance-calculator.php URL, accessed on 5 January 2023).

In an attempt to ascertain a possible relationship between disorder content and role as *driver* or *client*, we browsed the MLOsMetaDB (https://mlos.leloir.org.ar) [98]. This database integrates and analyzes the information across three LLPS-dedicated databases, namely PhaSePro [99], PhaSepDB [100], and DRLLPS [101]. It provides a consolidated and curated set of experimentally validated phase-separating proteins enabling a customized selection of different sets of proteins based on MLO location, database, and disorder content, among other attributes. Most interestingly for the present study, MLOsMetaDB collates curated experimental information on the role of the various proteins it contains as *drivers*, *clients*, and *regulators* of LLPS. We, thus, browsed the database and searched for entries corresponding to the set of proteins analyzed in this study. For each of them, we plotted their annotated role in the database as a function of their disorder content, estimated by PPIDR_PONDR VSL2_ (Figure 7A). 

Although no data were found for five proteins in our set, the overall results point to a trend whereby the most disordered proteins (i.e., those with a disorder content > 50%) would serve as *drivers*, whereas *clients* and *regulators* would be more ordered (Figure 7A). In Figure 7B, we focused on *drivers* and *clients* and assessed statistical significance between the two groups. Despite the small set size (*n* = 8 for *drivers* and *n* = 5 for *clients*), a statistically significant difference was found between the two data sets (*p* = 0.034, significance level = 96.6% and a *t*-score, i.e., number of standard deviations away from the mean of the *t*-distribution, of −2.14). These results support the hypothesis that disorder content determines the behavior of a given protein as a *driver* or *client*, in agreement with previous conclusions based on the analysis of the much larger data set of MLOsMetaDB (see Figure 4C in [98]). 

### 2.5. Structural Organization and Biological Functions of the Eukaryotic Proteins Recruited to Vir-MLOs

Below we provide a detailed description of the structural organization and main biological functions of each of the eukaryotic proteins considered in this study. We emphasize the connections between biological functions and the various predicted properties as provided by the present study.

#### 2.5.1. Human hnRNPs: FUS, TDP-43, and hnRNPA2

The family of the heterogeneous ribonucleoproteins (hnRNPs) includes several RNA-binding proteins (RBPs) contributing to various aspects of nucleic acid metabolism, such as regulation of alternative splicing, mRNA stabilization, and regulation of transcription and translation [102]. Considered below are three members of this family that are part of the data set used in this study: fused in sarcoma (FUS), TAR DNA-binding protein 43 (TDP-43), and heterogeneous nuclear ribonucleoprotein A2/B1 (hnRNPA2/B1, also indicated as hnRNPA2). It was recently shown that FUS, TDP-43, and hnRNPA2 colocalize within liquid droplets formed by the SARS-CoV-2 nucleocapsid protein (N) and viral RNA [66]. In addition, the low-complexity (LC) domains of these proteins are able to phase separate, and SARS-CoV-2 N partitions in vitro into these phase-separated LC domains [66]. Table 1 shows that the LLPS-prone regions predicted by FuzDrop overlap with or encompass the long IDRs of these proteins, and all these regions contain MoRFs. 

FUS. FUS has multiple biological functions, including RNA processing and regulation of pre-mRNA splicing [103], control and regulation of the transcription of target genes [104], and DNA repair [105]. FUS is the most disordered protein in the data set analyzed in this study (see Table 1 and Figure 1, Figure 2, Figure 3 and Figure 4). It is best known for its association with the pathogenesis of amyotrophic lateral sclerosis (ALS) and frontotemporal lobar degeneration (FTLD) [106]. FUS is present both in the nucleus and the cytoplasm and can shuttle between these two locations. While in the norm its predominant localization is the nucleus of neurons, in ALS and FTLD, FUS is mislocalized to the cytoplasm, where it forms characteristic inclusions [107]. This cytoplasmic mislocalization of mutated FUS, which causes aberrant SG biogenesis, is attributed to the fact that many ALS-associated mutations affect the nuclear localization sequence (NLS), located in the C-terminal region of this protein [107]. Mutated forms of FUS can bind to mRNAs, significantly altering target gene expression [108] and/or alternative splicing [109]. In addition to RNA, FUS can bind to several RNA-binding proteins from the hnRNP family [110]. Strikingly, SGs found in ALS predominantly contain mutated forms of FUS, indicating that pathogenic FUS mutations alter SG biogenesis [111]. 

FUS is a modular protein encompassing various functional modules that either include or are included in at least one MoRF (see Table 1 and Figure 4A). These observations suggest that the high interactability of this protein can be attributed to the presence of disorder-based binding sites. In line with this hypothesis, STRING-generated PPI network centered on FUS shows a dense network that includes 67 proteins connected by 523 interactions (see Figure 3C). In line with the known capability of FUS to form liquid condensates [112], this protein is predicted to have three long regions with high LLPS potential (see Table 1 and Figure 3D,E). 

TDP-43. TDP-43 is endowed with a multitude of functions that are mostly related to RNA processing [113], including the regulation of pre-mRNA splicing, transcriptional regulation, and regulation of processing, stability, and transport of mRNA [114]. Furthermore, TDP-43 is known to be engaged in numerous PPIs (see below), with examples of it most notable binding partners being hnRNPA1, hnRNPA2, hnRNPB1, and FUS [115]. Furthermore, the C-terminal PrLD of this protein plays a crucial role in its self-aggregation [116]. In sporadic ALS, aggregated TDP-43 is found within insoluble protein aggregates in both neurons and glial cells [117]. 

One of the best studied examples of LLPS is provided by the involvement of TDP-43 in the formation of SGs [118]. The association of TDP-43 with SGs is promoted by either direct binding of TDP-43 to specific SG proteins, such as TIA1, or through interactions with RNA [119]. Pathological aggregation of TDP-43 is linked to ALS and FTLD, and cell cultures and pathological brain tissues contain detergent insoluble TDP-43 aggregates and increased levels of TDP-43-containing SGs [119]. TDP-43 is capable of transitioning from soluble droplet-like to solid-like aggregates, with these transitions being implicated in the pathological aggregation and disease development [120]. 

TDP-43 contains four MoRFs indicating the capability of this region to serve as a major “docking port” for its binding partners [121] (see also Table 1 and Supplementary Figure S1). The binding promiscuity of TDP-43 is further witnessed by the highly connected PPI network (average node degree is 29.5) generated for this protein by STRING (see Supplementary Figure S1) that contains 88 proteins connected by 1297 interactions. Furthermore, there are two LLPS-prone regions in human TDP-43 (see Table 1). 

hnRNPA2. hnRNPA2 is a highly and ubiquitously expressed protein involved in numerous biological processes ranging from transcription to pre-mRNA processing, mRNA translation, RNA nuclear export and subcellular location, and control of the stability of mature mRNAs [122]. As such, it regulates the expression of a large number of genes. Mutations in the PrLD of hnRNPA2 are associated with ALS and multisystem proteinopathy [123]. These disease-associated mutations were shown to enhance the formation of cytoplasmic inclusions, the intrinsic tendency of this protein to form self-seeding fibrils, and the incorporation of hnRNPA2 into SGs [123,124]. 

Table 1 and Figure 4B show that the two Arg-Rich Motifs (RRMs) and the Prion-Like Domain (PrLD) of hnRNPA2 contain MoRFs, indicating that intrinsic disorder in these regions is important for the interactability of this protein. In line with this hypothesis, Supplementary Figure S2 shows that the PPI network centered on hnRNPA2 includes 97 partners connected by 1861 interactions, indicating that, on average, each member of this network is engaged in 38 interactions (note that hnRNPA2 itself interacts with 96 partners).

#### 2.5.2. MAVS/IPS1 and MDA5/IFIH1

MAVS. Mitochondrial antiviral signaling (MAVS) protein (also known as the interferon promoter-stimulating factor 1, IPS1) plays a crucial role in host innate immune defense against viruses [125,126,127,128,129,130,131,132,133]. It acts as an important adaptor protein in the toll-like receptor (TLR)-independent recognition of pathogens [134]. The binding of double-stranded RNA (dsRNA) or 5′-triphosphate RNA to cytosolic RNA-sensing receptors, RLRs (retinoid acid-inducible gene I (RIG-I)-like receptors), initiate signaling pathway by promoting interaction between the interferon (IFN)-inducible RNA helicases, such as RIG-I or MDA5 (melanoma differentiation-associated protein 5), and the adaptor protein IPS1, ultimately resulting in an antiviral response mediated by type I IFN production [132,135,136]. Interaction of the adaptor IPS1 protein with the RIG-I and MDA5 helicases is driven by their caspase recruitment domain (CARD) [130,131,132,133]. Although RIG-I and MDA5 share similar underlying mechanisms, these helicases are involved in responses to different viruses (likely in a dsRNA length-dependent manner [137]), with MDA5 being involved in the response to norovirus and picornavirus, and RIG-I being mainly responsible for the response to IAV and paramyxoviruses [138,139]. 

The numerous short functional motifs of this protein are located within or in close proximity to MoRFs (see Table 1 and Figure 4C). Based on STRING analysis, MAVS is involved in interaction with 86 proteins, with the resulting PPI network being characterized by 786 edges (see Supplementary Figure S3). As per FuzDrop analysis (see Table 1), MAVS contains five regions with strong LLPS potential.

MDA5. Melanoma differentiation-associated protein 5 (MDA5, also known as interferon-induced helicase C domain-containing protein 1, IFIH1) is an innate immune receptor acting as a cytoplasmic sensor of viral nucleic acids that activates a cascade of antiviral responses, such as induction of pro-inflammatory cytokines and interferons (INF-α and INF-β) [140,141]. After binding to a viral nucleic acid, MDA5/IFIH1 interacts with the mitochondrial antiviral signaling protein (MAVS/IPS1) and activates IKK-related kinases. Targets recognized by MDA5 include several viruses, including herpes simplex viruses (HSVs) [142], SARS-CoV-2 [143,144], DENV [145], West Nile virus (WNV) [146], ZIKV [146], reovirus [147], and norovirus [148], as well as dsDNA viruses, such as vaccinia virus [149].

It was shown that soon after infection with human RSV (hRSV, a negative-strand RNA virus of the *Pneumoviridae* family, within the *Mononegavirales* order that causes bronchiolitis in children), RIG-I and MDA5 colocalize with viral genomic RNA and N protein, and, at a later step of infection, contribute to form large viral IBs [68]. Today we know that these IBs, as in many negative-sense RNA viruses [31,48,150,151], are liquid and derived from LLPS, but their name dates back to their discovery, which occurred well before the role of LLPS in various biological processes and in the formation of liquid MLOs was recognized. Although cytoplasmic IBs of various sizes found in late hRSV infections contain all the components of the RSV polymerase complex, i.e., viral N, P, M2-1 (nucleocapsid-associated transcription factor), and L (RNA-dependent RNA polymerase) proteins and viral genomic RNA, the formation of such IB-like structures in hRSV-infected cells is driven by N and P proteins alone [68,152]. In hRSV, IBs are heterotypic, complex MLOs exhibiting functional and dynamic subcompartments. Indeed, newly synthetized viral mRNA and the nucleocapsid-associated viral transcription factor M2-1 are condensed within specific IB sub-compartments, referred to as IB-associated granules (BAGs), while viral genomic RNA and N, L, and P proteins are excluded from IBAGs [153]. In the course of infection, N is located in close proximity to MDA5 and MAVS within the hRSV IBs. As a result of the N-driven localization of MDA5 and MAVS into these IBs, the innate immune response to infection is modulated (e.g., expression of INF-β is significantly reduced) [68], indicating that, in addition to acting as sites of viral RNA synthesis, cytoplasmic IBs clearly play a role in controlling the innate immune response.

Curiously, of the four MoRFs found in this protein, two (residues 324–329 and 503–515) are located within the helicase ATP-binding domain, with the two remaining MoRFs being positioned within a long IDR connecting the second CARD domain to the helicase ATP-binding domain (see Table 1). It seems that intrinsic disorder confers high binding promiscuity to MDA5/IFIH1, which, according to STRING (see Supplementary Figure S4) forms a dense PPI network containing 96 proteins connected by 1162 interactions. Furthermore, there are five LLPS-promoting regions in this protein (see Table 1). 

#### 2.5.3. Ras-GAP SH3 Domain-Binding Proteins 1 and 2 (G3BP1 and G3BP2)

The Ras-GTPase-activating protein binding protein (G3BP) family is relatively conserved in eukaryotes [154], with mammals containing three highly homologous proteins, G3BP1, G3BP2a, and G3BP2b, with the latter two being commonly known as G3BP2 [155]. 

G3BP1. G3BP1, which is the most studied member of the G3BP family, is a multifunctional protein with a number of important roles in various biological processes, ranging from cell proliferation to metastasis, apoptosis, differentiation, and RNA metabolism [156]. The roles of G3BP1 in RNA metabolism include regulation of the axonal mRNA translation, ribosomal quality control, and regulation of RNA decay. G3BP1 also possesses an endoribonuclease activity that can be regulated by phosphorylation [157]. Furthermore, this ATP- and magnesium-dependent helicase has crucial roles in innate immunity [158], acting as an antiviral factor that can interact with viral proteins and regulate the assembly of SGs that are crucially involved in the inhibition of viral replication [156]. Importantly, the capability of G3BP1 to promote SG assembly is not limited to cases of viral infection, and this protein is considered one of the key regulators of the SG biogenesis, promoting assembly of these MLOs in response to various environmental stresses [159]. 

According to our analysis, G3BP1 contains nine MoRFs, which are spread through the IDRs and RRM region (Table 1). Binding promiscuity of G3BP1 is illustrated by its dense PPI network that includes 269 proteins connected via 5342 interactions (see Supplementary Figure S5). Additionally, FuzDrop identified four regions with high potential to undergo LLPS (see Table 1). 

G3BP2. G3BP2 is a scaffold protein with an essential role in cytoplasmic SG formation, acting as a platform for antiviral signaling [159]. G3BP2 is ubiquitously expressed and is recruited into SGs [160]. 

Since this second member of the human G3PF family is 98% identical to G3BP1, it is not surprising that these two proteins have a very similar domain organization [161]. Due to alternative splicing, G3BP2 exists in two isoforms: G3BP2a (a 482-residue long “canonical” isoform) and G3BP2b (a 449-residue-long isoform, which lacks residues 243–275). The N protein of SARS-CoV-2 was found to interact with G3BP1 and G3BP2 and localize to SGs [70]. N attenuates SG formation by sequestering host G3BPs away from their physiological interaction partners (e.g., Carpin-1 and Ubiquitin carboxyl-terminal hydrolase 10 (USP10)), and can also rewire the mRNA-binding profile of G3BP1 [70].

Our structural predictions indicate that G3BP2 is slightly more disordered than G3BP1 (PPIDR values = 64.94 and 63.95%, respectively, see Table 1). Furthermore, G3BP2 contains 12 MoRFs (see Figure 4E and Table 1). This protein forms a PPI network containing 105 partners connected by 145 interactions (see Supplementary Figure S6) and has three LLPS-prone regions (see Table 1). Note that due to the alternative splicing, one of the MoRFs (residues 232–281) and one of the LLPS-prone motifs (251–322) are removed or distorted in G3BP2b, suggesting that this isoform has expectedly both a different interactome and a different LLPS behavior.

#### 2.5.4. p53

Transcription factor p53 is a well-known tumor suppressor, exerting its protective function inducing cell cycle-arrest or activating apoptosis in response to several cellular stresses [162], including viral infections [163]. p53 displays a modular architecture, consisting of a DNA-binding and a tetramerization domain, and of IDRs that account for about 40% of the sequence of the entire protein [164]. These IDRs, which encompass a negatively charged N-terminal transactivation domain (TAD), a short proline-rich region, and a C-terminal regulatory domain, are functionally important. All the different functional domains coordinately achieve DNA binding and transactivation [165]. p53 has numerous biological functions, is able to interact with many binding partners, can form both homo-tetramers and isoform-based hetero-tetramers, and can also undergo LLPS and form amyloid-like fibrils. This protein carries multiple PTMs and has several isoforms generated by alternative splicing, alternative promoter usage, or alternative initiation of translation. Therefore, p53 serves as a prototypical illustration of the protein structure–function continuum concept, where the ability of this protein to have a multitude of structurally and functionally different states is determined by the existence of multiple proteoforms generated by various mechanisms [166]. Not by chance, p53 is targeted by the viral protein machinery, as exemplified by the E6 oncoprotein from HPV that binds p53, impairing its transcriptional activity and eventually inducing its degradation [167]. Furthermore, p53 has been shown to form complexes with other tumor virus proteins, namely HSV40 T-antigen and E1B-58K adenovirus protein [168], thus providing a mechanistic explanation for their oncogenic effect. Moreover, p53 was identified at the viral replication sites of SV40, HSV-1, cytomegalovirus, and adenovirus [169,170,171,172]. Recently, the C-terminal DNA-binding domain of HPV16 E2 protein was shown to undergo heterotypic condensation in vitro with p53 [69]. In addition, transfection experiments revealed that E2 co-localizes with p53 in the nucleus with a grainy pattern, with both proteins being found in chromatin-associated, liquid-like foci likely resulting from LLPS [69]. In that study, p53 was proposed to serve as a scaffold for biocondensation, while E2 was proposed to act as both a client and a modulator. The authors proposed that biomolecular condensation of E2 with p53 enables modulating HPV gene function, which is strictly dependent on the host cell replication and transcription machinery [69].

p53 ranks fourth in terms of disorder content among the nineteen cellular proteins herein studied. Indeed, Table 1 and Figure 4D indicate that more than 68% of p53 residues are predicted to be disordered. The high disorder content of p53, readily inferable from Figure 2D, is in line with the ability of this protein to interact with multiple partners, reflected by the presence of eight MoRFs (see Figure 4D and Table 1). This protein forms a PPI network containing 427 partners connected by 3677 interactions (see Supplementary Figure S7) and has four LLPS-prone regions in agreement with its established ability to undergo LLPS (see Table 1). 

#### 2.5.5. p65

Transcription factor p65, also known as nuclear factor NF-κB p65 subunit, is the most investigated member of the NF-κB/Rel family of transcription factors [173], with important activities in cell cycle regulation, cell differentiation, inflammatory and immune cell response, and protection from apoptosis [174]. In mammals, this family includes Rel-A (p65), Rel-B, c-Rel, NF-κB_1_ (p50/p105), and NF-κB_2_ (p52/p100) [174]. Since in the cytoplasm NF-κB is bound to inhibitors of the IκB family, the activation of NF-κB factors and their consequent translocation to the nucleus requires degradation of IκB [175,176]. Activation of NF-κB factors can be triggered by various stimuli ranging from proinflammatory cytokines to various growth factors, bacterial endotoxins, ultraviolet radiation, oxidants, viral proteins, and double-stranded RNA. The p65 subunit is responsible for the strong transcription activating potential of NF-κB [177].

During viral infection, NF-κB- and IRF-dependent signaling is induced by the activation of pattern recognition receptors (PRRs), such as Toll-like receptors (TLRs) and cytoplasmic nucleic acid receptors (RIG-I and MDA5) [178,179,180]. One of numerous strategies utilized by RSV to overcome innate immune response is the inhibition of the NF-κB activation by the RSV SH protein [181,182]. In RSV-infected cells, the NF-κB subunit p65 is rapidly sequestered into perinuclear intracytoplasmic puncta (>3 μm^2^ that increase as infection progresses, eventually approaching a mean area of >22 μm^2^), which were shown to correspond to RSV IBs formed via LLPS [71]. Sequestration of the transcription factor NF-κB subunit p65 to IBs formed during viral infection is a common mechanism for human and bovine RSVs. The process is driven by the viral N and P proteins and leads to an efficient suppression of the NF-κB p65 activation, thereby representing a novel mechanism of immune evasion [71]. 

Figure 4F and Table 1 show that almost 64% of p65 residues are predicted to be disordered, indicating that, like many transcription factors [183], p65 is a highly disordered protein. It has 12 MoRFs and five LLPS-prone regions (see Table 1). Five MoRFs are located within the Rel homology domain (RHD, residues 19–306), while the other MoRFs are found within the three Transcriptional Activation Domains (TADs). The p65-centered PPI network includes 323 partners connected by 5565 interactions (see Supplementary Figure S8). 

#### 2.5.6. Fibrillarin-2 and G3BP-like SG Nucleator from *N. benthamiana*

An interesting mechanism of virus-induced LLPS was described in *N. benthamiana* cells infected with single-stranded, positive-sense RNA Pea enation mosaic virus 2 (PEMV2) [72]. Here, dense and poorly dynamic condensates containing PEMV2 p26, a protein required for the trafficking of viral RNA through the vascular system of infected plants [184]), were observed in the nucleolus of infected cells. These condensates, in addition to viral p26, contain nucleolar protein fibrillarin (Fib2) and PEMV2 genomic RNAs [72]. The recruitment of Fib2 into droplets and the ability to systemically traffic a virus vector requires p26′s ability to phase separate, as both of these activities are suppressed in phase separation-deficient p26 mutants [72]. Fib2, which is involved in the systemic trafficking of viral ribonucleoprotein complexes [185,186], itself forms the dense fibrillar component of the nucleolus [187], indicating that it acts as a scaffold responsible for recruiting client proteins into the nucleolus [72]. Although there is no structural information on *N. benthamiana* Fib2, its counterpart from *Arabidopsis thaliana* was shown to contain an N-terminal intrinsically disordered glycine- and arginine-rich (GAR) domain (Fib2_GAR_) that is sufficient, per se, to drive Fib2 phase separation [72]. In line with these data, we predicted three short IDRs and a GAR domain (residues 1–83) in *N. benthamiana* Fib2 (see Table 1). Our analysis unveiled the presence of three MoRFs located within the Fib2_GAR_, which, according to FuzDrop, also shows high LLPS potential (see Table 1 and Supplementary Figure S9).

Furthermore, p26 was shown to partition into SGs [72], where it colocalizes with a nuclear transport factor 2 (NTF2) protein endowed with an RNA-binding domain that functions as a G3BP-like SG nucleator in plants [188], whose clustering after stress results in SG assembly. Table 1 and Supplementary Figure S10 show that this G3BP-like SG nucleator is predicted to have nine MoRFs and forms a dense PPI network with 410 partners connected by 27,495 interactions. Furthermore, there are four droplet-promoting regions in this protein (see Table 1).

#### 2.5.7. p38MAPKα

The stress-activated p38 mitogen-activated protein kinase (p38MAPKα, also known as Mitogen-activated protein kinase 14, MAPK14) is a central mediator involved in the regulation of cellular inflammatory, stress responses, and cellular protein synthesis [189]. p38MAPKα (or p38α) is one of four members of the p38MAPK family (with the remaining three being p38β, p38γ, and p38δ); it is activated by multiple extracellular stimuli and is known to regulate more than sixty substrates [190]. 

Under stress conditions, p38MAPKα and MAPK2, another serine/threonine-protein kinase that is activated by stress via p38MAPKα phosphorylation, were shown to play important roles in post-transcriptional mRNA metabolism [77]. During RSV infection, p38MAPKα is mostly sequestered within viral IBs, where it colocalizes with the viral M2-1 and P proteins [77]. Sequestration of p38MAPKα within IBs results in a dramatic decrease in the cellular levels of MAPK2, with the remaining protein being mostly unphosphorylated, inducing efficient inhibition of the downstream pathways [77]. 

From a structural point of view, human p38MAPKα is a globular protein containing an N-terminal protein kinase domain (residues 24–308), which includes a TXY motif (residues 180–182) containing threonine and tyrosine residues whose phosphorylation activates the MAP kinases. Although this protein is mostly ordered, it is predicted to have six short IDRs, with one of them acting as a droplet-promoting region (DPR, see Table 1). Since p38MAPKα is a promiscuous kinase modulating the activities of multiple partners, it is not surprising to find that this protein forms a very dense and highly connected PPI network with 263 proteins and 3555 interactions (see Supplementary Figure S11).

#### 2.5.8. FAK1

Focal adhesion kinase 1 (FAK1, also known as Protein-tyrosine kinase 2, PTK2) is a cytoplasmic non-receptor protein-tyrosine kinase preferentially localized at cellular focal contacts. FAK1 has a number of crucial roles in the regulation of angiogenesis, cell adhesion, apoptosis, migration, proliferation, and spreading, as well as in the control of cell cycle progression, formation, and disassembly of focal adhesions and cell protrusions, and the reorganization of the actin cytoskeleton [191]. Among the important activities of FAK1 are regulation of integrin and growth factor signaling pathways [192]. This kinase is most known for its role in many invasive and metastatic cancers, such as breast cancer, lung cancer, neck cancer, ovarian cancer, and prostate cancer, where high FAK levels are associated with poor prognosis.

FAK1 was shown to be involved in RABV infection via interaction with the viral P protein, being one of the human interacting proteins isolated in a two-hybrid screen using RABV P as a bait [73]. Importantly, FAK1 was shown to accumulate in Negri bodies during RABV infection, and this recruitment is mediated by the interaction of P with FAK1 [73]. 

In terms of its architecture, FAK1 contains a central kinase domain flanked by long N- and C-terminal domains [73]. While the N-terminal region is responsible for the regulation of FAK1 activity, the C-terminal region includes a proline-rich IDR and the focal-adhesion-targeting (FAT) domain, responsible for localizing FAK1 to focal adhesions [193]. 

Our analysis showed that FAK1 contains fourteen MoRFs, with two of them being located within the FAT domain (see Table 1 and Supplementary Figure S12). It is likely that the presence of such a large number of MoRFs dictates the binding promiscuity of FAK1, which is predicted to form a PPI network containing 262 partners involved in 5082 interactions (see Supplementary Figure S12). With four DPRs and a p_LLPS_ of 0.6417, FAK1 can be considered a LLPS driver. Surprisingly, however, the PSPredictor failed to recognize FAK1 as a phase-separating protein. 

#### 2.5.9. TIAR1

Nucleolysin TIAR1 (T-cell intracellular antigen 1 (TIA-1)-related protein 1) is an RNA-binding protein related to alternative pre-RNA splicing along with the formation, organization, and function of SGs. In SG biogenesis, TIAR1 acts downstream of the stress-induced phosphorylation of EIF2S1/EIF2A, promoting the recruitment of untranslated mRNAs to cytoplasmic SGs [194]. 

The biogenesis of canonical and non-canonical SGs induced by a variety of pharmacological stresses is efficiently suppressed in cells infected by EBOV, a single-stranded negative-sense RNA virus belonging to the *Filoviridae* family within the *Mononegavirales* order, responsible for severe human hemorrhagic fever. Suppression of SG formation is mediated by the EBOV protein 35 (VP35) [74] through the sequestration of SG-specific host proteins, including TIAR1. Furthermore, VP35 was found to colocalize with TIAR1 in cytoplasmic aggregates, which are likely to be viral inclusion bodies [74,195]. Therefore, akin to many other viruses [196], EBOV is capable of blocking SG biogenesis and subverting the SG components for its own benefits. 

Structurally, TIAR1 is characterized by the presence of three RNA recognition motifs, RRM1-3, that have different functions. The binding of TIAR1 to target RNA depends on RRM2, which is both necessary and sufficient for this interaction, whereas RRM1 and RRM3 contribute to the affinity of the interaction with RNA [197]. According to our computational analyses, TIAR1 contains three MoRFs, one of which is embedded within RRM3, and one DPR (see Table 1 and Supplementary Figure S13). The protein has at least nine phosphorylation sites. Furthermore, its Lys122 can be either ubiquitinated or acetylated (see Supplementary Figure S13). It seems that disorder-based interaction sites and PTMs are related to the high interactivity of this protein that forms a dense and highly connected PPI network including 22 proteins and 6457 interactions (see Supplementary Figure S13). 

#### 2.5.10. HSP70-1

Heat shock 70 kDa protein 1A (HSP70-1) is the major inducible heat shock protein chaperone with well-established roles in protein homeostasis and regulation of various cellular processes, ranging from protein translation to protein folding, intracellular trafficking, and degradation. Beyond its activity as a chaperone preventing protein misfolding and aggregation [198], HSP70-1 is also involved in the regulation of apoptosis, cell cycle regulation, innate immunity, and signal transduction [199,200]. Therefore, it is not surprising that host chaperones, including HSP70-1, are in high demand during viral infection, as viruses utilize them for controlling the correct folding of the massively produced viral proteins and also for interfering with the regulation of fundamental cellular processes controlled by chaperones. In line with these considerations, it was established that HSP70-1 is commonly recruited by viruses, being involved in different stages of the life cycle of different viruses [201,202,203,204], and that many DNA, positive-strand RNA, and negative-strand RNA viruses are capable of specifically inducing HSP70-1 expression [201,205]. Although this enhanced HSP70-1 expression mostly has proviral effects, e.g., leading to elevated expression of viral genes, in some cases, it can confer antiviral protection [206,207,208,209,210], and in other cases can show both positive and negative regulatory effects [211]. Proviral effects have been documented in the case of cells infected by RABV, in which HSP70-1 expression is enhanced, and in which this chaperone accumulates within NBs [28]. The sequestration of HSP70-1 within NBs is driven by the interaction with RABV N protein [212]. Furthermore, HSP70-1 was also found in purified nucleocapsids and in purified RABV particles [212]. The proviral effect of HSP70-1 during RABV infection is supported by the fact that the down-regulation of HSP70-1 expression is accompanied by the inhibition of different steps of the viral cycle [212]. Likewise, HSP70 was found to be associated with the nucleocapsid of various paramyxoviruses, including canine distemper virus (CDV) [213] and MeV, where it was shown to stimulate viral transcription and replication [204] via interaction with the C-terminal intrinsically disordered domain of MeV N protein [203].

Human HSP70-1 is a multidomain protein containing a nucleotide-binding domain and a substrate-binding domain joined by a flexible linker. Table 1 and Supplementary Figure S14 show that human HSP70-1 contains several short and two long IDRs. Although four MoRFs and three DPRs are all located within the disordered C-tail (residues 490–641), this protein is heavily decorated by a multitude of various PTMs, such as phosphorylation, methylation, ubiquitination, and acetylation. Since HSP70-1 is a molecular chaperone, it is expected to be a promiscuous binder. In line with this, HSP70-1 lies at the center of a network of 4876 interactions among 214 binding partners (see Supplementary Figure S14). 

#### 2.5.11. RAB11

RAB11 is a small, globular protein belonging to a large family of GTPases called Ras-related in brain (RAB), with 44 subfamilies encoded in the human genome [214]. Normally, RAB11 is the main regulator of endocytic vesicle trafficking, used by the cell both to acquire endocytosed material and to export vesicles transited through the Golgi apparatus to the cell surface. The activity of each RAB depends on its association with GTP. 

RAB11 was recently shown to play a key role in the viral cycle of IAV, a member of the *Orthomyxoviridae* family [215]. A peculiar aspect of IAV virions is the host-derived plasma membrane envelope surrounding its segmented genome consisting of eight negative-sense single-stranded RNA segments. Each segment forms a rod-shaped ribonucleoprotein particle (vRNP). In addition to RNA, all vRNPs contain several copies of viral nucleoprotein (NP) and an RNA-dependent RNA-polymerase molecule.

The life cycle of IAV includes translocation of the vRNPs to the nucleus, where transcription and replication of the viral RNA genome, nuclear export and assembly of the vRNPs near the host cell membrane, and release of new virions from the host cell take place. The two most obscure steps are genome transport to the budding sites and genome assembly, the latter made critical by the segmented nature of the IAV genome [215]. The exact mechanism by which each virion contains an entire set of vRNPs in the correct assortment is not clear [215]. It has been recently observed that, once out of the nucleus, vRNPs accumulate into fluid, membrane-less droplets, in which RAB11 also colocalizes. These cellular inclusions, which can be considered viral factories, enlarge as the infection progresses [58]. Furthermore, as viral factories approach the plasma membrane from the nucleus, they tend to become larger and contain more vRNPs, until they reach the final arrangement of eight segments, in a spatiotemporal process of maturation. Viral inclusions appear to be closely associated with endoplamsic reticulum (ER) tubules, which likely guide their movements [58]. 

Recent findings cast doubt on whether RAB11-GTPase has a necessary function in driving exocytosis of forming IAV virions to budding sites. On the contrary, some data are in favor of a complete disruption of RAB11 natural function, which might be dampened or annulled through its recruitment to viral inclusions. Consider in this regard also the fact that RAB11 was found to be redirected towards the ER during IAV infection [216]. It remains to be discovered whether RAB11 plays an active role in the formation of inclusions, although its nature as a single-domain, globular protein, along with its low propensity to LLPS, suggest a role as *client*.

Table 1 and Supplementary Figure S15 show that human RAB11 possesses two MoRFs, one DPR, and multiple PTM sites. This protein interacts with 96 partners and the resulting PPI network has 485 edges (see Supplementary Figure S15).

#### 2.5.12. PP1

The human protein serine/threonine phosphatase type 1 (PP1) regulates important cellular functions ranging from cell division to glycogen metabolism, muscle contractility, and protein synthesis [217]. It is also involved in the regulation of ion conductance and long-term synaptic plasticity and embryonic development. PP1 has an overall globular structure, except for a C-terminal disordered region of ~30 residues and short loops connecting secondary structure elements (see Table 1). PP1 was found to have a key role in the life cycle of human RSV [75]. While this phosphatase has no propensity to form coacervates on its own, it behaves as a client of viral P protein coacervates, in cytoplasmic IBs formed in infected cells. Cellular IBs contain viral N and P proteins, viral polymerase, and the viral transcription factor M2-1, and are sites where viral RNA synthesis occurs [153]. Within the cellular inclusions, the viral transcription factor M2-1 is juxtaposed to PP1 and thus de-phosphorylated, a modification that causes its activation as an anti-terminator of viral transcription.

How does PP1 serve viral replication? PP1 is a pleiotropic enzyme. The ability of PP1 to catalyze about one third of all dephosphorylation reactions occurring inside a mammalian cell depends on its ability to interact with over 200 different cell adaptors and regulatory proteins, often IDPs/IDRs, while retaining high specificity [218,219]. In line with these observations, the STRING-generated PPI network of this protein includes 190 partners (see Supplementary Figure S16). The viral P protein acts both as a condensation scaffold and an adaptor that allows further expansion of the PP1 substrate specificity to include the viral M2-1 protein. In RSV, P protein contains a well-conserved ‘RVxF’ motif required for the interaction with PP1. On the other hand, the degenerate RKPLVSF motif, with the consensus KxxVxF, is also shared among the P proteins of all *Pneumoviridae* members. This suggests that the P proteins of pneumoviruses may also interact with PP1 to regulate the phosphorylation of their M2-1 proteins [75]. The sharing of this molecular mechanism suggests a certain probability of interspecific spillover, and this knowledge might represent an important contribution to the design of new broad-spectrum antiviral drugs.

#### 2.5.13. CAD

Carbamoyl-phosphate synthase (CAD) is a giant 1.5 mDa protein composed of six identical protomers. Each protomer is divided into domains that catalyze reactions from the de novo biosynthetic pathway of pyrimidine nucleotides starting from bicarbonate. The de novo pathway produces uridine 5-monophosphate (UMP), from which all other pyrimidine nucleotides are obtained through several reactions catalyzed by individual catalytic domains linked by IDRs and acting in a coordinated manner [220]. This type of architecture is found in the CAD of all metazoans and is also found in human glutamine-dependent CAD.

It is known that CAD localizes in the cytoplasm of resting cells, and growth and proliferation stimuli are transmitted via the MAP kinase-mediated phosphorylation of its Thr-456. This PTM is followed by the translocation of a small fraction of CAD into the nucleus, suggesting a specific but yet to be elucidated function. 

The role of CAD was explored in relation to EBOV infection. Like other members of the *Filoviridae* family, upon infection, EBOV forms cytoplasmic IBs, where transcription and replication take place. The minimal element for the formation of IBs is the viral nucleoprotein (N) that also acts as a scaffold for CAD recruitment [76]. The latter relies on interaction between N and the first N-terminal globular domain of CAD with glutaminase activity. It is likely that EBOV exploits the catalytic activity of this cell enzyme to fulfill its replicative needs and provide enough pyrimidines for replication and transcription of the EBOV genome. This hypothesis is consistent with the observation that pyrimidine synthesis inhibitors are effective against EBOV in vitro, underlining the importance of the pyrimidine pathway for this virus [221,222]. 

Human glutamine-dependent CAD contains multiple functional domains bearing various enzymatic activities. Therefore, it is not surprising that it is predicted to be a mostly ordered protein. Nevertheless, data shown in Table 1 and Supplementary Figure S17 indicate that human CAD contains multiple IDRs, most of which are relatively short, with the exception of two long IDRs (residues 360–402 and 1807–1923). Despite its overall relatively high level of order, CAD is predicted to have nine MoRFs and one droplet-promoting region (DPR, see Table 1 and Supplementary Figure S17). Therefore, CAD belongs to the category of LLPS client proteins (it is characterized by a low p_LLPS_ score of 0.2011, but contains a DPR). According to the STRING analysis, human CAD is positioned at the center of a broad PPI network containing 119 partners connected by 1358 interactions (see Supplementary Figure S17). In line with the ability of CAD to undergo phosphorylation, our analysis revealed that CAD contains multiple sites of different PTMs, such as phosphorylation, ubiquitination, and acetylation (see Supplementary Figure S17), suggesting that at least some biological functions are PTM-controlled.

#### 2.5.14. OGT

The O-linked N-acetylglucosamine transferase 110 kDa subunit (OGT, also known as UDP-N-acetylglucosamine-peptide N-acetylglucosaminyltransferase 110 kDa subunit) catalyzes the transfer of a single N-acetylglucosamine (OGN) from UDP-GlcNAc to a serine or threonine residue in proteins destined for extracellular export. OGT is an ER-resident protein that glycosylates the Thr residue located between the fifth and sixth conserved cysteine of EGF-like folded domains. The post-translational addition of OGN to target proteins regulates various cellular processes, including signal transduction, transcription, translation, and stress response.

A variety of stresses that alter proteostasis can activate an integrated stress response (ISR) in mammalian cells. ISR is an adaptive mechanism that involves the phosphorylation, through various kinases, of eIF2a translation initiation factor. This results in the blockade of protein translation, promoting intracellular accumulation of ribonucleoproteins and untranslated mRNA that eventually condense into SGs. It is noteworthy that the formation of SGs requires the addition of OGN to various ribosomal proteins that form ribonucleoprotein complexes [223,224,225,226]. SGs are rapidly dissolved in the cytoplasm after stress release [227,228,229], but can promote apoptosis when they persist [230].

Although the role of SGs in viral infections is still unclear, data are accumulating to support the hypothesis that their formation is altered or counteracted during viral infections, which could represent a persistent stress for the cell. OGT has been found in IBs formed in the cytoplasm of RSV-infected cells. We hypothesize that this does not depend on its intrinsic ability to form condensates and rather likely depends on OGT recruitment by viral scaffold proteins through structural motifs that are not known yet [77].

The structure of OGT has not yet been determined experimentally, and AlphaFold2 predicts a predominantly globular structure for it (see Figure 2S), making it the most ordered protein among the proteins in our data set. FuzDrop identified one short droplet-promoting region (see Table 1 and Supplementary Figure S18). Although it is predicted to possess 16 short IDRs (see Table 1), it does not contain any MoRFs. However, according to the STRING analysis (see Supplementary Figure S18), OGT is expected to be involved in interaction with 188 partners forming a dense PPI network with 1609 interactions. 

## 3. Discussion

The work presented here is based on a manually curated list of 19 cellular proteins derived from an unbiased search with respect to their disorder content and LLPS propensity. The data set includes either host proteins recruited to viral factories or proteins responsible for the recruitment of viral proteins within cellular MLOs, with both types of liquid-like compartments having been referred to as “viral infection-related MLOs” (vir-MLOs). In spite of its small size, the data set enabled us to consider a broad cross-section of possible behaviors in different virus families. Computational analysis of the data set revealed that cellular proteins found in vir-MLOs have, on average, a high disorder content and are enriched in intrinsically disordered binding sites (i.e., MoRFs), as well as in sites of various PTMs. In agreement, these proteins are highly promiscuous and typically characterized by very well developed and dense PPI networks, as unveiled by STRING-based analysis. Their functions are mainly, though not exclusively, related to SG biogenesis, innate immune response, and viral nucleic acid sensing. 

A correlation was found between the content of intrinsic disorder in the analyzed proteins and their propensity to undergo LLPS. Although there is a well-known correlation between the intrinsic disorder status of a protein and its LLPS propensity [14], the actual disorder–LLPS relationship is not very obvious. In fact, not all IDPs are unequivocally engaged in LLPS, and not all proteins undergoing LLPS are highly disordered. This is exemplified by our results that show that although most cellular proteins recruited to vir-MLOs are enriched in disorder, a few of them are not, which is per se a novel and non-predictable finding. Furthermore, the relationship between LLPS and disorder has never been systematically analyzed before for cellular proteins recruited to vir-MLOs. Therefore, the observations reported in this study contribute to the enlargement of our knowledge in the field and pave the way towards new investigations. 

With few exceptions, when the disorder content is >50%, the vir-MLO proteins of our data set were found to fall in the category of *bona fide drivers* according to experimental evidence collated in MLOsMetaDB [98]. A complementary observation is that more ordered cellular proteins tend instead to be *clients* or even *regulators* of viral factories. This role is enabled by their strong promiscuity, evidenced by their ability to interact with multiple adaptor molecules that modulate their physiological function (see, for example, RAB11 and PP1). It is, therefore, tempting to hypothesize that the higher the disorder content, the higher the chances that a phase-separating protein behaves as a *driver*. It should be emphasized that the role of *(co)-driver* or *client/regulator* we herein proposed based on disorder content has to be taken with caution, considering that vir-MLOs form in the context of viral infection, which can significantly affect protein concentration, thus adding extra complexity to the system. In particular, the ability to drive phase separation is not just an intrinsic property of a protein, but it also depends on its concentration and environment (temperature, pH, partners, etc.) [13]. In addition, one should keep in mind that some proteins may act as *drivers* in some MLOs but behave as *clients* in others, and that the current classification used in MLOsMetaDB is susceptible to evolve, as Orti and co-workers emphasized [98]. It is, nevertheless, conceivable that viral proteins participating in vir-MLOs may play roles as *clients* and *regulators*, thus taking advantage of the scaffolding abilities provided by cellular proteins. This might be regarded as good news, because it would provide a conceptual basis for designing new antiviral drugs aimed at interfering and abrogating a specific viral function, without altering the intrinsic LLPS behavior of host cell proteins. 

Finally, we would like to emphasize that the emerging trend, whereby cellular proteins with a more globular structure tend to be *clients* of viral factories, cannot be generalized at this stage, considering the size of the data set, the latter being intrinsically limited by the availability of reported examples in the literature. Future enlargements of the data set will rely on new examples of host cell proteins colocalizing in vir-MLOs that will be reported in the literature. For instance, candidates to be included in this data set are proteins found in SGs or involved in the NF-κB pathway [65,66], as well as specific kinases and phosphatases expected to be client proteins of IBs formed by MeV N and P proteins [48,67], whose participation in vir-MLOs has been only hypothesized and not yet experimentally demonstrated [48,67]. We anticipate that in the future, the list of cellular proteins recruited to vir-MLOs will grow fast and we hope that the present work will stimulate future studies aiming to experimentally assess this trend. Considering that many proteins that undergo LLPS *in cellula* also phase separate in vitro, with the resulting condensates recapitulating those observed in vivo, it should be possible for any given cellular protein recruited to vir-MLOs to experimentally assess its proposed role as *client* or *(co)-driver* by combining information on its predicted/experimental disorder content with in vitro LLPS assays with purified proteins. 

## 4. Materials and Methods

### 4.1. Data Set Generation

The data set of *bona fide* cellular proteins that are either recruited to virus-specific condensates or are found in cellular MLOs into which viral proteins colocalize was generated manually through literature data mining. Specifically, research articles pertaining to the viral condensates resulting from LLPS were retrieved by using the following search criteria in PUBMED: (“virus” OR “viral”) AND (“condensates” OR “liquid” OR “LLPS” OR “phase separation”). Then, a list of cellular proteins shown to be recruited to liquid viral condensates or of cell proteins found in cell MLOs recruiting viral proteins was generated. This resulted in a relatively short list of targets whose sequences, in FASTA format, were retrieved from UniProt [231].

### 4.2. Predictions of LLPS Propensity

Phase separation predictions were conducted using the FuzDrop web server (https://fuzdrop.bio.unipd.it/predictor) (last accessed on 11 January 2023), which performs a sequence-based identification of both droplet-promoting and aggregation promoting regions on the query sequence (UniProt code [231] or FASTA file as an input) [232,233,234]. The FuzDrop output provides a probability score of spontaneous LLPS (p_LLPS_ ≥ 0.60 for droplet *drivers*) and an interactive graph with droplet-promoting probabilities (pDP) per single residue. Proteins with p_LLPS_ < 0.60 yet with at least one droplet-promoting region are classified as droplet–client proteins, as they partition into droplets interacting with a partner. Below this graph, a scheme represents the droplet promoting regions (DPRs) with blue boxes, defined as a window of ≥10 residues with pDP ≥ 0.60. Analogously, aggregation hotspots, defined as part of droplet-promoting regions with large binding mode diversity (S_bind_ ≥ 2.20), are shown as orange boxes. These regions have a minimum length of five residues with a gap of a maximum of two residues. The droplet-promoting and aggregation promoting regions are then displayed by Mol* [235] on the structure predicted with AlphaFold2 [236] and stored in the AlphaFold2 database [237]. 

The propensity of a query protein for phase separation was also analyzed by the PSPredictor (http://www.pkumdl.cn:8000/PSPredictor/) (last accessed on 9 January 2023), which is a sequence-based tool for the prediction of potential phase separation proteins (PSPs) [238]. PSPredictor takes up to 100 proteins in FASTA format and generates PSP score(s) for query protein(s) in a tabulated form showing PSP scores ranging from 0 to 1 and information on the PSP status of a query protein in a “Yes/No” form. A query protein can be considered a potential phase separating protein if its PSP score is ≥0.50.

### 4.3. Disorder Predictions

Disorder predictions were run using an in-house modified version of the MeDor disorder metaserver [87]. This version includes all the predictors implemented in MobiDB-lite 3.0 [239]. MobiDB-lite uses eight different predictors, namely GlobPlot [240], three versions of eSpritz (DisProt, eSpritz-D; NMR, eSpritz-N; and X-ray, eSpritz-X), two versions of IUPred (long and short), and two versions of DisEMBL (465 and hotloops) to derive a consensus that is refined to remove short disordered regions and keep only those that consist of at least 20 consecutive residues predicted to be disordered. In addition, MeDor aggregates the results of four additional predictors, i.e., DorA, MoreRONN [241], FoldIndex [242], and FoldUnfold [243] (see below). 

The MeDor output displays the MobiDB-lite consensus (referred to as Consensus MobiDB) and two other types of global consensus: Consensus 1, which corresponds to regions predicted to be disordered by more than half of the implemented predictors, and Consensus 2, which corresponds to regions consistently predicted to be disordered by all the implemented predictors. The eight individual predictors included in MobiDB-lite 3.0, as well as the four additional disorder predictors implemented in MeDor, are described below.

GlobPlot is a web server for the identification of regions of globularity and disorder within protein sequences that is based on a running sum of the propensity for amino acids to be in an ordered or disordered state [240]. ESpritz is an ensemble of protein disorder predictors, which are based on bidirectional recursive neural networks and trained on three different “flavors” of disorder, X-ray disorder (regions with missing electron density in crystallographic structures), DisProt disorder (regions/proteins annotated as IDR/IDPs in the DisProt database [82,244]), and NMR mobility (data sets calculated using the Mobi server based on a simple algorithm to find regions with different conformations among all models in an NMR ensemble) [245,246]. IUPred2A predicts whether a protein (or protein region) is structured or disordered based on the estimation of the total pairwise inter-residue interaction energy. This approach is based on the assumption that the lack of structure in IDPs/IDRs is defined by their inability to form sufficient stabilizing inter-residue interactions. Since amino acid sequence features of long and short IDRs are known to be different, the long option of IUPred2A predicts global structural disorder that encompasses at least 30 consecutive residues of the protein, whereas the short option of IUPred2A uses a parameter set best suited for predicting short IDRs [92,93]. Finally, DisEMBL is another computational tool for the prediction of IDRs within a protein sequence, which is based on artificial neural networks trained for predicting several definitions of disorder from protein crystal structures, such as hotloops (i.e., loops with a high degree of mobility as determined from Cα temperature factors (B factors)) and missing coordinates in X-Ray structure as defined by remark465 entries in PDB [247].

DorA is an unpublished predictor developed in the AFMB lab that uses the size and abundance of hydrophobic clusters in the Hydrophobic Cluster Analysis (HCA) plot [248,249] to predict disorder. MoreRONN [250] is based on a Bio-Basis Function Neural Network. It relies on a set of curated disordered sequences and it does not need any other information (e.g., amino-acid characteristics or secondary structure predictions), which makes it a solid and fast predictor. FoldIndex distinguishes globular and intrinsically disordered proteins based on the ratio of net charge versus hydropathy. It computes the charge/hydropathy ratio using a sliding window along the protein and provides a per-residue (dis)order score [242]. FoldUnfold calculates the expected average number of contacts per residue from the amino acid sequence alone, where the average number of contacts per residue was computed from a data set of globular proteins [243]. A region is considered to be natively unfolded if the expected number of close residues is less than 20.4 for its amino acids and the region is greater or equal in size to the averaging window [243]. 

Beyond aggregating disorder predictions, the output of MeDor also displays low-complexity regions (as predicted by SEG [251]), transmembrane regions (as predicted by Phobius, http://phobius.sbc.su.se/index.html, last accessed on 9 December 2022), secondary structure elements (as predicted based on the StrBioLib library of the Pred2ary program [252]), and a HCA plot [248,249] (see Appendix A).

Intrinsic disorder predisposition analysis of all the proteins was also conducted using the high-efficiency web-based disorder predictor Rapid Intrinsic Disorder Analysis Online (RIDAO) designed to facilitate the application of protein intrinsic disorder analysis in genome-scale structural bioinformatics and comparative genomics/proteomics [253]. RIDAO aggregates the outputs of six well-known disorder predictors: PONDR^®^ VLXT [88], PONDR^®^ VL3 [89], PONDR^®^ VLS2 [90], PONDR^®^ FIT [91], IUPred2A (Short), and IUPred2A (Long) [92,93], and also provides mean disorder predictions for query proteins by averaging the outputs of these six predictors. Individual predictors included in RIDAO are briefly described below. 

PONDR^®^ VLXT utilizes different neural networks to separately analyze N- and C-terminal regions and the internal region of the sequence of a query protein. Each of these neural networks is trained using a specific data set encompassing only the amino acid residues of that specific region, and their input features include selected compositions and profiles from the primary sequences. The final prediction combines the outputs of the individual predictors in their respective regions, with the transition from one predictor to another being accomplished by computing the average scores of the two predictors for a short region of overlap at the boundary between the two regions [88]. Although PONDR^®^ VLXT may underestimate the occurrence of long disordered regions in proteins and is not the most accurate disorder predictor, this tool was shown to be sensitive to the local sequence peculiarities, and, therefore, to have significant advantages in finding potential disorder-based binding sites [95,254]. PONDR^®^ VL3 was specifically designed to predict long IDRs. This predictor employs ten neural networks that use different sequence attributes as inputs and selects the final prediction by simple majority voting [89]. PONDR^®^ VSL2 combines neural network predictors for both short (<30 residues) and long (≥30 residues) IDRs, with each individual predictor being trained using the data sets of sequences with that specific length. The final prediction is a weighted average determined by a second layer predictor. PONDR^®^ VSL2 is the most accurate stand-alone disorder predictor from the PONDR family [90]. PONDR^®^ FIT is a consensus artificial neural network (ANN) prediction method (a meta-predictor) that uses the outputs of several individual disorder predictors, such as PONDR^®^ VLXT [88], PONDR^®^ VL3 [89], PONDR^®^ VLS2 [90], PONDR^®^ FIT [91], IUPred [93], FoldIndex [242], and TopIDP [255]. PONDR^®^ FIT is characterized by an improved prediction accuracy over a range of 3 to 20% with an average of 11% compared to the single predictors, depending on the datasets being used [91]. 

In this study, in addition to generating composite disorder profiles for each query protein, the predicted percentage of intrinsically disordered residues (PPIDR, i.e., percent of residues with disorder scores exceeding 0.5) was calculated based on the outputs of PONDR^®^ VLS2, which is characterized by high predictive power, as evidenced by the results of the recently conducted ‘Critical assessment of protein intrinsic disorder prediction’ (CAID) experiment, where this tool was found to rank as predictor #3 among the 43 evaluated methods [79].

Disorder-based binding sites, also known as molecular recognition features, MoRFs, i.e., short disordered regions that fold upon interaction with binding partners [95,96], were identified from the D^2^P^2^ output, in which MoRFs are predicted using ANCHOR [256]. Since D^2^P^2^ does not have data for Fib2 (UniProt ID: B7VCB9), MoRFs for this protein were predicted by IUPred2A using the option ANCHOR2 [97]. 

The global disorder status of query proteins was assessed by CH–CDF analysis [84,257,258,259] that combines the outputs of two binary predictors, the charge-hydropathy (CH) plot [260,261] and the cumulative distribution function (CDF) plot [257,261,262], to create a CH–CDF phase space, where proteins are classified as ordered (proteins predicted to be ordered by both binary predictors), putative native “molten globules” or hybrid proteins (proteins predicted to be ordered/compact by CH, but disordered by CDF), putative native coils and native pre-molten globules (proteins predicted to be disordered by both methods), and proteins predicted to be disordered by CH-plot, but ordered by CDF.

### 4.4. Generation of Functional Disorder Profiles

The potential disorder-related functionality of query proteins was analyzed using the D^2^P^2^ platform, which is a database of predicted disorder for proteins from completely sequenced genomes (http://d2p2.pro/) (last accessed on 9 December 2022) [94]. D^2^P^2^ uses the outputs of ESpritz [246], PONDR^®^ VLXT [88], PONDR^®^ VSL2B [78,89], PrDOS [263], PV2 [94], and IUPred [92,93] to show disorder predispositions of query proteins and to evaluate their agreement. The platform also shows the positions of functional SCOP domains [264,265] predicted by the SUPERFAMILY predictor [266]. The functional disorder profile also includes information on the location of predicted disorder-based binding sites (molecular recognition features, MoRFs) identified by the ANCHOR algorithm [267] and various PTMs assigned using the outputs of the PhosphoSitePlus [268]. 

### 4.5. Analysis of Protein Interactivity

The Search Tool for the Retrieval of Interacting Genes, STRING, http://string-db.org/ (last accessed on 9 December 2022) [269], was used to acquire information on the interactome of the target eukaryotic proteins. The STRING output represents a network of predicted and experimentally validated protein–protein interactions using seven types of evidence, such as co-expression evidence, co-occurrence evidence, neighborhood evidence, database evidence, experimental evidence, fusion evidence, and text mining evidence [269]. Protein–protein interaction networks of Fibrillarin 2 (UniProt ID: B7VCB9) could not be obtained as STRING does not have any information on this protein. 

Accession numbers. UniProt IDs: P35637, Q7Z434, P22626, P04637, Q9UN86, Q04206, Q13283, Q13148, Q84JH2, Q9BYX4, B7VCB9, Q05397, Q01085, P0DMV8, P62491, P62136, P27708, Q16539, and O15294.

## 5. Conclusions

In conclusion, our work constitutes the first building block towards the generation of a database meant to gather experimental or predictive data on LLPS propensity and disordered nature of cellular proteins recruited to vir-MLOs. Work to generate such a publicly available database is in progress. We expect that such a database will be rapidly expanding and hope that the present study will foster future efforts aiming to decipher general rules and molecular patterns underlying the function of these cellular proteins, thus paving the way towards the rational design of innovative antiviral strategies.

## Figures and Tables

**Table 2:** GO annotation results for the “internal” interaction network. The table shows the five most enriched terms for biological processes, molecular functions, and cellular components among the network of reciprocal interactions predicted by STRING for the 17 human proteins considered in this study. In order to include all proteins in the network, the minimum required interaction score: low confidence (0.150) was used.

GO Terms	GO ID	*p* Value
**Biological processes (five most enriched)**		
Symbiotic process	0044403	1.68 × 10^−7^
Viral process	0016032	1.17 × 10^−7^
Interspecies interaction between organisms	0044419	1.20 × 10^−5^
Negative regulation of catabolic process	0009895	1.69 × 10^−5^
Cellular response to stimulus	0051716	1.85 × 10^−5^
**Molecular functions (five most enriched)**		
mRNA binding	0003729	9.29 × 10^−6^
mRNA 3-UTR binding	0003730	1.86 × 10^−5^
RNA binding	0003723	0.0031
Protein phosphatase binding	0019903	0.0064
Organic cyclic compound binding	0097159	0.0073
**Cellular components (five most enriched)**		
Cytoplasmic stress granule	0010494	0.00086
Ribonucleoprotein complex	1990904	0.00086
Cell junction	0030054	0.0107
Protein-containing complex	0032991	0.0107
Nuclear matrix	0016363	0.0193

**Table 3:** GO annotation results for the “external” interaction network. The table shows the five most enriched terms among biological processes, molecular functions, and cellular components for the network of interactions that the 17 human proteins considered in this study establish with the human proteome, as predicted by STRING using minimum required interaction score: highest confidence (0.900).

GO Terms	GO ID	*p* Value
**Biological processes (five most enriched)**		
Positive regulation of nitrogen compound metabolic process	0051173	1.34 × 10^−122^
Positive regulation of cellular metabolic process	0031325	3.13 × 10^−119^
Positive regulation of cellular process	0048522	1.80 × 10^−116^
Positive regulation of macromolecule metabolic process	0010604	1.19 × 10^−115^
Regulation of cellular metabolic process	0031323	1.87 × 10^−115^
**Molecular functions (five most enriched)**		
Enzyme binding	0019899	1.02 × 10^−93^
Protein binding	0005515	1.58 × 10^−92^
Binding	0005488	1.39 × 10^−60^
Transcription factor binding	0008134	8.70 × 10^−51^
Kinase binding	0019900	2.08 × 10^−59^
**Cellular components (five most enriched)**		
Nucleoplasm	0005654	1.67 × 10^−95^
Nuclear lumen	0031981	5.16 × 10^−83^
Nucleus	0005634	4.98 × 10^−82^
Intracellular organelle lumen	0070013	1.14 × 10^−73^
Cytosol	0005829	3.48 × 10^−65^

## Data Availability

The data present in the current study are available from the corresponding author on reasonable request.

## Supplementary Materials

The following supporting information can be downloaded at https://www.mdpi.com/article/10.3390/ijms24032151/s1.

## Appendix A

The hydrophobic cluster analysis (HCA) provides a graphical representation of the sequence that enables identifying disordered regions [249]. Although HCA was not originally conceived to predict disorder, it is very useful for identifying disordered regions. HCA outputs can be obtained from https://mobyle.rpbs.univ-paris-diderot.fr/cgi-bin/portal.py#forms::HCA (accessed on 1 December 2022). HCA provides a two-dimensional helical representation of protein sequences in which hydrophobic clusters are plotted along the sequence. Glycine residues are represented as diamonds, prolines as stars, threonines as squares, and serines as squares containing a dot. Acidic and basic residues are in red and in blue, respectively, and hydrophobic residues are shown in green. Disordered regions are recognizable as they are depleted in (or devoid of) hydrophobic clusters. HCA also enables identifying short regions with propensity to fold as they look like regions locally enriched in small hydrophobic clusters within regions otherwise devoid of such clusters.
