# Supplementary material for: Liaisons dangereuses: Intrinsic Disorder in Cellular Proteins Recruited to Viral Infection-Related Biocondensates
Source: Int J Mol Sci. 2023 Jan 21;24(3):2151. doi: 10.3390/ijms24032151 (PMC9917183; doi:10.3390/ijms24032151)
Supplement: Supplementary file 1 [file ijms-24-02151-s001.zip › ijms-2106084-supplementary.pdf]

Supplementary Material

Liaisons dangereuses: Intrinsic Disorder in Cellular Proteins Recruited to Viral Infection-Related Biocondensates

Greta Bianchi [1], Stefania Brocca [1,*], Sonia Longhi [2,*] and Vladimir N. Uversky [3]

[1] Department of Biotechnology and Biosciences, University of Milano-Bicocca, 20126 Milano, Italy; g.bianchi31@campus.unimib.it
[2] Laboratoire Architecture et Fonction des Macromolécules Biologiques (AFMB), UMR 7257, Aix Marseille University and CNRS, 13288 Marseille, France
[3] Department of Molecular Medicine, Byrd Alzheimer's Research Institute, Morsani College of Medicine, University of South Florida, Tampa, FL 33601, USA; vuversky@usf.edu
* Correspondence: stefania.brocca@unimib.it (S.B.); sonia.longhi@univ-amu.fr (S.L.)

Supplementary Table S1. Structural information about proteins analyzed in this study

**RNA-binding protein FUS (23 PDB entries; 526 residues)**						
PDB ID	Method	Resolution	Chain	Position	Image	Reference
6SNJ	NMR		A	260-390	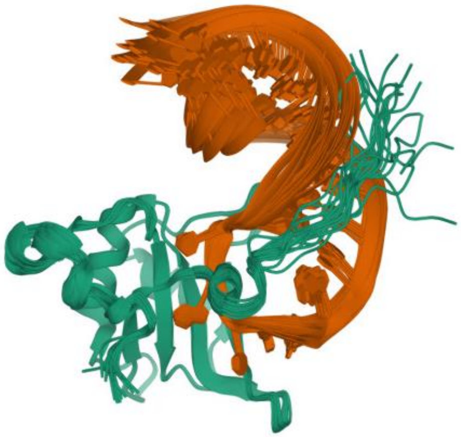	PubMed: 33311468<br>Note: Complex of Complex of the FUS/TLS RNA recognition motif (green) with U1 snRNA stem loop III (orange)
2LCW	NMR		A	278-385	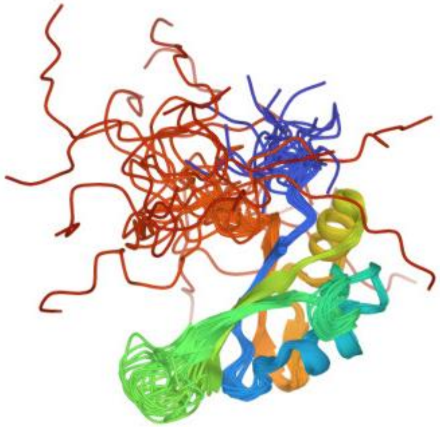	PDB DOI: 10.2210/pdb2LCW/pdb<br>Note: solution structure of the FUS/TLS RRM domain

6GBM	NMR		B	280-377	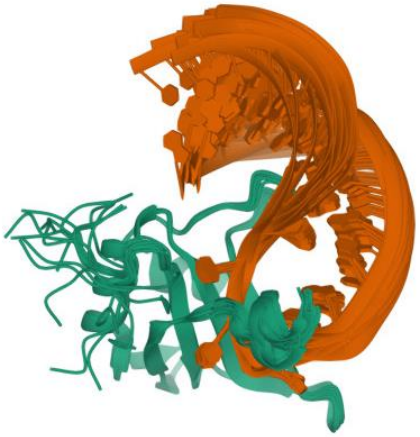	PubMed: 30581145<br>Note: Complex of an RRM domain of FUS (green) with RNA (orange)
2LA6	NMR		A	282-370	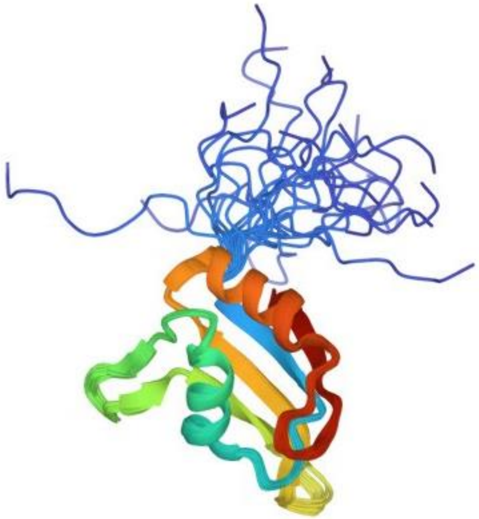	PDB DOI: 10.2210/pdb2LA6/pdb<br>Note: Solution structure of RRM domain of RNA-binding protein FUS

6G99	NMR		B	419-454	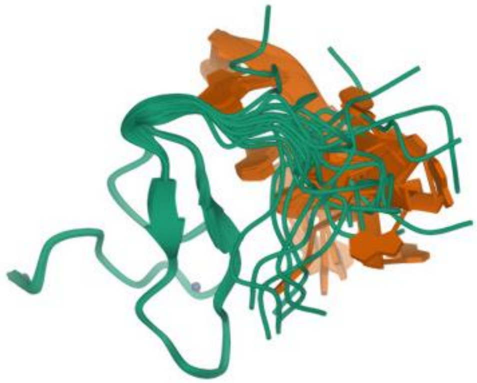	PubMed: 30581145 Note: Complex of a zinc-finger domain of FUS (green) with RNA (UGGUG; orange)
5YVG	X-ray	4.05	X/Y	1-526	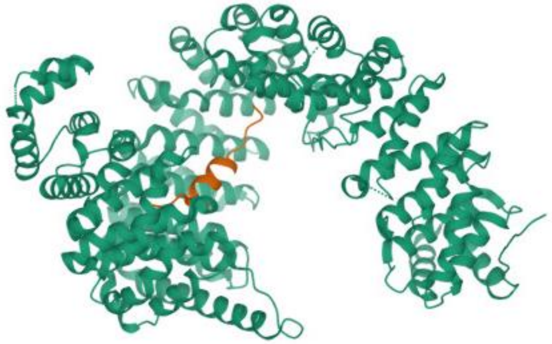	PubMed: 29677513 Note: Complex of Transportin-1 also known as karyopherin-β2 (green) with full length FUS (orange). Only residues 517-523 are resolved; remaining part of FUS are not modeled (missing electron density region)
7CYL	X-ray	2.70 Å	B	476-526	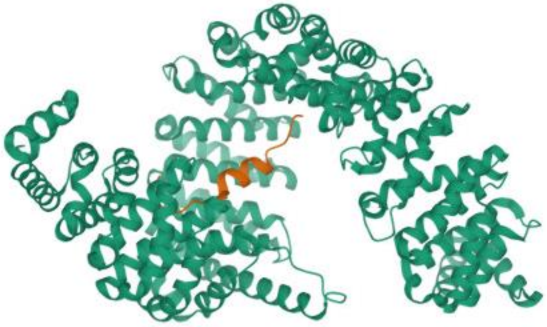	PubMed: 33580145 Note: Complex of Transportin-1 also known as karyopherin-β2 karyopherin-β2 (green) with full length FUS (orange). Only residues 513-521 are resolved; remaining parts of FUS are not modeled (missing electron density regions)

4FDD	X-ray	2.30 Å	B	498-526	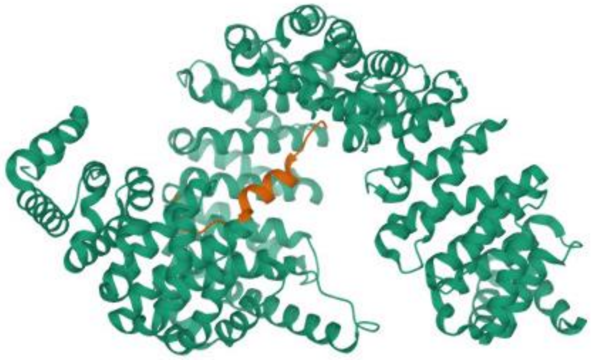	PubMed: 22778397 Note: Complex of Transportin-1 also known as karyopherin-β2 (green) with a FUS fragment (orange); Only residues 513-522 are resolved; remaining parts of this FUS fragment are not modeled (missing electron density regions)
4FQ3	X-ray	3.00 Å	B	495-526	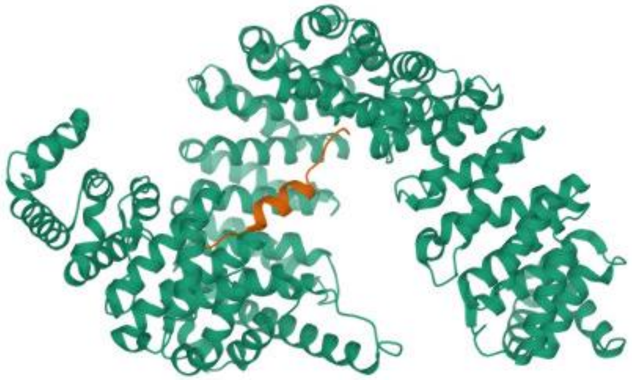	PDB DOI: 10.2210/pdb4FQ3/pdb Note: Complex of Transportin-1 also known as karyopherin-β2 (green) with a FUS fragment (orange); Only residues 513-522 are resolved; remaining parts of this FUS fragment are not modeled (missing electron density regions)

Mitochondrial antiviral-signaling protein MAVS (10 PDB entries; 540 residues)						
PDB ID	Method	Resolution	Chain	Position	Image	Reference
2MS8	NMR		A	1-100	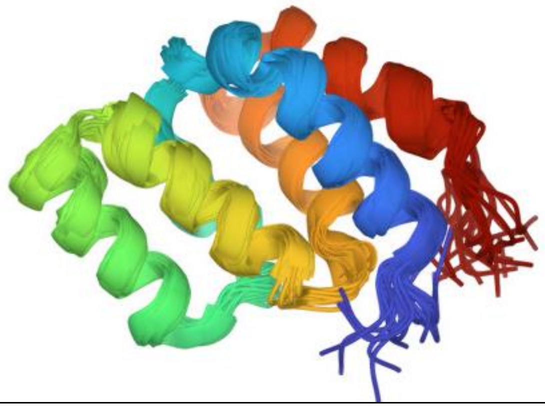	PubMed: 26733681<br>Note: Solution NMR structure of MAVS CARD
2MS7	NMR		A/B/C/D/<br>E/F/G/H/I/<br>J/K/L/M/N/<br>O/P/Q/R/<br>S/T/U	1-100	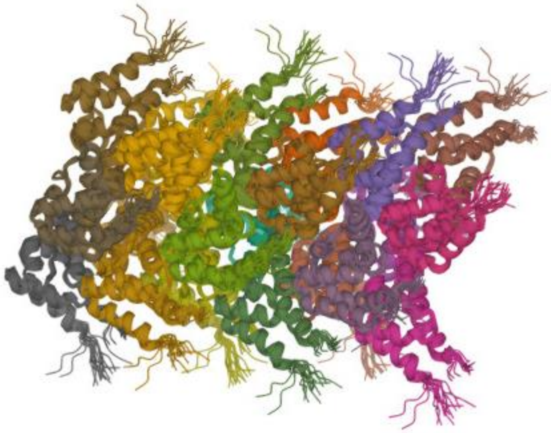	PubMed: 26733681<br>Note: Solid-state NMR structure of the helical signal transduction filament MAVS CARD

5JEK		2.40 Å	C/D	433-448	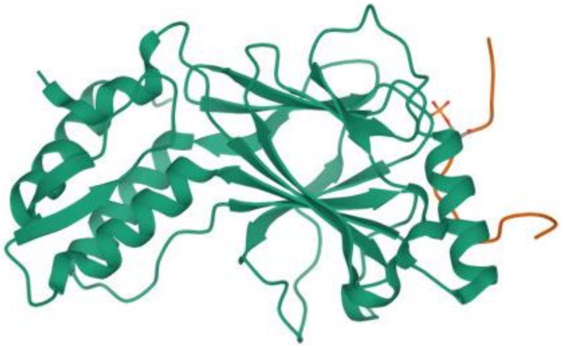	PubMed: 27302953<br>Note: Phosphorylated MAVS (orange) in complex with IRF-3 (green)
4Z8M	X-ray	2.95 Å	C/D	450-468	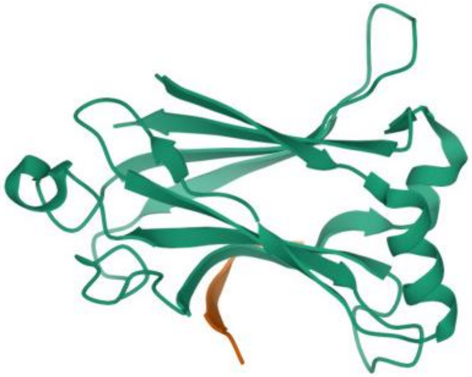	PubMed: 26385923<br>Note: A complex of MAVS (orange) with TRAF6 (green)

Heterogeneous nuclear ribonucleoprotein A2/B1 (hnRNPA2B1; 9 PDB entries; 353 residues)						
PDB ID	Method	Resolution	Chain	Position	Image	Reference
1X4B	NMR		A	1-103	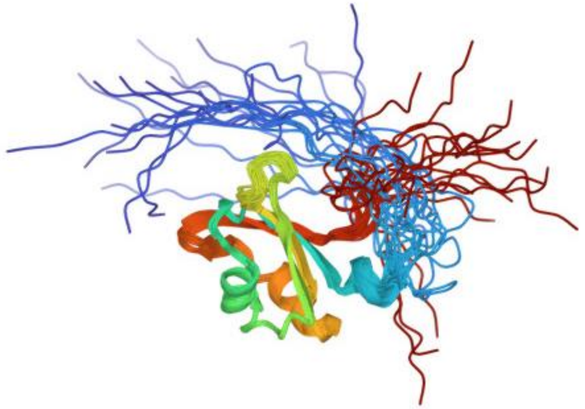	PDB DOI: 10.2210/pdb1X4B/pdb Note: Solution structure of RRM domain of hnRNPA2
5EN1	X-ray	2.58 Å	A	12-195	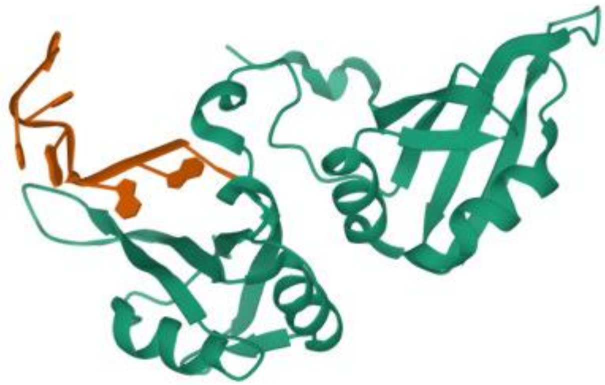	PDB DOI: 10.2210/pdb5EN1/pdb Note: Crystal structure of hnRNPA2B1 (green) in complex with RNA (orange)

Cellular tumor antigen p53 (TP53; 246 PDB entries; 393 residues)						
PDB ID	Method	Resolution	Chain	Position	Image	Reference
6XRE	Cryo-EM	4.60 Å	M	1-393	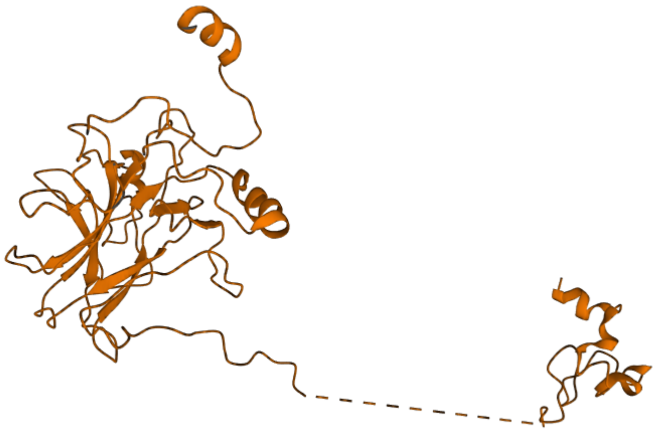	PubMed: 33767390 Note: Structure of p53 is computationally extracted from the p53/RNA polymerase II assembly, which includes DNA-directed RNA polymerase II subunits RPB1, RPB2, RPB3, RPB4, RPB5, RPABC2, RPB7, RPABC3, RPB9, RPABC5, RPB11-a, RPABC4, and p53
2LY4	NMR		A	1-93	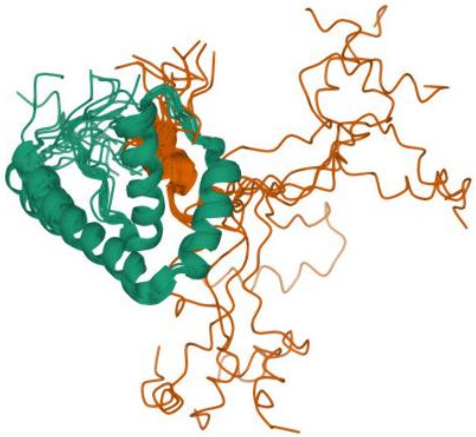	PubMed: 23063560 Note: Solution structure of a complex between high mobility group protein B1 (green) and a p53 transactivation domain (orange)

2MEJ	NMR			96-312	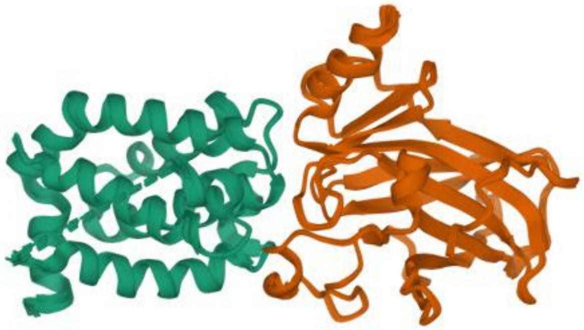	PubMed: 24814347<br>Note: Solution structure of a complex between BCL-xL (green) and the p53 core (DNA-binding) domain (orange)
1AIE	X-ray	1.50 Å	A	326-356	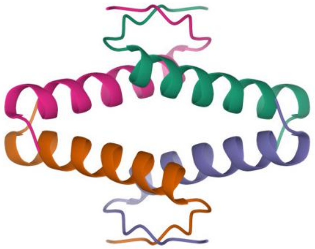	PubMed: 9761820<br>Note: Crystal structure of the homo-tetramer of the p53 tetramerization domain
1JSP	NMR		A	367-386	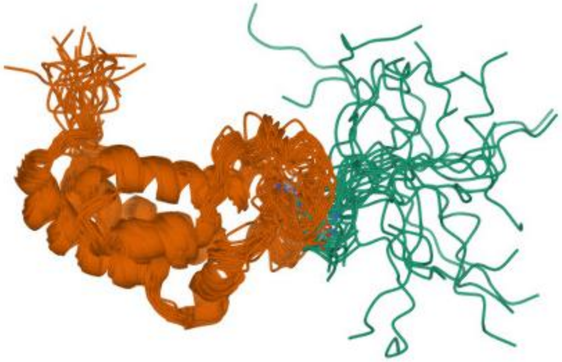	PubMed: 14759370<br>Note: solution structure of the Bromodomain of CREB-binding protein (orange) in a complex with the C-terminal negative regulatory domain of p53 (green)

6R5L	X-ray	1.88 Å	P	385-393	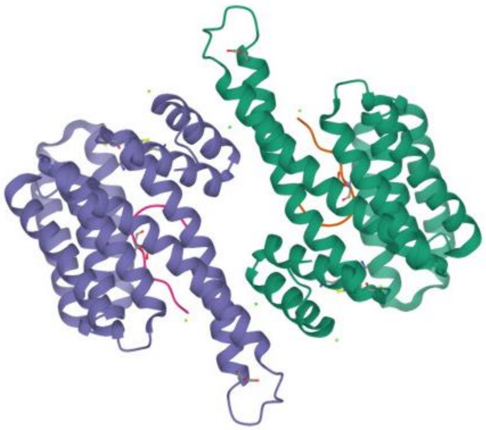	PubMed: 32501690<br>Note: Crystal structure of a 14-3-3 sigma dimer (green and blue) with a C-terminal phosphorylated fragment of p53 (p53pT387, orange)
**Ras GTPase-activating protein-binding protein 2 (G3BP2; 1 PDB entry; 482 residues)**						
PDB ID	Method	Resolution	Chain	Position	Image	Reference
5DRV	X-ray	2.75 Å	A	1-139	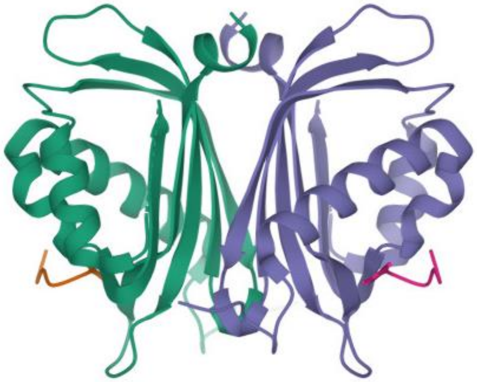	PubMed: 26410532<br>Note: A crystal structure of the G3BP2 NTF2-like domain (green and blue) in complex with a canonical FGDF motif peptide (orange and red)

Transcription factor p65 (Nuclear factor NF-kappa-B p65 subunit; 88 PDB entries; 551 residues)						
PDB ID	Method	Resolution	Chain	Position	Image	Reference
1NFI	X-ray	2.70 Å	A/C	20-320	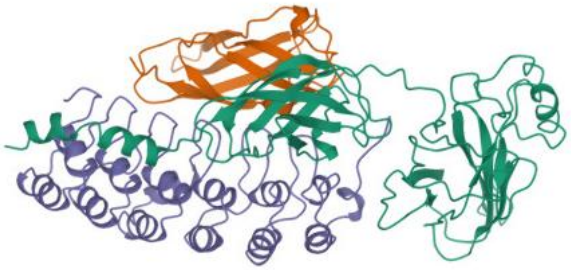	PubMed: 9865693<br>Note: A crystal structure of the NF-kappa-B p65 (green) – NF-kappa-B p50 (orange) – I - kappa-B-alpha (blue) complex.
5U4K	NMR		B	521-551	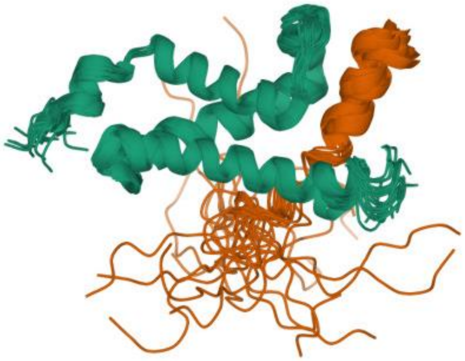	PubMed: 28334776<br>Note: NMR structure of the complex between the KIX domain of CBP (green) and the transactivation domain 1 of p65 (orange)
**Ras GTPase-activating protein-binding protein 1 (G3BP1; 7 PDB entries; 466 residues)**						
PDB ID	Method	Resolution	Chain	Position	Image	Reference

4FCJ	X-ray	1.62 Å	A/B	1-139	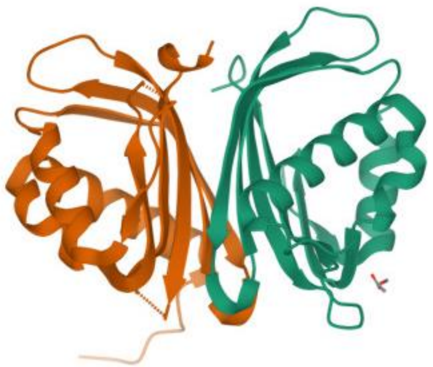	PubMed: 24324649<br>Note: Crystal homodimeric structure of the NTF2-like domain of human G3BP1
**TAR DNA-binding protein 43 (TDP-43; 34 PDB entries; 414 residues)**						
PDB ID	Method	Resolution	Chain	Position	Image	Reference
2N4P	NMR		A	1-77	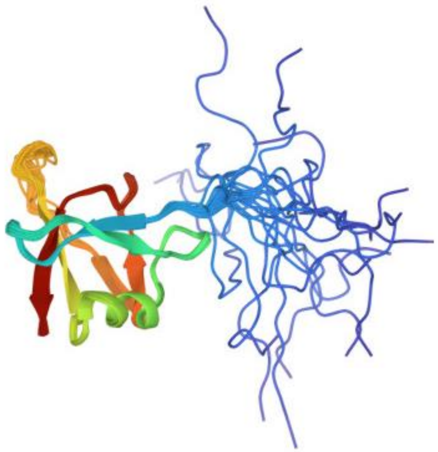	PubMed: 26756435<br>Note: Solution structure of the N-terminal domain of TDP-43

2CQG	NMR		A	96-185	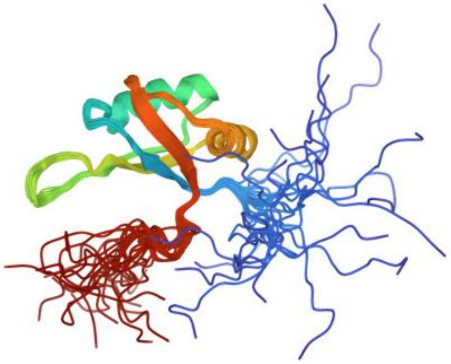	PDB DOI: 10.2210/pdb2CQG/pdb Note: Solution structure of the RNA binding domain of TAR DNA-binding protein-43
1WF0	NMR		A	193-267	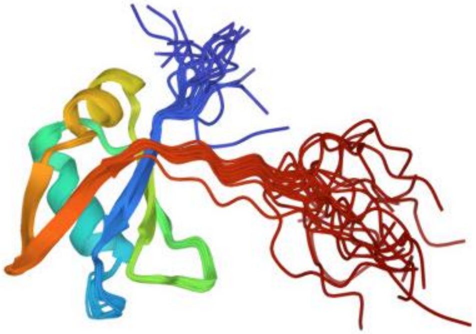	PDB DOI: 10.2210/pdb1WF0/pdb Note: Solution structure of RRM domain in TAR DNA-binding protein-43
2N3X	NMR		A	311-360	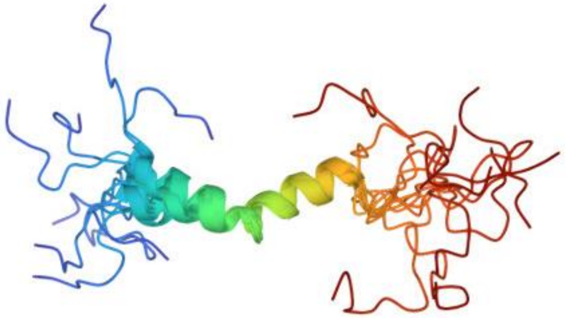	Note: Solution structure of TDP-43 amyloidogenic core region
**Nuclear transport factor 2 (NTF2) family protein with RNA binding (RRM-RBD-RNP motifs) domain-containing protein (no experimental structure; 458 residues)**						
**Melanoma differentiation-associated protein 5 (MDA-5; 8 PDB entries; 1,025 residues)**						
PDB ID	Method	Resolution	Chain	Position	Image	Reference

7DNJ	Cryo-EM	3.30 Å	A/B/C/D	1-208	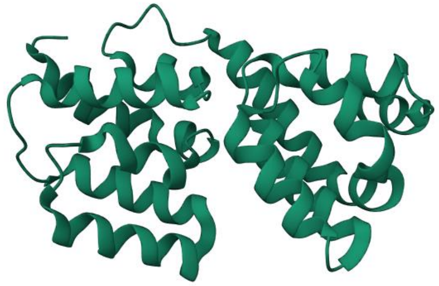	PubMed: 34644557<br>Note: Structure is computationally extracted from K63-polyUb MDA5CARDs complex containing 4 MDA- chains and 8 ubiquitin chains
7JL0	Cryo-EM	4.30 Å	A	287-<br>1025	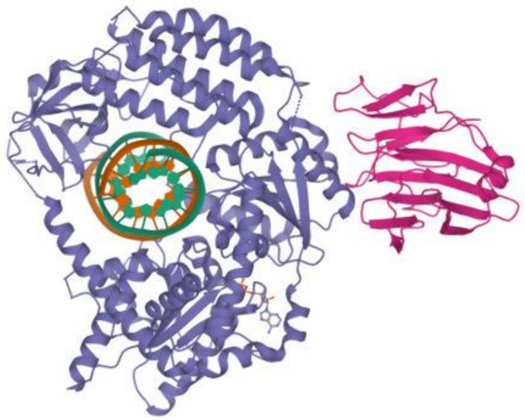	PubMed: 33373584<br>Cryo-EM structure of MDA5 (blue) - dsRNA (green and orange) in complex with TRIM65 PSpry domain (pink)
**Fibrillarin 2 (no experimental structure; 314 residues)**						
**Focal adhesion kinase 1 (FAK1; 35 PDB entries; 1,052 residues)**						
PDB ID	Method	Resolution	Chain	Position	Image	Reference

4NY0	X-ray	2.80 Å	A/B/C/D	31-405	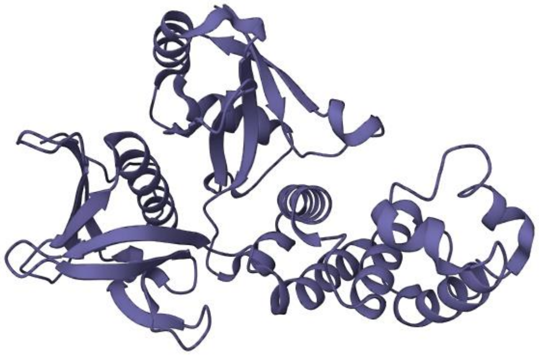	PubMed: 24480479<br>Note: Crystal structure of FERM domain of human focal adhesion kinase
1MP8	X-ray	2.80 Å	A	406-686	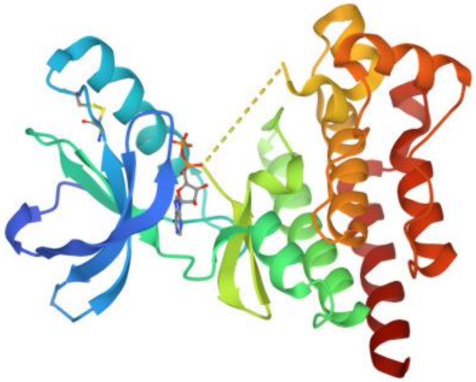	PubMed: 12467573<br>Note: Structure contains 3 regions with missing electron density: residues 406-413, 445-446, and 565-583
1K04	X-ray	1.95 Å	A	891-1052	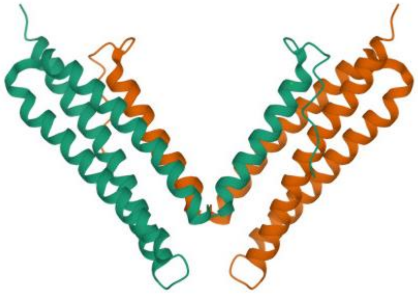	PubMed: 12005431<br>Note: Crystal structure of the focal adhesion targeting domain of focal adhesion kinase
**Nucleolysin TIAR (TIAR1; 3 PDB entries; 375 residues)**						
PDB ID	Method	Resolution	Chain	Position	Image	Reference

2CQI	NMR		A	1-90	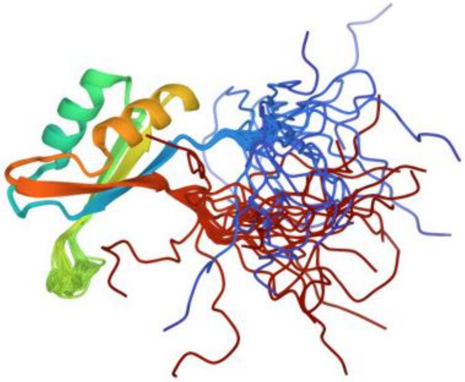	PDB DOI: 10.2210/pdb2CQI/pdb Note: Solution structure of the RNA binding domain of Nucleolysin TIAR
2DH7	NMR		A	89-180	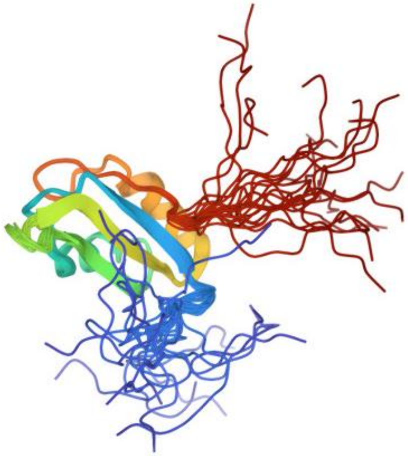	PDB DOI: 10.2210/pdb2DH7/pdb Note: Solution structure of the second RNA binding domain in Nucleolysin TIAR
1X4G	NMR		A	187-282	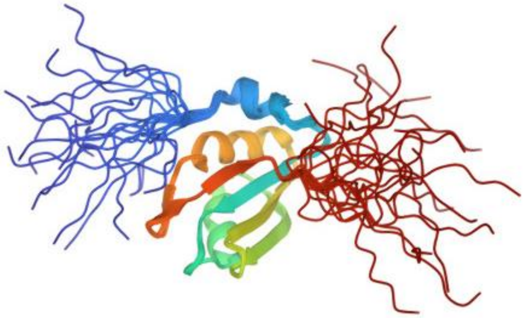	PDB DOI: 10.2210/pdb1X4G/pdb Solution structure of RRM domain in Nucleolysin TIAR

Heat shock 70 kDa protein 1A (HSP70-1A; 44 PDB entries; 641 residues)						
PDB ID	Method	Resolution	Chain	Position	Image	Reference
1S3X	X-ray	1.84 Å	A	1-382	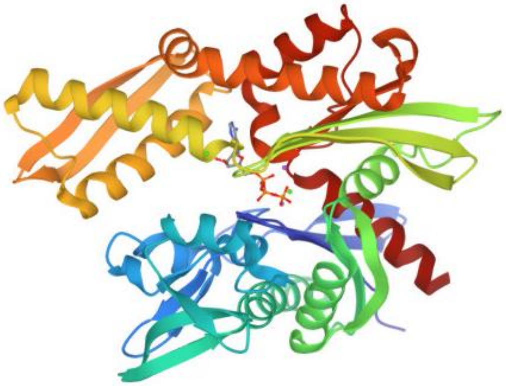	PubMed: 9083109<br>Note: The crystal structure of the human Hsp70 ATPase domain (or Nucleotide-binding domain (NBD))
5GJJ	NMR		A	385-641	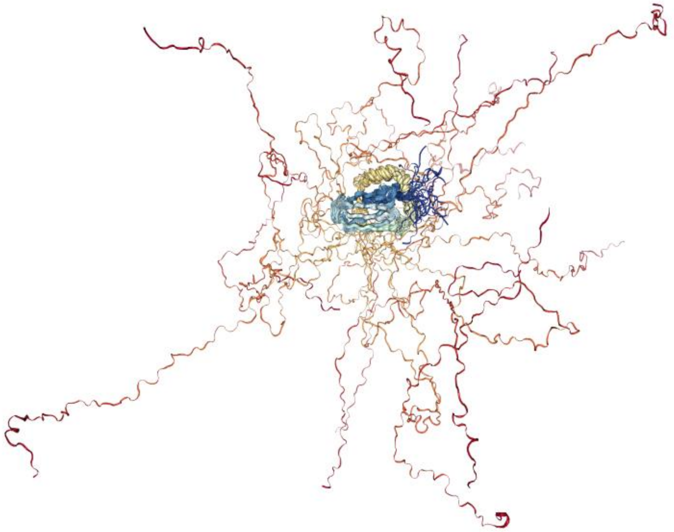	PDB DOI: 10.2210/pdb5GJJ/pdb<br>Note: Solution structure of substrate-binding domain (SBD)

**Ras-related protein Rab-11A (RAB11; 28 PDB entries; 216 residues)**						
PDB ID	Method	Resolution	Chain	Position	Image	Reference
5FBL	X-ray	3.37 Å	B	1-216	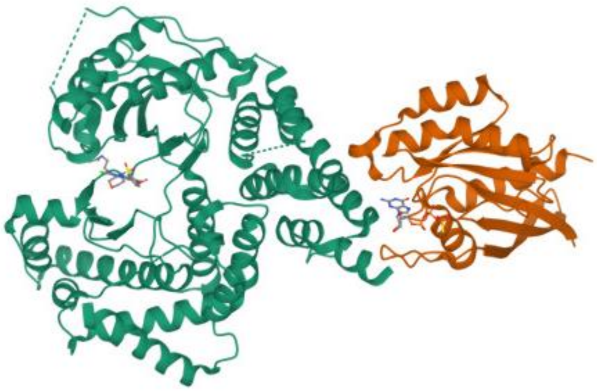	PDB DOI: 10.2210/pdb5FBL/pdb Note: Crystal structure of a complex between Phosphatidylinositol 4-kinase beta (green) and RAB11 (orange); in RAB11 structure, residues 108, 68-73, and 177-216 are regions of missing electron density

**Serine/threonine-protein phosphatase PP1-alpha catalytic subunit (PP1; 36 PDB entries; 330 residues)**						
PDB ID	Method	Resolution	Chain	Position	Image	Reference
3EGG	X-ray	1.85 Å	A/B	7-330	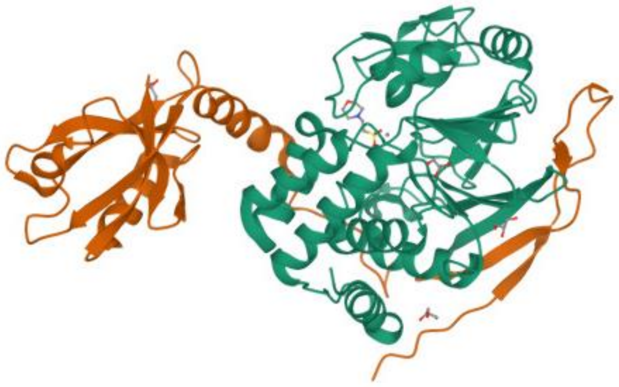	PubMed: 20305656 Note: Crystal structure of a complex between Protein Phosphatase 1 alpha (PP1; green) and the PP1 binding and PDZ domains of Spinophilin (orange); in PP1 structure, residues 301-330 represent a region of missing electron density

Glutamine-dependent carbamoyl-phosphate synthase (CAD; 34 PDB entries; 2,225 residues)						
PDB ID	Method	Resolution	Chain	Position	Image	Reference
4C6B	X-ray	1.66 Å	A	1454-1846	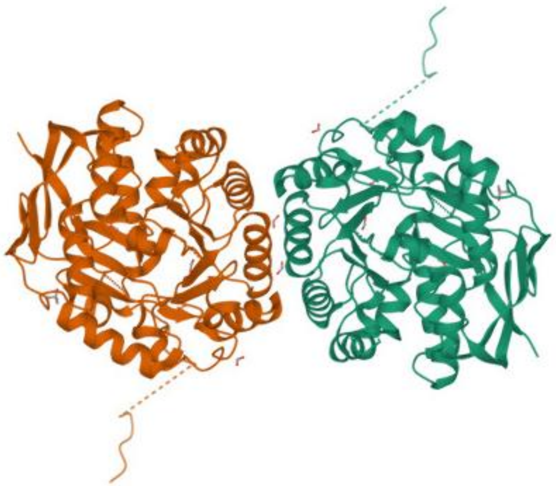	PubMed: 24332717<br>Note: Crystal structure of the dihydroorotase domain of human CAD; residues 1454-1459, 1476-1482, 1650-1657, 1697-1698, 1824-1830, and 1840-1846 are missing
5G1N	X-ray	2.10 Å	A/B/C/D/E/F	1912-2225	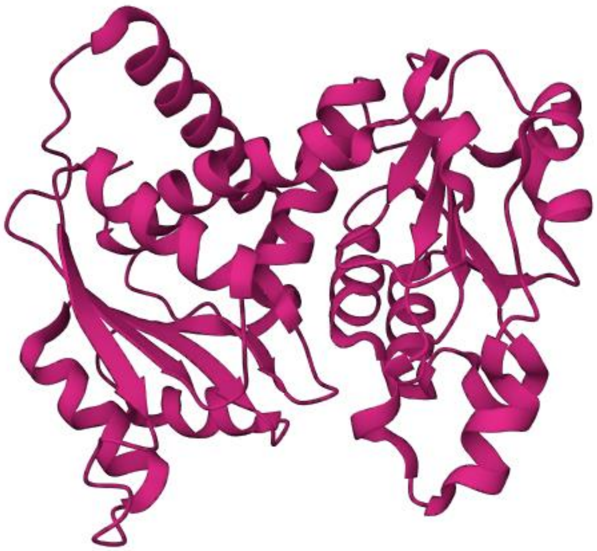	PubMed: 27265852<br>Note: Crystal structure of the aspartate transcarbamoylase domain of human CAD; residues 1912-1919 are missing

Mitogen-activated protein kinase 14 (MAPK14; 245 PDB entries; 360 residues)						
PDB ID	Method	Resolution	Chain	Position	Image	Reference
2LGC	NMR		A	2-354	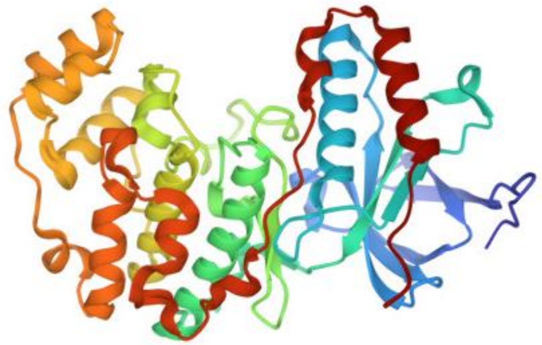	PubMed: 22275118<br>Note: NMR solutions structure of MAPK14

UDP-N-acetylglucosamine--peptide N-acetylglucosaminyltransferase 110 kDa subunit or O-linked N-acetylglucosamine transferase 110 kDa subunit (OGT; 38 PDB entries; 1,046 residues)						
PDB ID	Method	Resolution	Chain	Position	Image	Reference
6EOU	X-ray	1.75 Å	A	23-410	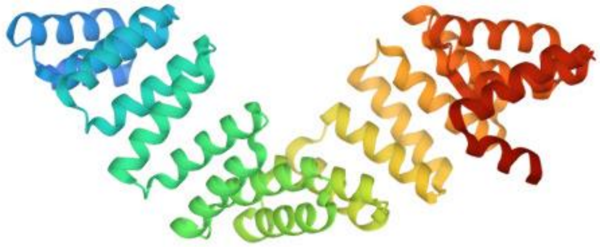	PubMed: 29606577<br>Note: Crystal structure of the TPR domain of O-linked β-N-acetyl- D -glucosamine (O-GlcNAc) transferase (OGT); residues 23-57 and 401-410 represent regions of missing electron density

6IBO	X-ray	2.17 Å	A	319-1041	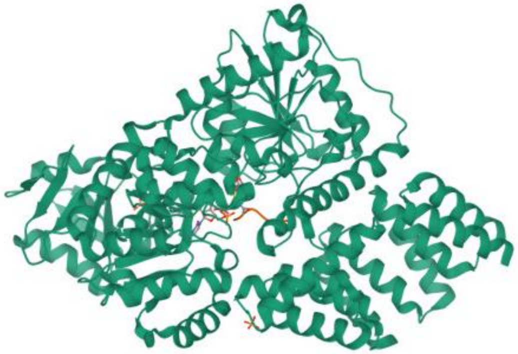	PubMed: 31296563<br>Note: Cristal structure of the catalytic OGT domain; residues 319-324, 726-728, 757-771, and 1038-1041 form regions of missing electron density

Supplementary Figures S1–S18

All supplementary figures have identical structural organization, where panel **A** shows the amino acid sequence of the query protein in FASTA format, panel **B** represents the RIDAO-generated disorder profile, panel **C** shows the D^2P^2-generated functional disorder profile (where available), panel **D** gives the FuzDrop output, panel **E** represents the AlphaFold2-generated structure with FuzDrop annotations (where available), panel **F** represents the STRING-generated PPI network with corresponding statistics (where available), panel **G** shows the MeDor output and panel **H** shows the plot of the *per* residue disorder score (as provided by PONDR® VLS2) *versus* (100-pLDDT), where pLDDT is the corresponding AlphaFold2 confidence score. For detailed information on the RIDAO and MeDor outputs see Figure 3 legend.

Supplementary Figure S1. Functional disorder in TAR DNA-binding protein 43. **A.** Amino acid sequence. **B.** RIDAO-generated disorder profile. **C.** D2P2-generated functional disorder profile. **D.** FuzDrop output. **E.** AlphaFold2 structure with FuzDrop annotations. **F.** STRING-generated PPI network with corresponding statistics. **G.** MeDor output. **H.** Plot of the *per* residue disorder score (as provided by PONDR® VLS2) versus (100-pLDDT), where pLDDT is the corresponding AlphaFold2 confidence score.

A

>sp|Q13148|TADBP_HUMAN TAR DNA-binding protein 43 OS=Homo sapiens OX=9606 GN=TARDBP PE=1 SV=1
MSEYIRVTEDENDEPIEIPSEDDGTVLLSTVTAQFPGACGLRYRNPVSQCMRGVRLVEGILHAPDAGWGNLVYVVNYPKDNKRKMDETDASSAVKVKRAVQKTSDLIVLGLPWKTTEQ
DLKEYFSTFGEVLMVQVKKDLKTGHSKGFGFVRFTEYETQVKVMSQRHMIDGRWCDCKLPNSKQSQDEPLRSRKVFVGRCTEDMTEDELREFFSQYGDVMDVFIPKPFRAFAFVTFAD
DQIAQSLCGEDLIIKGISVHISNAEPKHNSNRQLERSGRFGGNPGGFGNQGGFGNSRGGGAGLGNNQGSNMGGGMNFGAFSINPAMMAAAQAALQSSWGMMGMLASQQNQSGPSGNNQ
NQGNMQREPNQAFGSGNNSYSGSNSGAAIGWGSASNAGSGSGFNGGFGSSMDSKSSGWGM

B

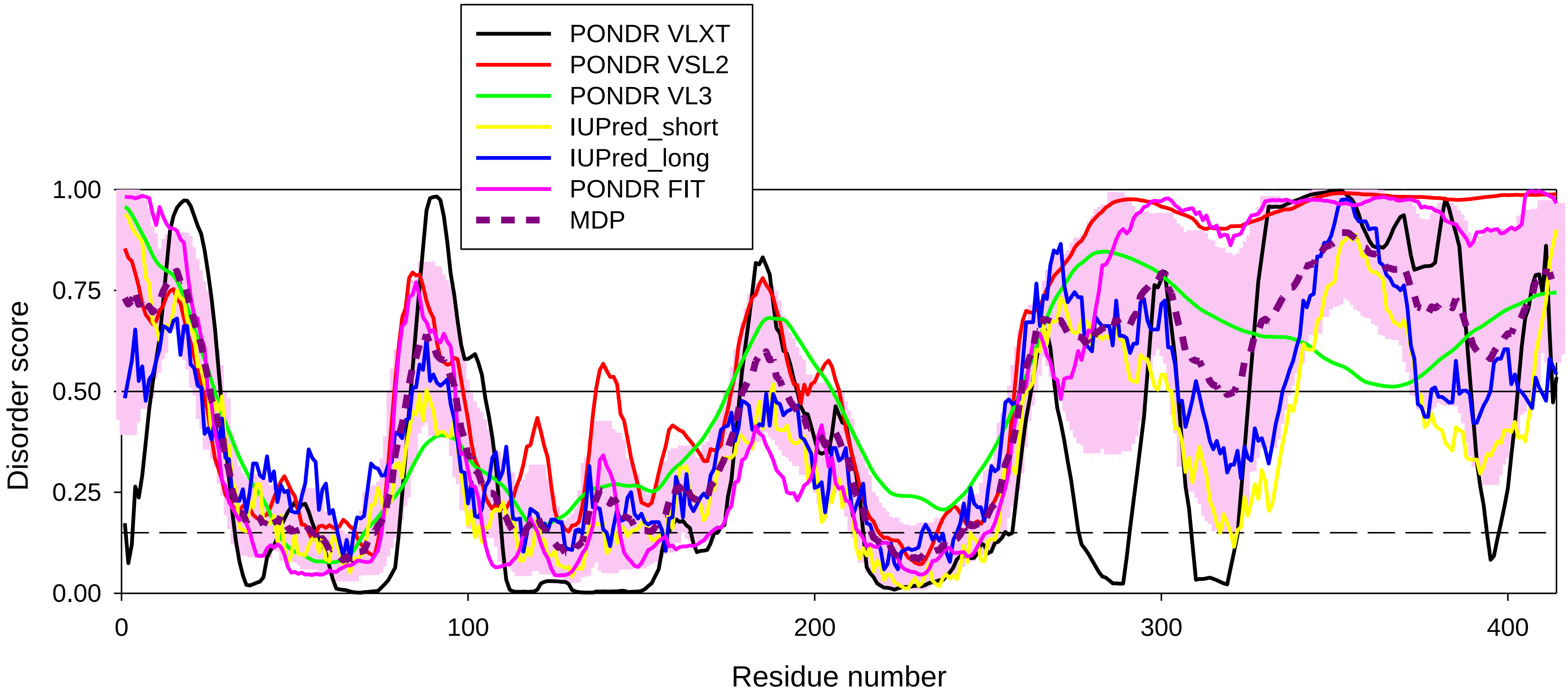

C

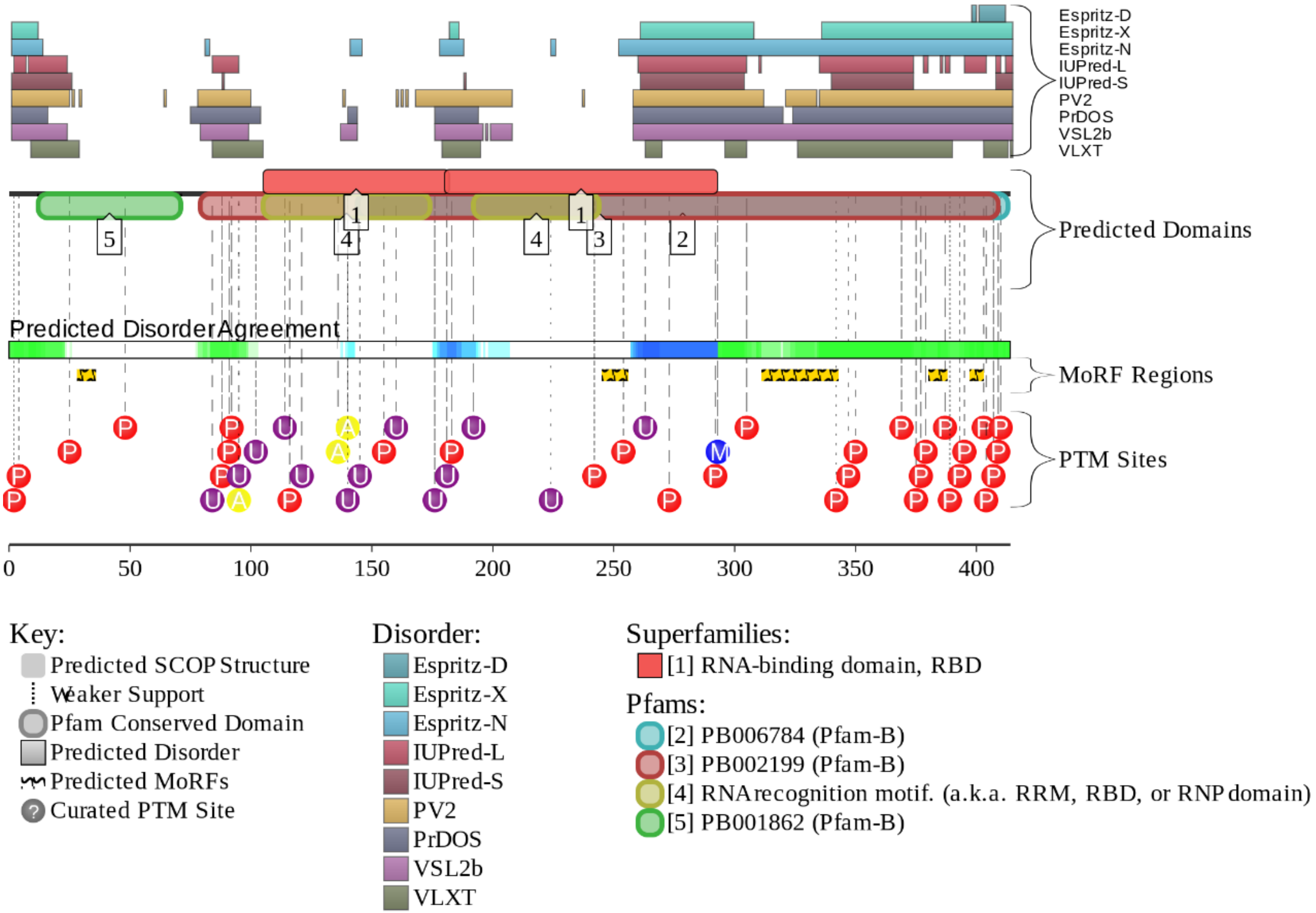
Espritz-D
Espritz-X
Espritz-N
IUPred-L
IUPred-S
PV2
PrDOS
VSL2b
VLXT
Predicted Domains
Predicted Disorder Agreement
MoRF Regions
PTM Sites
0 50 100 150 200 250 300 350 400
Key:
Predicted SCOP Structure
Weaker Support
Pfam Conserved Domain
Predicted Disorder
Predicted MoRFs
Curated PTM Site
Disorder:
Espritz-D
Espritz-X
Espritz-N
IUPred-L
IUPred-S
PV2
PrDOS
VSL2b
VLXT
Superfamilies:
[1] RNA-binding domain, RBD
Pfams:
[2] PB006784 (Pfam-B)
[3] PB002199 (Pfam-B)
[4] RNA recognition motif. (a.k.a. RRM, RBD, or RNP domain)
[5] PB001862 (Pfam-B)

D

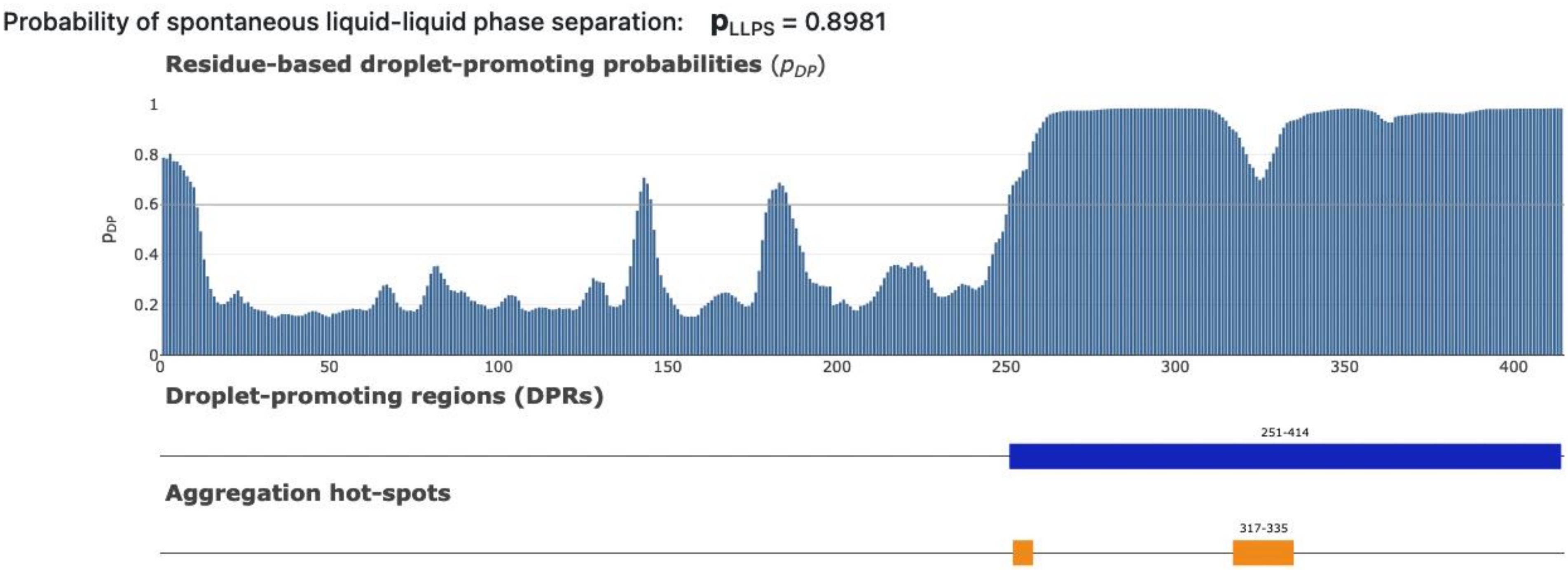

Probability of spontaneous liquid-liquid phase separation: pLLPS = 0.8981
Residue-based droplet-promoting probabilities (pDP)
pDP
1
0.8
0.6
0.4
0.2
0
0
50
100
150
200
250
300
350
400
Droplet-promoting regions (DPRs)
251-414
Aggregation hot-spots
317-335

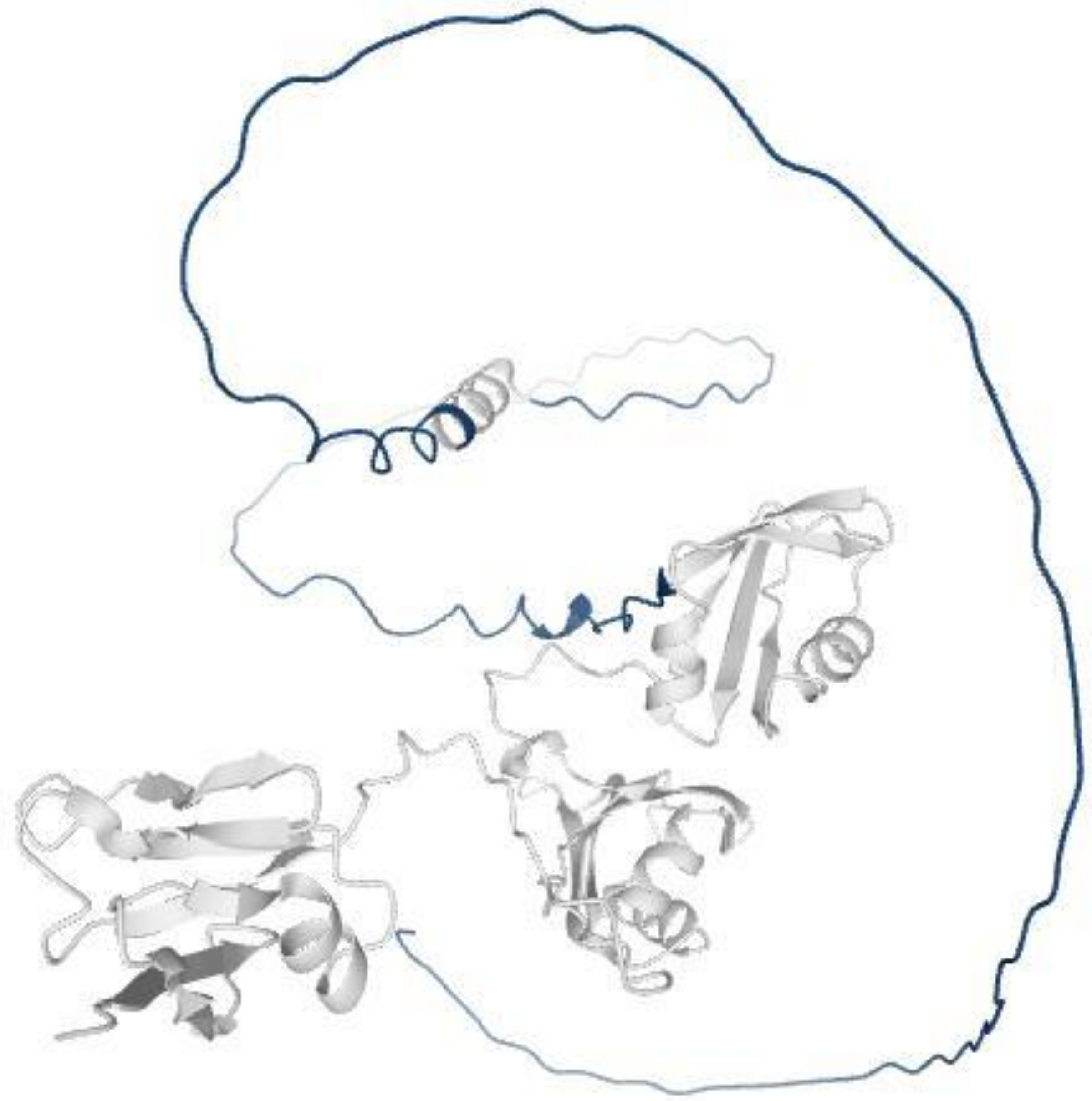

E

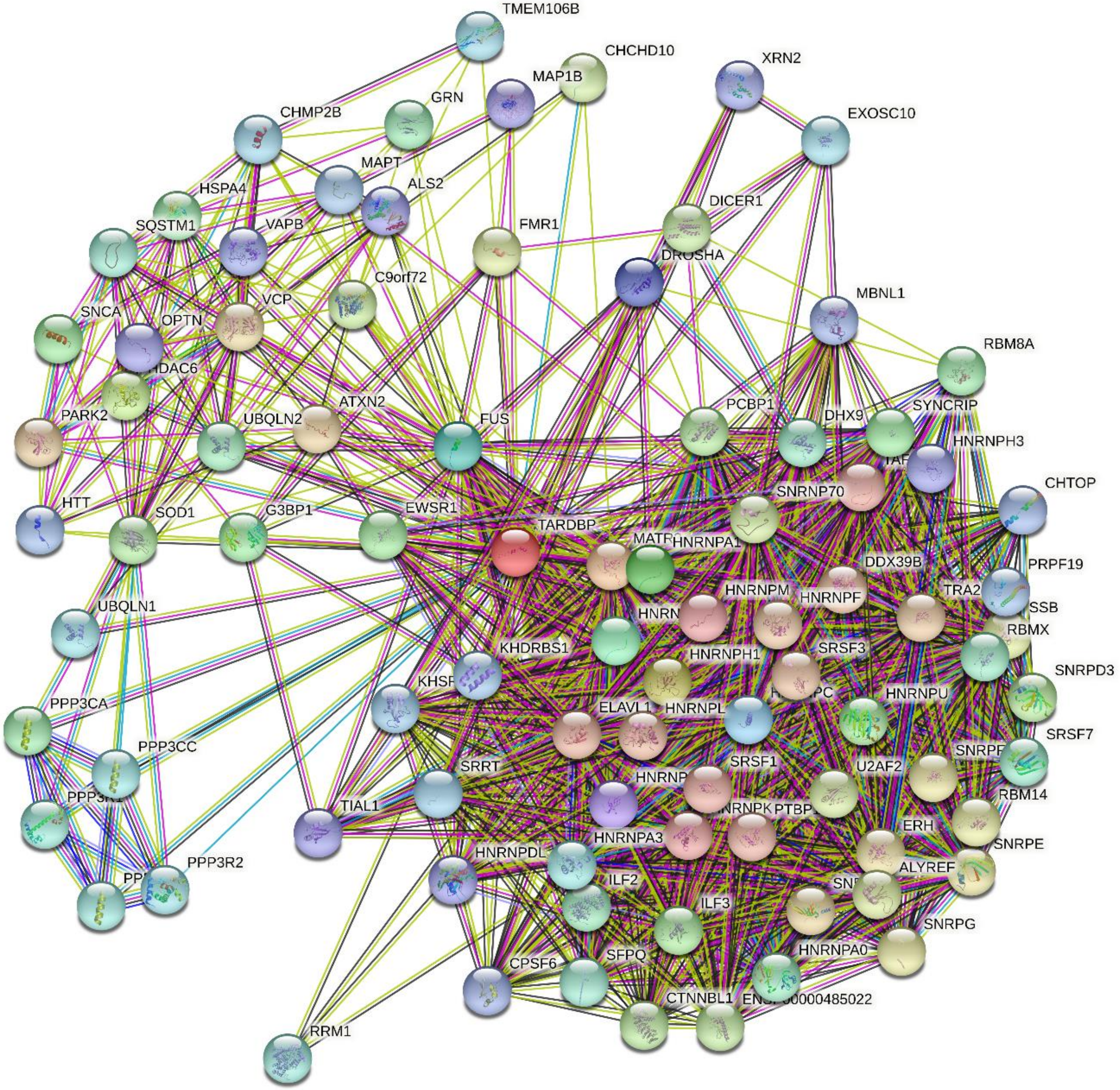
TMEM106B
CHCHD10
XRN2
MAP1B
GRN
CHMP2B
EXOSC10
MAPT
ALS2
HSPA4
DICER1
SQSTM1
VAPB
FMR1
DROSHA
C9orf72
SNCA
VCP
OPTN
MBNL1
HDAC6
RBM8A
ATXN2
PARK2
PCBP1
UBQLN2
FUS
DHX9
SYNCRIP
HNRNPH3
CHTOP
SNRNP70
HTT
SOD1
G3BP1
EWSR1
TARDBP
HNRNPA1
DDX39B
PRPF19
HNRNPM
HNRNPF
TRA2
SSB
UBQLN1
RBMX
KHDRBS1
SRSF3
SNRPD3
HNRNPH1
HNRNPU
PPP3CA
ELAVL1
SRSF7
PPP3CC
SRRT
SRSF1
U2AF2
RBM14
TIAL1
HNRNPK
ERH
SNRPE
PPP3R2
HNRNPA3
HNRNPDL
ALYREF
ILF2
ILF3
SNRPG
HNRNPA0
CPSF6
SFPQ
CTNNBL1
RRM1

F

minimum required interaction score: high confidence (0.700)

number of nodes:	88
number of edges:	1297
average node degree:	29.5
avg. local clustering coefficient:	0.795
expected number of edges:	159
PPI enrichment p-value:	< 1.0e-16

G

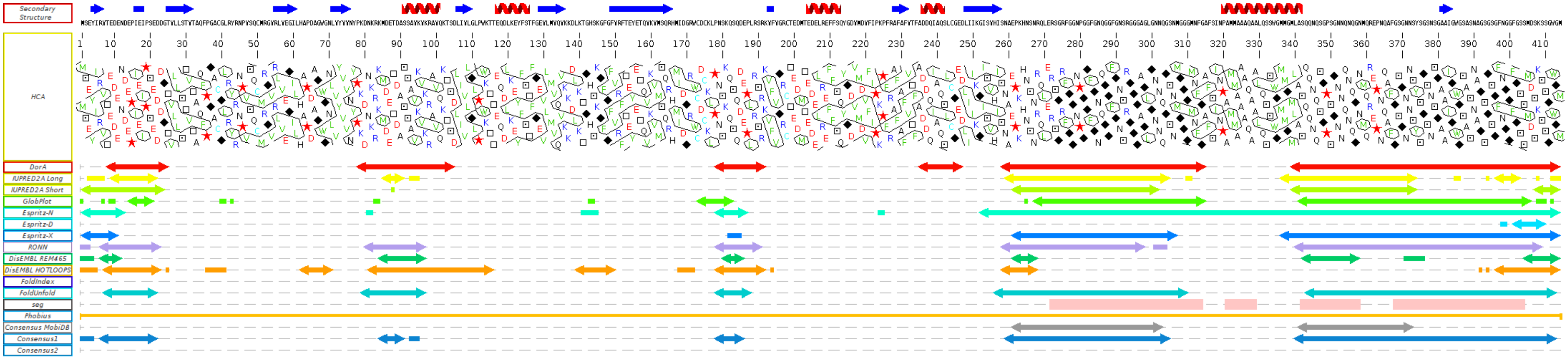

Secondary Structure
HCA
DorA
IUPRED2A Long
IUPRED2A Short
GlobPlot
Espritz-N
Espritz-D
Espritz-X
RONN
DisEMBL REM465
DisEMBL HOTLOOPS
FoldIndex
FoldUnfold
seg
Phobius
Consensus MobiDB
Consensus1
Consensus2

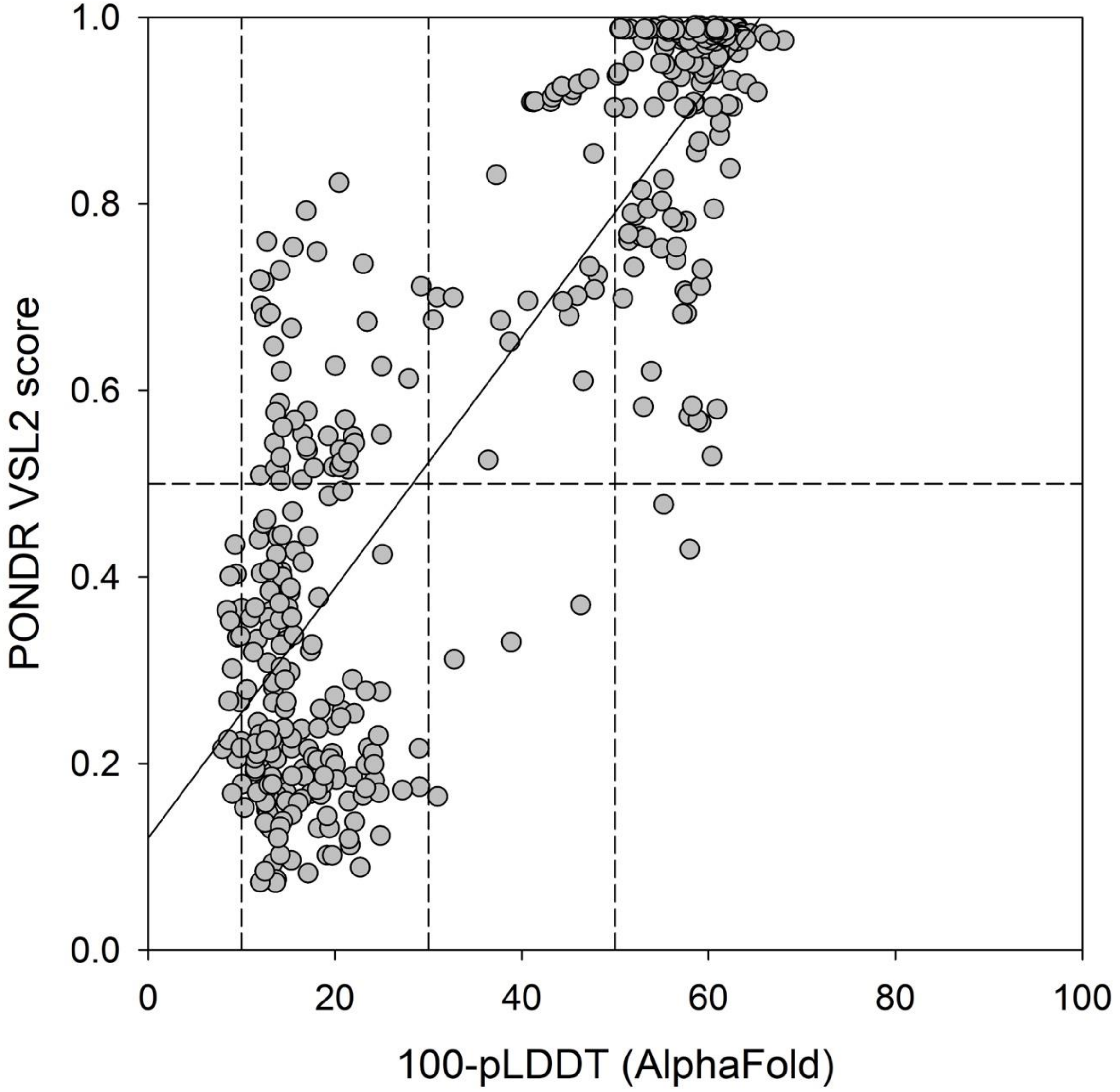
PONDR VSL2 score
100-pLDDT (AlphaFold)
1.0
0.8
0.6
0.4
0.2
0.0
0
20
40
60
80
100

H

Supplementary Figure S2. Functional disorder in heterogeneous nuclear ribonucleoproteins A2/B1. **A.** Amino acid sequence. **B.** RIDAO-generated disorder profile. **C.** D2P2-generated functional disorder profile. **D.** FuzDrop output. **E.** AlphaFold2 structure with FuzDrop annotations. **F.** STRING-generated PPI network with corresponding statistics. **G.** MeDor output. **H.** Plot of the *per* residue disorder score (as provided by PONDR® VLS2) versus (100-pLDDT), where pLDDT is the corresponding AlphaFold2 confidence score.

A

>sp|P22626|ROA2_HUMAN Heterogeneous nuclear ribonucleoproteins A2/B1 OS=Homo sapiens OX=9606 GN=HNRNPA2B1 PE=1 SV=2
MEKTLETVPLERKKREKEQFRKLFIGGLSFETTEESLRNYYEQWGKLTDCVVMRDPASKRSRGFGFVTFSSMAEVDAAMAARPHSIDGRVVEPKRAVAREESGKPGAHVTVKKLFVGG
IKEDTEEHHLRDYFEEYGKIDTIEIITDRQSGKKRGFGFVTFDDHDPVDKIVLQKYHTINGHNAEVRKALSRQEMQEVQSSRSGRGGNFGFGDSRGGGGNFGPGPGSNFRGGSDGYGS
GRGFGDGYNGYGGGPGGGNFGGSPGYGGGRGGYGGGGPGYGNQGGGYGGGYDNYGGGNYGSGNYNDFGNYNQQPSNYGPMKSGNFGGSRNMGGPYGGGNYGPGGSGGSGGYGGRSRY

B

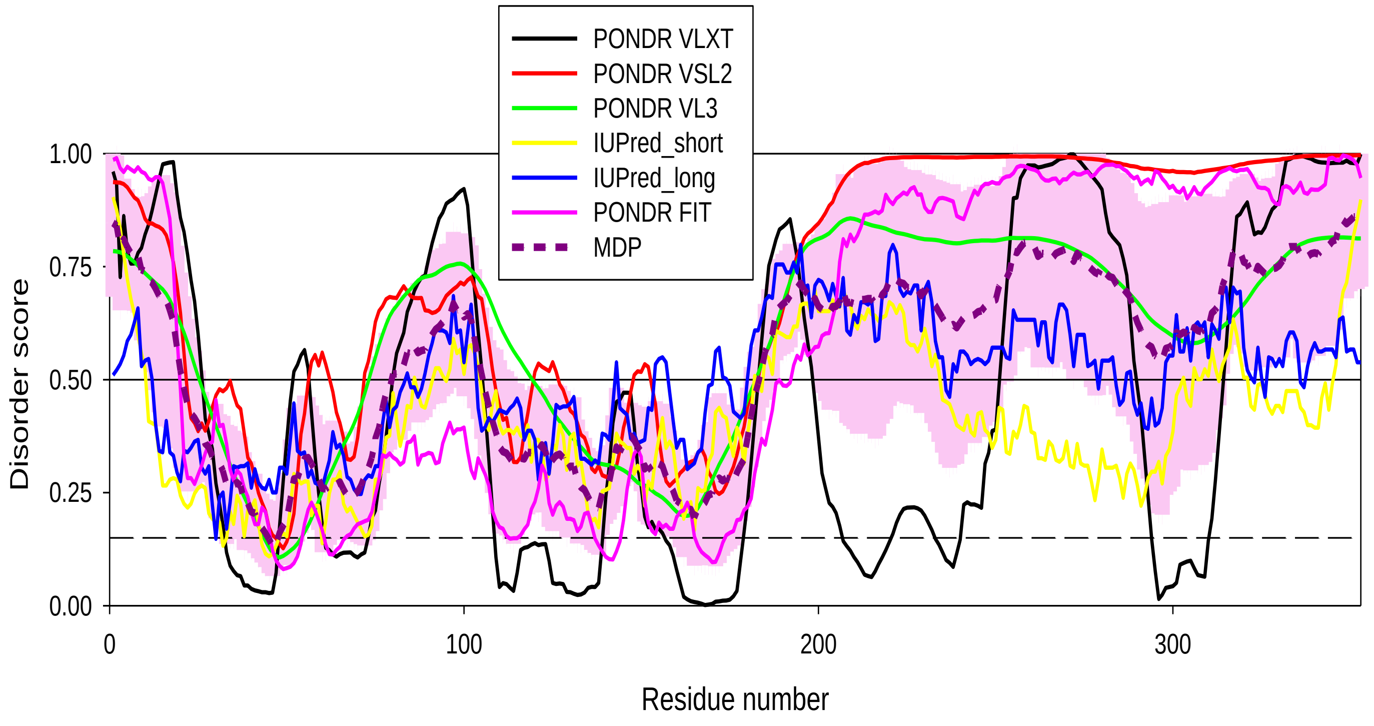

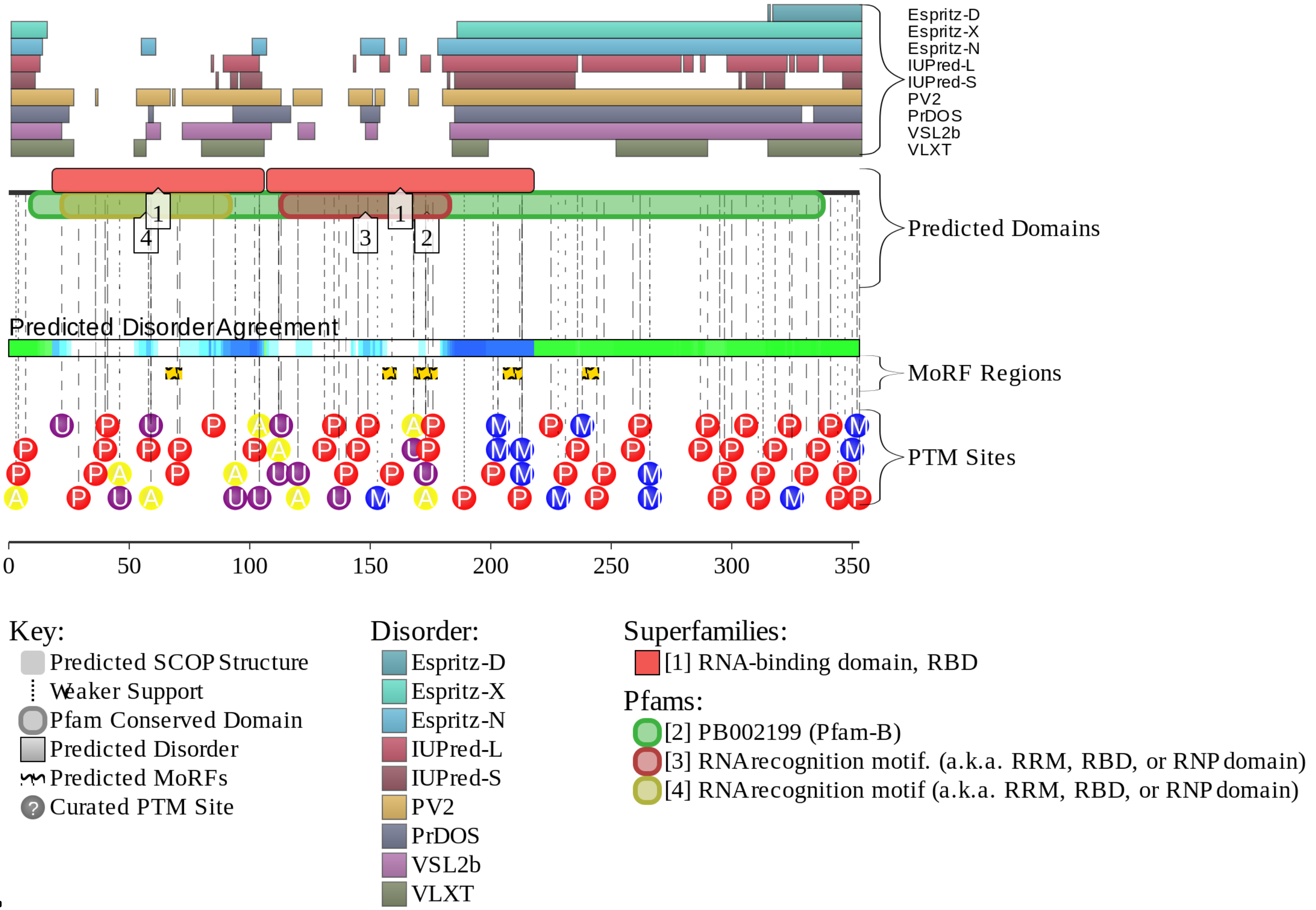

Espritz-D
Espritz-X
Espritz-N
IUPred-L
IUPred-S
PV2
PrDOS
VSL2b
VLXT
Predicted Domains
Predicted Disorder Agreement
MoRF Regions
PTM Sites
0
50
100
150
200
250
300
350
Key:
Predicted SCOP Structure
Weaker Support
Pfam Conserved Domain
Predicted Disorder
Predicted MoRFs
Curated PTM Site
Disorder:
Espritz-D
Espritz-X
Espritz-N
IUPred-L
IUPred-S
PV2
PrDOS
VSL2b
VLXT
Superfamilies:
[1] RNA-binding domain, RBD
Pfams:
[2] PB002199 (Pfam-B)
[3] RNA recognition motif. (a.k.a. RRM, RBD, or RNP domain)
[4] RNA recognition motif (a.k.a. RRM, RBD, or RNP domain)

C

D

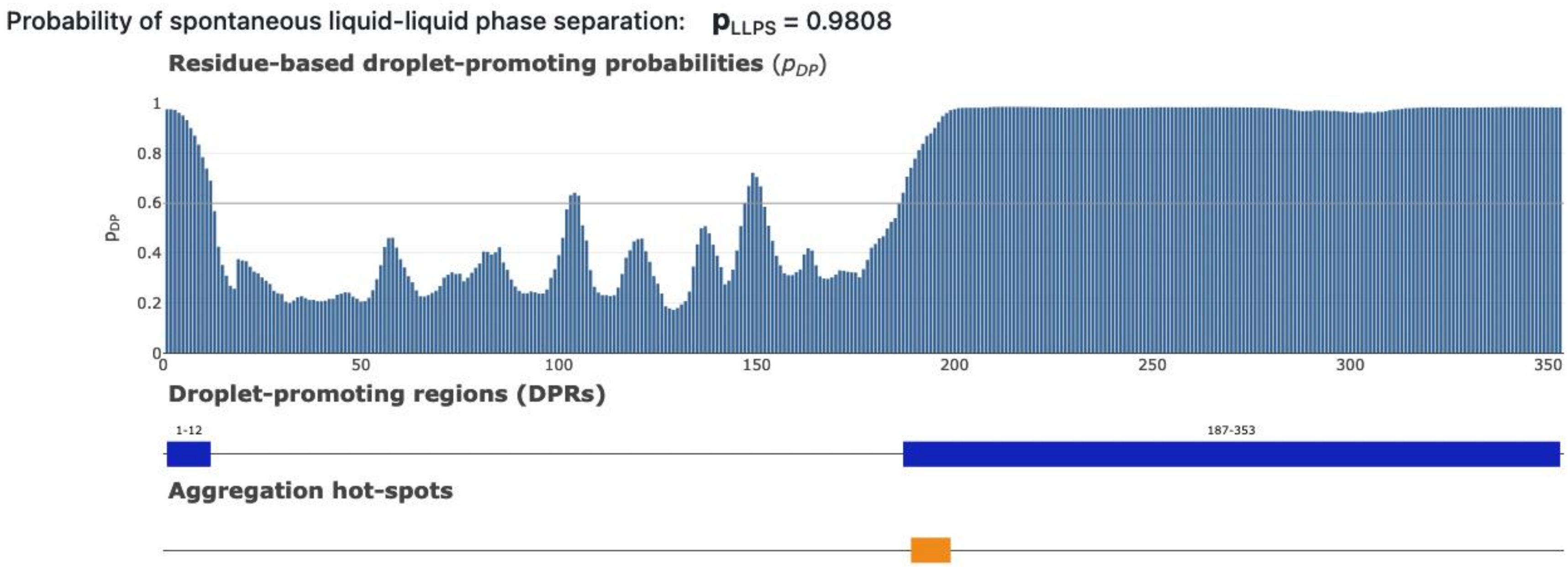
Probability of spontaneous liquid-liquid phase separation: pLLPS = 0.9808
Residue-based droplet-promoting probabilities (pDP)
1
0.8
0.6
0.4
0.2
0
pDP
0
50
100
150
200
250
300
350
Droplet-promoting regions (DPRs)
1-12
187-353
Aggregation hot-spots

E

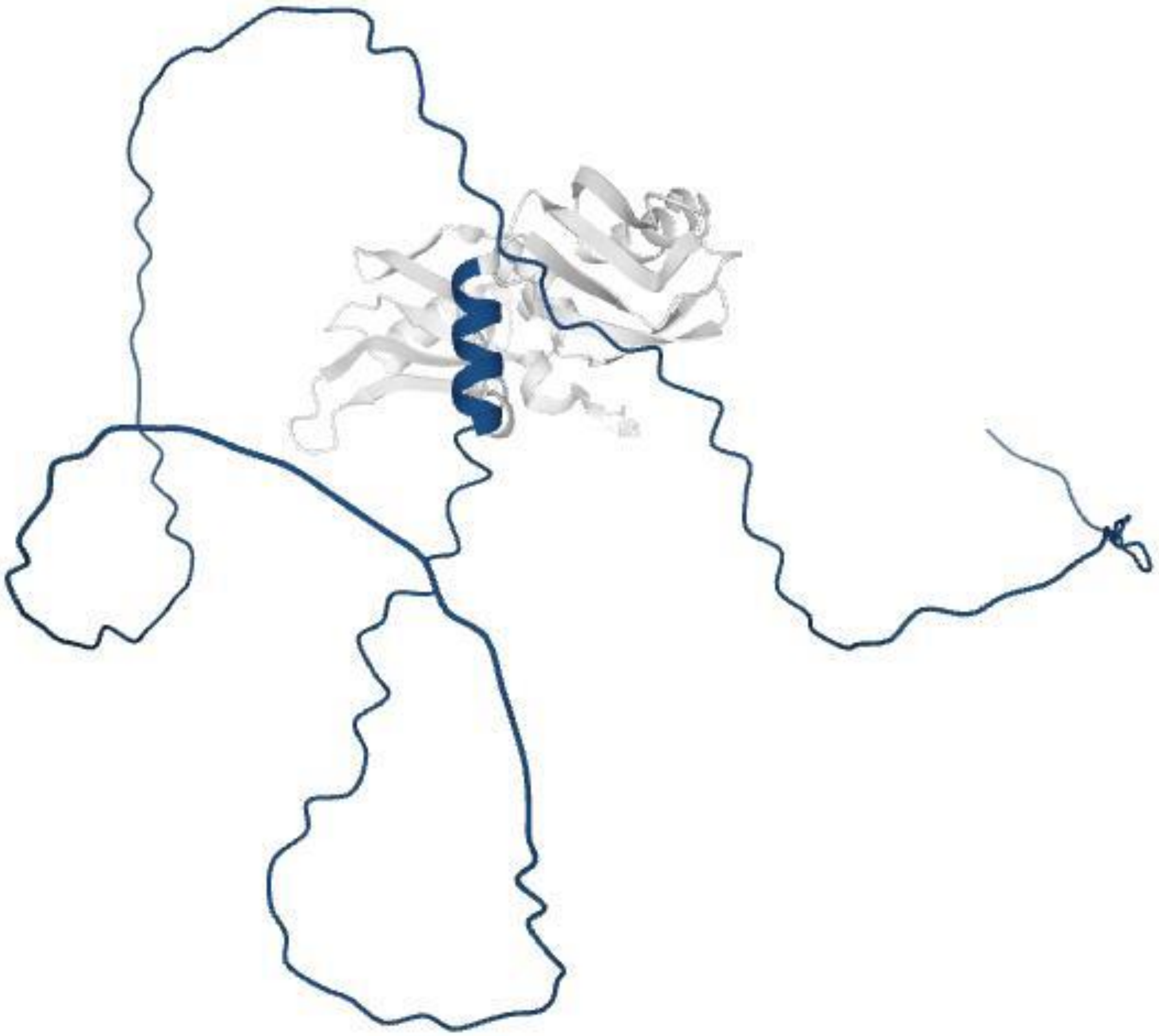

F

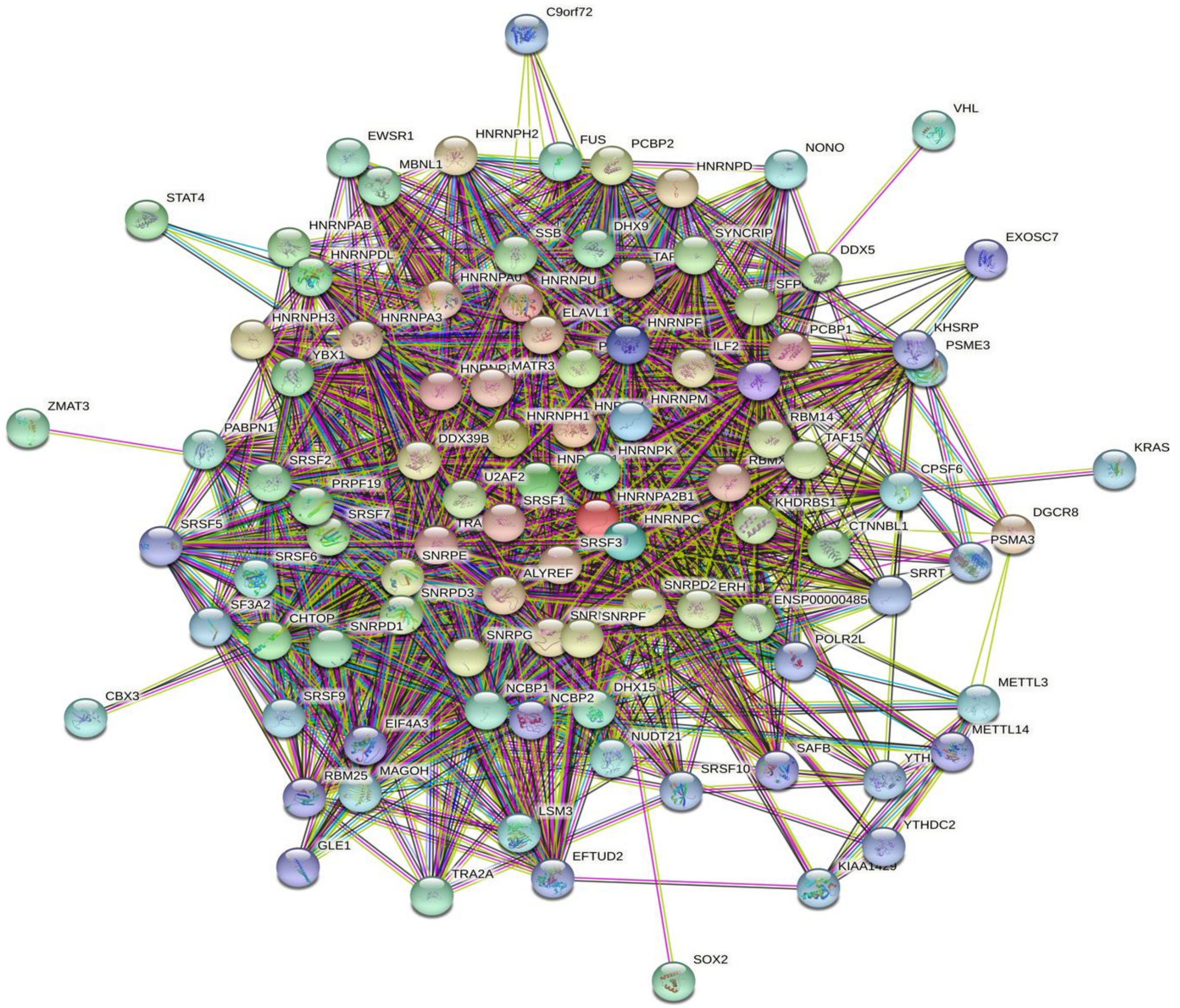

minimum required interaction score: high confidence (0.700)
number of nodes: 97

number of edges: 1861
average node degree: 38.4
avg. local clustering coefficient: 0.762
expected number of edges: 193
PPI enrichment p-value: < 1.0e-16

G

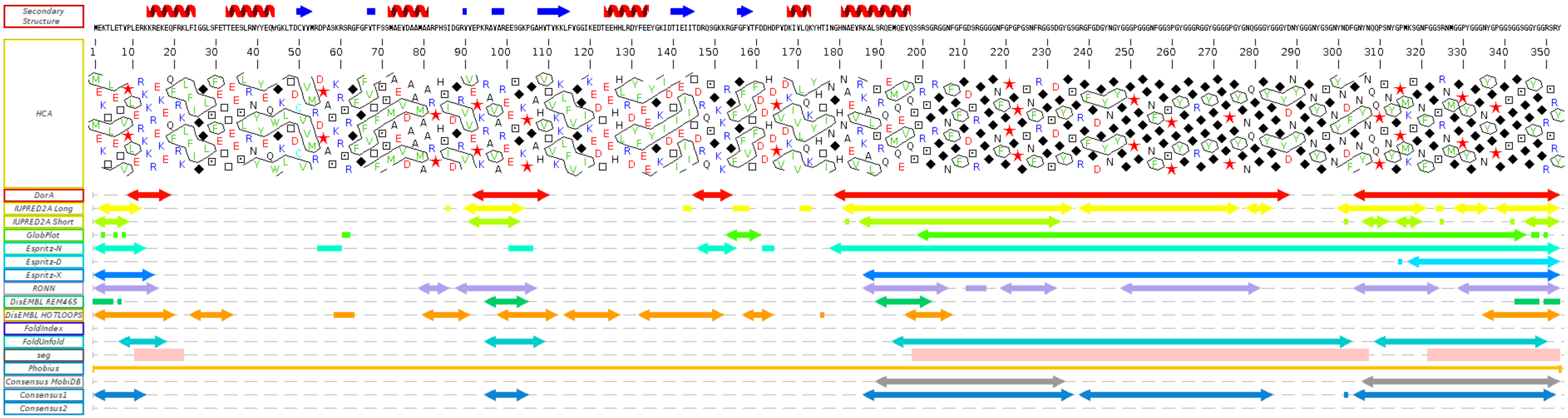
Secondary Structure
HCA
DorA
IUPRED2A Long
IUPRED2A Short
GlobPlot
Espritz-N
Espritz-D
Espritz-X
RONN
DisEMBL REM465
DisEMBL HOTLOOPS
FoldIndex
FoldUnfold
seg
Phobius
Consensus MobiDB
Consensus1
Consensus2
1 10 20 30 40 50 60 70 80 90 100 110 120 130 140 150 160 170 180 190 200 210 220 230 240 250 260 270 280 290 300 310 320 330 340 350

H

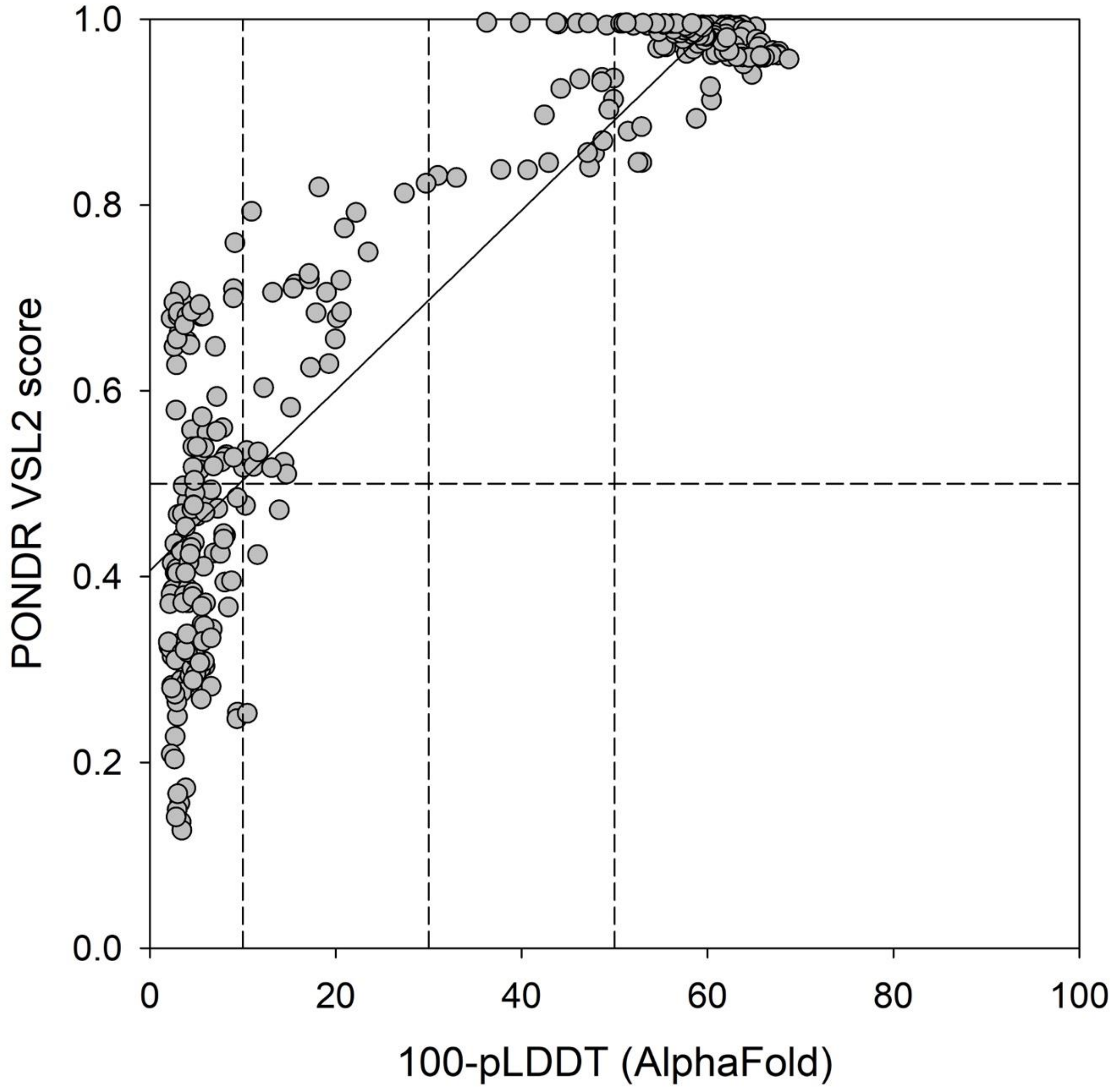
1.0
0.8
0.6
0.4
0.2
0.0
0
20
40
60
80
100
PONDR VSL2 score
100-pLDDT (AlphaFold)

Supplementary Figure S3. Functional disorder in mitochondrial antiviral-signaling protein MAVS. **A.** Amino acid sequence. **B.** RIDAO-generated disorder profile. **C.** D2P2-generated functional disorder profile. **D.** FuzDrop output. **E.** AlphaFold2 structure with FuzDrop annotations. **F.** STRING-generated PPI network with corresponding statistics. **G.** MeDor output. **H.** Plot of the *per* residue disorder score (as provided by PONDR® VLS2) versus (100-pLDDT), where pLDDT is the corresponding AlphaFold2 confidence score.

A

```
>sp|Q7Z434|MAVS_HUMAN Mitochondrial antiviral-signaling protein OS=Homo sapiens OX=9606 GN=MAVS PE=1 SV=2
MPFAEDKTYKYICRNFSNFCNVDVVEILPYLPCLTARDQDRLRATCTLSGNRDTLWHLFNTLQRRPGWVEYFIAALRGCELVDLADEVASVYQSYQPRTSDRPPDPLEPPSLPAERPG
PPTPAAAHSIPYNSCREKEPSYPMPVQETQAPESPGENSEQALQTLSPRAIPRNPDGGPLESSSDLAALSPLTSSGHQEQDTELGSTHTAGATSSLTPSRGPVSPSVSFQPLARSTPR
ASRLPGPTGSVVSTGTSFSSSSPGLASAGAAEGKQGAESDQAEPIICSSGAEAPANSLPSKVPTTLMPVNTVALKVPANPASVSTVPSKLPTSSKPPGAVPSNALTNPAPSKLPINST
RAGMVPSKVPTSMVLTKVSASTVPTDGSSRNEETPAAPTPAGATGGSSAWLDSSSENRGLGSELSKPGVLASQVDSPFSGCFEDLAISASTSLGMGPCHGPEENEYKSEGTFGIHVAE
NPSIQLLEGNPGPPADPDGGPRPQADRKFQEREVPCHRPSPGALWLQVAVTGVLVVTLLVVLYRRRLH
```

B

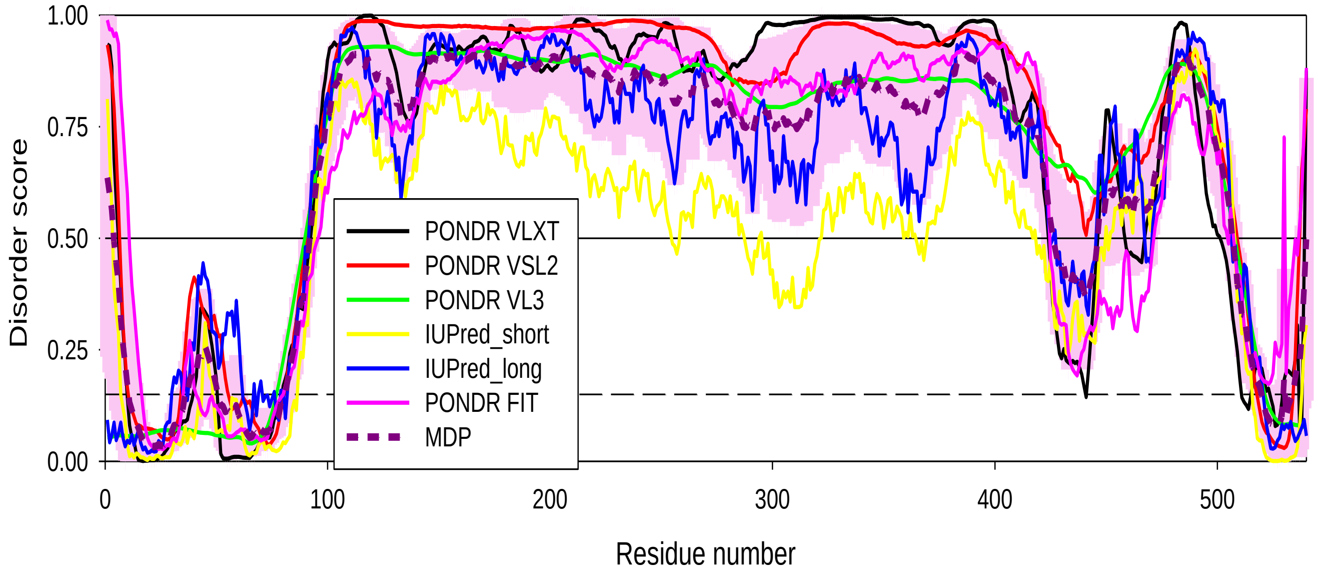

C

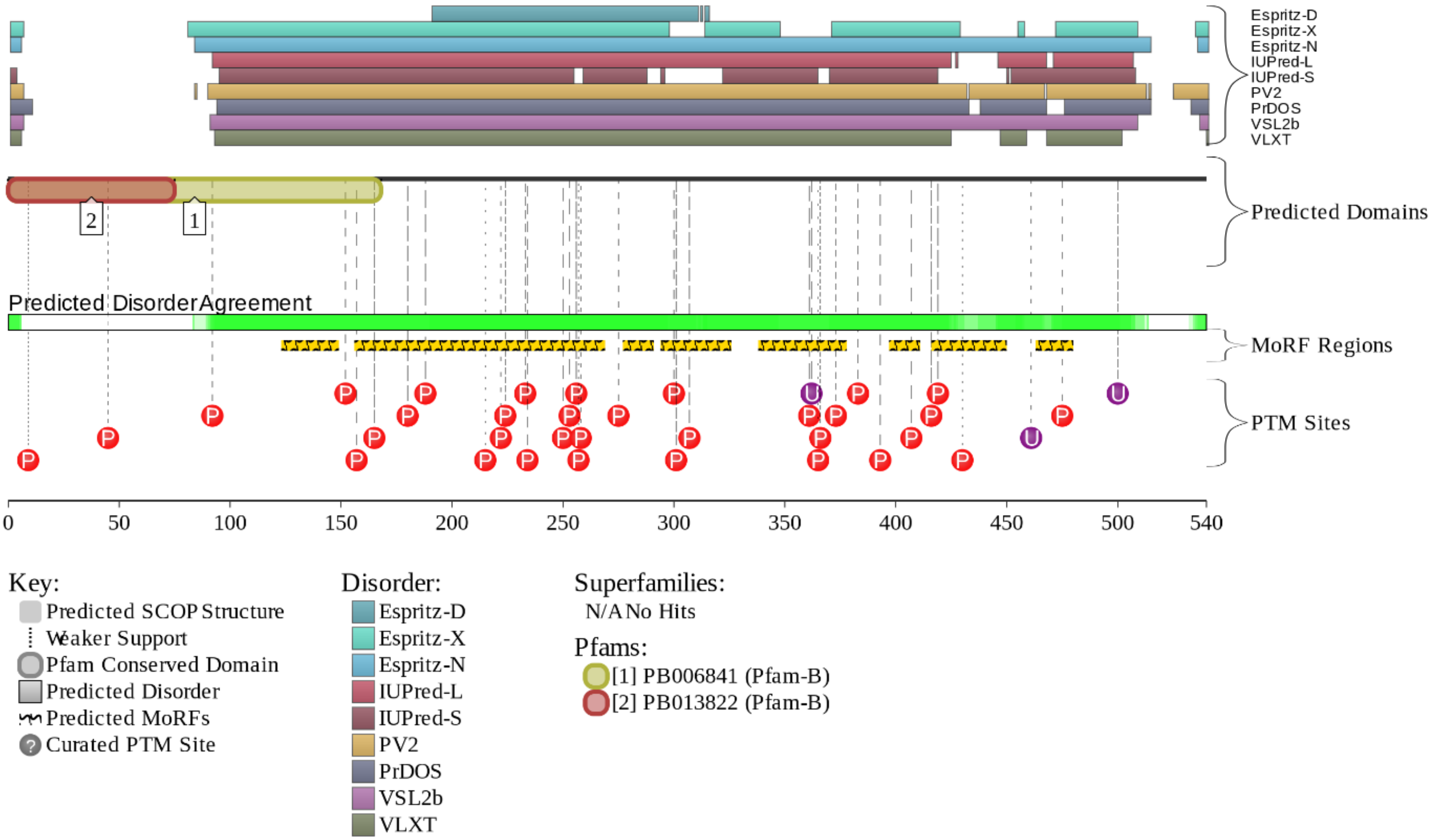

Espritz-D
Espritz-X
Espritz-N
IUPred-L
IUPred-S
PV2
PrDOS
VSL2b
VLXT
Predicted Domains
2
1
Predicted Disorder Agreement
MoRF Regions
PTM Sites
0
50
100
150
200
250
300
350
400
450
500
540
Key:
Predicted SCOP Structure
Weaker Support
Pfam Conserved Domain
Predicted Disorder
Predicted MoRFs
Curated PTM Site
Disorder:
Espritz-D
Espritz-X
Espritz-N
IUPred-L
IUPred-S
PV2
PrDOS
VSL2b
VLXT
Superfamilies:
N/A No Hits
Pfams:
[1] PB006841 (Pfam-B)
[2] PB013822 (Pfam-B)

D

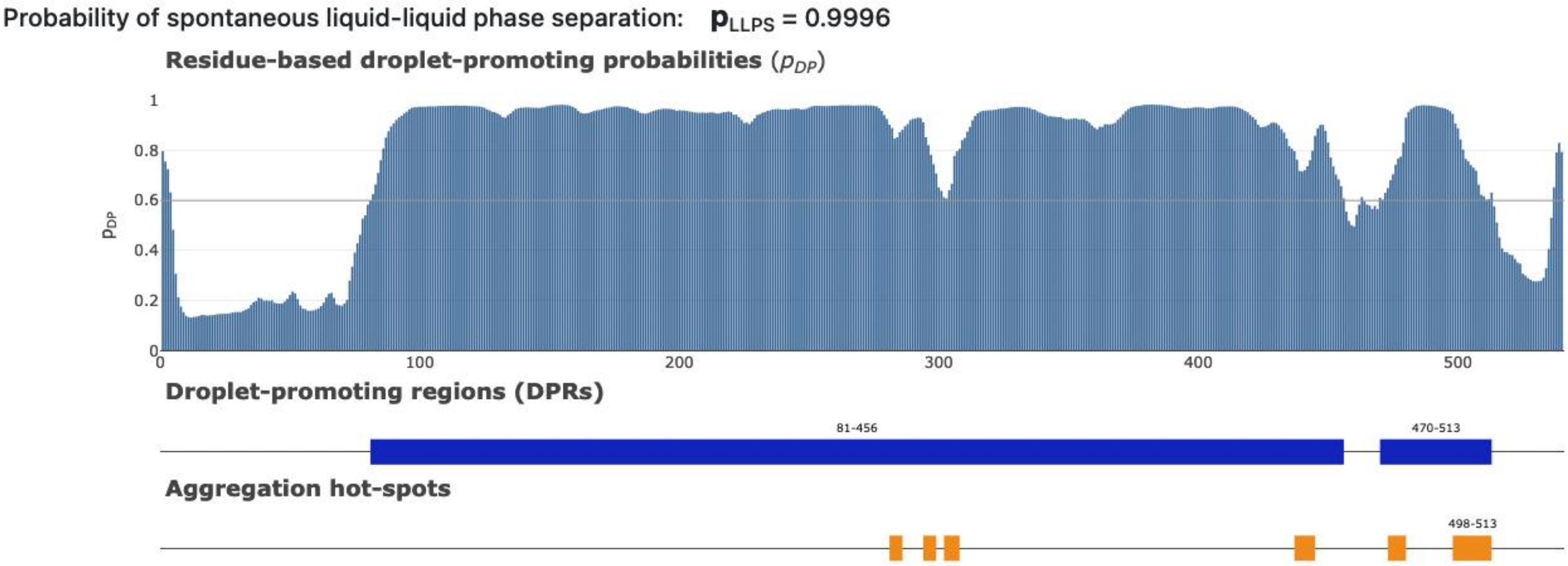

Probability of spontaneous liquid-liquid phase separation: p_{LLPS} = 0.9996
Residue-based droplet-promoting probabilities (p_{DP})
1
0.8
0.6
0.4
0.2
0
pDP
0
100
200
300
400
500
Droplet-promoting regions (DPRs)
81-456
470-513
Aggregation hot-spots
498-513

E

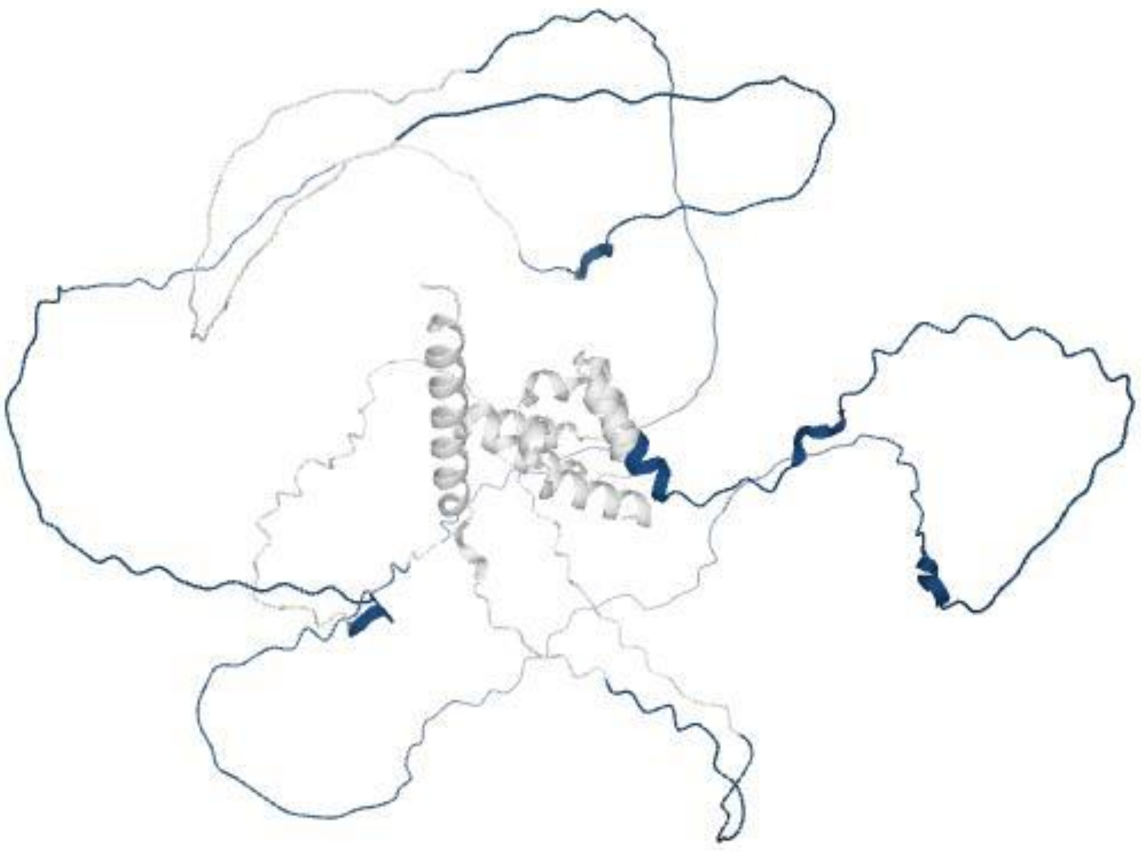

F

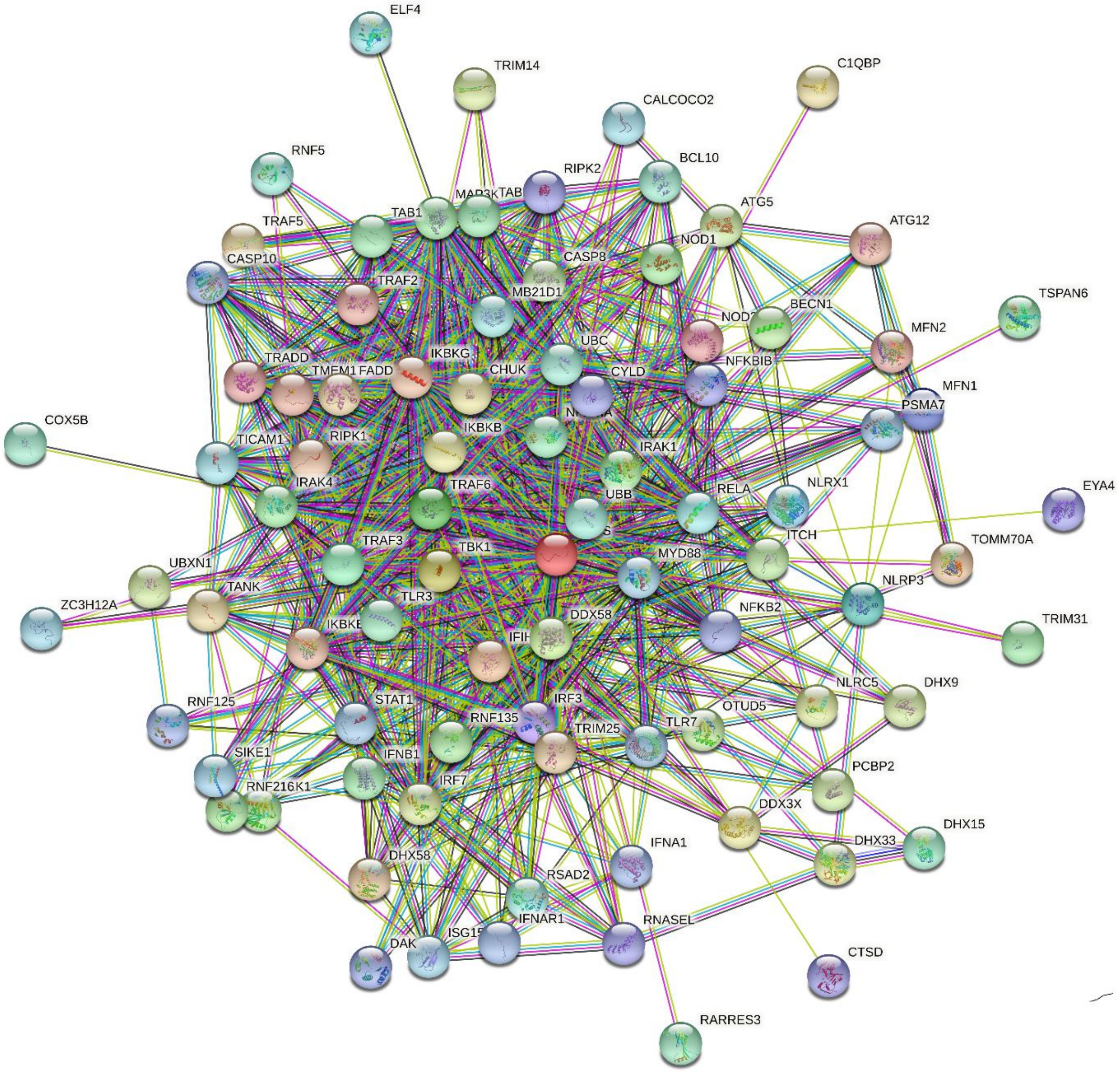
ELF4
TRIM14
C1QBP
CALCOCO2
RNF5
RIPK2
BCL10
TAB1
ATG5
TRAF5
ATG12
NOD1
CASP8
CASP10
TRAF2
MB21D1
TSPAN6
BECN1
MFN2
UBC
TRADD
FADD
IKBKG
CHUK
CYLD
NFKBIB
MFN1
PSMA7
COX5B
TICAM1
RIPK1
IKBKB
IRAK1
IRAK4
TRAF6
UBB
RELA
NLRX1
EYA4
ITCH
TOMM70A
TRAF3
TBK1
MYD88
UBXN1
NLRP3
TANK
ZC3H12A
TLR3
DDX58
NFKB2
TRIM31
NLRC5
DHX9
RNF125
STAT1
IRF3
RNF135
OTUD5
TRIM25
TLR7
SIKE1
IFNB1
PCBP2
IRF7
DDX3X
DHX15
DHX33
DHX58
IFNA1
RSAD2
IFNAR1
RNASEL
DAK
CTSD
RARRES3

minimum required interaction score: high confidence (0.700)
number of nodes: 87
number of edges: 786
average node degree: 18.1
avg. local clustering coefficient: 0.7
expected number of edges: 151
PPI enrichment p-value: < 1.0e-16

G

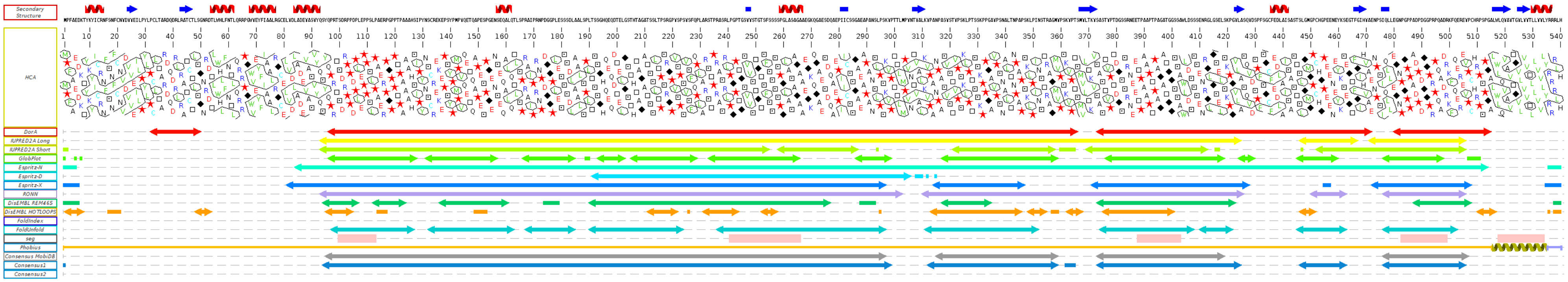

Secondary Structure
HCA
DorA
IUPRED2A Long
IUPRED2A Short
GlobPlot
Espritz-N
Espritz-D
Espritz-X
RONN
DisEMBL REM465
DisEMBL HOTLOOPS
FoldIndex
FoldUnfold
seg
Phobius
Consensus MobiDB
Consensus1
Consensus2

H

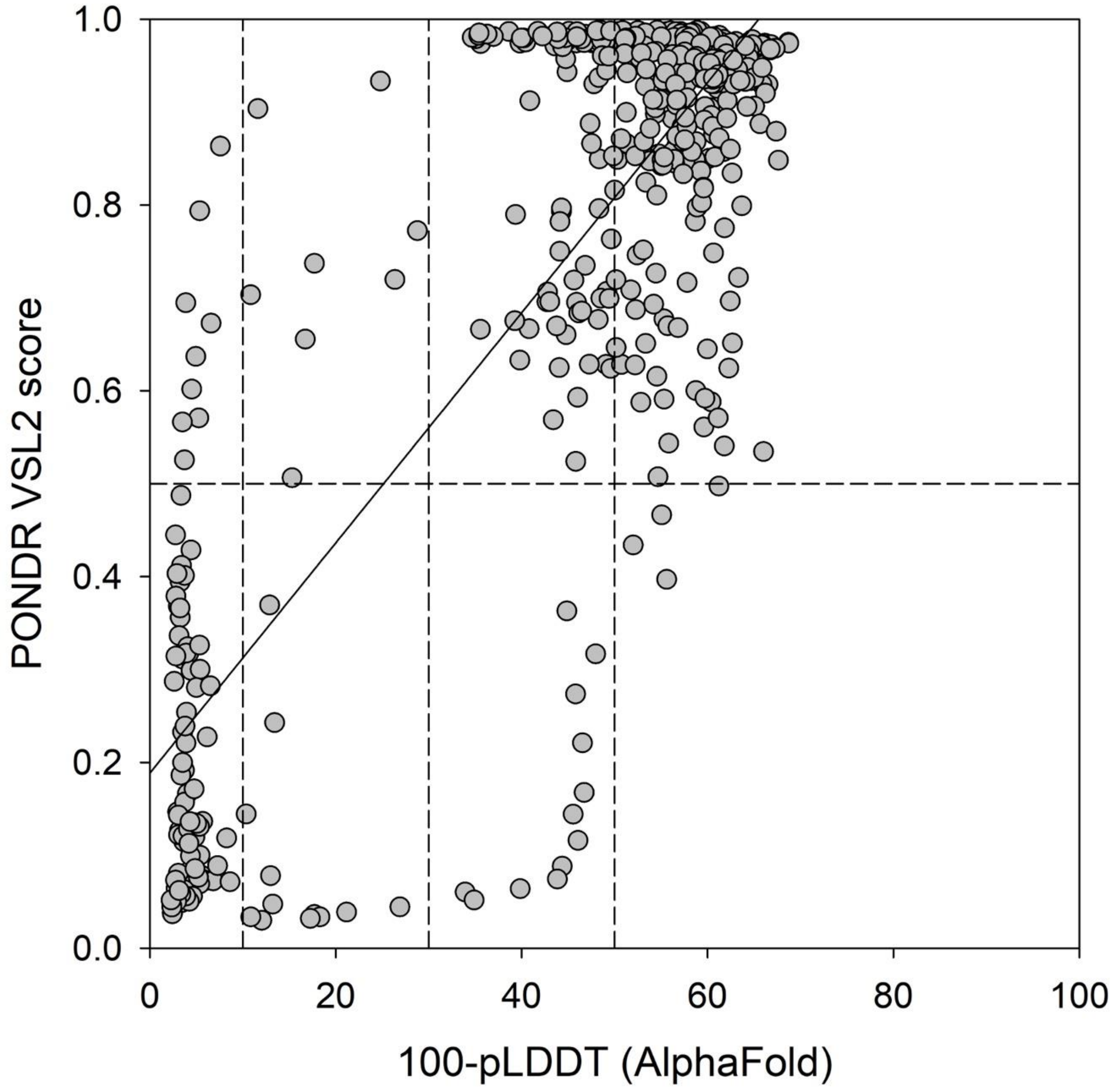

1.0
0.8
0.6
0.4
0.2
0.0
PONDR VSL2 score
0
20
40
60
80
100
100-pLDDT (AlphaFold)

Supplementary Figure S4. Functional disorder in interferon-induced helicase C domain-containing protein 1, MDA5. **A.** Amino acid sequence. **B.** RIDAO-generated disorder profile. **C.** D2P2-generated functional disorder profile. **D.** FuzDrop output. **E.** AlphaFold2 structure with FuzDrop annotations. **F.** STRING-generated PPI network with corresponding statistics. **G.** MeDor output. **H.** Plot of the *per* residue disorder score (as provided by PONDR® VLS2) versus (100-pLDDT), where pLDDT is the corresponding AlphaFold2 confidence score.

A

>sp|Q9BYX4|IFIH1_HUMAN Interferon-induced helicase C domain-containing protein 1 OS=Homo sapiens OX=9606 GN=IFIH1 PE=1 SV=3
MSNGYSTDENFRYLISCFRARVKMYIQVEPVLDYLTFLPAEVKEQIQRTVATSGNMQAVELLLSTLEKGVWHLGWTREFVEALRRTGSPLAARYMNPELTDLPSPSFENAHDEYLQLL
NLLQPTLVDKLLVRDVLDKCMEEELLTIEDRNRIAAAENNGNESGVRELLKRIVQKENWFSAFLNVLRQTGNNELVQELTGSDCSESNAEIENLSQVDGPQVEEQLLSTTVQPNLEKE
VWGMENNSSESSFADSSVVSESDTSLAEGSVSCLDESLGHNSNMGSDSGTMGSDSDEENVAARASPEPELQLRPYQMEVAQPALEGKNIIICLPTGSGKTRVAVYIAKDHLDKKKKAS
EPGKVIVLVNKVLLVEQLFRKEFQPFLKKWYRVIGLSGDTQLKISFPEVVKSCDIIISTAQILENSLLNLENGEDAGVQLSDFSLIIIDECHHTNKEAVYNNIMRHYLMQKLKNNRLK
KENKPVIPLPQILGLTASPGVGGATKQAKAEEHILKLCANLDAFTIKTVKENLDQLKNQIQEPCKKFAIADATREDPFKEKLLEIMTRIQTYCQMSPMSDFGTQPYEQWAIQMEKKAA
KEGNRKERVCAEHLRKYNEALQINDTIRMIDAYTHLETFYNEEKDKKFAVIEDDSDEGGDDEYCDGDEDEDDLKKPLKLDETDRFLMTLFFENNKMLKRLAENPEYENEKLTKLRNTI
MEQYTRTEESARGIIFTKTRQSAYALSQWITENEKFAEVGVKAHHLIGAGHSSEFKPMTQNEQKEVISKFRTGKINLLIATTVAEEGLDIKECNIVIRYGLVTNEIAMVQARGRARAD
ESTYVLVAHSGSGVIEHETVNDFREKMMYKAIHCVQNMKPEEYAHKILELQMQSIMEKKMKTKRNIAKHYKNNPSLITFLCKNCSVLACSGEDIHVIEKMHHVNMTPEFKELYIVREN
KALQKKCADYQINGEIICKCGQAWGTMMVHKGLDLPCLKIRNFVVVFKNNSTKKQYKKWVELPITFPNLDYSECCLFSDED

B

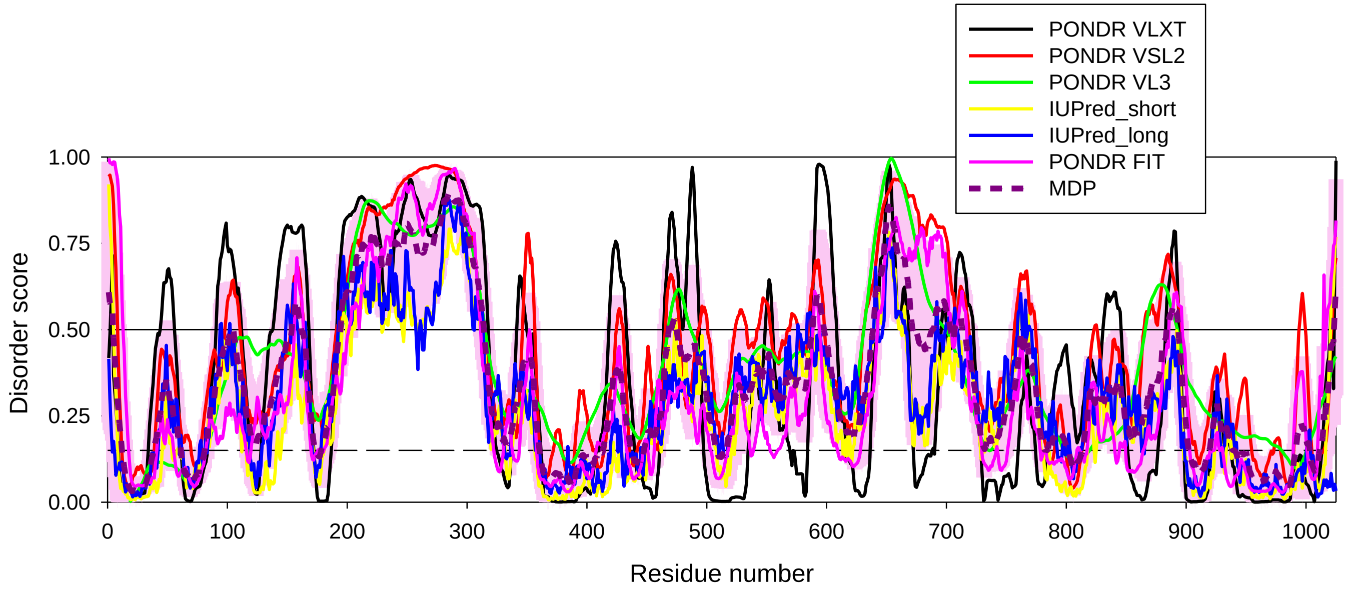

C

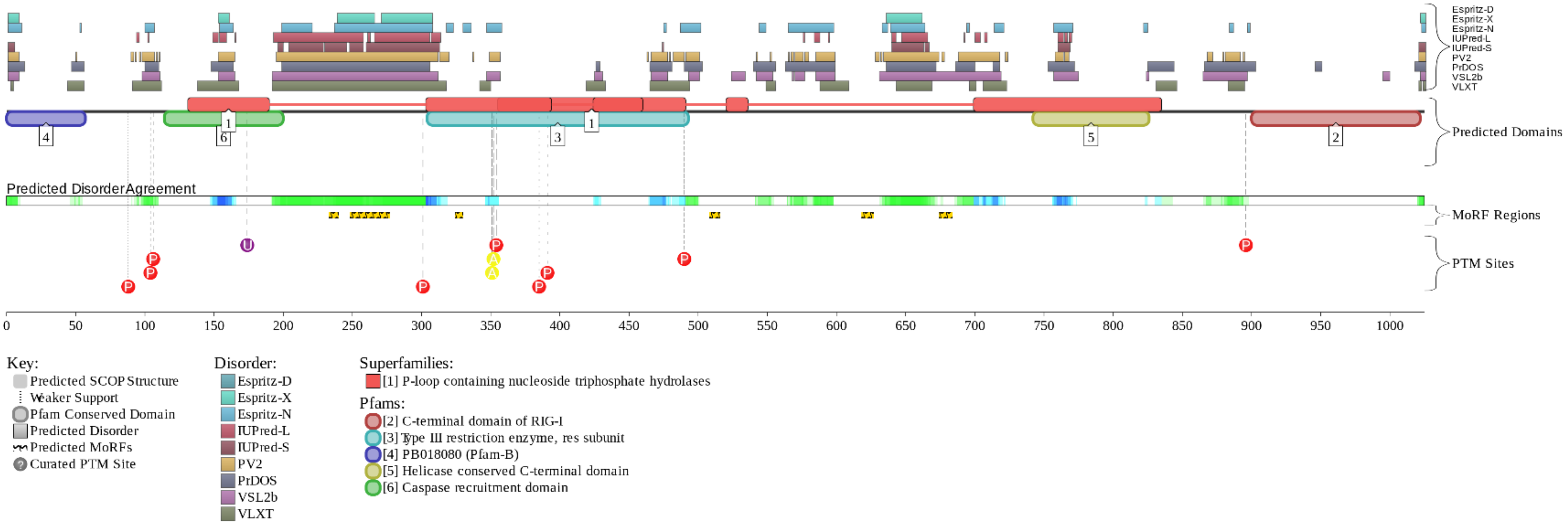

Esspritz-D
Esspritz-X
Esspritz-N
IUPred-L
IUPred-S
PV2
PrDOS
VSL2b
VLXT
Predicted Domains
Predicted DisorderAgreement
MoRF Regions
PTM Sites
0 50 100 150 200 250 300 350 400 450 500 550 600 650 700 750 800 850 900 950 1000
Key:
Predicted SCOP Structure
Weaker Support
Pfam Conserved Domain
Predicted Disorder
Predicted MoRFs
Curated PTM Site
Disorder:
Espritz-D
Espritz-X
Espritz-N
IUPred-L
IUPred-S
PV2
PrDOS
VSL2b
VLXT
Superfamilies:
[1] P-loop containing nucleoside triphosphate hydrolases
Pfams:
[2] C-terminal domain of RIG-I
[3] Type III restriction enzyme, res subunit
[4] PB018080 (Pfam-B)
[5] Helicase conserved C-terminal domain
[6] Caspase recruitment domain

D

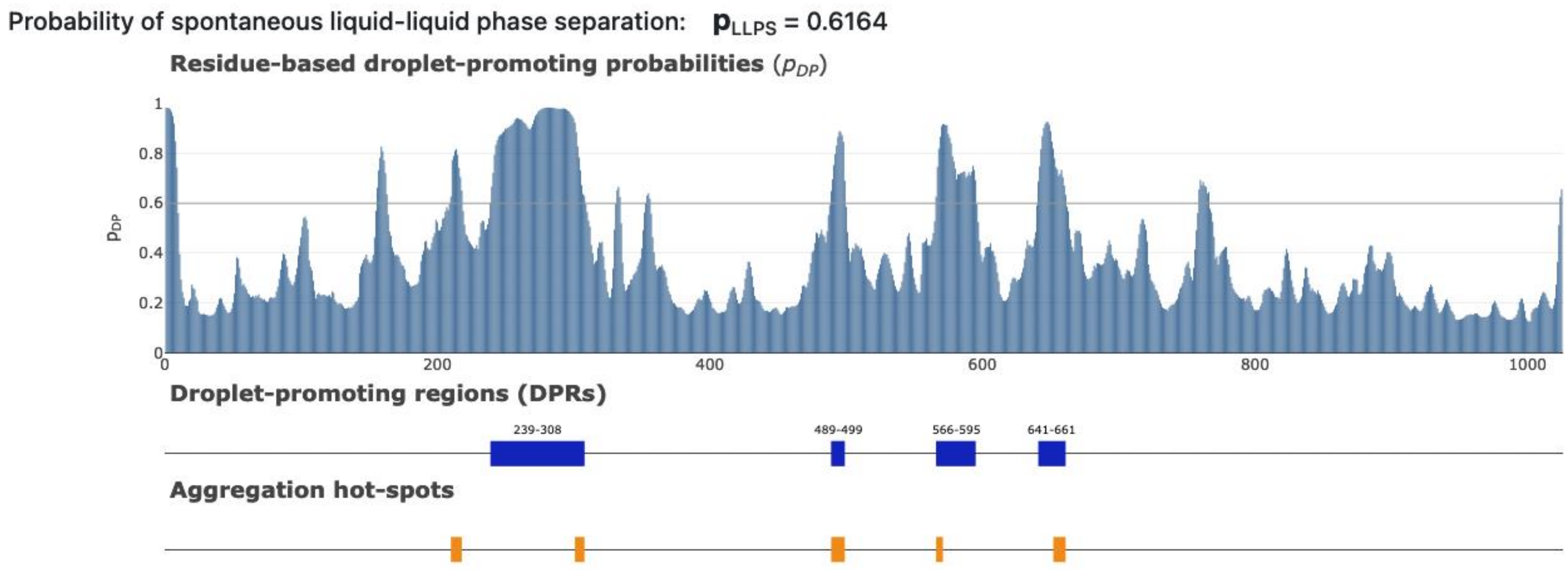

E

There is no AlphaFold2-generated structure for this protein at FuzDrop

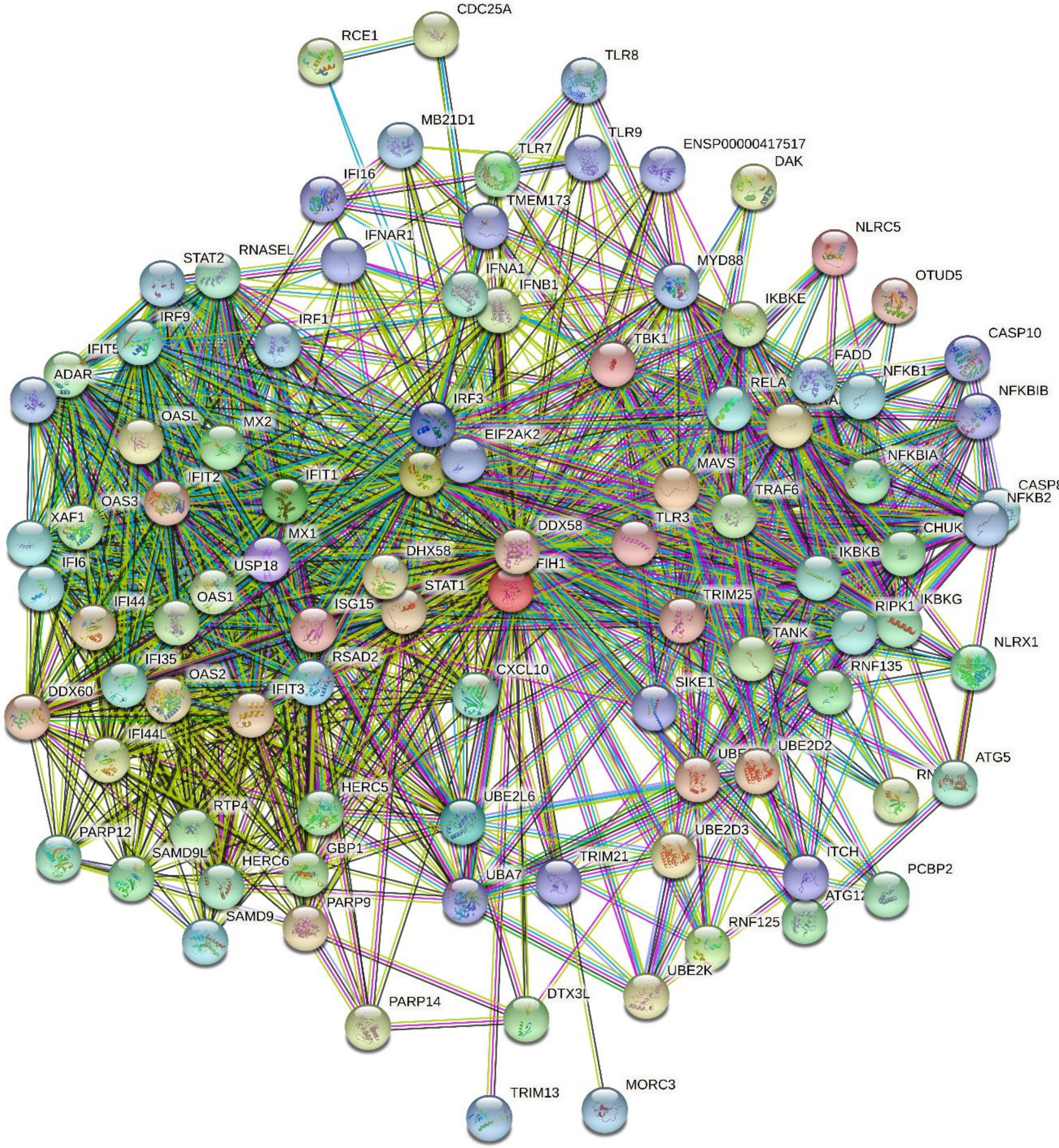

CDC25A
RCE1
TLR8
MB21D1
TLR7
TLR9
ENSP00000417517
DAK
IFI16
TMEM173
NLRC5
IFNAR1
RNASEL
STAT2
MYD88
OTUD5
IFNA1
IFNB1
IKBKE
IRF9
IRF1
TBK1
CASP10
IFIT5
FADD
NFKB1
ADAR
RELA
NFKBIB
IRF3
OASL
MX2
EIF2AK2
NFKBIA
IFIT2
IFIT1
MAVS
CASP8
NFKB2
OAS3
TRAF6
XAF1
TLR3
DDX58
CHUK
MX1
DHX58
IKBKB
USP18
FIH1
IFI6
STAT1
TRIM25
IKBKG
RIPK1
ISG15
IFI44
OAS1
TANK
NLRX1
RSAD2
IFI35
RNF135
CXCL10
OAS2
SIKE1
DDX60
IFIT3
IFI44L
UBE2D2
ATG5
HERC5
UBE2L6
RTP4
PARP12
UBE2D3
ITCH
SAMD9L
HERC6
GBP1
TRIM21
PCBP2
UBA7
RNF125
SAMD9
PARP9
UBE2K
PARP14
DTX3L
TRIM13
MORC3

F

minimum required interaction score: high confidence (0.700)
number of nodes: 96
number of edges: 1162
average node degree: 24.2
avg. local clustering coefficient: 0.751
expected number of edges: 146
PPI enrichment p-value: < 1.0e-16

G

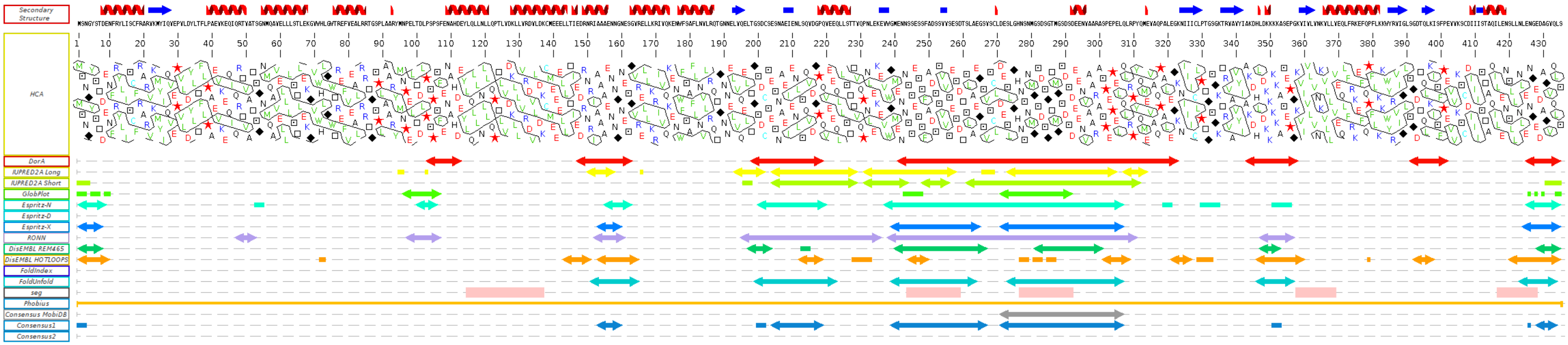
Secondary Structure
HCA
DorA
IUPRED2A Long
IUPRED2A Short
GlobPlot
Espritz-N
Espritz-D
Espritz-X
RONN
DisEMBL REM465
DisEMBL HOTLOOPS
FoldIndex
FoldUnfold
seg
Phobius
Consensus MobiDB
Consensus1
Consensus2

H

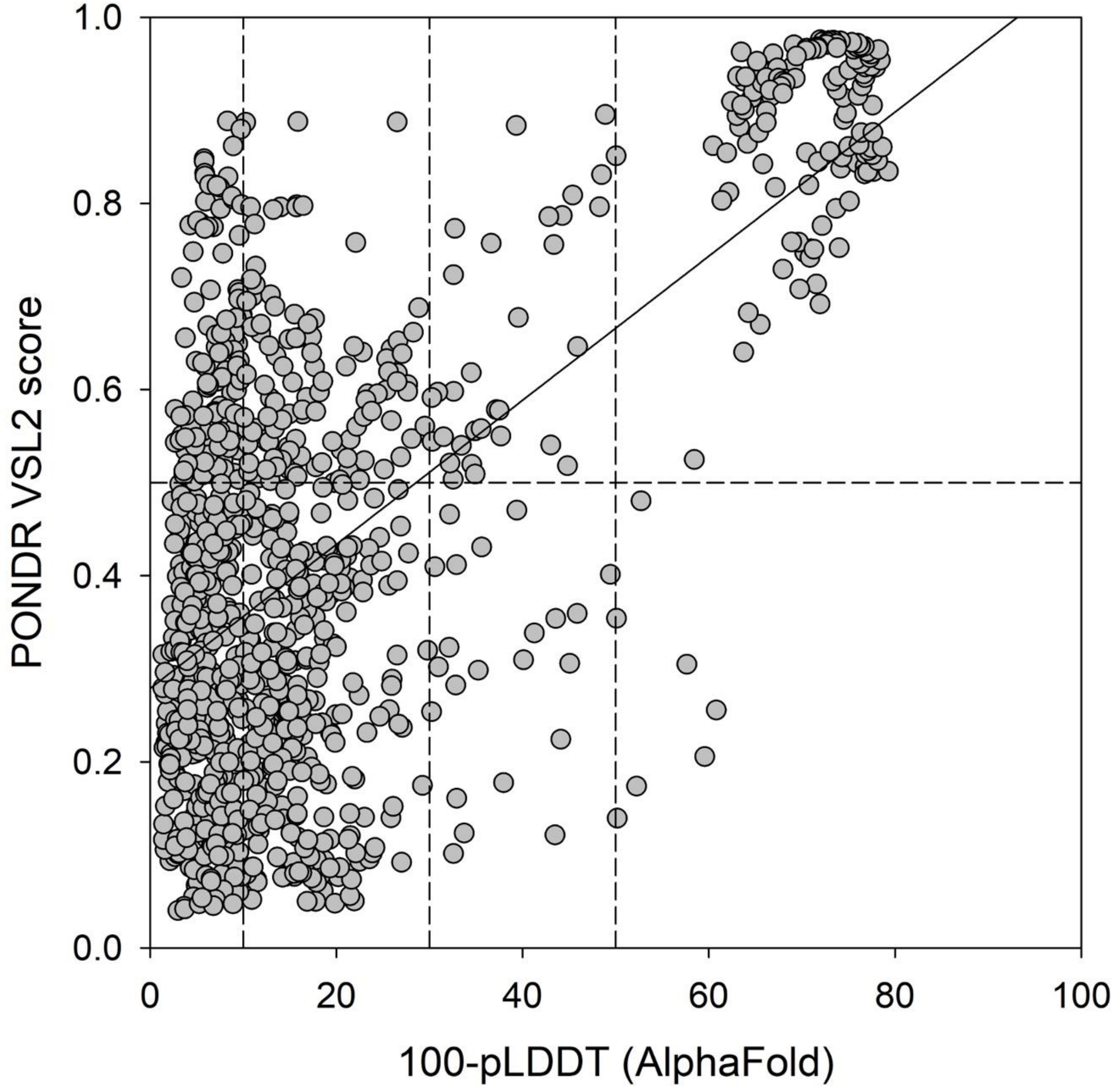

PONDR VSL2 score
0.0
0.2
0.4
0.6
0.8
1.0
0
20
40
60
80
100
100-pLDDT (AlphaFold)

Supplementary Figure S5. Functional disorder in Ras GTPase-activating protein-binding protein 1, G3BP1. **A.** Amino acid sequence. **B.** RIDAO-generated disorder profile. **C.** D2P2-generated functional disorder profile. **D.** FuzDrop output. **E.** AlphaFold2 structure with FuzDrop annotations. **F.** STRING-generated PPI network with corresponding statistics. **G.** MeDor output. **H.** Plot of the *per* residue disorder score (as provided by PONDR® VLS2) versus (100-pLDDT), where pLDDT is the corresponding AlphaFold2 confidence score.

A

>sp|Q13283|G3BP1_HUMAN Ras GTPase-activating protein-binding protein 1 OS=Homo sapiens OX=9606 GN=G3BP1 PE=1 SV=1
MVMEKPSPLLVGREFVRQYYTLLNQAPDMLHRFYGKNSSYVHGGLDSNGKPADAVYGQKEIHRKVMSQNFTNCHTKIRHVDAHATLNDGVVVQVMGLLSNNNQALRRFMQTFVLAPEG
SVANKFYVHNDIFRYQDEVFGGFVTEPQEESEEEVEEPEERQQTPEVVPDDSGTFYDQAVVSNDMEEHLEEPVAEPEPDPEPEPEQEPVSEIQEEKPEPVLEETAPEDAQKSSSPAPA
DIAQTVQEDLRTFSWASVTSKNLPPSGAVPVTGIPPHVVKVPASQPRPESKPESQIPPQRPQRDQRVREQRINIPPQRGPRPIREAGEQGDIEPRRMVRHPDSHQLFIGNLPHEVDKS
ELKDFFQSYGNVVELRINSGGKLPNFGFVVFDDSEPVQKVLSNRPIMFRGEVRLNVEEKKTRAAREGDRRDNRLRGPGGPRGGLGGGMRGPPRGGMVQKPGFGVGRGLAPRQ

B

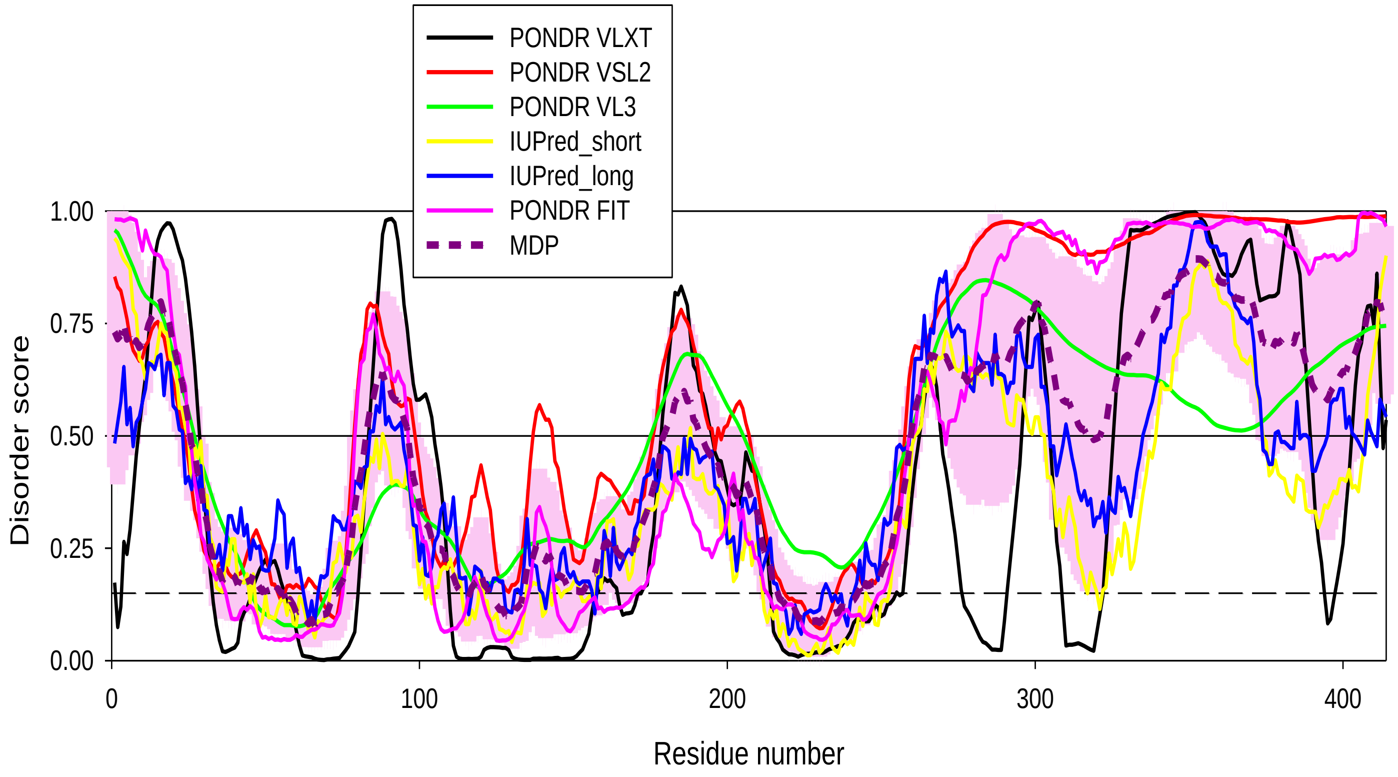

C

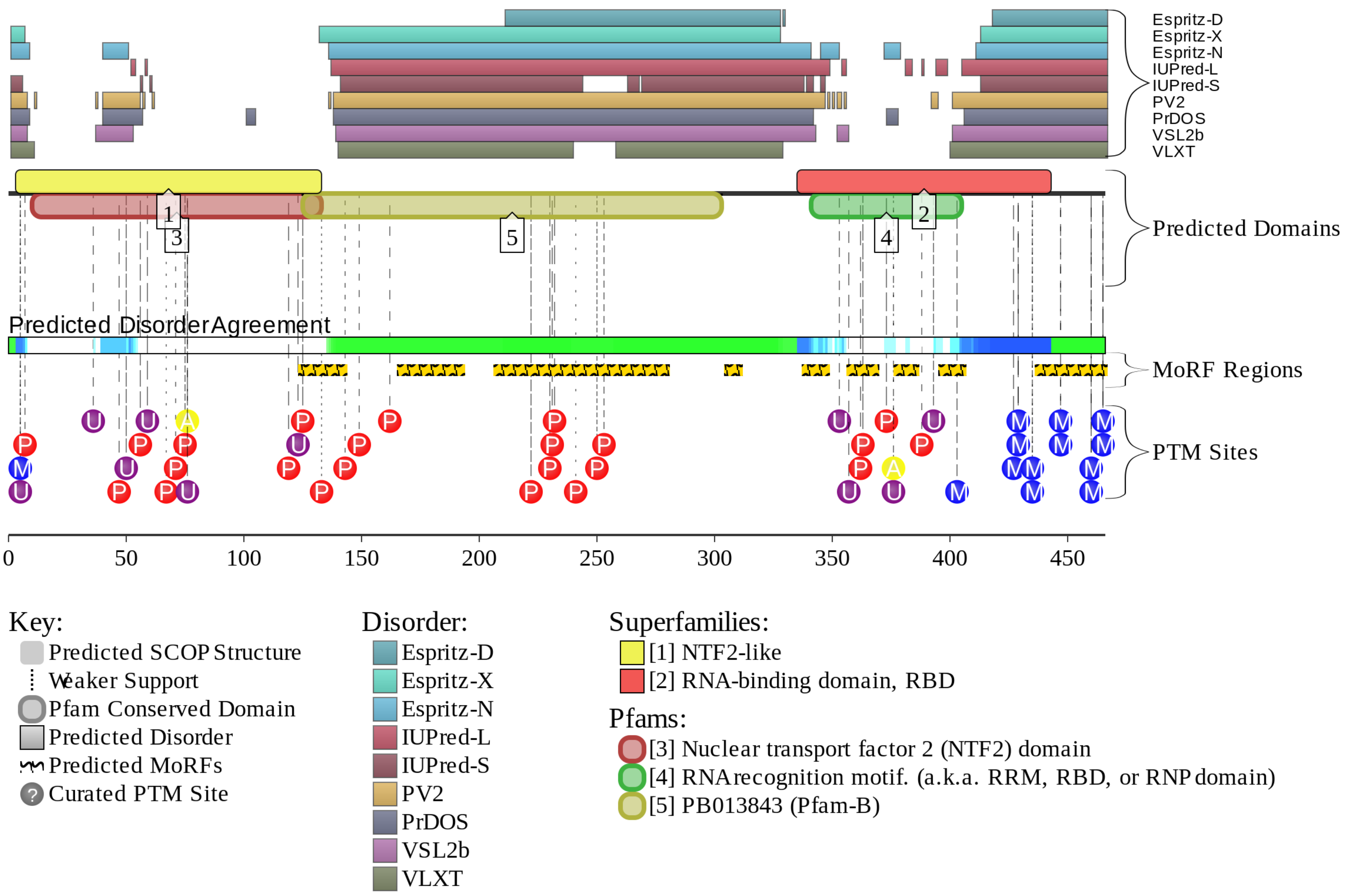

Espritz-D
Espritz-X
Espritz-N
IUPred-L
IUPred-S
PV2
PrDOS
VSL2b
VLXT
Predicted Domains
Predicted Disorder Agreement
MoRF Regions
PTM Sites
0
50
100
150
200
250
300
350
400
450
Key:
Predicted SCOP Structure
Weaker Support
Pfam Conserved Domain
Predicted Disorder
Predicted MoRFs
Curated PTM Site
Disorder:
Espritz-D
Espritz-X
Espritz-N
IUPred-L
IUPred-S
PV2
PrDOS
VSL2b
VLXT
Superfamilies:
[1] NTF2-like
[2] RNA-binding domain, RBD
Pfams:
[3] Nuclear transport factor 2 (NTF2) domain
[4] RNA recognition motif. (a.k.a. RRM, RBD, or RNP domain)
[5] PB013843 (Pfam-B)

D

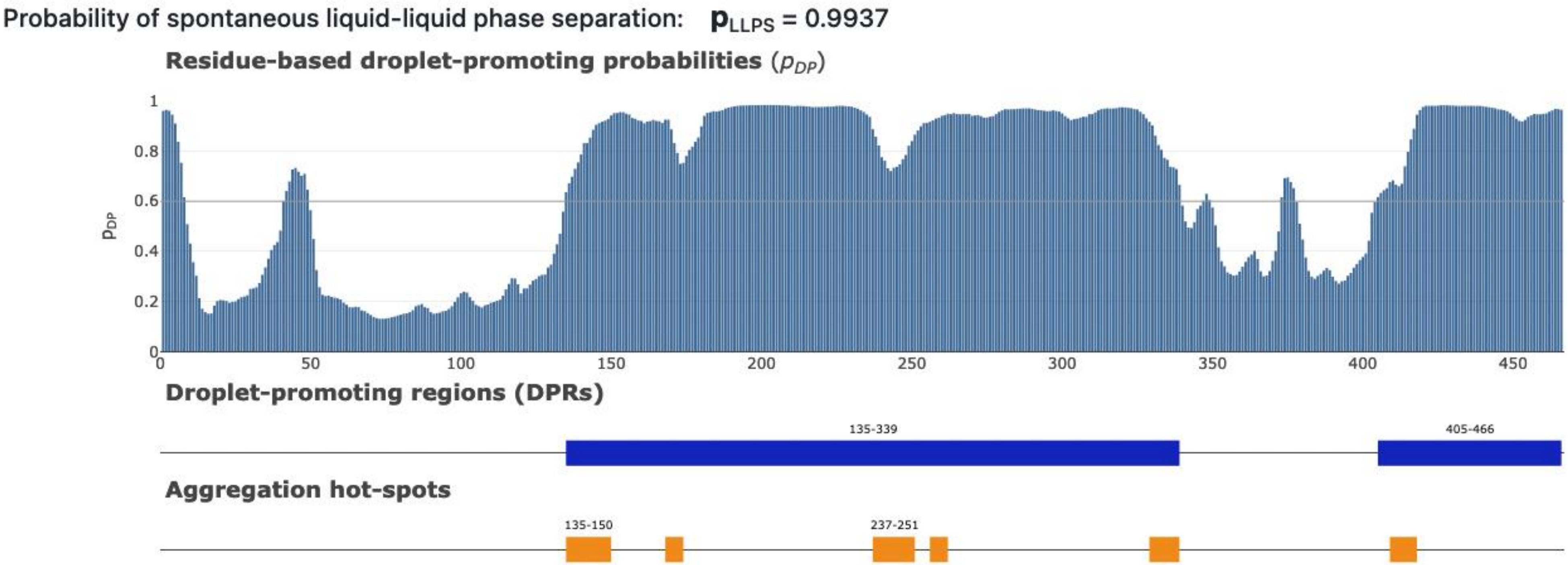

Probability of spontaneous liquid-liquid phase separation: p_{LLPS} = 0.9937
Residue-based droplet-promoting probabilities (p_{DP})
p_{DP}
1
0.8
0.6
0.4
0.2
0
0
50
100
150
200
250
300
350
400
450
Droplet-promoting regions (DPRs)
135-339
405-466
Aggregation hot-spots
135-150
237-251

E

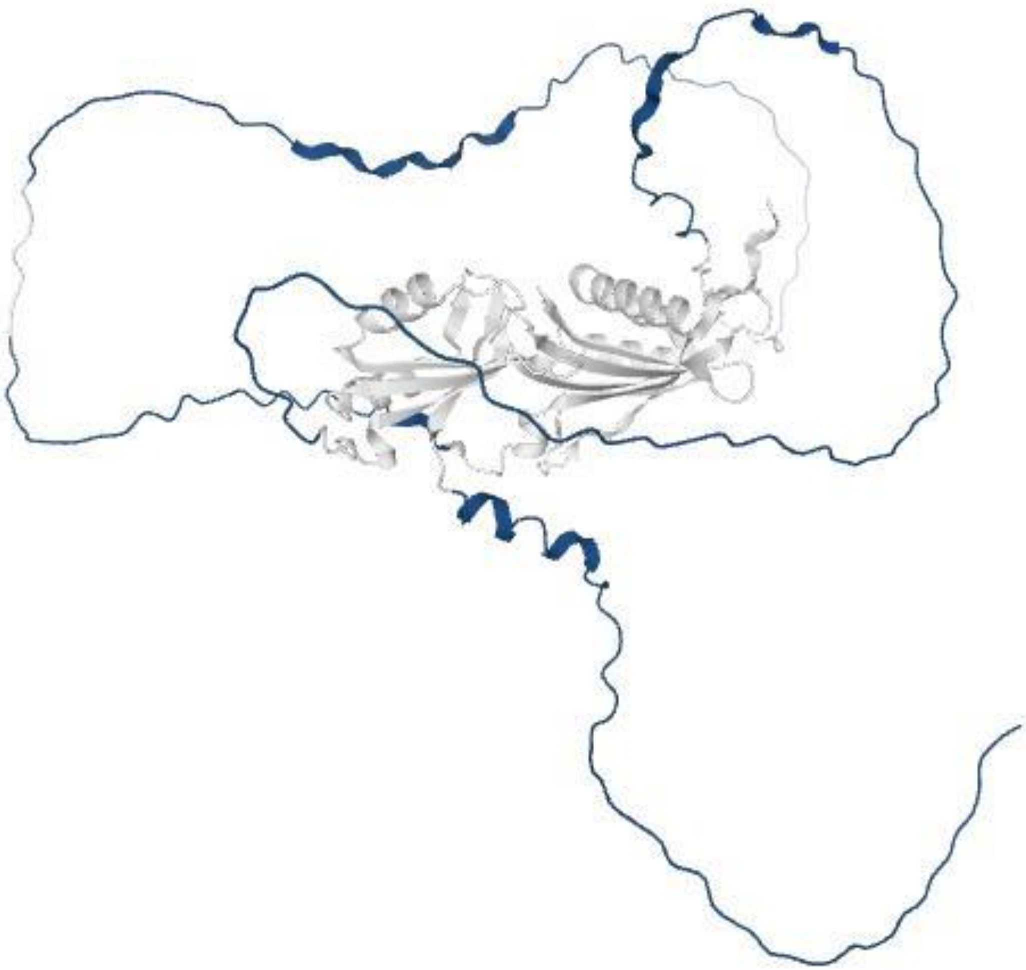

F

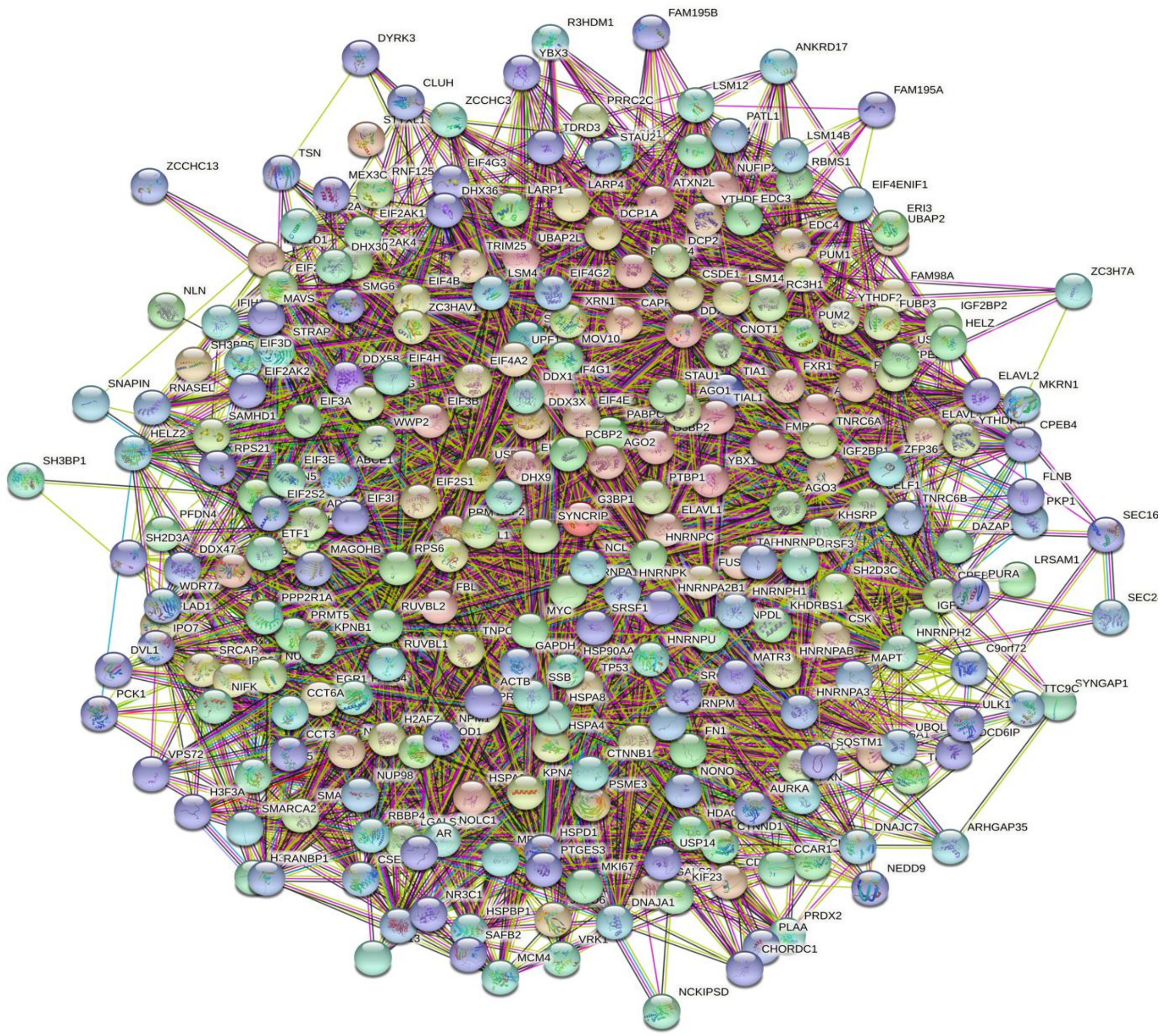

minimum required interaction score: medium confidence (0.400)
number of nodes: 269

number of edges:	5342
average node degree:	39.7
avg. local clustering coefficient:	0.541
expected number of edges:	1729
PPI enrichment p-value:	< 1.0e-16

G

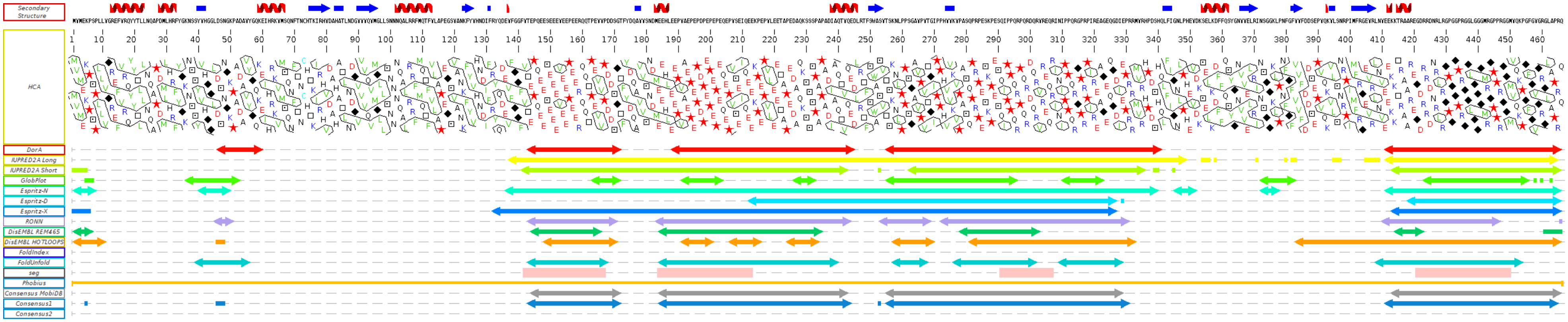
Secondary Structure
HCA
DorA
IUPRED2A Long
IUPRED2A Short
GlobPlot
Espritz-N
Espritz-D
Espritz-X
RONN
DisEMBL REM465
DisEMBL HOTLOOPS
FoldIndex
FoldUnfold
seg
Phobius
Consensus MobiDB
Consensus1
Consensus2
1 10 20 30 40 50 60 70 80 90 100 110 120 130 140 150 160 170 180 190 200 210 220 230 240 250 260 270 280 290 300 310 320 330 340 350 360 370 380 390 400 410 420 430 440 450 460

H

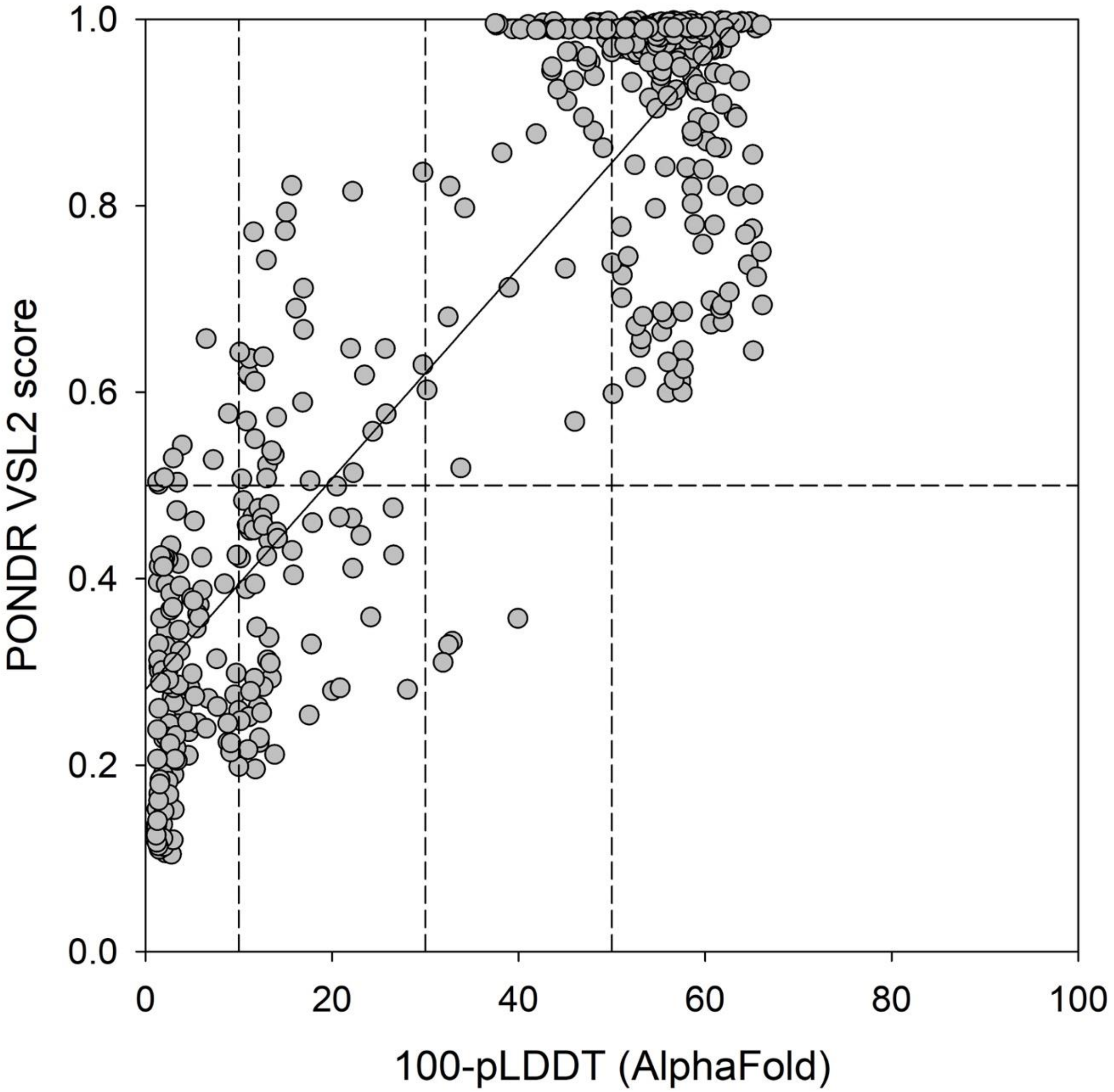

1.0
0.8
0.6
0.4
0.2
0.0
PONDR VSL2 score
0
20
40
60
80
100
100-pLDDT (AlphaFold)

Supplementary Figure S6. Functional disorder in Ras GTPase-activating protein-binding protein 2, G3BP2. **A.** Amino acid sequence. **B.** RIDAO-generated disorder profile. **C.** D2P2-generated functional disorder profile. **D.** FuzDrop output. **E.** AlphaFold2 structure with FuzDrop annotations. **F.** STRING-generated PPI network with corresponding statistics. **G.** MeDor output. **H.** Plot of the *per* residue disorder score (as provided by PONDR® VLS2) versus (100-pLDDT), where pLDDT is the corresponding AlphaFold2 confidence score.

A

>sp|Q9UN86|G3BP2_HUMAN Ras GTPase-activating protein-binding protein 2 OS=Homo sapiens OX=9606 GN=G3BP2 PE=1 SV=2
MVMEKPSPLLVGREFVRQYYTLLNKAPEYLHRFYGRNSSYVHGGVDASGKPQEAVYGQNDIHHKVLSLNFSECHTKIRHVDAHATLSDGVVVQVMGLLSNSGQPERKFMQTFVLAPEG
SVPNKFYVHNDMFRYEDEVFGDSEPELDEESEDEVEEEQEERQPSPEPVQENANSGYYEAHPVTNGIEEPLEESSHEPEPEPESETKTEELKPQVEEKNLEELEEKSTTPPPAEPVSL
PQEPPKAFSWASVTSKNLPPSGTVSSSGIPPHVKAPVSQPRVEAKPEVQSQPPRVREQRPRERPGFPPRGPRPGRGDMEQNDSDNRRIIRYPDSHQLFVGNLPHDIDENELKEFFMSF
GNVVELRINTKGVGGKLPNFGFVVFDDSEPVQRILIAKPIMFRGEVRLNVEEKKTRAARERETRGGGDDRRDIRRNDRGPGGPRGIVGGGMMRDRDGRGPPPRGGMAQKLGSGRGTGQ
MEGRFTGQRR

B

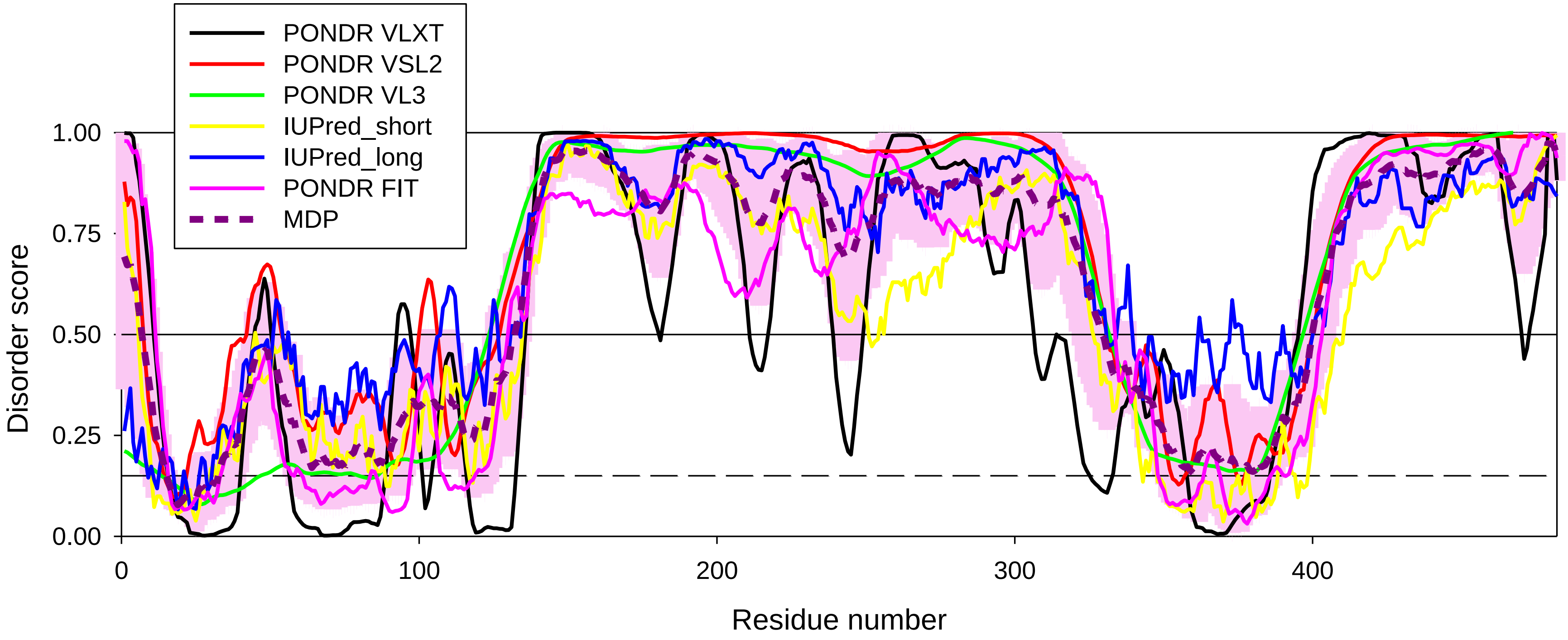

C

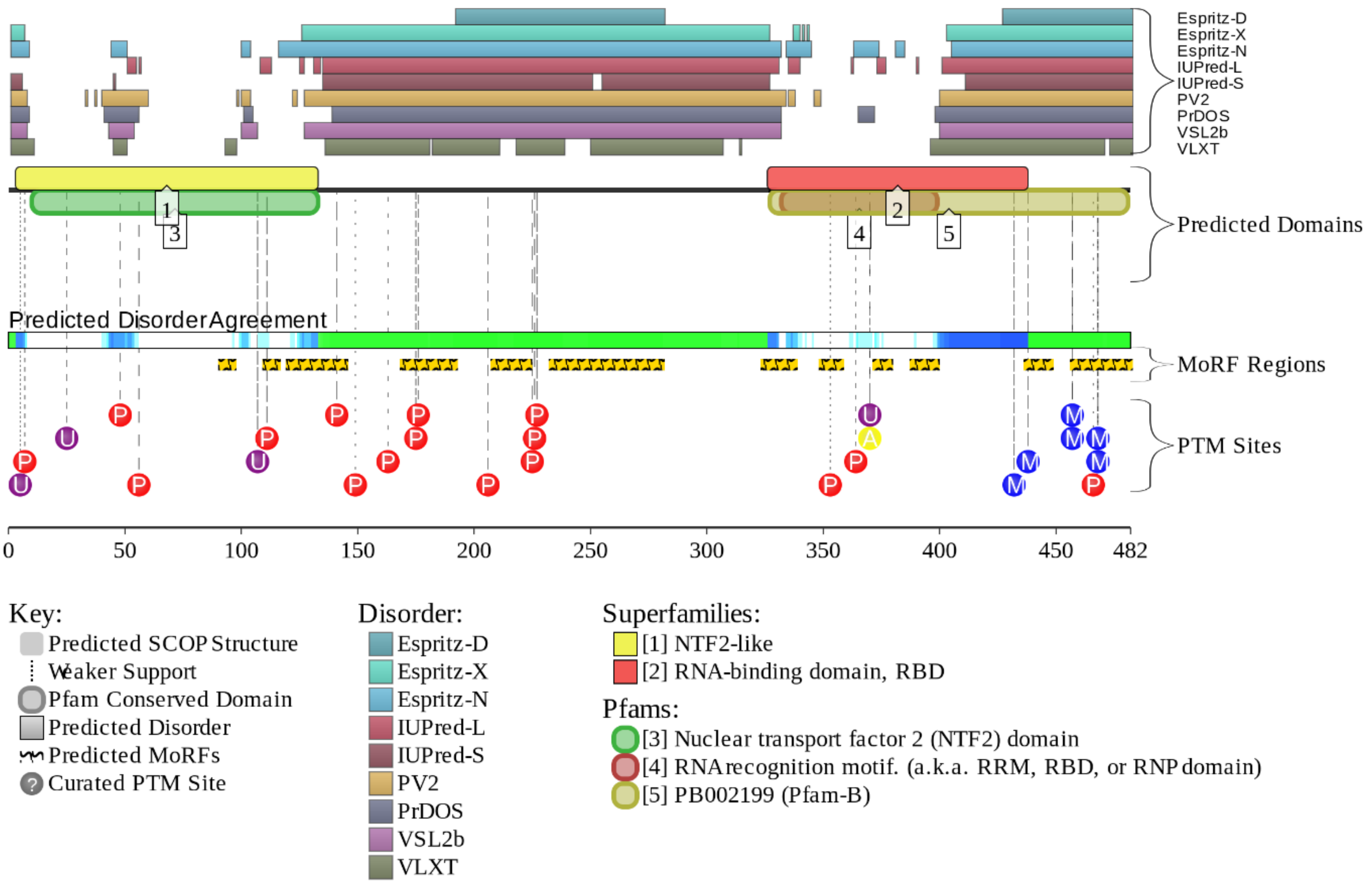

Espritz-D
Espritz-X
Espritz-N
IUPred-L
IUPred-S
PV2
PrDOS
VSL2b
VLXT
Predicted Domains
Predicted Disorder Agreement
MoRF Regions
PTM Sites
1
2
3
4
5
0
50
100
150
200
250
300
350
400
450
482
Key:
Predicted SCOP Structure
Weaker Support
Pfam Conserved Domain
Predicted Disorder
Predicted MoRFs
Curated PTM Site
Disorder:
Espritz-D
Espritz-X
Espritz-N
IUPred-L
IUPred-S
PV2
PrDOS
VSL2b
VLXT
Superfamilies:
[1] NTF2-like
[2] RNA-binding domain, RBD
Pfams:
[3] Nuclear transport factor 2 (NTF2) domain
[4] RNA recognition motif. (a.k.a. RRM, RBD, or RNP domain)
[5] PB002199 (Pfam-B)

D

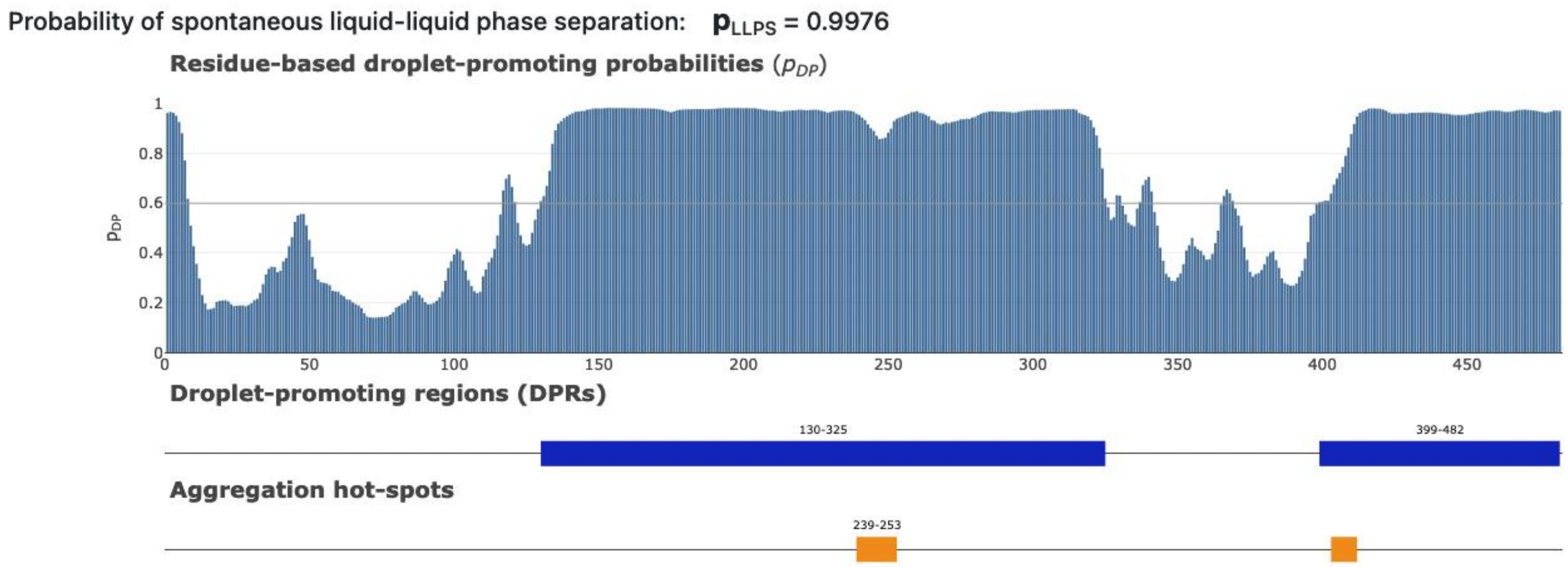

Probability of spontaneous liquid-liquid phase separation: p_{LLPS} = 0.9976
Residue-based droplet-promoting probabilities (p_{DP})
p_{DP}
1
0.8
0.6
0.4
0.2
0
0
50
100
150
200
250
300
350
400
450
Droplet-promoting regions (DPRs)
130-325
399-482
Aggregation hot-spots
239-253

E

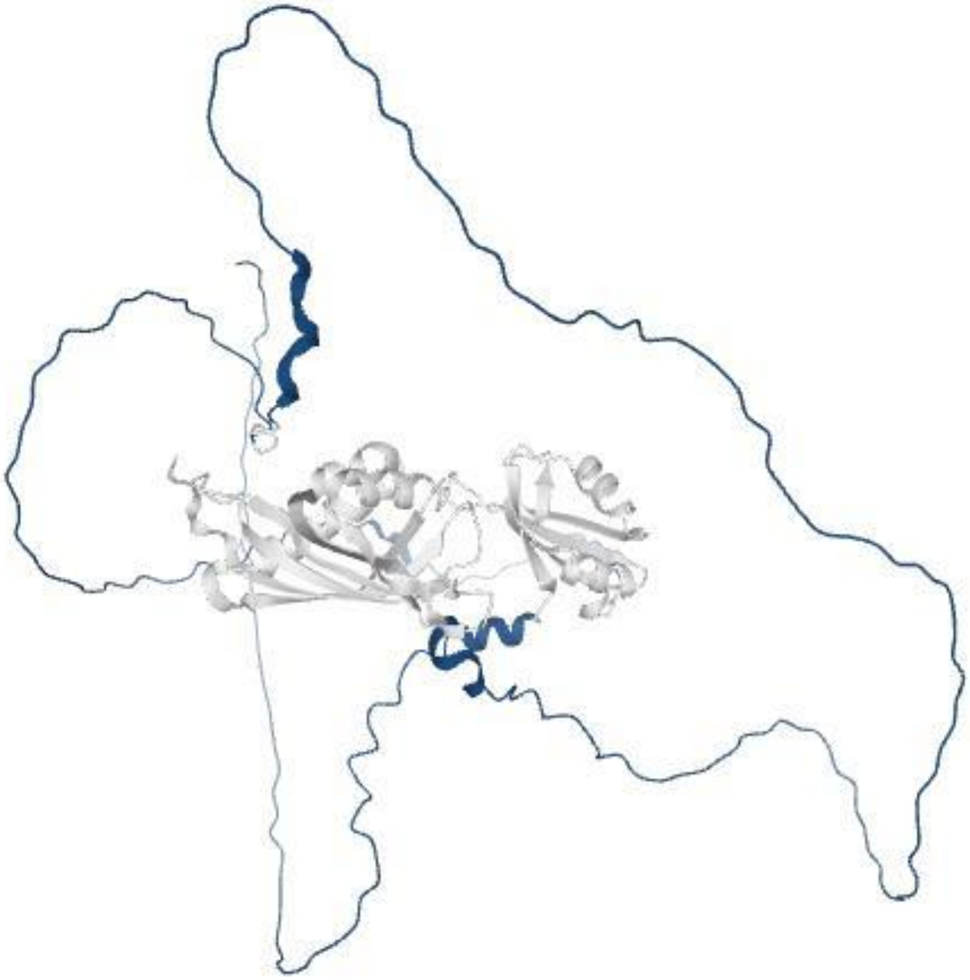

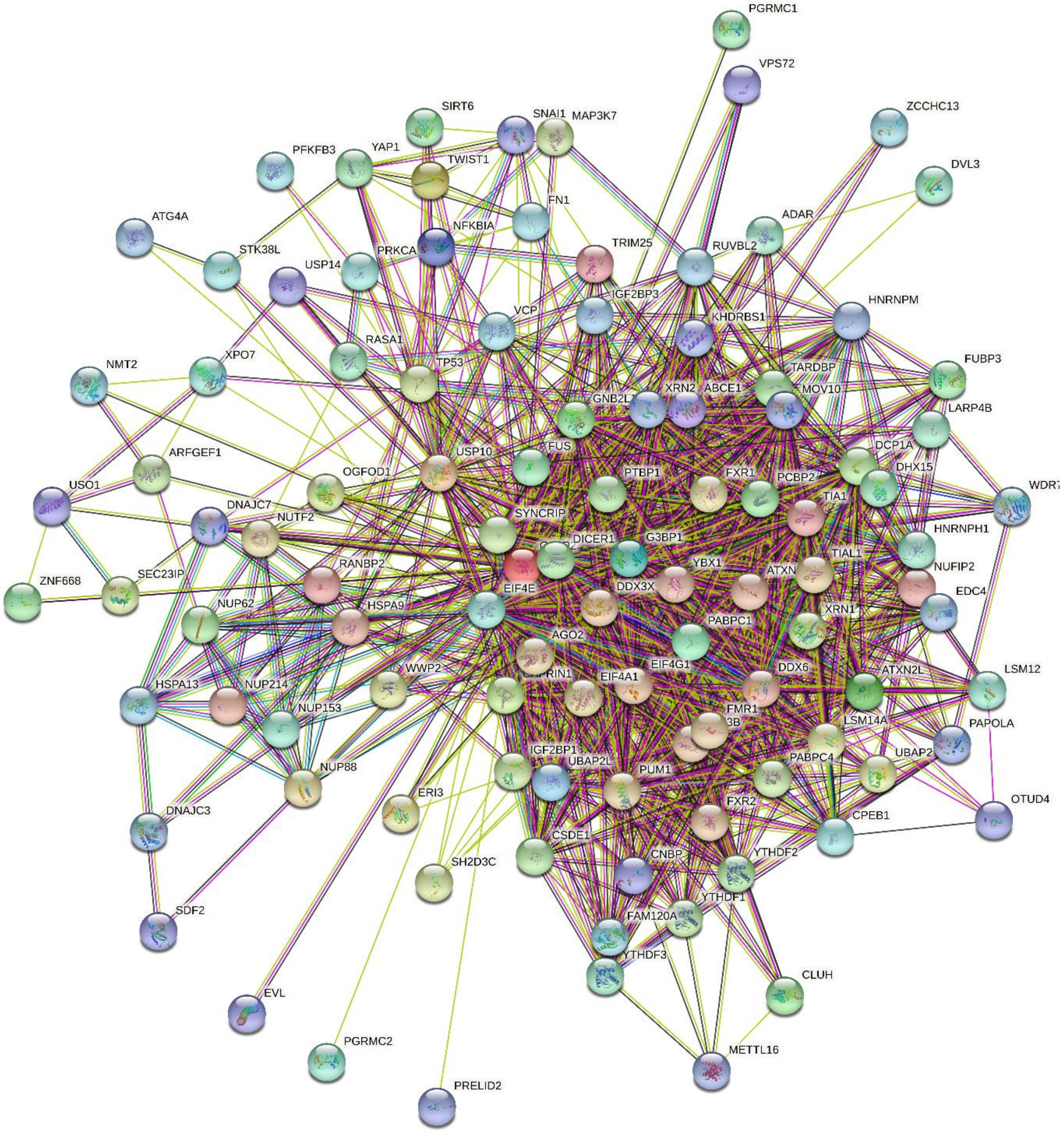
PGRMC1
VPS72
ZCCHC13
DVL3
SIRT6
SNAI1
MAP3K7
PFKFB3
YAP1
TWIST1
ADAR
FN1
ATG4A
NFKBIA
TRIM25
STK38L
PRKCA
RUVBL2
USP14
IGF2BP3
HNRNPM
VCP
KHDRBS1
RASA1
FUBP3
NMT2
XPO7
TP53
TARDBP
XRN2
ABCE1
MOV10
GNB2L1
LARP4B
FUS
DCP1A
ARFGEF1
USP10
DHX15
USO1
OGFOD1
PTBP1
FXR1
PCBP2
DNAJC7
TIA1
NUTF2
SYNCRIP
HNRNPH1
DICER1
G3BP1
ZNF668
SEC23IP
RANBP2
YBX1
TIAL1
NUFIP2
EIF4E
DDX3X
EDC4
NUP62
HSPA9
XRN1
PABPC1
AGO2
HSPA13
NUP214
WWP2
EIF4G1
DDX6
ATXN2L
LSM12
EIF4A1
NUP153
FMR1
LSM14A
PAPOLA
IGF2BP1
UBAP2L
PUM1
PABPC4
UBAP2
NUP88
ERI3
FXR2
OTUD4
DNAJC3
CPEB1
CSDE1
SH2D3C
CNBP
YTHDF2
SDF2
YTHDF1
FAM120A
YTHDF3
CLUH
EVL
METTL16
PGRMC2
PRELID2

F

minimum required interaction score: medium confidence (0.400)
number of nodes: 106
number of edges: 1045
average node degree: 19.7
avg. local clustering coefficient: 0.667
expected number of edges: 281
PPI enrichment p-value: < 1.0e-16

G

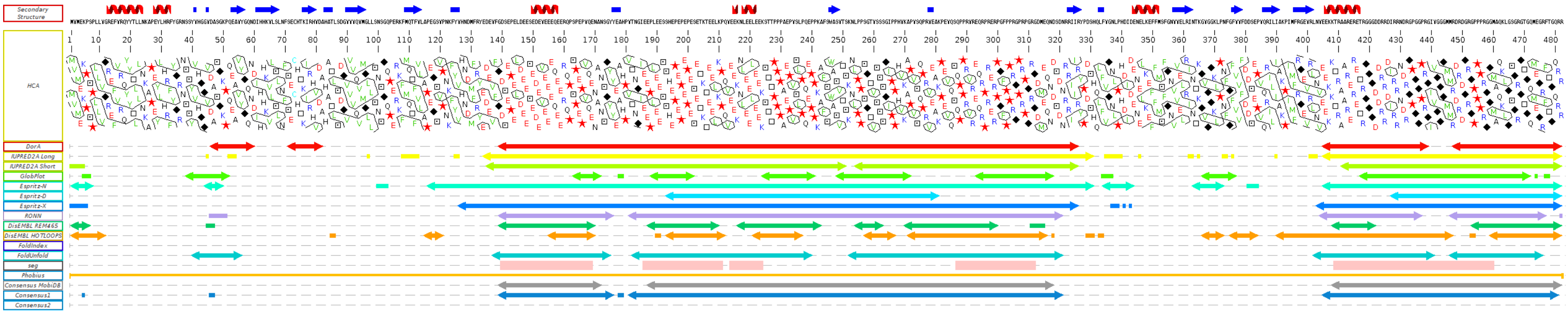
Secondary Structure
HCA
DorA
IUPRED2A Long
IUPRED2A Short
GlobPlot
Espritz-N
Espritz-D
Espritz-X
RONN
DisEMBL REM465
DisEMBL HOTLOOPS
FoldIndex
FoldUnfold
seg
Phobius
Consensus MobiDB
Consensus1
Consensus2
1 10 20 30 40 50 60 70 80 90 100 110 120 130 140 150 160 170 180 190 200 210 220 230 240 250 260 270 280 290 300 310 320 330 340 350 360 370 380 390 400 410 420 430 440 450 460 470 480

H

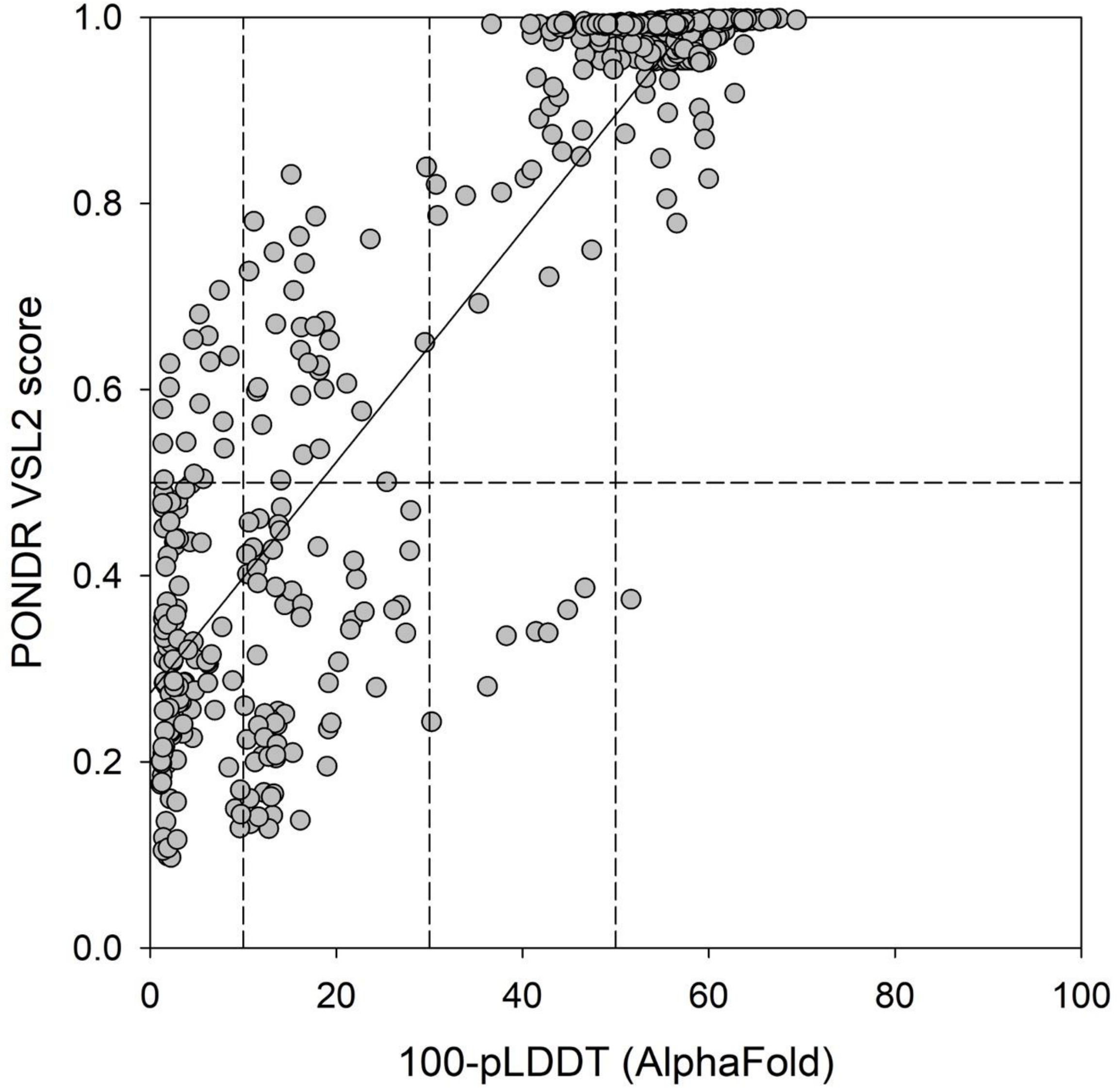

1.0
0.8
0.6
0.4
0.2
0.0
0
20
40
60
80
100
PONDR VSL2 score
100-pLDDT (AlphaFold)

Supplementary Figure S7. Functional disorder in cellular tumor antigen p53. **A.** Amino acid sequence. **B.** RIDAO-generated disorder profile. **C.** D2P2-generated functional disorder profile. **D.** FuzDrop output. **E.** AlphaFold structure with FuzDrop annotations. **F.** STRING-generated PPI network with corresponding statistics. Note that due to the high p53 interactivity, STRING analysis was conducted using highest confidence of 0.900. **G.** MeDor output. **H.** Plot of the *per* residue disorder score (as provided by PONDR® VLS2) versus (100-pLDDT), where pLDDT is the corresponding AlphaFold2 confidence score.

A

>sp|P04637|P53_HUMAN Cellular tumor antigen p53 OS=Homo sapiens OX=9606 GN=TP53 PE=1 SV=4
MEEPQSDPSVEPPLSQETFSDLWKLLPENNVLSPLPSQAMDDLMLSPDDIEQWFTEDPGPDEAPRMPEAAPPVAPAPAAPTPAAPAPAPSWPLSSSVPSQKTYQGSY
GFRLGFLHSGTAKSVTCTYSPALNKMFCQLAKTCPVQLWVDSTPPPGTRVRAMAIYKQSQHMTEVVRRCPHHERCSDSDGLAPPQHLIRVEGNLRVEYLDDRNTFRH
SVVVPYEPPEVGSDCTTIHYNYMCNSSCMGGMNRRPILTIITLEDSSGNLLGRNSFEVRVCACPGRDRRTEEENLRKKGEPHHELPPGSTKRALPNNTSSSPQPKKK
PLDGEYFTLQIRGRERFEMFRELNEALELKDAQAGKEPGGSRAHSSHLKSKKGQSTSRHKKLMFKTEGPDSD

B

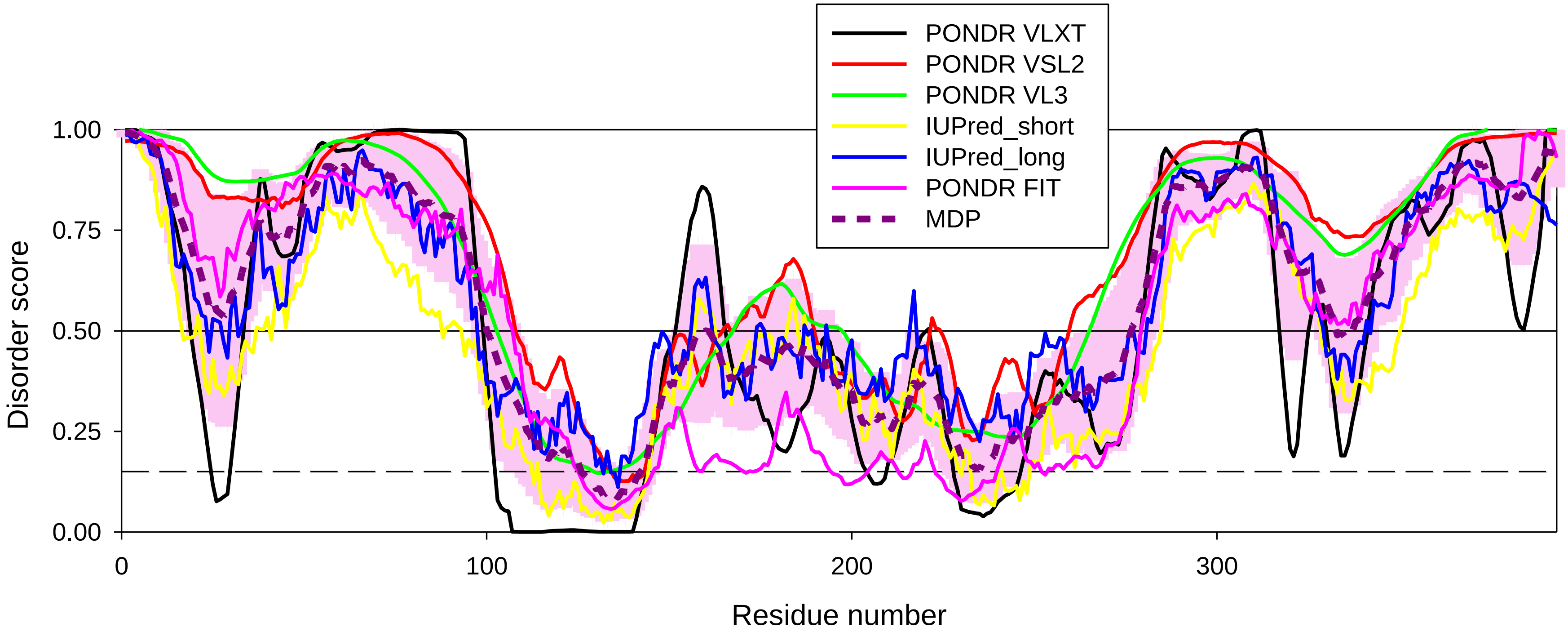

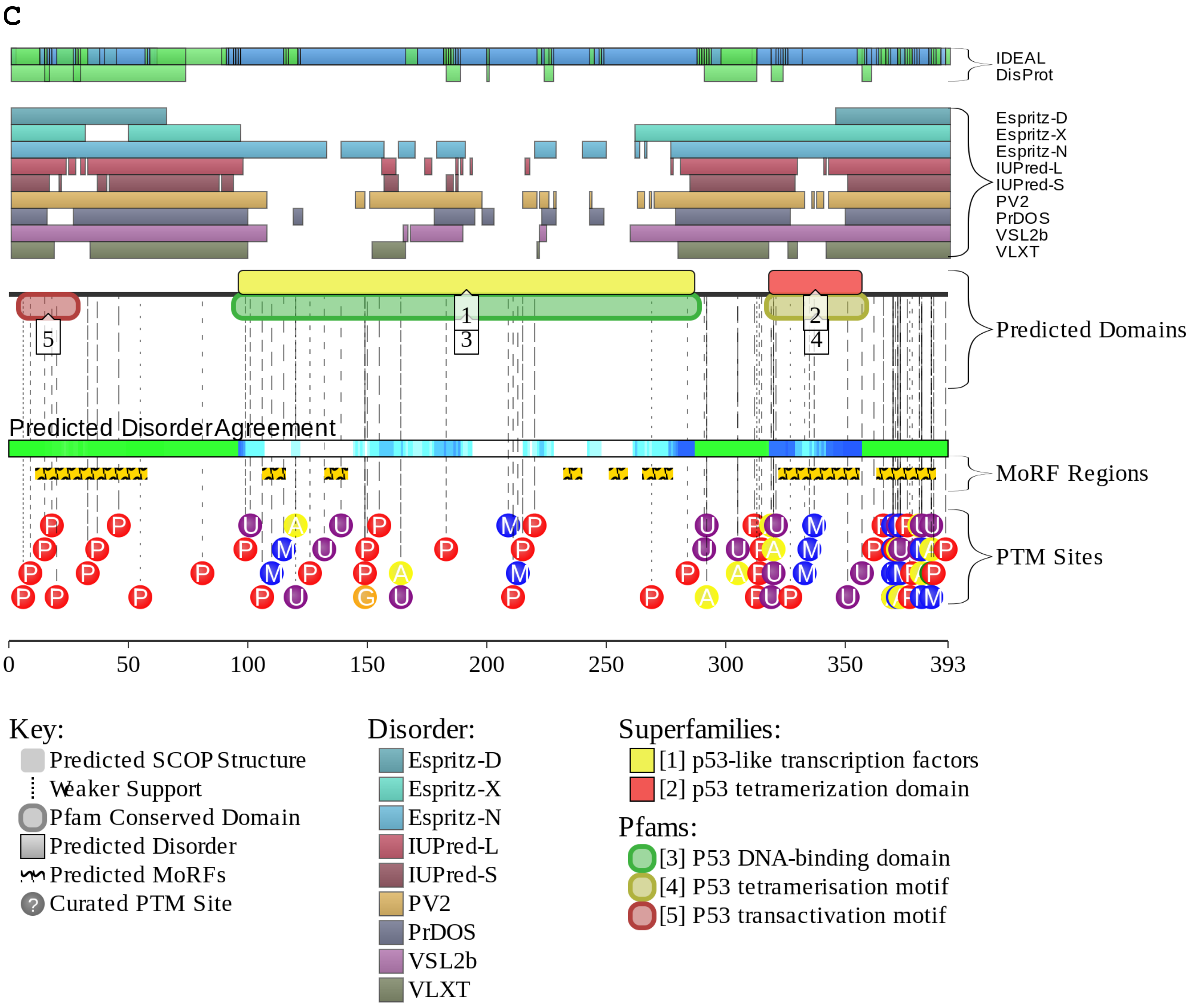
C
IDEAL
DisProt
Espritz-D
Espritz-X
Espritz-N
IUPred-L
IUPred-S
PV2
PrDOS
VSL2b
VLXT
Predicted Domains
Predicted Disorder Agreement
MoRF Regions
PTM Sites
0
50
100
150
200
250
300
350
393
Key:
Predicted SCOP Structure
Weaker Support
Pfam Conserved Domain
Predicted Disorder
Predicted MoRFs
Curated PTM Site
Disorder:
Espritz-D
Espritz-X
Espritz-N
IUPred-L
IUPred-S
PV2
PrDOS
VSL2b
VLXT
Superfamilies:
[1] p53-like transcription factors
[2] p53 tetramerization domain
Pfams:
[3] P53 DNA-binding domain
[4] P53 tetramerisation motif
[5] P53 transactivation motif

D

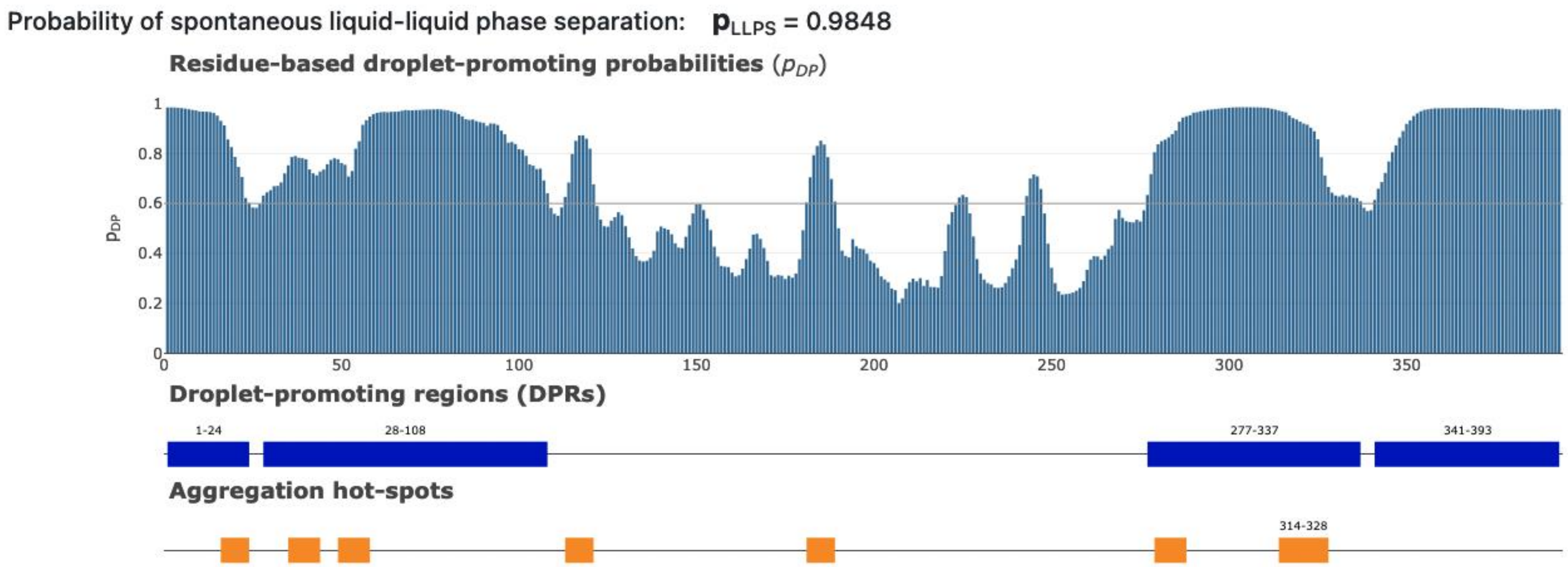

Probability of spontaneous liquid-liquid phase separation: pLLPS = 0.9848
Residue-based droplet-promoting probabilities (pDP)
pDP
1
0.8
0.6
0.4
0.2
0
0
50
100
150
200
250
300
350
Droplet-promoting regions (DPRs)
1-24
28-108
277-337
341-393
Aggregation hot-spots
314-328

E

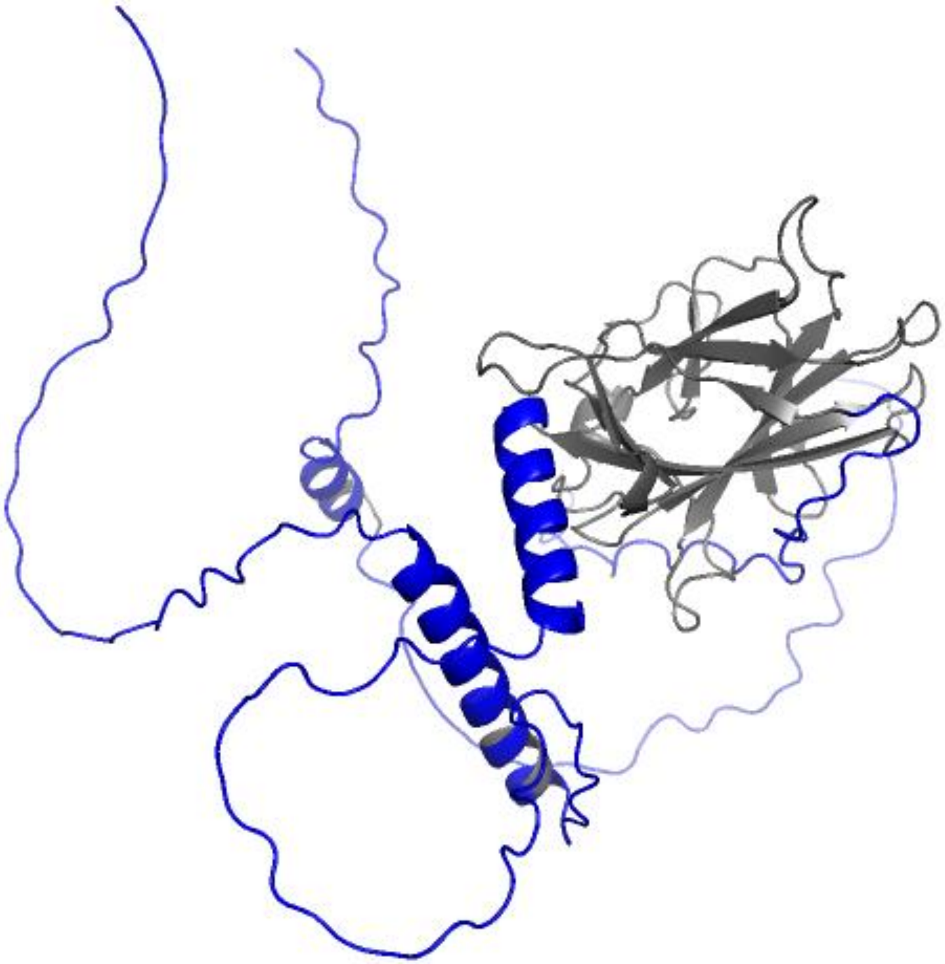

F

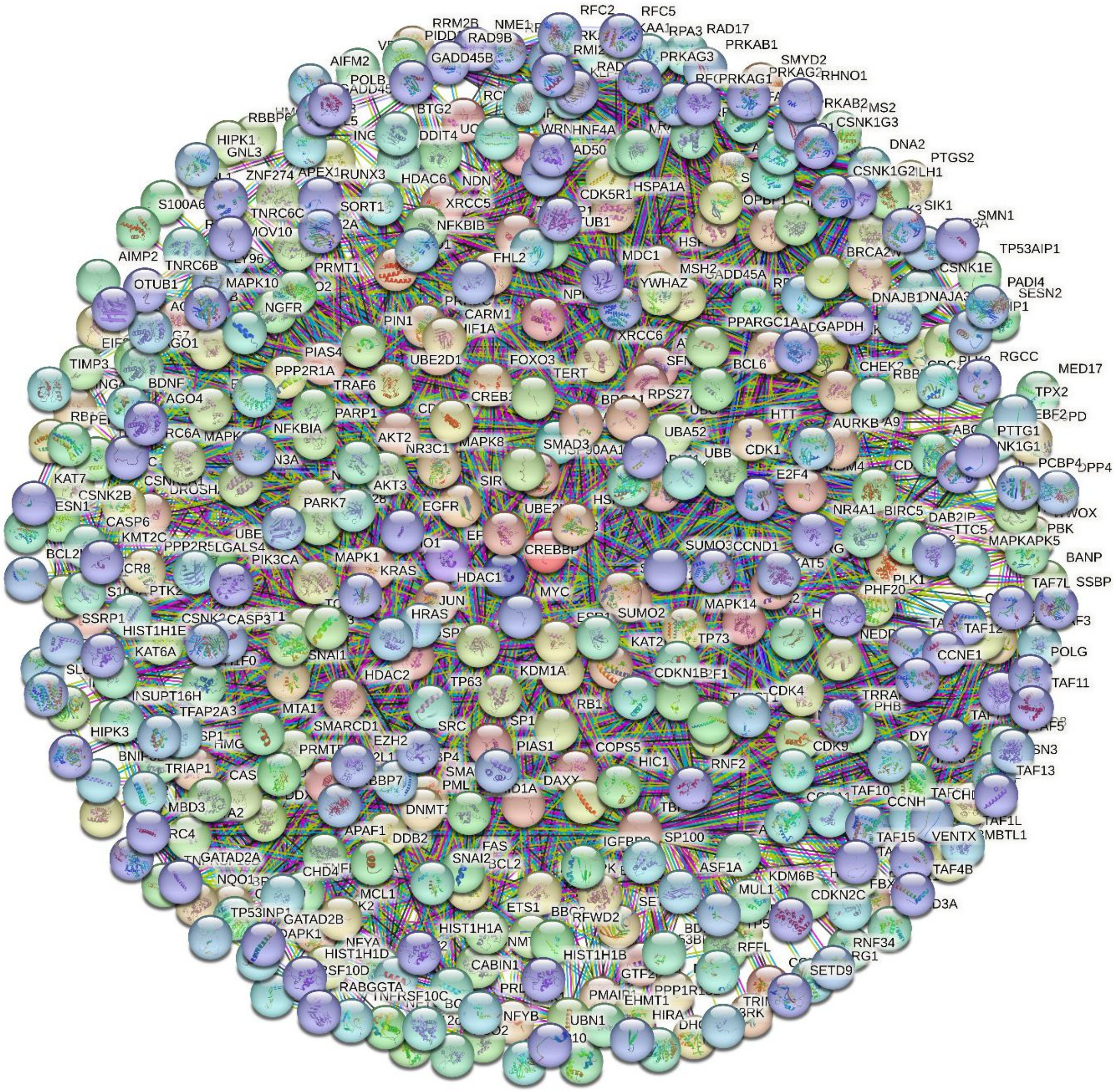

RRM2B
NME1
RFC2
RFC5
RAD9B
RPA3
RAD17
PRKAB1
AIFM2
GADD45B
RMI2
PRKAG3
SMYD2
POLB
PRKAG1
RHNO1
PRKAB2
BTG2
CSNK1G3
HIPK1
DDIT4
GNL3
DNA2
PTGS2
ZNF274
APEX1
RUNX3
HDAC6
NDN
CDK5R1
HSPA1A
CSNK1G2
S100A6
XRCC5
SORT1
SIK1
SMN1
TNRC6C
MOV10
NFKBIB
TP53AIP1
AIMP2
TNRC6B
PRMT1
FHL2
MDC1
MSH2
GADD45A
CSNK1E
OTUB1
MAPK10
YWHAZ
DNAJB1
PADI4
SESN2
NGFR
CARM1
PPARGC1A
PIN1
HIF1A
XRCC6
TIMP3
PIAS4
PPP2R1A
UBE2D1
FOXO3
TERT
BCL6
RGCC
MED17
BDNF
TRAF6
RPS27
TPX2
AGO4
PARP1
HTT
AURKB
NFKBIA
AKT2
MAPK8
NR3C1
SMAD3
UBA52
UBB
CDK1
PTTG1
PCBP4
KAT7
CSNK2B
AKT3
E2F4
PARK7
EGFR
CASP6
NR4A1
BIRC5
DAB2IP
TTC5
PBK
KMT2C
MAPKAPK5
CREBBP
SUMO3
CCND1
BANP
PIK3CA
MAPK1
KRAS
HDAC1
MYC
PLK1
PHF20
TAF7L
JUN
SUMO2
MAPK14
SSRP1
HIST1H1E
CASP3
HRAS
TP73
TAF12
CCNE1
POLG
KAT6A
SNAI1
HDAC2
TP63
KDM1A
CDKN1B
CDK4
TAF11
SUPT16H
MTA1
RB1
PHB
HIPK3
SMARCD1
EZH2
SRC
SP1
CDK9
PIAS1
COPS5
TRIAP1
HIC1
RNF2
TAF13
DAXX
MBD3
DNMT1
TAF10
CCNH
APAF1
DDB2
FAS
SP100
TAF15
VENTX
GATAD2A
CHD4
SNAI2
BCL2
ASF1A
TAF4B
NQO1
MCL1
MUL1
KDM6B
CDKN2C
ETS1
RFWD2
TP53INP1
GATAD2B
HIST1H1A
RNF34
NFYA
HIST1H1D
CABIN1
HIST1H1B
RFFL
SETD9
RABGGTA
PMAIP1
EHMT1
NFYB
UBN1
HIRA
TAF1L

minimum required interaction score: highest confidence (0.900)
number of nodes: 427
number of edges: 3677
average node degree: 17.2
avg. local clustering coefficient: 0.62
expected number of edges: 1903
PPI enrichment p-value: < 1.0e-16

G

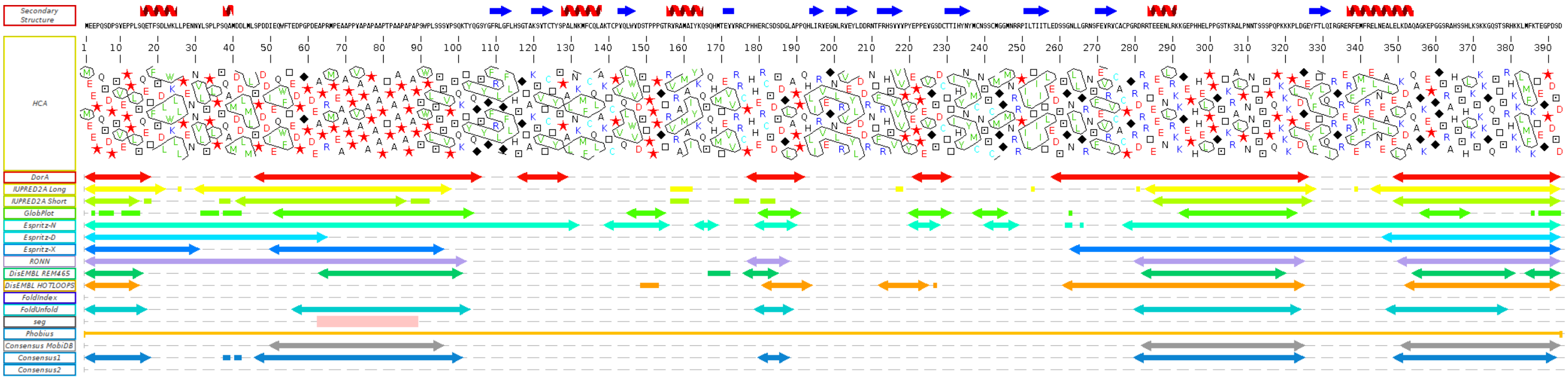

Secondary Structure
HCA
DorA
IUPRED2A Long
IUPRED2A Short
GlobPlot
Espritz-N
Espritz-D
Espritz-X
RONN
DisEMBL REM465
DisEMBL HOTLOOPS
FoldIndex
FoldUnfold
seg
Phobius
Consensus MobiDB
Consensus1
Consensus2

H

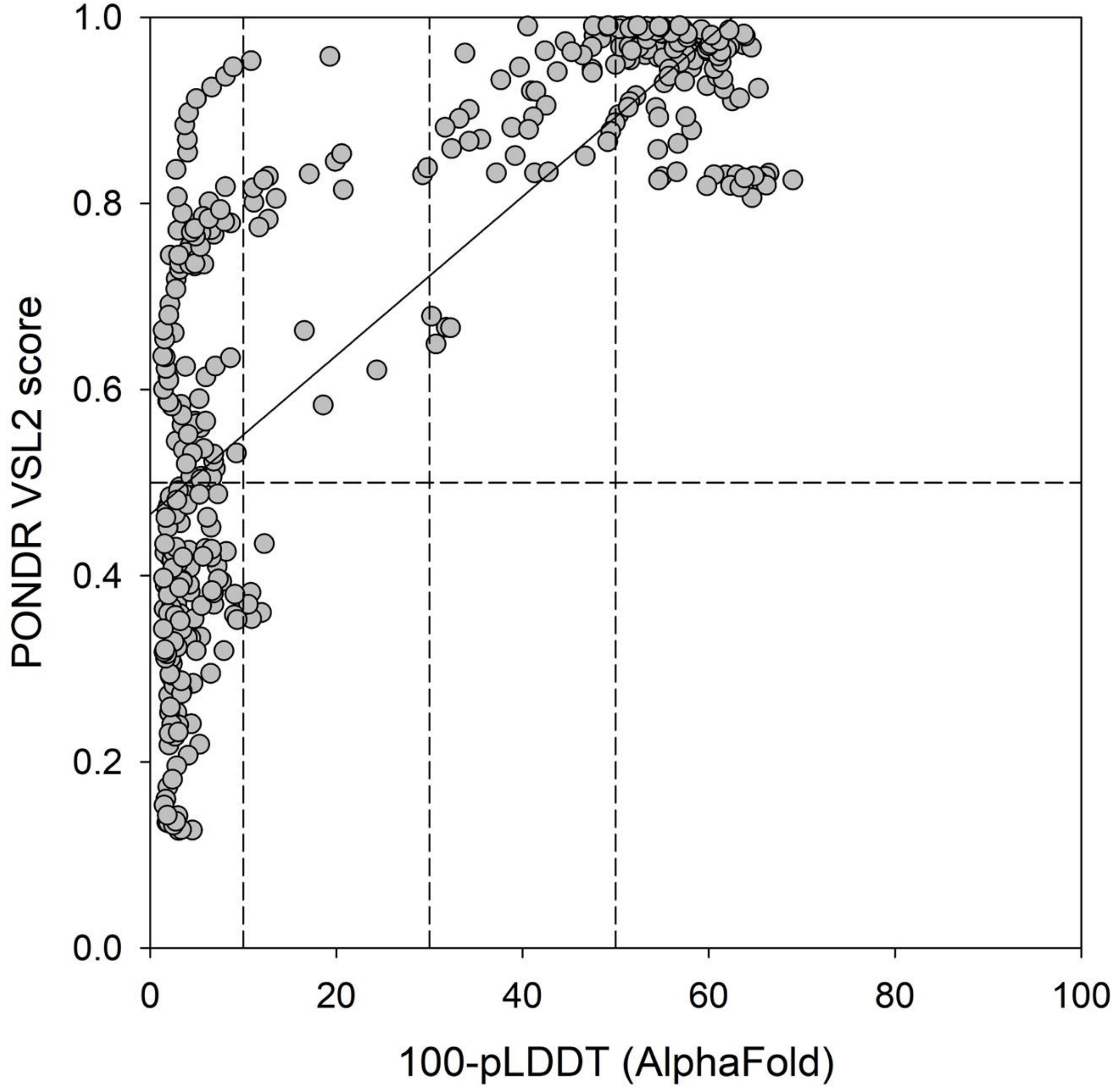
PONDR VSL2 score
1.0
0.8
0.6
0.4
0.2
0.0
0
20
40
60
80
100
100-pLDDT (AlphaFold)

Supplementary Figure S8. Functional disorder in transcription factor p65. **A.** Amino acid sequence. **B.** RIDAO-generated disorder profile. **C.** D2P2-generated functional disorder profile. **D.** FuzDrop output. **E.** AlphaFold2 structure with FuzDrop annotations. **F.** STRING-generated PPI network with corresponding statistics. **G.** MeDor output. **H.** Plot of the *per* residue disorder score (as provided by PONDR® VLS2) versus (100-pLDDT), where pLDDT is the corresponding AlphaFold2 confidence score.

A

>sp|Q04206|TF65_HUMAN Transcription factor p65 OS=Homo sapiens OX=9606 GN=RELA PE=1 SV=2
MDELFPLIFPAEPAQASGPYVEIIEQPKQRGMRFRYKCEGRSAGSIPGERSTDTTKTHPTIKINGYTGPGTVRISLVTKDPPHRPHPHELVGKDCRDGFYEAELCPDRCIHSFQNLGI
QCVKKRDLEQAISQRIQTNNNPFQVPIEEQRGDYDLNAVRLCFQVTVRDPSGRPLRLPPVLSHPIFDNRAPNTAELKICRVNRNSGSCLGGDEIFLLCDKVQKEDIEVYFTGPGWEAR
GSFSQADVHRQVAIVFRTPPYADPSLQAPVRVSMQLRRPSDRELSEPMEFQYLPDTDDRHRIEEKRKRTYETFKSIMKKSPFSGPTDPRPPPRRIAVPSRSSASVPKPAPQPYPFTSS
LSTINYDEFPTMVFPSGQISQASALAPAPPQVLPQAPAPAPAPAMVSALAQAPAPVPVLAPGPPQAVAPPAPKPTQAGEGTLSEALLQLQFDDEDLGALLGNSTDPAVFTDLASVDNS
EFQQLLNQGIPVAPHTTEPMLMEYPEAITRLVTGAQRPPDPAPAPLGAPGLPNGLLSGDEDFSSIADMDFSALLSQISS

B

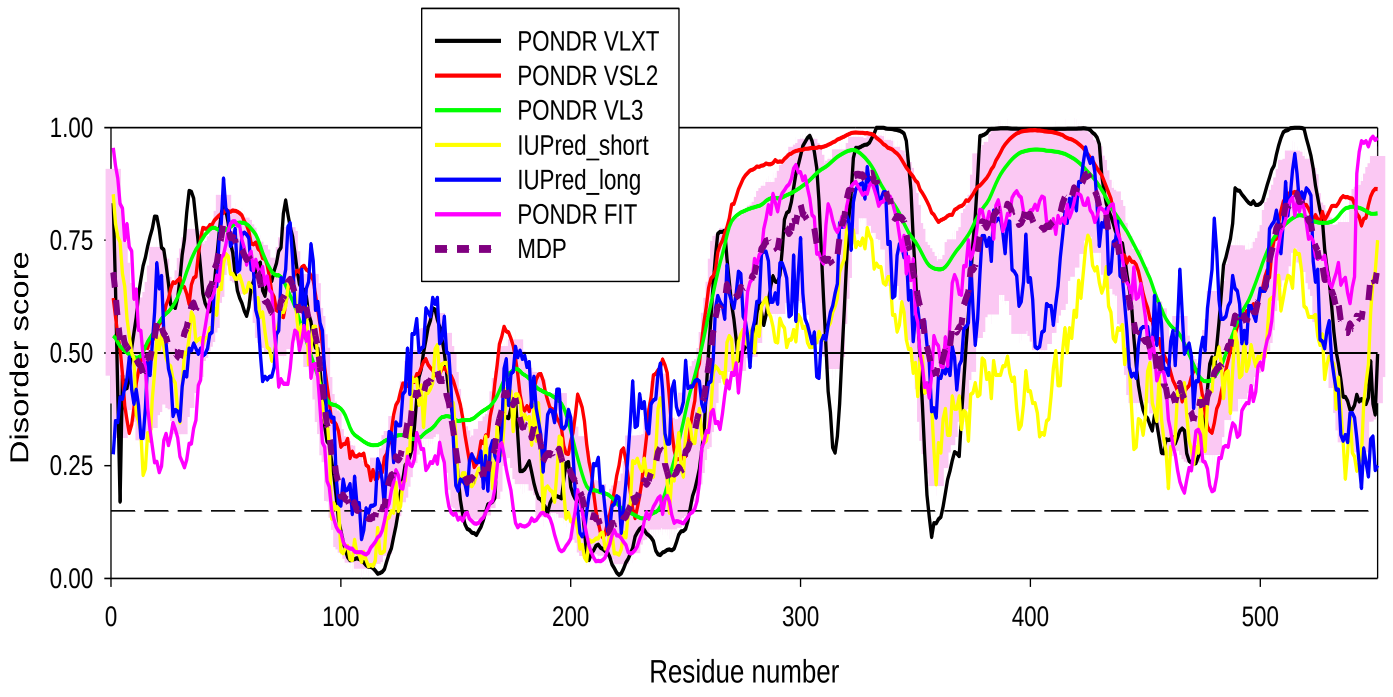

C

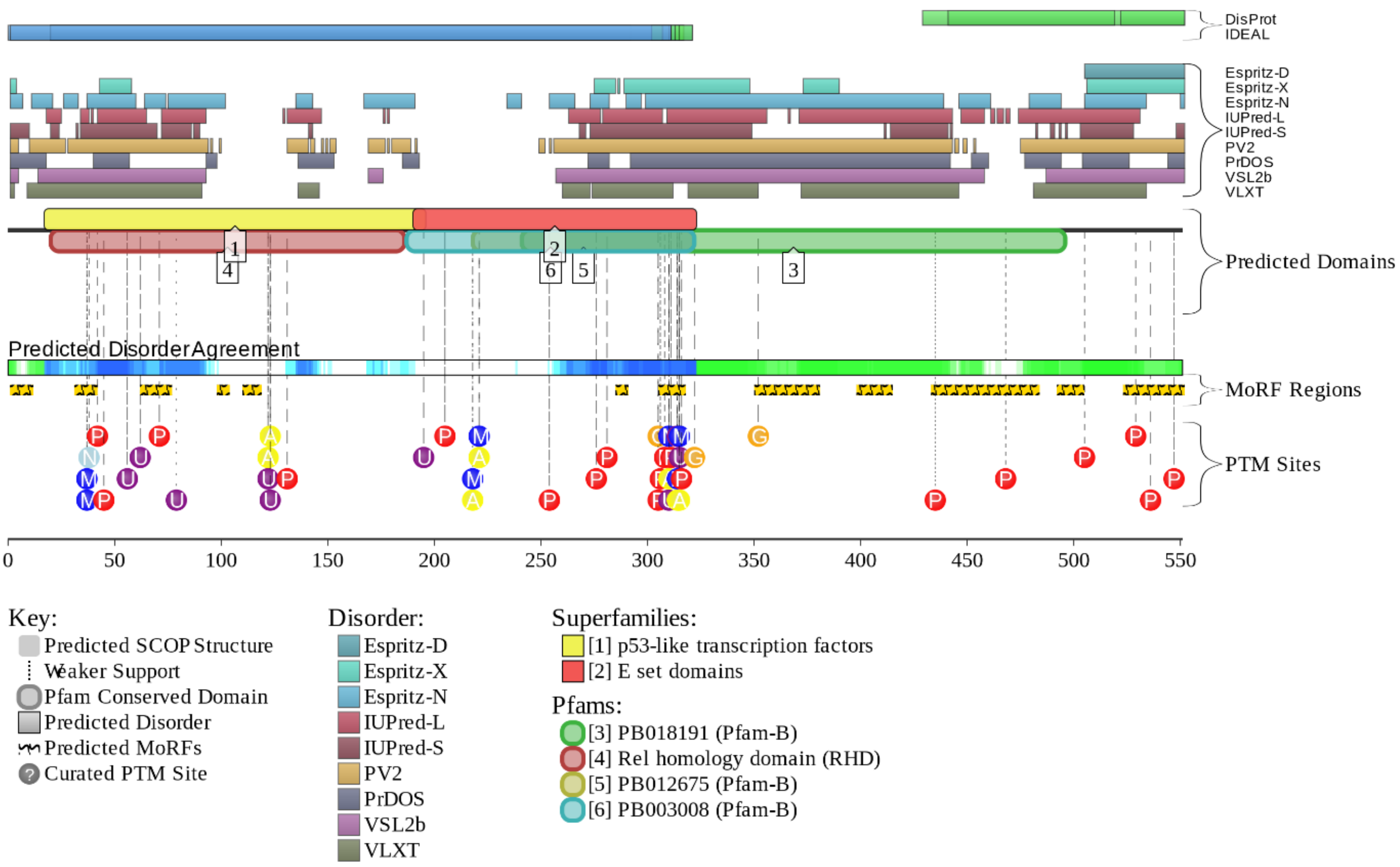
DisProt
IDEAL
Espritz-D
Espritz-X
Espritz-N
IUPred-L
IUPred-S
PV2
PrDOS
VSL2b
VLXT
Predicted Domains
Predicted Disorder Agreement
MoRF Regions
PTM Sites
0
50
100
150
200
250
300
350
400
450
500
550
Key:
Predicted SCOP Structure
Weaker Support
Pfam Conserved Domain
Predicted Disorder
Predicted MoRFs
Curated PTM Site
Disorder:
Espritz-D
Espritz-X
Espritz-N
IUPred-L
IUPred-S
PV2
PrDOS
VSL2b
VLXT
Superfamilies:
[1] p53-like transcription factors
[2] E set domains
Pfams:
[3] PB018191 (Pfam-B)
[4] Rel homology domain (RHD)
[5] PB012675 (Pfam-B)
[6] PB003008 (Pfam-B)

D

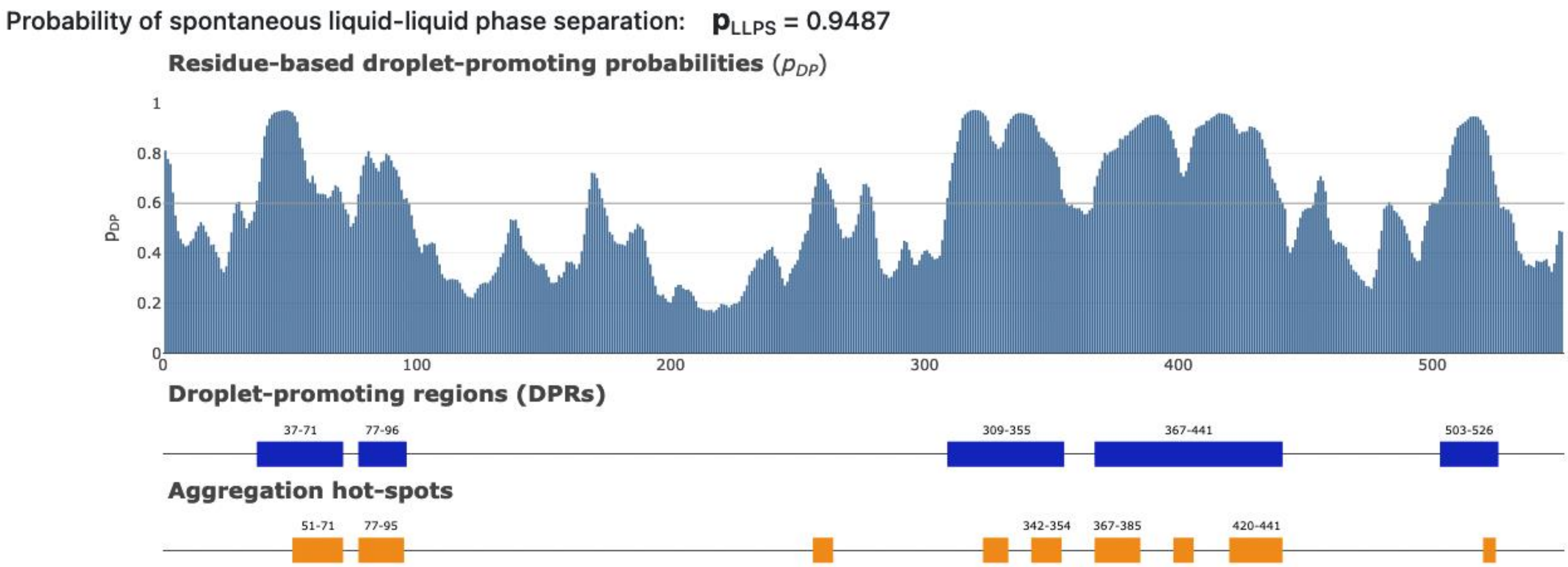

Probability of spontaneous liquid-liquid phase separation: pLLPS = 0.9487
Residue-based droplet-promoting probabilities (pDP)
pDP
1
0.8
0.6
0.4
0.2
0
0
100
200
300
400
500
Droplet-promoting regions (DPRs)
37-71
77-96
309-355
367-441
503-526
Aggregation hot-spots
51-71
77-95
342-354
367-385
420-441

E

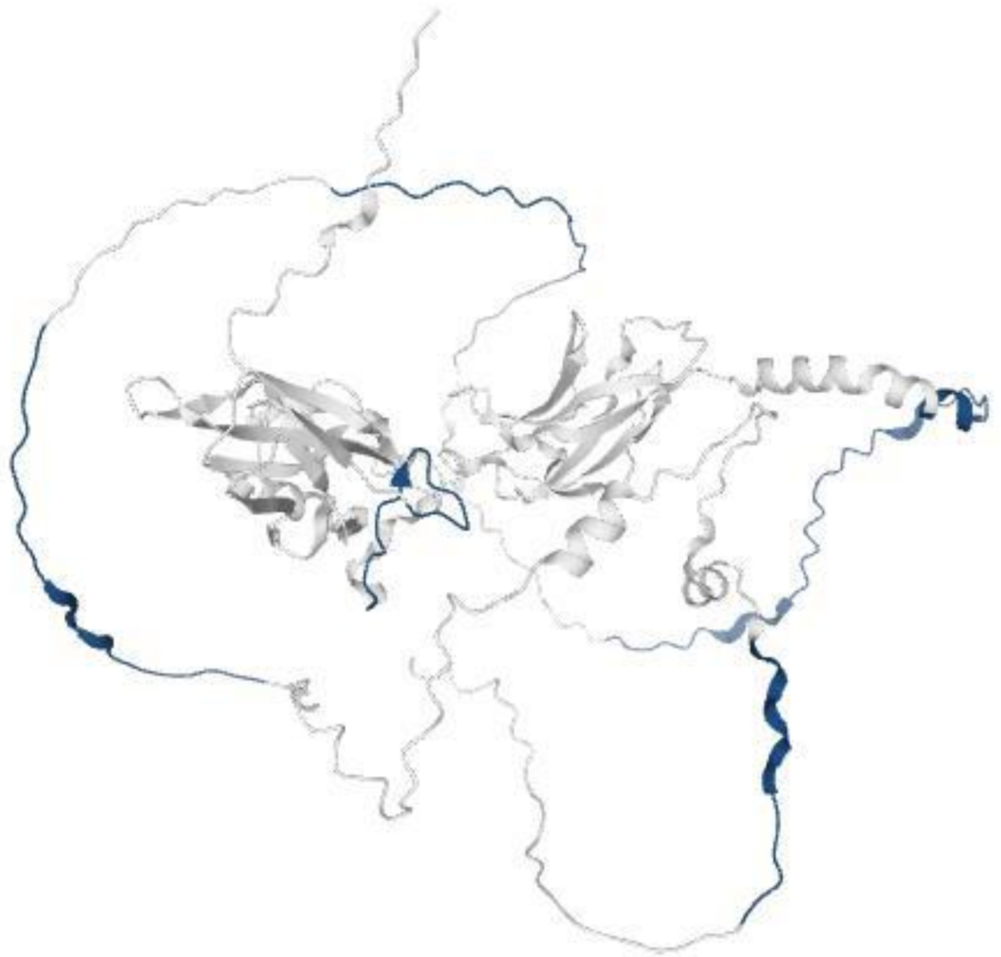

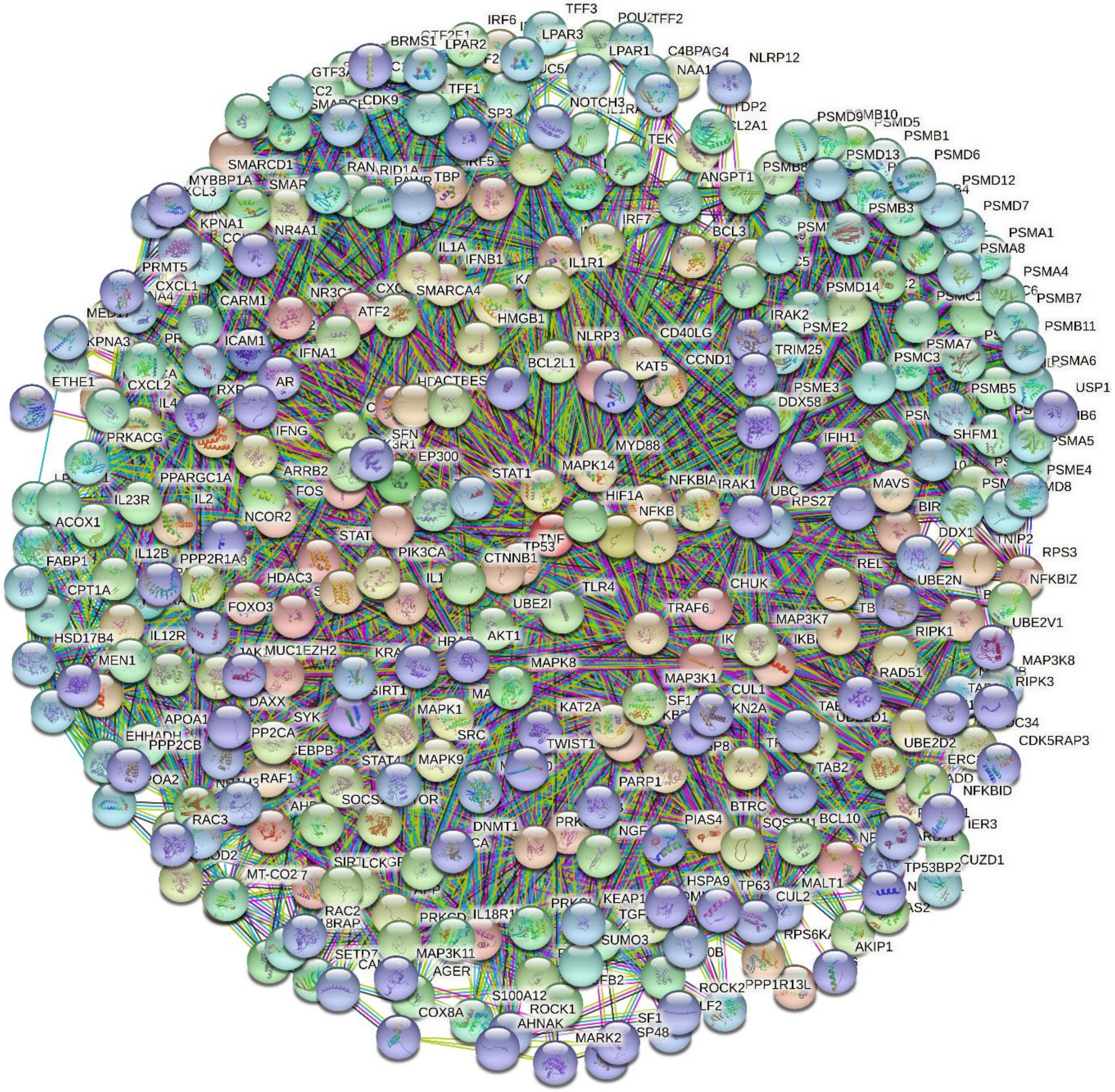
IRF6
TFF3
TFF2
BRMS1
LPAR2
LPAR3
LPAR1
C4BPA
NLRP12
GTF3A
CDK9
TFF1
SP3
NOTCH3
NAA1
TDP2
TEK
ANGPT1
PSMD9
PSMB10
PSMD5
PSMB1
PSMD6
PSMD13
PSMB8
PSMD12
PSMD7
PSMB3
PSMA1
PSMA8
PSMA4
PSMB7
PSMB11
PSMA6
USP1
PSMA5
PSME4
SMARCD1
MYBBP1A
RAN
TBP
IRF5
IRF7
BCL3
KPNA1
NR4A1
IL1A
IFNB1
IL1R1
PRMT5
CXCL1
CARM1
NR3C1
SMARCA4
HMGB1
PSMD14
IRAK2
PSME2
PSMC1
PSMA7
PSMC3
PSMB5
MED1
KPNA3
ICAM1
IFNA1
ATF2
NLRP3
CD40LG
BCL2L1
KAT5
CCND1
TRIM25
PSME3
DDX58
ETHE1
CXCL2
AR
IFNG
SFN
EP300
MYD88
IFIH1
SHFM1
PRKACG
PPARGC1A
ARRB2
STAT1
MAPK14
NFKBIA
IRAK1
UBC
RPS27
MAVS
IL23R
IL2
FOS
HIF1A
ACOX1
NCOR2
DDX1
RPS3
FABP1
IL12B
PPP2R1A
PIK3CA
CTNNB1
TP53
TNF
REL
UBE2N
NFKBIZ
CPT1A
HDAC3
TLR4
CHUK
FOXO3
UBE2I
TRAF6
UBE2V1
HSD17B4
IL12R
AKT1
MAP3K7
RIPK1
MAP3K8
MEN1
EZH2
MAPK8
RAD51
RIPK3
DAXX
SIRT1
MAP3K1
CUL1
APOA1
SYK
MAPK1
KAT2A
SF1
EHHADH
PP2CA
SRC
PPP2CB
CEBPB
STAT4
MAPK9
TWIST1
UBE2D2
CDK5RAP3
RAF1
PARP1
TAB2
NFKBID
RAC3
SOCS1
IER3
DNMT1
PIAS4
BTRC
BCL10
CUZD1
MT-CO2
HSPA9
TP63
MALT1
TP53BP2
RAC2
IL18R1
KEAP1
CUL2
PRKCD
SUMO3
RPS6KA
AKIP1
SETD7
MAP3K11
AGER
ROCK2
PPP1R13L
S100A12
ROCK1
COX8A
AHNAK
SF1
MARK2

F

minimum required interaction score: high confidence (0.700)
number of nodes: 323
number of edges: 5565
average node degree: 34.5
avg. local clustering coefficient: 0.661
expected number of edges: 2037
PPI enrichment p-value: < 1.0e-16

G

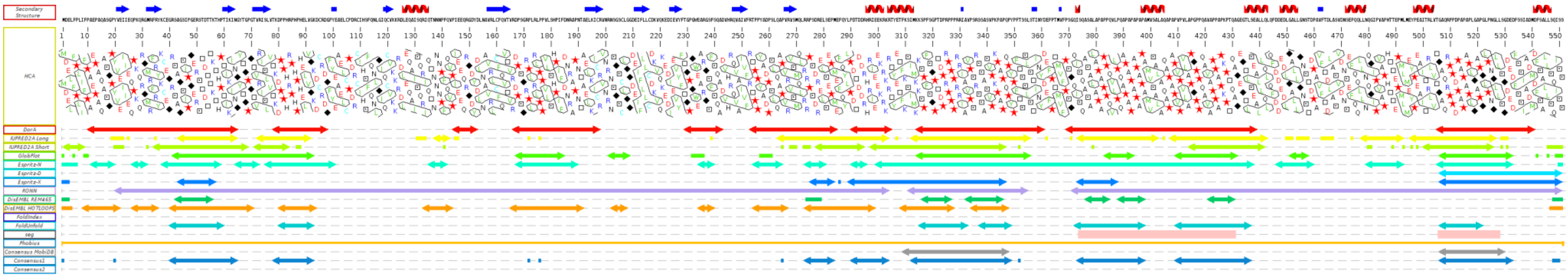

Secondary Structure
HCA
DorA
IUPRED2A Long
IUPRED2A Short
GlobPlot
Espritz-N
Espritz-D
Espritz-X
RONN
DisEMBL REM465
DisEMBL HOTLOOPS
FoldIndex
FoldUnfold
seg
Phobius
Consensus MobiDB
Consensus1
Consensus2

H

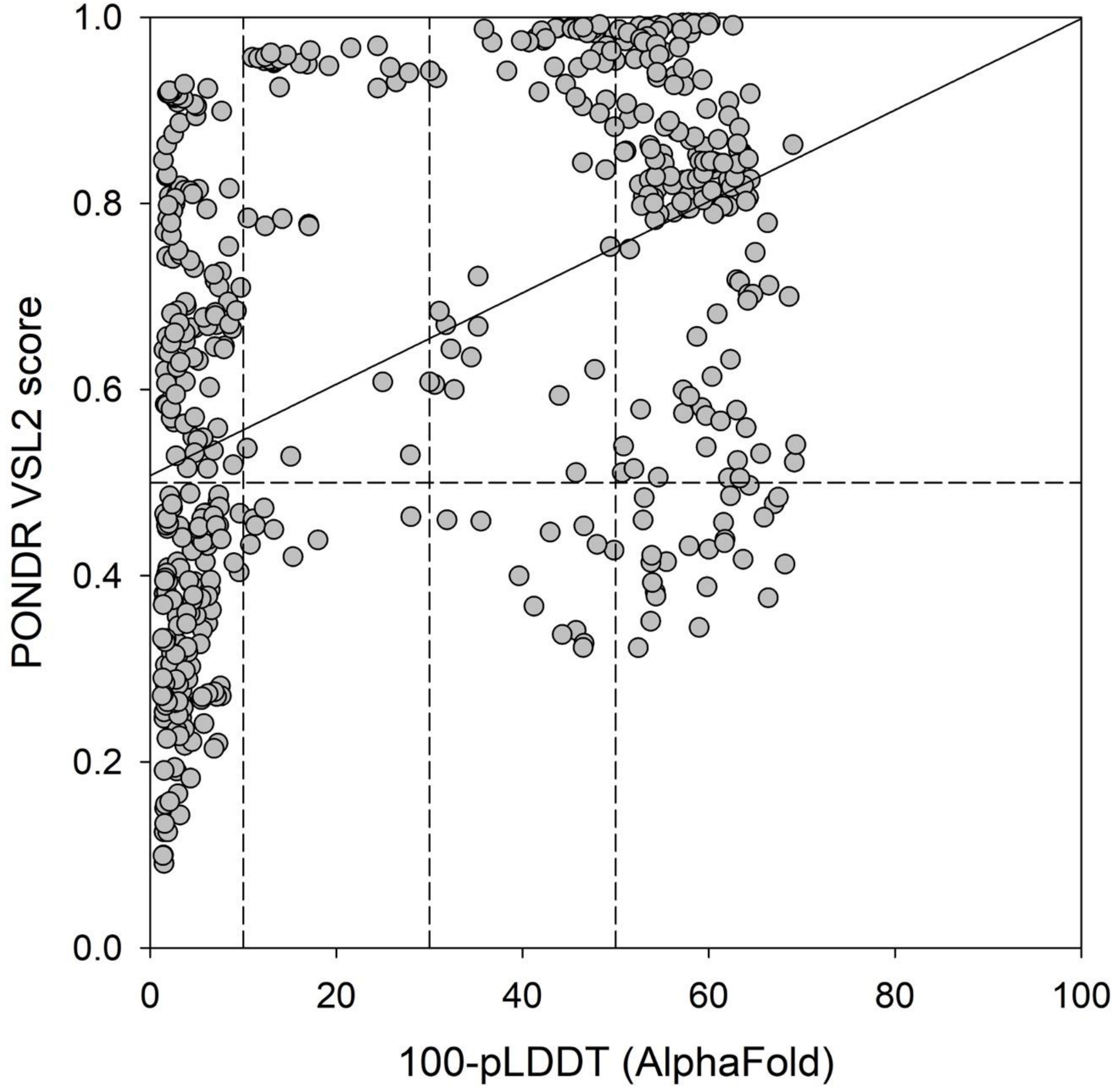
1.0
0.8
0.6
0.4
0.2
0.0
0
20
40
60
80
100
PONDR VSL2 score
100-pLDDT (AlphaFold)

Supplementary Figure S9. Functional disorder in fibrillarin 2. **A.** Amino acid sequence. **B.** RIDAO-generated disorder profile. **C.** D2P2-generated functional disorder profile. **D.** FuzDrop output. **E.** AlphaFold2 structure with FuzDrop annotations. **F.** STRING-generated PPI network with corresponding statistics. **G.** MeDor output. **H.** Plot of the *per* residue disorder score (as provided by PONDR® VLS2) versus (100-pLDDT), where pLDDT is the corresponding AlphaFold2 confidence score.

A

>tr|B7VCB9|B7VCB9_NICBE Fibrillarin 2 OS=Nicotiana benthamiana OX=4100 GN=fib2 PE=2 SV=1
MVAPTRGRGGGGFRGGRGDGGGRGGRGGRGGFGGGRGGGGSAMKRGGGRGGGGRGGGGRGGGRGGRGGGFKGGNKVVVEPHRHGGVFIAKGKEDALCTKNLVPGEAVYNEKRISVQNED
GTKVEYRVWNPFRSKLAAAVLGGVDDIWIKPGAKVLYLGAASGTTVSHVSDLVGPGGVVYAVEFSHRSGRDLVNMAKKRTNVIPIIEDARHPAKYRMLVGMVDVIFSDVAQPDQARILA
LNASYFLKAGGHFVISIKANCIDSTVPAEAVFAQEVKKLQAEQFKPMEQVTLEPFERDHACVVGAYRVPKKQKAAA

B

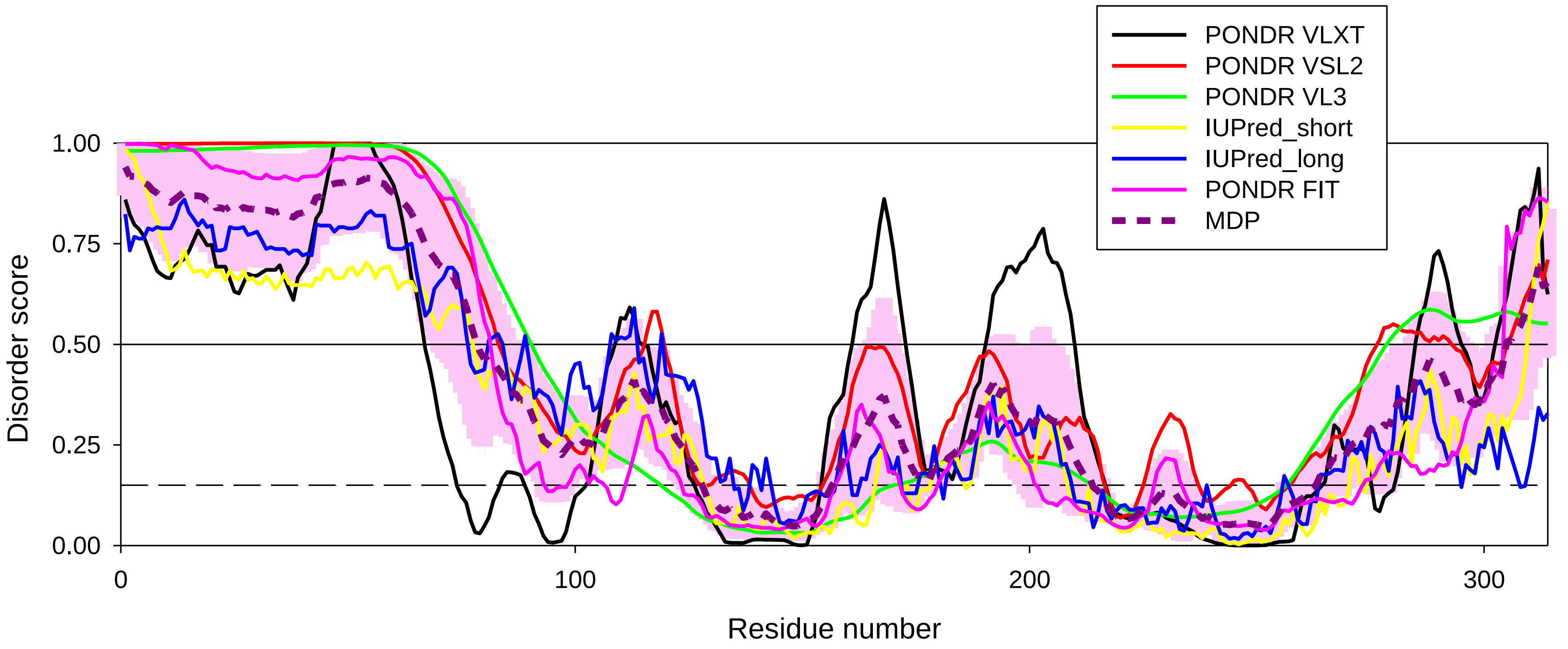

C

D^2P^2 does not have data for Fibrillarin 2 and functional disorder profile cannot be generated

D

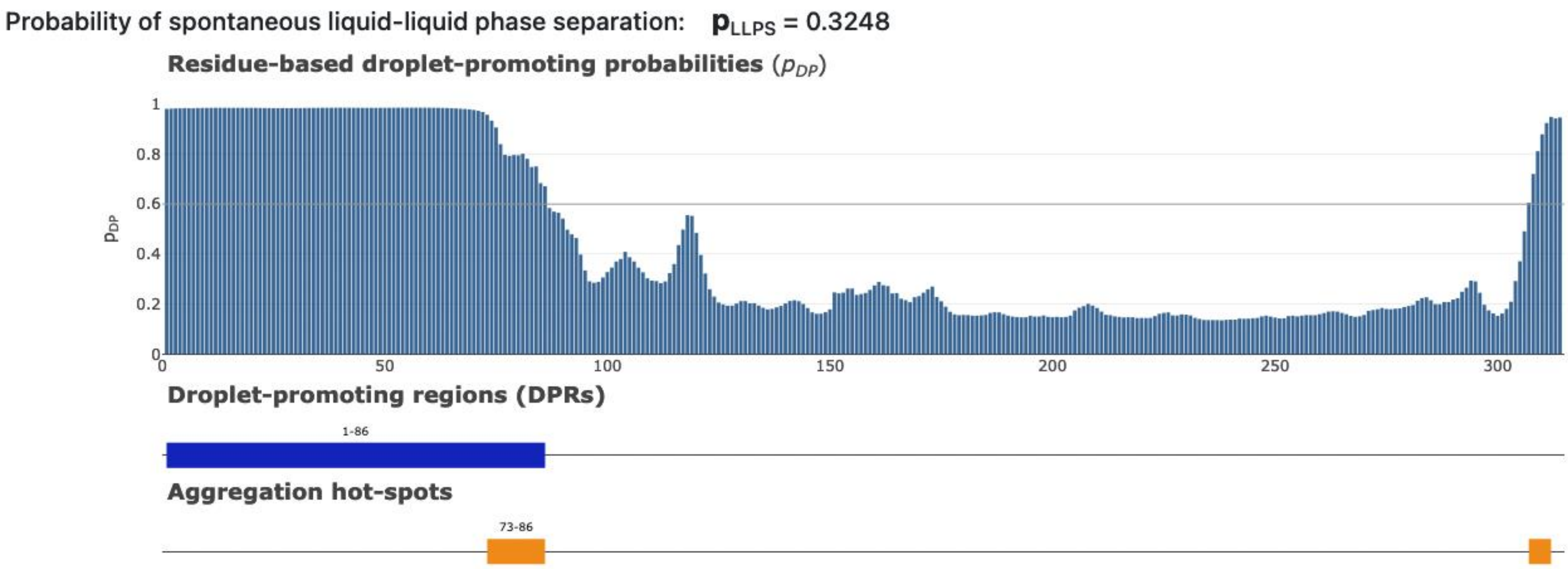

E

There is no AlphaFold modeled structure for Fibrillarin 2

F

STRING-based protein-protein interaction network centered on Fibrillarin 2 is not available

G

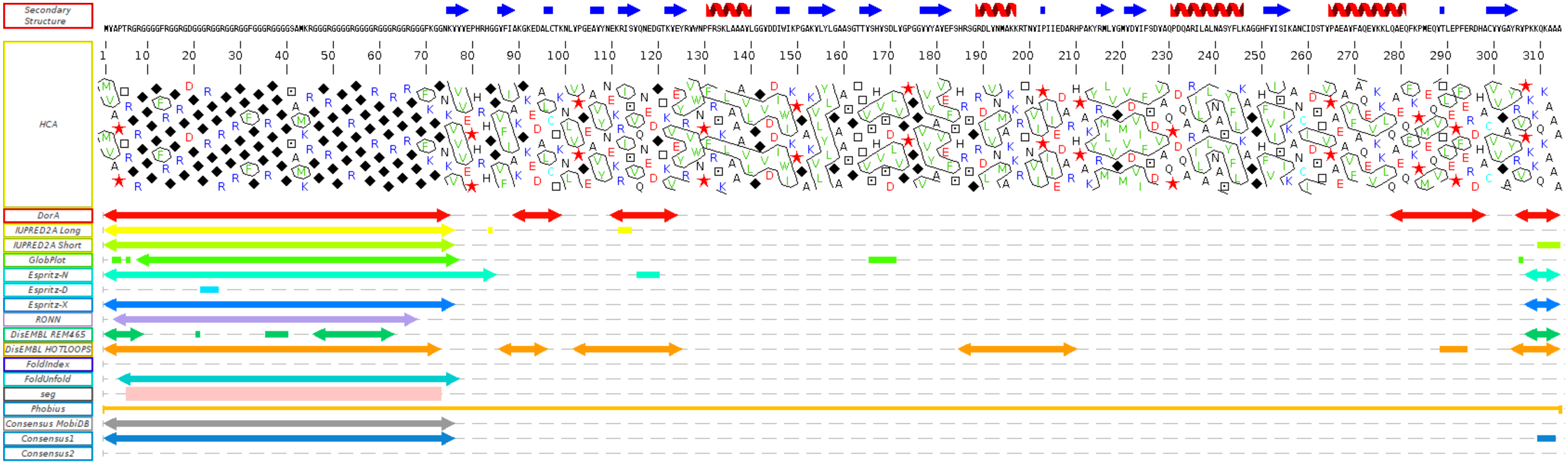

Secondary Structure
HCA
DorA
IUPRED2A Long
IUPRED2A Short
GlobPlot
Espritz-N
Espritz-D
Espritz-X
RONN
DisEMBL REM465
DisEMBL HOTLOOPS
FoldIndex
FoldUnfold
seg
Phobius
Consensus MobiDB
Consensus1
Consensus2
1 10 20 30 40 50 60 70 80 90 100 110 120 130 140 150 160 170 180 190 200 210 220 230 240 250 260 270 280 290 300 310

H

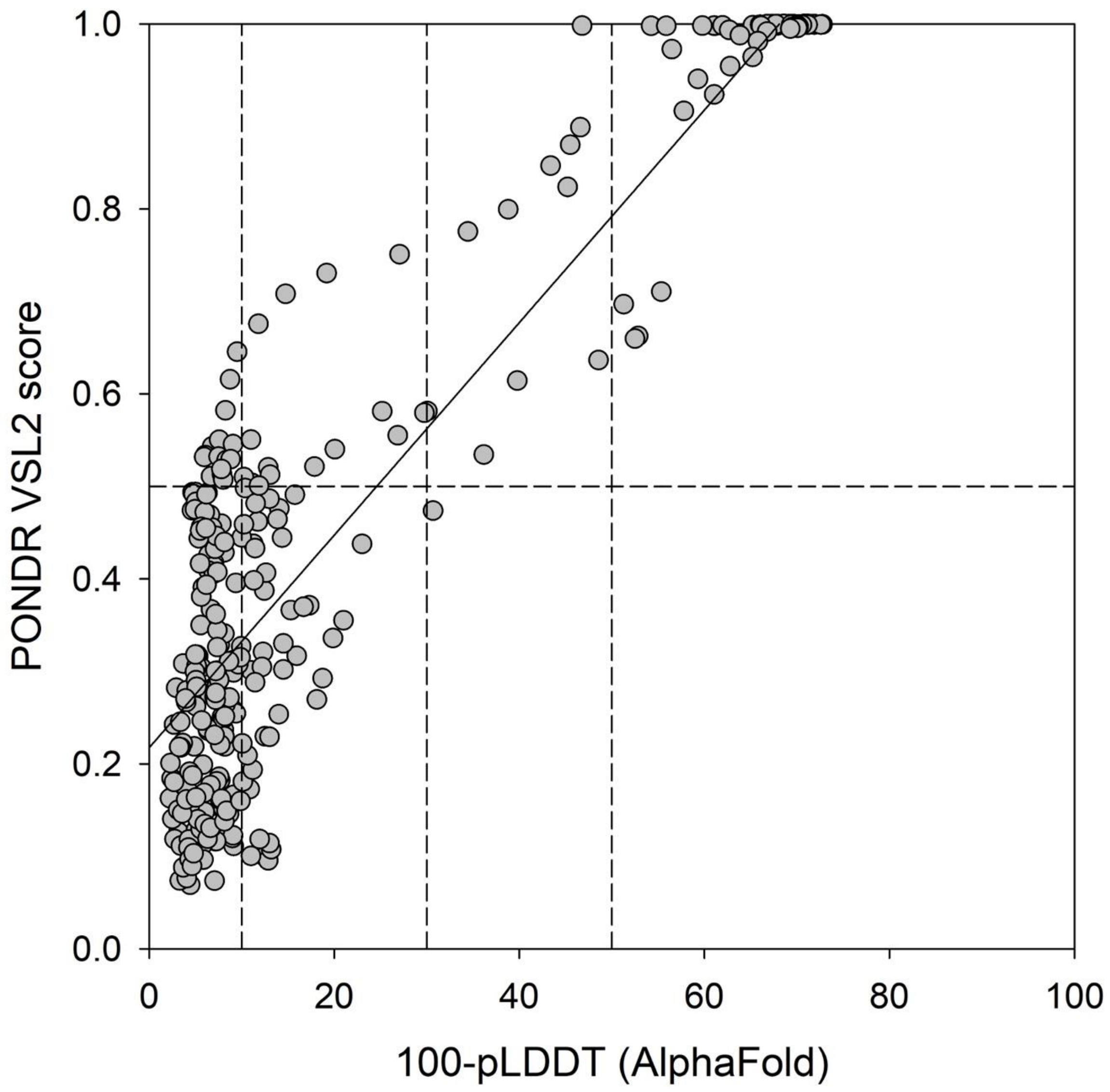

1.0
0.8
0.6
0.4
0.2
0.0
0
20
40
60
80
100
PONDR VSL2 score
100-pLDDT (AlphaFold)

Supplementary Figure S10. Functional disorder in nuclear transport factor 2 (NTF2) family protein with RNA binding (RRM-RBD-RNP motifs) domain-containing protein. **A.** Amino acid sequence. **B.** RIDAO-generated disorder profile. **C.** D2P2-generated functional disorder profile. **D.** FuzDrop output. **E.** AlphaFold2 structure with FuzDrop annotations. **F.** STRING-generated PPI network with corresponding statistics. **G.** MeDor output. **H.** Plot of the *per* residue disorder score (as provided by PONDR® VLS2) versus (100-pLDDT), where pLDDT is the corresponding AlphaFold2 confidence score.

A

>tr|Q84JH2|Q84JH2_ARATH Nuclear transport factor 2 (NTF2) family protein with RNA binding (RRM-RBD-RNP motifs) domain-containing protein OS=Arabidopsis thaliana OX=3702 GN=At5g48650 PE=1 SV=1
MDSTAATKRVVDPLTVGSAFVNQYYYIFCNMPEHLPRFYQEISRVGRVGQDGVMRDFSTFQGISEELKRLTYGDCNSAEITSYDTQESHNGGFLLFVTGYFTLNERSRRKFTQTFFLA
PQEKGFFVLNDILRFVNDDAKDNVPETIDGEVVSGINSTTPTIINGMKGSEQAACVSVNPVCKEVSKPLDNENAKDNVLVPEIANEVARTEITCKEVADDSQKNYDPDDGLADAPKKS
YASVLKVTKDKFGVPAVSLPSPKKIPKDQEHQAPSDPSTGQILKDQGQQASSDPSQVIESDTVSESVDASENGHNQEAVAEGTSIYVRHLPFNANIDMLEAEFKQFGAITNGGIQVIN
QRGLGYPYGFVEFEEADAAHRAIEASPVKIGGLRAFVEEKLSTSRGKRGNGNVGYGNRNVGVGMRGRGSYGYGYDYRRGGRGPGGGGRSFNRRGNEYVASINSY

B

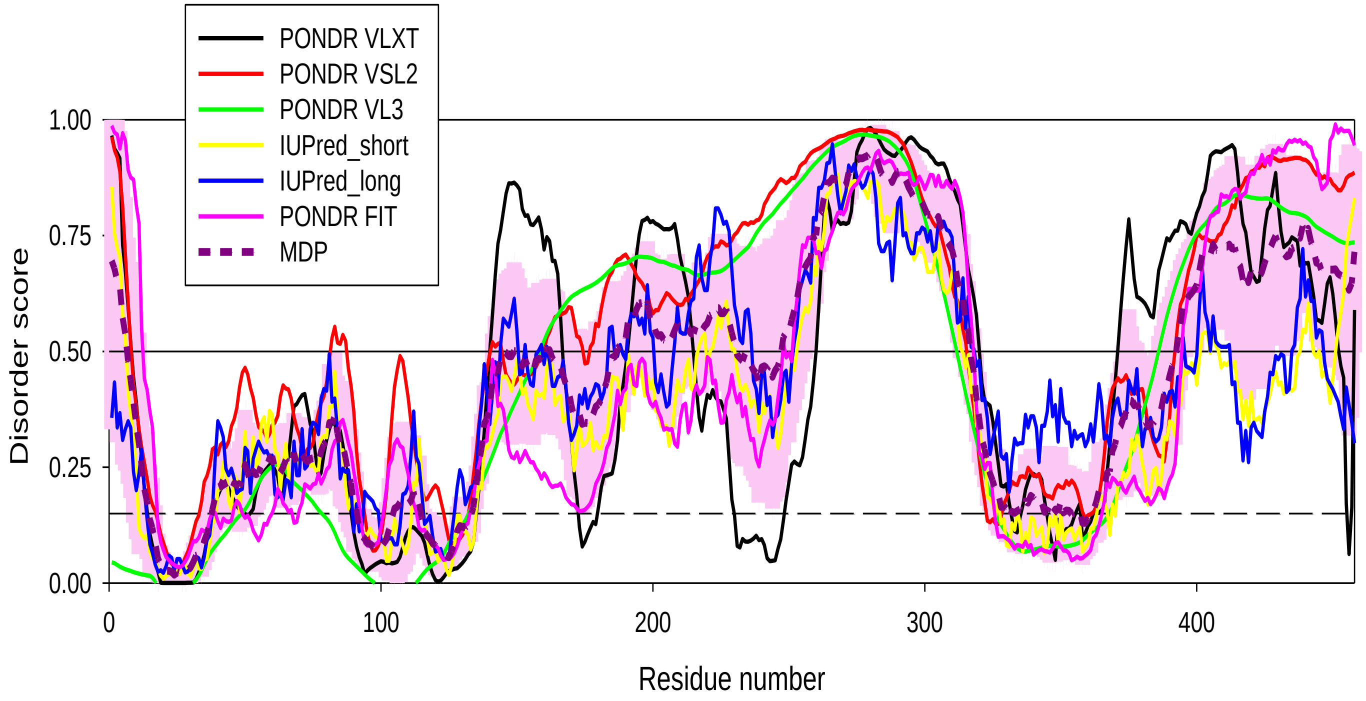

C

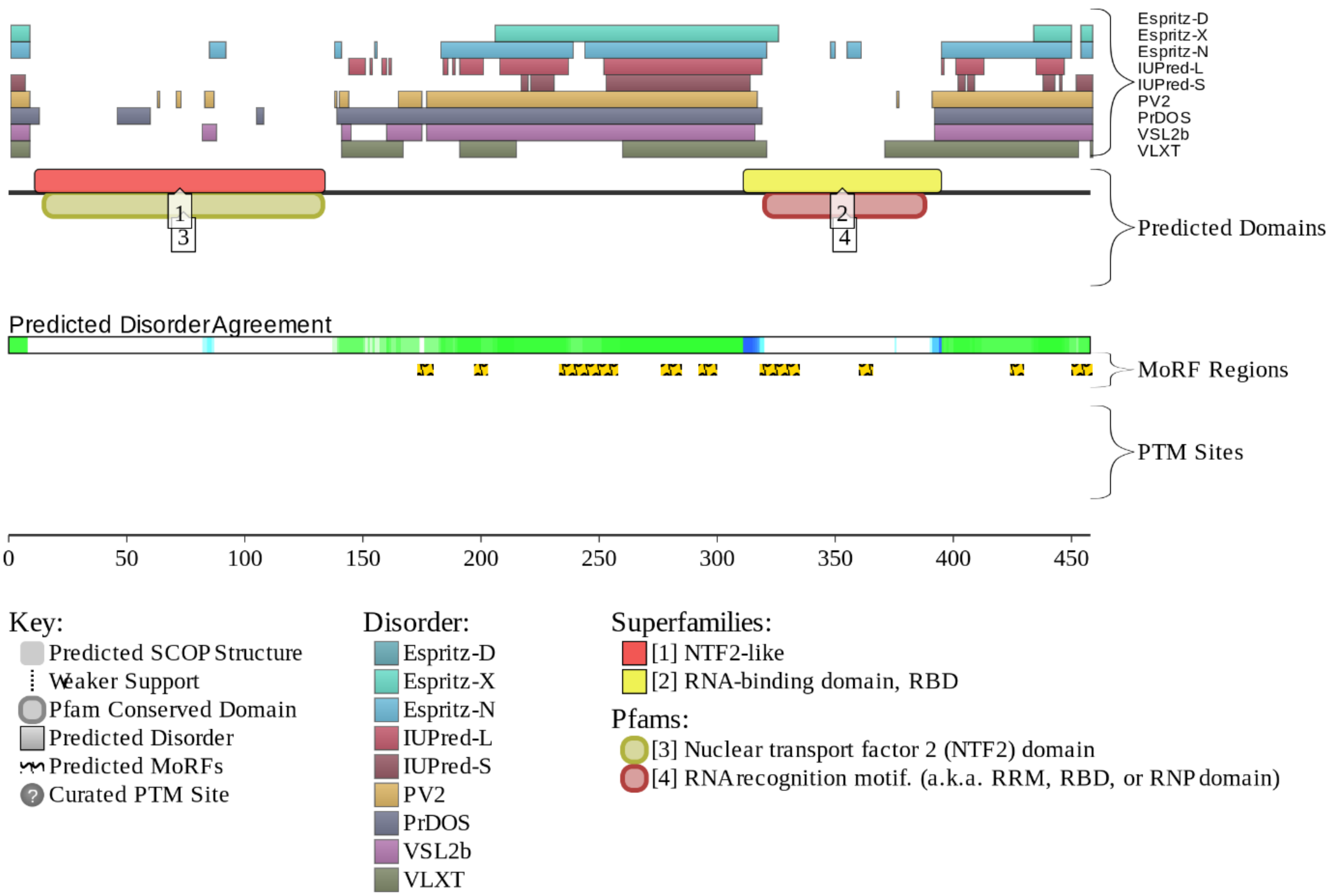
Espritz-D
Espritz-X
Espritz-N
IUPred-L
IUPred-S
PV2
PrDOS
VSL2b
VLXT
Predicted Domains
1
3
2
4
Predicted Disorder Agreement
MoRF Regions
PTM Sites
0
50
100
150
200
250
300
350
400
450
Key:
Predicted SCOP Structure
Weaker Support
Pfam Conserved Domain
Predicted Disorder
Predicted MoRFs
Curated PTM Site
Disorder:
Espritz-D
Espritz-X
Espritz-N
IUPred-L
IUPred-S
PV2
PrDOS
VSL2b
VLXT
Superfamilies:
[1] NTF2-like
[2] RNA-binding domain, RBD
Pfams:
[3] Nuclear transport factor 2 (NTF2) domain
[4] RNA recognition motif. (a.k.a. RRM, RBD, or RNP domain)

D

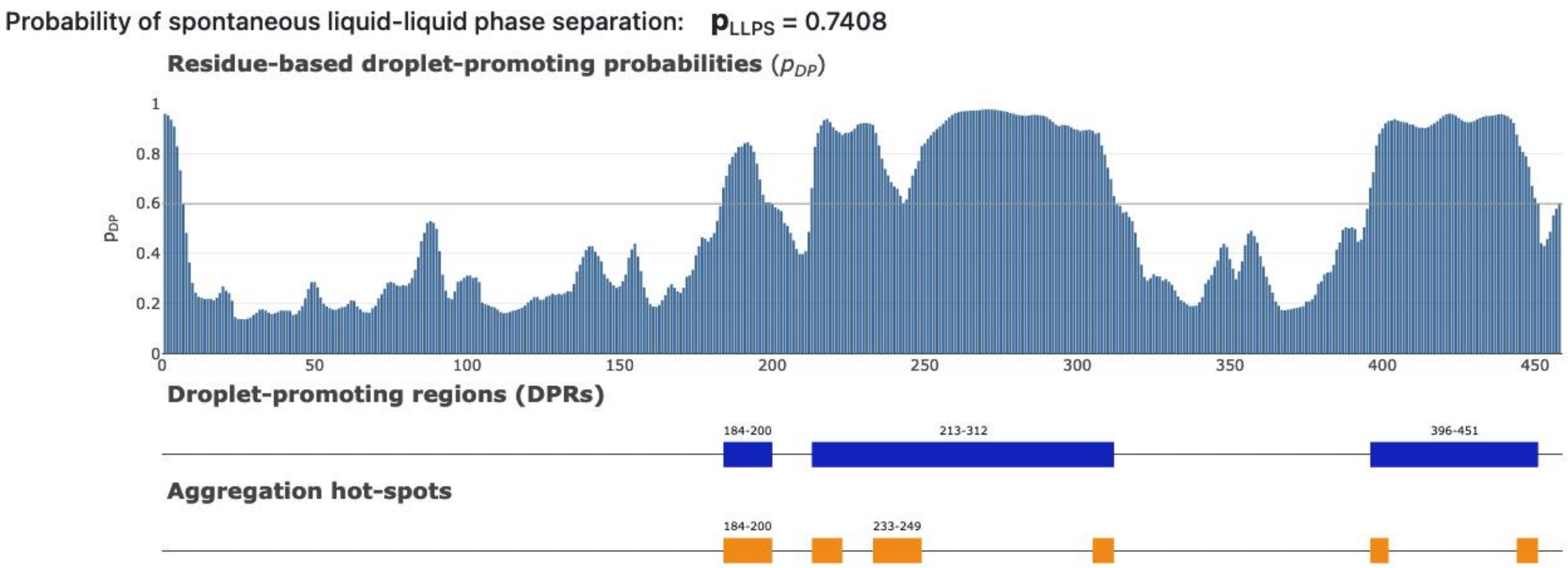

E

There is no AlphaFold2-generated structure for this protein at FuzDrop

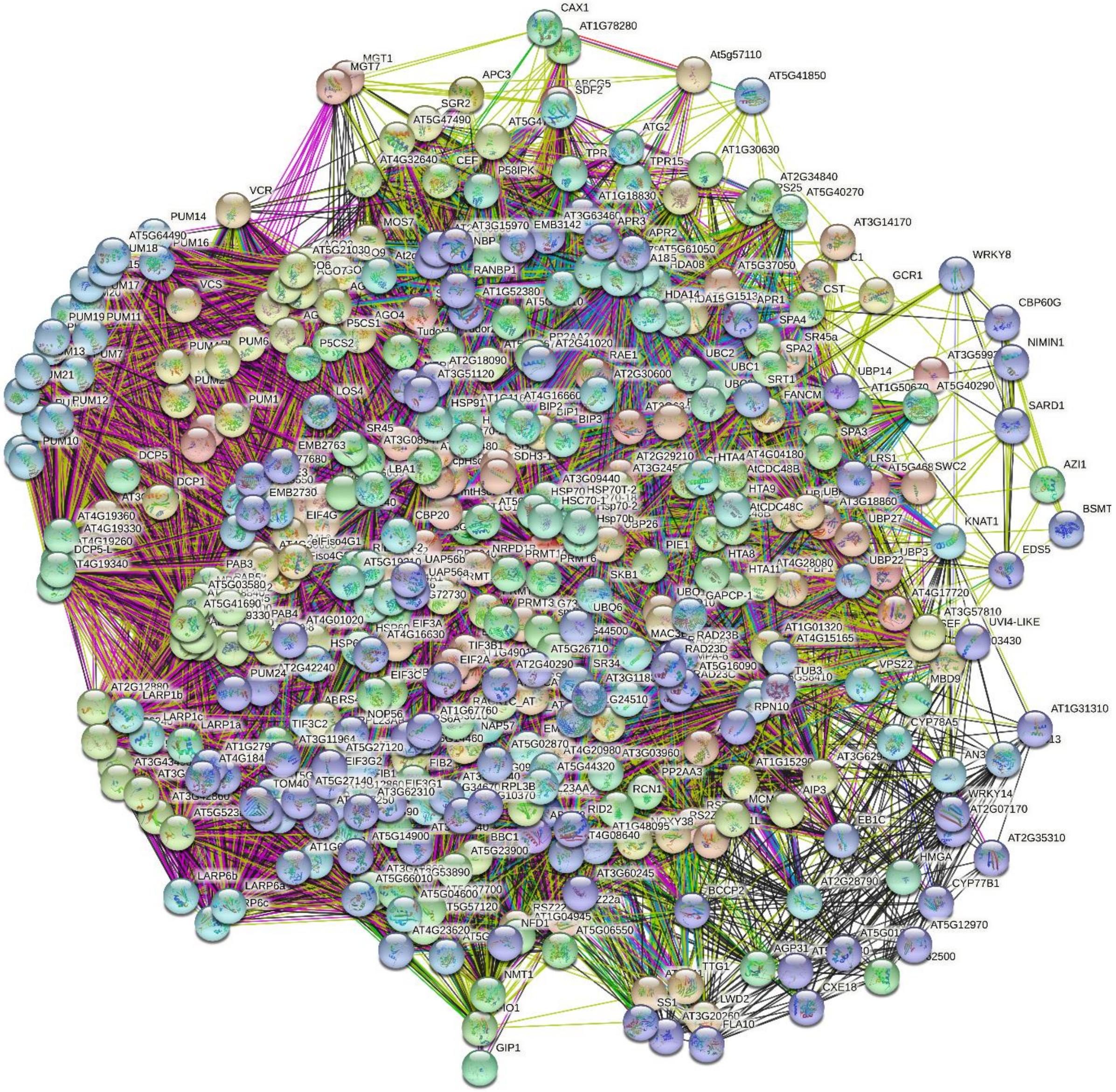
CAX1
AT1G78280
MGT1
MGT7
APC3
At5g57110
AT5G41850
SDF2
SGR2
AT5G47490
ATG2
TPR
TPR15
AT1G30630
AT4G32640
CEF
P58IPK
AT2G34840
AT5G40270
AT1G18830
VCR
PUM14
AT3G15970
EMB3142
AT3G63460
APR3
APR2
AT3G14170
AT5G64490
PUM18
PUM16
MOS7
AT5G21030
RANBP1
AT5G61050
HDA08
AT5G37050
WRKY8
GCR1
CST
PUM17
VCS
AT1G52380
HDA14
HDA15
APR1
CBP60G
PUM19
PUM11
P5CS1
AGO4
SPA4
SR45a
NIMIN1
PUM13
PUM7
PUM6
P5CS2
AT2G41020
RAE1
UBC2
SPA2
UBP14
AT5G40290
PUM21
AT2G18090
AT3G51120
AT2G30600
UBC1
SRT1
AT1G50670
SARD1
PUM12
PUM1
LOS4
HSP91
AT4G16660
BIP2
FANCM
BIP3
PUM10
SR45
SDH3-1
SPA3
DCP5
EMB2763
LBA1
AT2G29210
HTA4
AT4G04180
LRS1
SWC2
AZI1
DCP1
AT3G09440
AtCDC48B
EMB2730
HSP70
HSP70T-2
HTA9
AT3G18860
BSMT
AT4G19360
AT4G19330
EIF4G
CBP20
AtCDC48C
UBP27
KNAT1
AT4G19260
DCP5-L
PIE1
UBP3
EDS5
AT4G19340
UAP56b
NRPD1B
PRMT1
HTA8
AT4G28080
UBP22
PAB3
AT5G03580
UAP56a
SKB1
HTA11
GAPCP-1
AT4G17720
AT5G41690
PAB4
PRMT3
UBQ6
AT3G57810
UVI4-LIKE
AT4G01020
EIF3A
MAC3B
RAD23B
AT1G01320
AT4G16630
AT4G15165
TIF3B1
AT5G26710
RAD23D
EIF2A
AT5G16090
TUB3
PUM24
AT2G42240
EIF3C
AT2G40290
VPS22
AT2G12880
LARP1b
MBD9
RPN10
AT1G31310
LARP1c
LARP1a
NOP56
AT1G67760
CYP78A5
TIF3C2
NAP57
AT3G11964
AT5G02870
AT4G20980
AT3G03960
AT5G27120
EIF3G2
FIB2
AT5G44320
PP2AA3
AT1G15290
AIP3
TOM40
AT5G27140
FIB1
AT3G62310
RPL3B
RID2
RCN1
MCM
WRKY14
AT2G07170
AT5G14900
BBC1
AT4G08640
AT1G48095
EB1C
AT2G35310
AT5G23900
HMGA
AT5G66010
AT3G60245
AT2G28790
CYP77B1
LARP6b
LARP6a
AT5G04600
BCCP2
AT5G57120
RSZ22
AT1G04945
AT5G12970
AT4G23620
NFD1
AT5G06550
AGP31
CXE18
TTG1
NMT1
SS1
LWD2
AT3G20260
FLA10
GIP1

F

minimum required interaction score: low confidence (0.150)

number of nodes: 411

number of edges: 27495

average node degree: 134

avg. local clustering coefficient: 0.611

expected number of edges: 13087

PPI enrichment p-value: < 1.0e-16

G

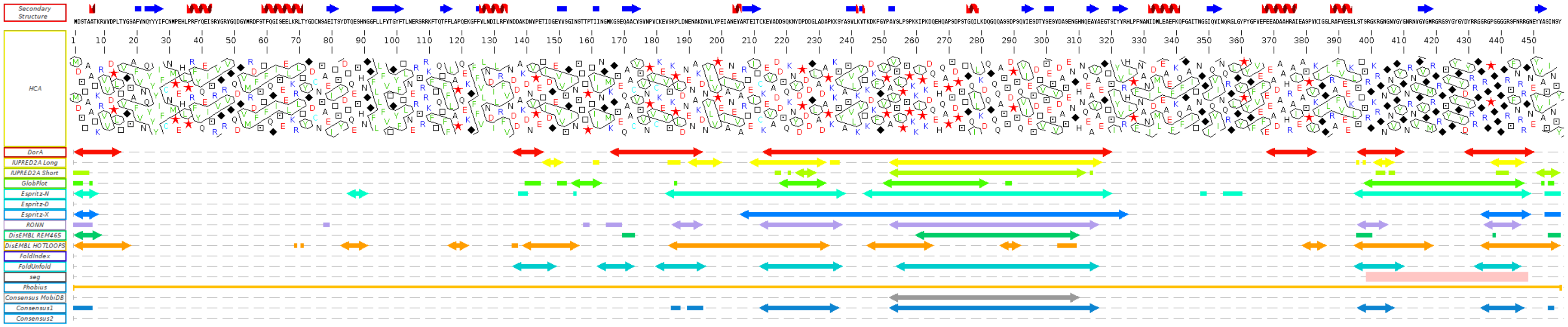
Secondary Structure
HCA
DorA
IUPRED2A Long
IUPRED2A Short
GlobPlot
Espritz-N
Espritz-D
Espritz-X
RONN
DisEMBL REM465
DisEMBL HOTLOOPS
FoldIndex
FoldUnfold
seg
Phobius
Consensus MobiDB
Consensus1
Consensus2

H

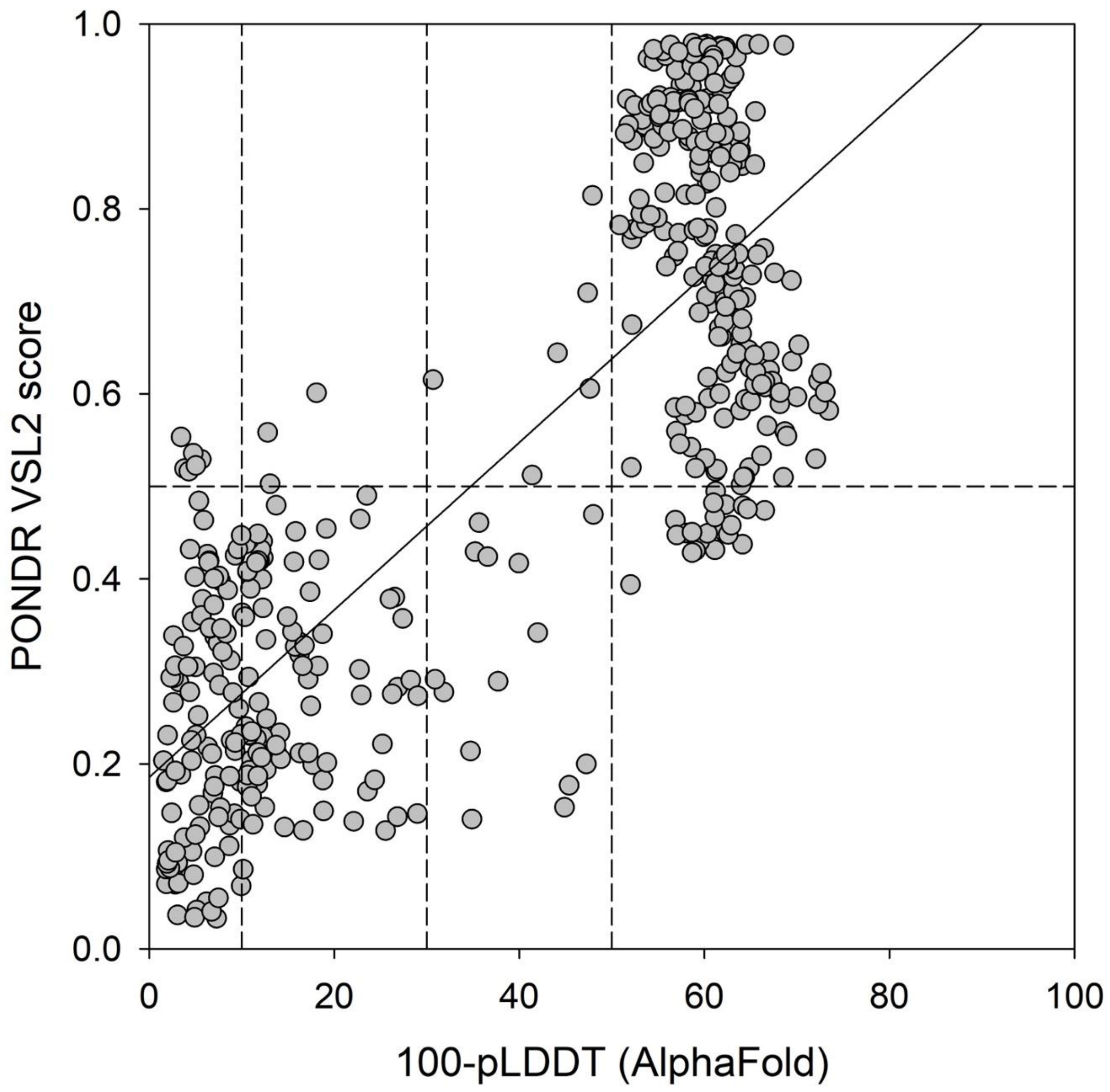

1.0
0.8
0.6
0.4
0.2
0.0
0
20
40
60
80
100
PONDR VSL2 score
100-pLDDT (AlphaFold)

Supplementary Figure S11. Functional disorder in mitogen-activated protein kinase 14. **A.** Amino acid sequence. **B.** RIDAO-generated disorder profile. **C.** D2P2-generated functional disorder profile. **D.** FuzDrop output. **E.** AlphaFold2 structure with FuzDrop annotations. **F.** STRING-generated PPI network with corresponding statistics. **G.** MeDor output. **H.** Plot of the *per* residue disorder score (as provided by PONDR® VLS2) versus (100-pLDDT), where pLDDT is the corresponding AlphaFold2 confidence score.

A

>sp|Q16539|MK14_HUMAN Mitogen-activated protein kinase 14 OS=Homo sapiens OX=9606 GN=MAPK14 PE=1 SV=3
MSQERPTFYRQELNKTIWEVPERYQNLSPVGSGAYGSVCAAFDTKTGLRVAVKKLSRPFQSIIHAKRTYRELRLLKHMKHENVIGLLDVFTPARSLEEFNDVYLVTHLMGADLNNIVK
CQKLTDDHVQFLIYQILRGLKYIHSADIIHRDLKPSNLAVNEDCELKILDFGLARHTDDEMTGYVATRWYRAPEIMLNWMHYNQTVDIWSVGCIMAELLTGRTLFPGTDHIDQLKLIL
RLVGTPGAELLKKISSESARNYIQSLTQMPKMNFANVFIGANPLAVDLLEKMLVLDSDKRITAAQALAHAYFAQYHDPDDEPVADPYDQSFESRDLLIDEWKSLTYDEVISFVPPPLD
QEEMES

B

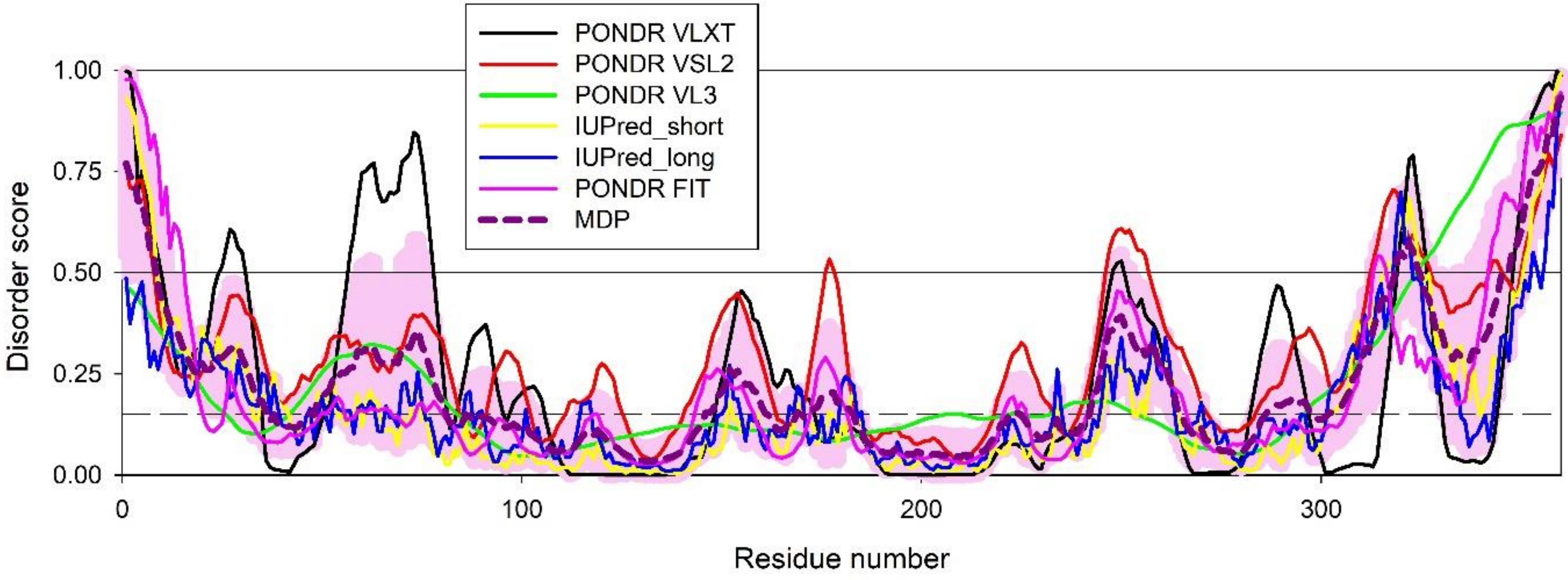

C

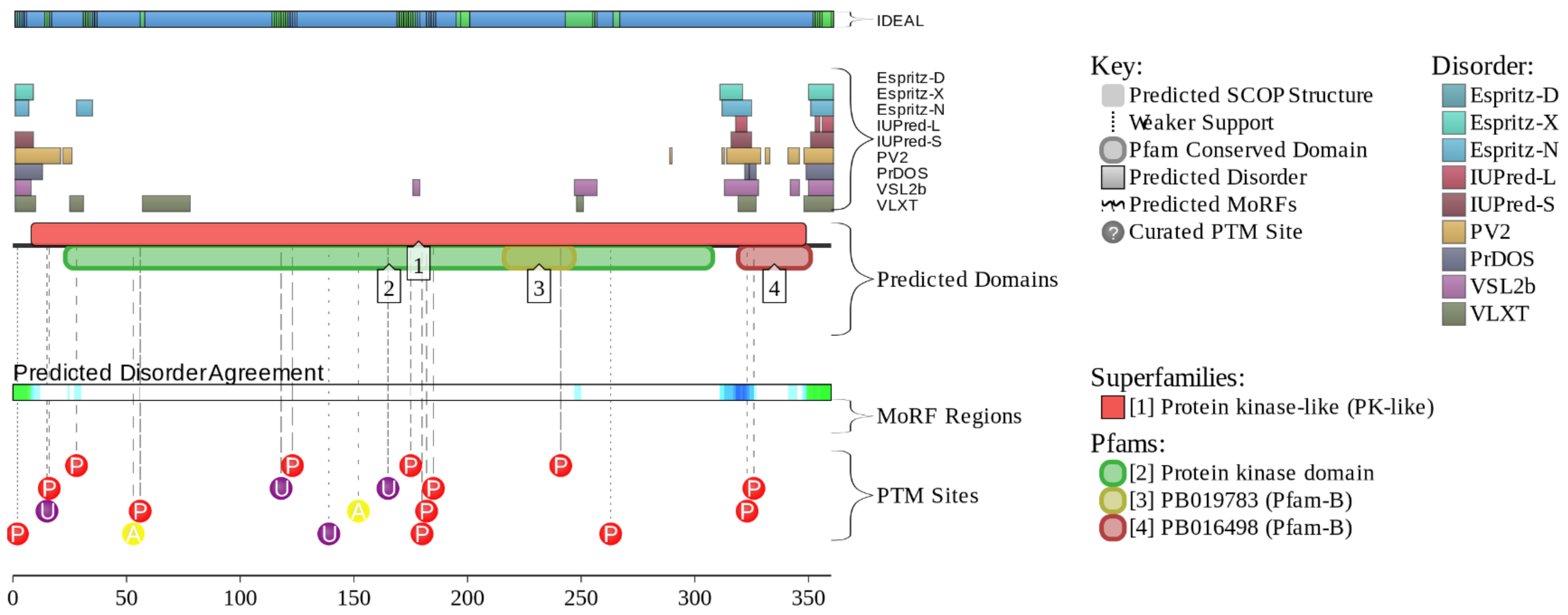
IDEAL
Espritz-D
Espritz-X
Espritz-N
IUPred-L
IUPred-S
PV2
PrDOS
VSL2b
VLXT
Predicted Domains
Predicted Disorder Agreement
MoRF Regions
PTM Sites
0
50
100
150
200
250
300
350
Key:
Predicted SCOP Structure
Weaker Support
Pfam Conserved Domain
Predicted Disorder
Predicted MoRFs
Curated PTM Site
Disorder:
Espritz-D
Espritz-X
Espritz-N
IUPred-L
IUPred-S
PV2
PrDOS
VSL2b
VLXT
Superfamilies:
[1] Protein kinase-like (PK-like)
Pfams:
[2] Protein kinase domain
[3] PB019783 (Pfam-B)
[4] PB016498 (Pfam-B)

D

Probability of spontaneous liquid-liquid phase separation: $\mathbf{p}_{LLPS}$ = 0.1119

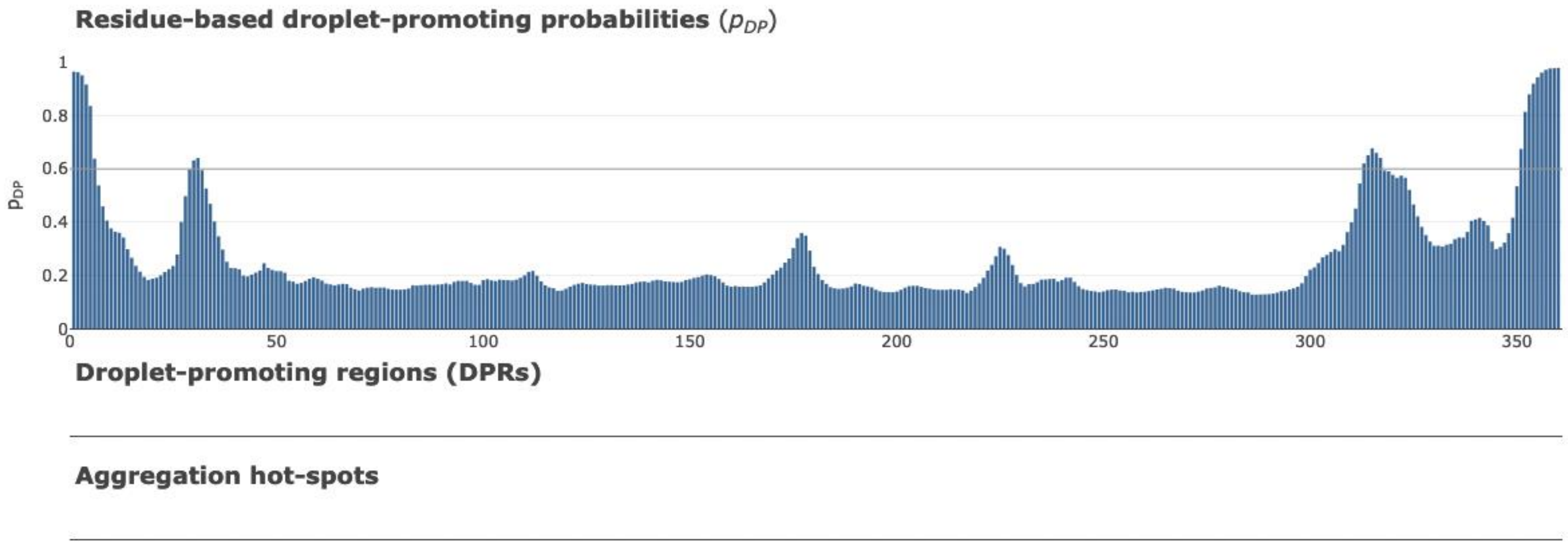

E

There is no AlphaFold2 generated 3D structure for this protein at FuzDrop

F

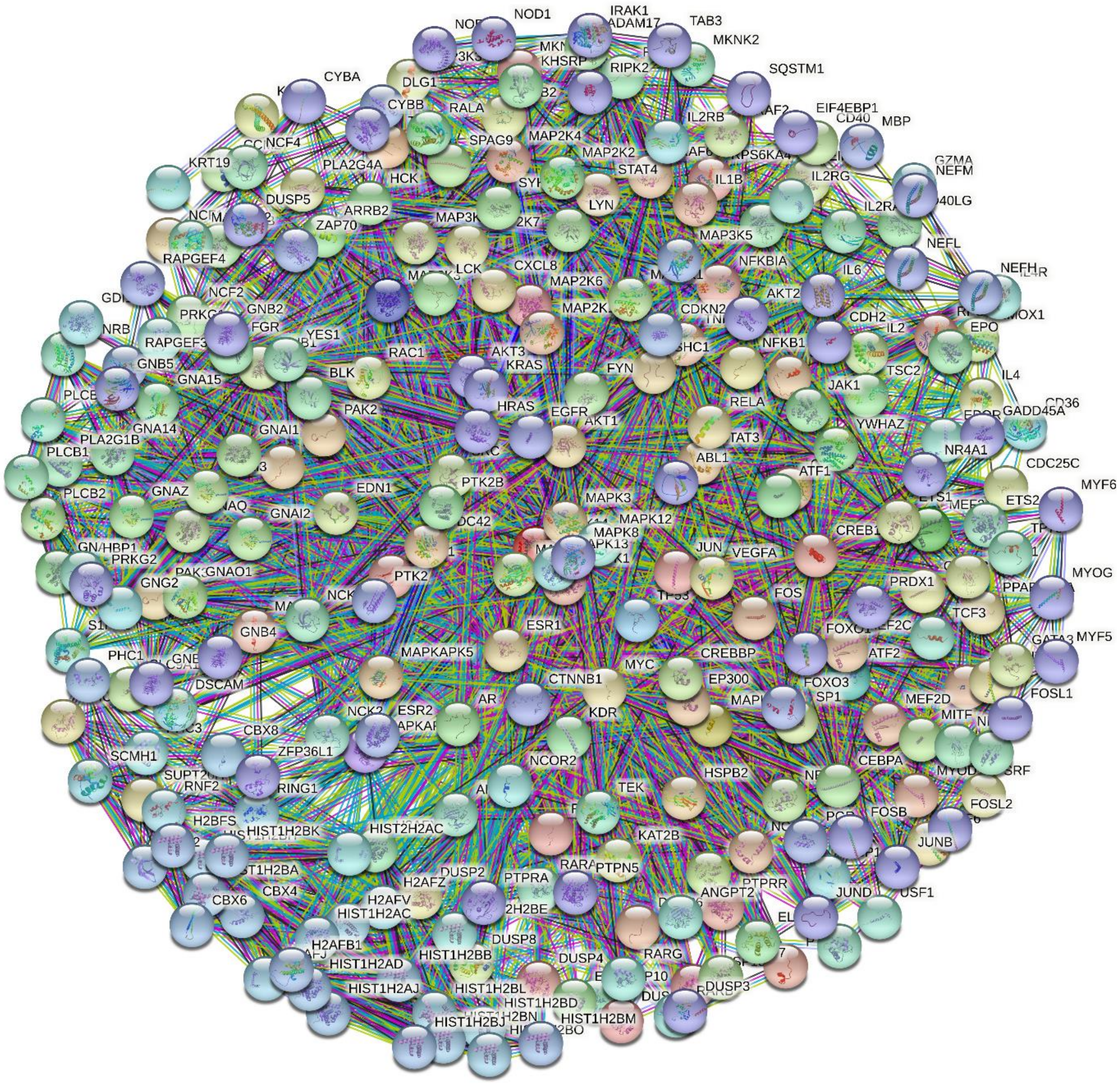

NOD1
IRAK1
ADAM17
TAB3
MKNK2
SQSTM1
KHSRP
RIPK2
CYBA
DLG1
CYBB
RALA
EIF4EBP1
CD40
MBP
SPAG9
MAP2K4
IL2RB
NCF4
PLA2G4A
MAP2K2
STAT4
IL1B
IL2RG
GZMA
NEFM
KRT19
HCK
LYN
IL2RA
DUSP5
ARRB2
ZAP70
MAP3K5
NEFL
RAPGEF4
LCK
CXCL8
NFKBIA
IL6
NEFH
NCF2
MAP2K6
AKT2
GNB2
CDKN2
CDH2
MOX1
RAPGEF3
FGR
YES1
RAC1
AKT3
SHC1
NFKB1
IL2
EPO
NRB
GNB5
KRAS
FYN
JAK1
TSC2
IL4
BLK
GNA15
PAK2
HRAS
EGFR
AKT1
RELA
GADD45A
CD36
PLA2G1B
GNA14
GNAI1
STAT3
YWHAZ
PLCB1
ABL1
NR4A1
CDC25C
PLCB2
GNAZ
EDN1
PTK2B
ATF1
MYF6
GNAI2
MAPK3
ETS2
MAPK12
CREB1
MAPK8
MAPK13
JUN
VEGFA
PRKG2
GNAO1
PTK2
MYOG
GNG2
FOS
PRDX1
TCF3
ESR1
GNB4
FOXO1
MYF5
MAPKAPK5
MYC
CREBBP
ATF2
PHC1
CTNNB1
EP300
FOXO3
FOSL1
DSCAM
AR
SP1
MEF2D
NCK2
ESR2
KDR
CBX8
ZFP36L1
SCMH1
NCOR2
HSPB2
CEBPA
SRF
RNF2
RING1
TEK
FOSL2
H2BFS
HIST1H2BK
HIST2H2AC
FOSB
JUNB
KAT2B
HIST1H2BA
PTPRA
RARA
PTPN5
PTPRR
CBX6
CBX4
H2AFZ
DUSP2
ANGPT2
JUND
USF1
H2AFV
HIST1H2AC
H2AFB1
HIST1H2BB
DUSP8
RARG
HIST1H2AD
DUSP4
DUSP3
HIST1H2AJ
HIST1H2BL
HIST1H2BD
HIST1H2BJ
HIST1H2BN
HIST1H2BM

minimum required interaction score: high confidence (0.700)
number of nodes: 263
number of edges: 3555
average node degree: 27
avg. local clustering coefficient: 0.621
expected number of edges: 1281
PPI enrichment p-value: < 1.0e-16

G

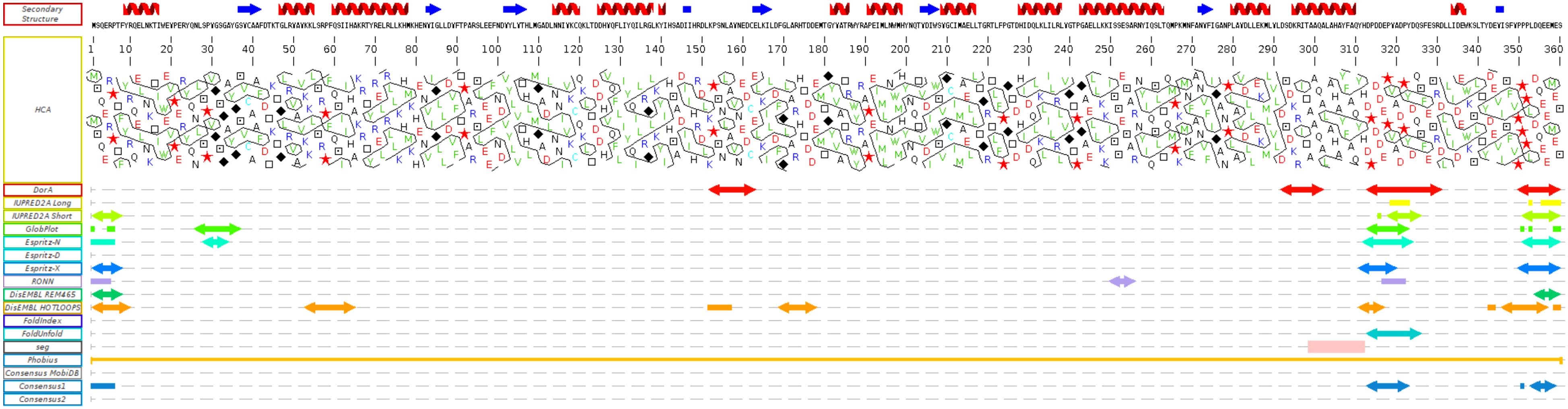
Secondary Structure
HCA
DorA
IUPRED2A Long
IUPRED2A Short
GlobPlot
Espritz-N
Espritz-D
Espritz-X
RONN
DisEMBL REM465
DisEMBL HOTLOOPS
FoldIndex
FoldUnfold
seg
Phobius
Consensus MobiDB
Consensus1
Consensus2

H

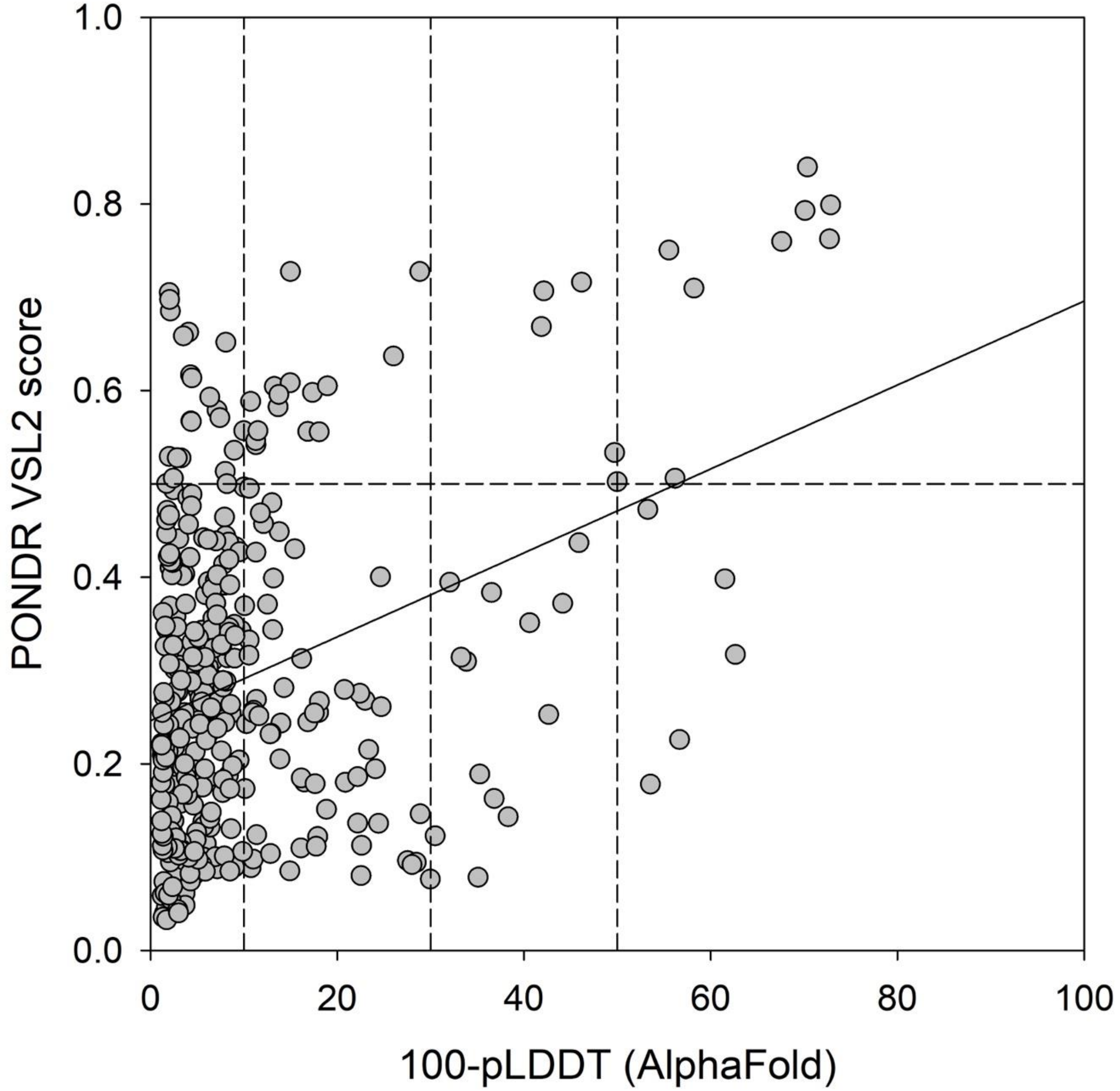

1.0
0.8
0.6
0.4
0.2
0.0
PONDR VSL2 score
0
20
40
60
80
100
100-pLDDT (AlphaFold)

Supplementary Figure S12. Functional disorder in focal adhesion kinase 1. **A.** Amino acid sequence. **B.** RIDAO-generated disorder profile. **C.** D2P2-generated functional disorder profile. **D.** FuzDrop output. **E.** AlphaFold2 structure with FuzDrop annotations. **F.** STRING-generated PPI network with corresponding statistics. **G.** MeDor output. **H.** Plot of the *per* residue disorder score (as provided by PONDR® VLS2) versus (100-pLDDT), where pLDDT is the corresponding AlphaFold2 confidence score.

A

>sp|Q05397|FAK1_HUMAN Focal adhesion kinase 1 OS=Homo sapiens OX=9606 GN=PTK2 PE=1 SV=2
MAAAYLDPNLNHTPNSSTKTHLGTGMERSPGAMERVLKVFHYFESNSEPTTWASIIRHGDATDVRGIIQKIVDSHKVKHVACYGFRLSHLRSEEVHWLHVDMGVSSVREKYELAHPPE
EWKYELRIRYLPKGFLNQFTEDKPTLNFFYQQVKSDYMLEIADQVDQEIALKLGCLEIRRSYWEMRGNALEKKSNYEVLEKDVGLKRFFPKSLLDSVKAKTLRKLIQQTFRQFANLNR
EESILKFFEILSPVYRFDKECFKCALGSSWIISVELAIGPEEGISYLTDKGCNPTHLADFTQVQTIQYSNSEDKDRKGMLQLKIAGAPEPLTVTAPSLTIAENMADLIDGYCRLVNGT
SQSFIIRPQKEGERALPSIPKLANSEKQGMRTHAVSVSETDDYAEIIDEEDTYTMPSTRDYEIQRERIELGRCIGEGQFGDVHQGIYMSPENPALAVAIKTCKNCTSDSVREKFLQEA
LTMRQFDHPHIVKLIGVITENPVWIIMELCTLGELRSFLQVRKYSLDLASLILYAYQLSTALAYLESKRFVHRDIAARNVLVSSNDCVKLGDFGLSRYMEDSTYYKASKGKLPIKWMA
PESINFRRFTSASDVWMFGVCMWEILMHGVKPFQGVKNNDVIGRIENGERLPMPPNCPPTLYSLMTKCWAYDPSRRPRFTELKAQLSTILEEEKAQQEERMRMESRRQATVSWDSGGS
DEAPPKPSRPGYPSPRSSEGFYPSPQHMVQTNHYQVSGYPGSHGITAMAGSIYPGQASLLDQTDSWNHRPQEIAMWQPNVEDSTVLDLRGIGQVLPTHLMEERLIRQQQEMEEDQRWL
EKEERFLKPDVRLSRGSIDREDGSLQGPIGNQHIYQPVGKPDPAAPPKKPPRPGAPGHLGSLASLSSPADSYNEGVKLQPQEISPPPTANLDRSNDKVYENVTGLVKAVIEMSSKIQP
APPEEYVPMVKEVGLALRTLLATVDETIPLLPASTHREIEMAQKLLNSDLGELINKMKLAQQYVMTSLQQEYKKQMLTAAHALAVDAKNLLDVIDQARLKMLGQTRPH

B

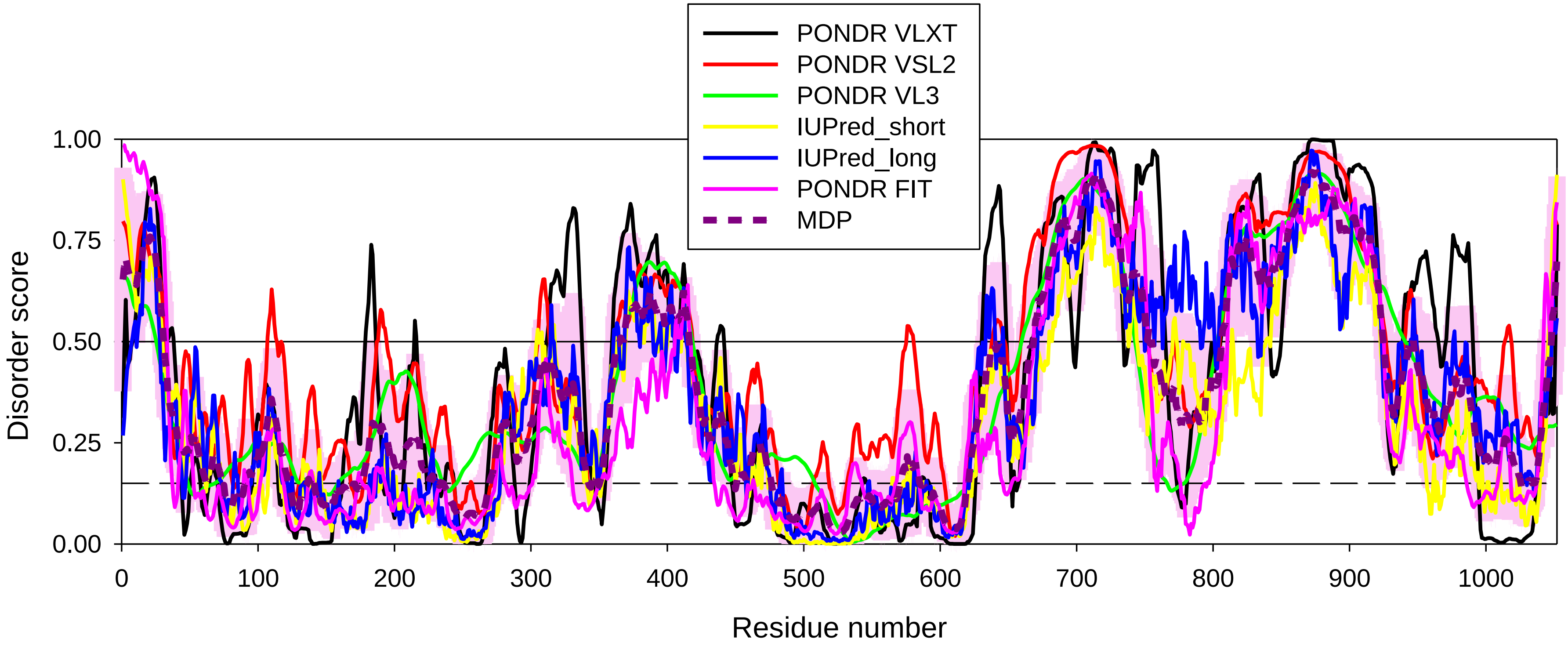

PONDR VLXT
PONDR VSL2
PONDR VL3
IUPred_short
IUPred_long
PONDR FIT
MDP
Disorder score
1.00
0.75
0.50
0.25
0.00
0
100
200
300
400
500
600
700
800
900
1000
Residue number

C

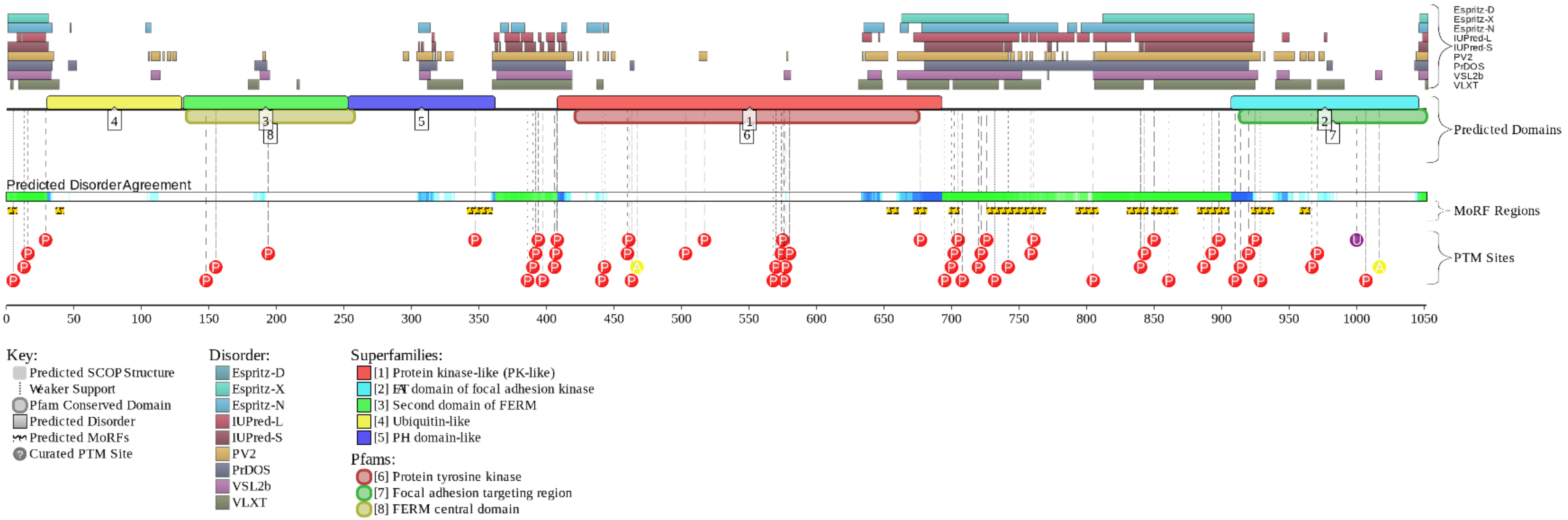

Espritz-D
Espritz-X
Espritz-N
IUPred-L
IUPred-S
PV2
PrDOS
VSL2b
VLXT
Predicted Domains
Predicted DisorderAgreement
MoRF Regions
PTM Sites
0 50 100 150 200 250 300 350 400 450 500 550 600 650 700 750 800 850 900 950 1000 1050
Key:
Predicted SCOP Structure
Weaker Support
Pfam Conserved Domain
Predicted Disorder
Predicted MoRFs
Curated PTM Site
Disorder:
Espritz-D
Espritz-X
Espritz-N
IUPred-L
IUPred-S
PV2
PrDOS
VSL2b
VLXT
Superfamilies:
[1] Protein kinase-like (PK-like)
[2] FAT domain of focal adhesion kinase
[3] Second domain of FERM
[4] Ubiquitin-like
[5] PH domain-like
Pfams:
[6] Protein tyrosine kinase
[7] Focal adhesion targeting region
[8] FERM central domain

D

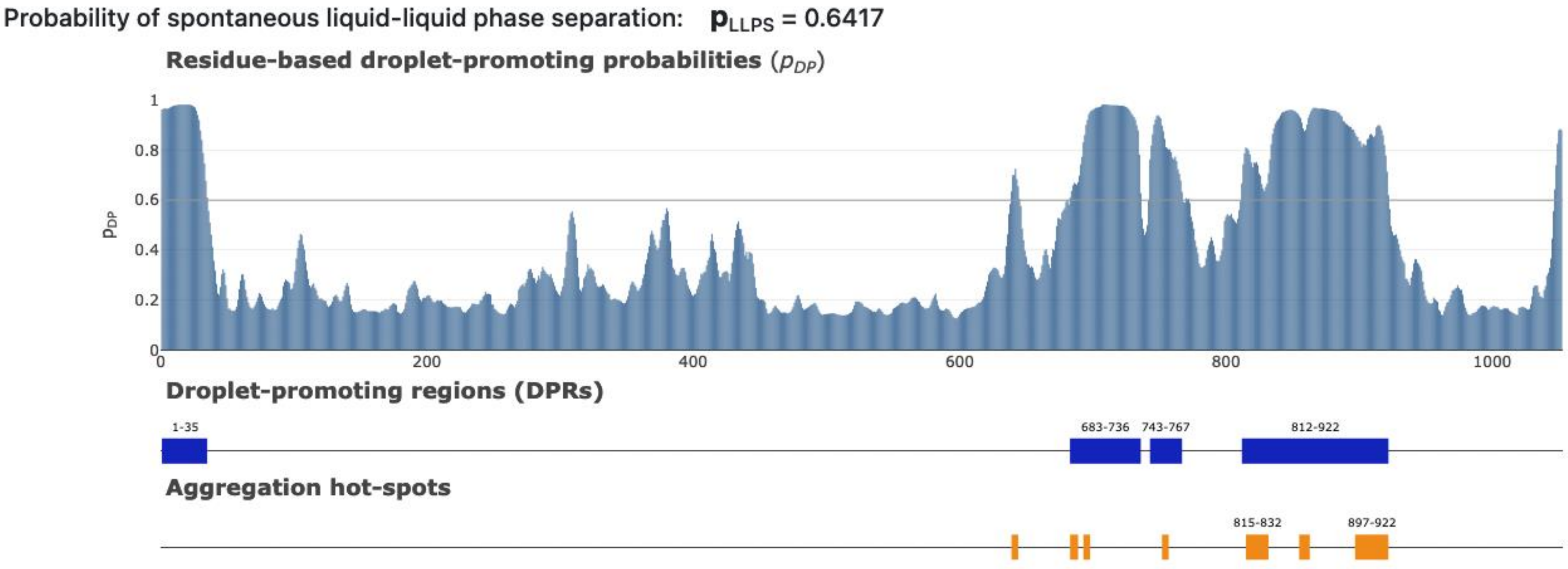
Probability of spontaneous liquid-liquid phase separation: pLLPS = 0.6417
Residue-based droplet-promoting probabilities (pDP)
pDP
1
0.8
0.6
0.4
0.2
0
0
200
400
600
800
1000
Droplet-promoting regions (DPRs)
1-35
683-736
743-767
812-922
Aggregation hot-spots
815-832
897-922

E

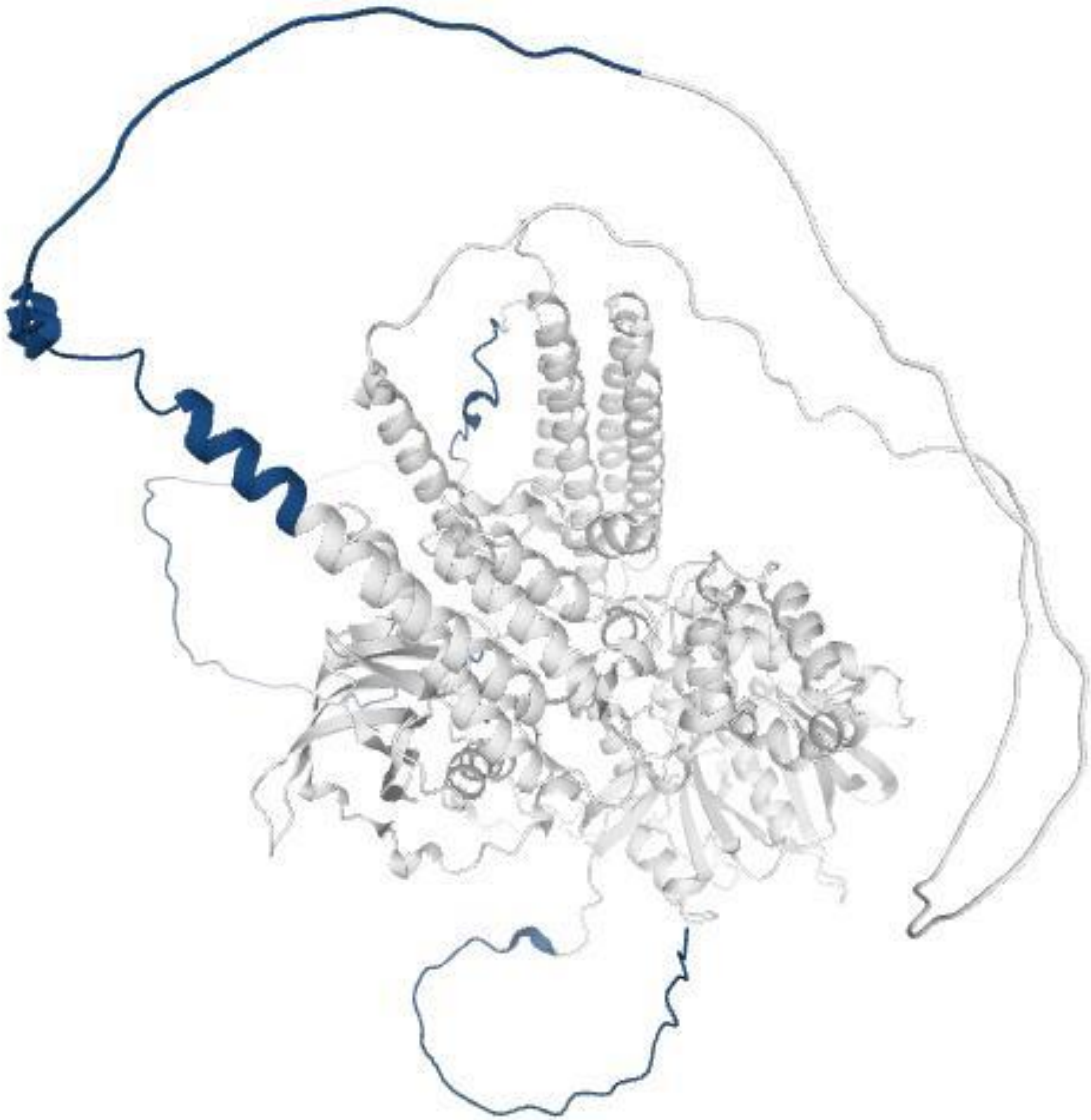

F

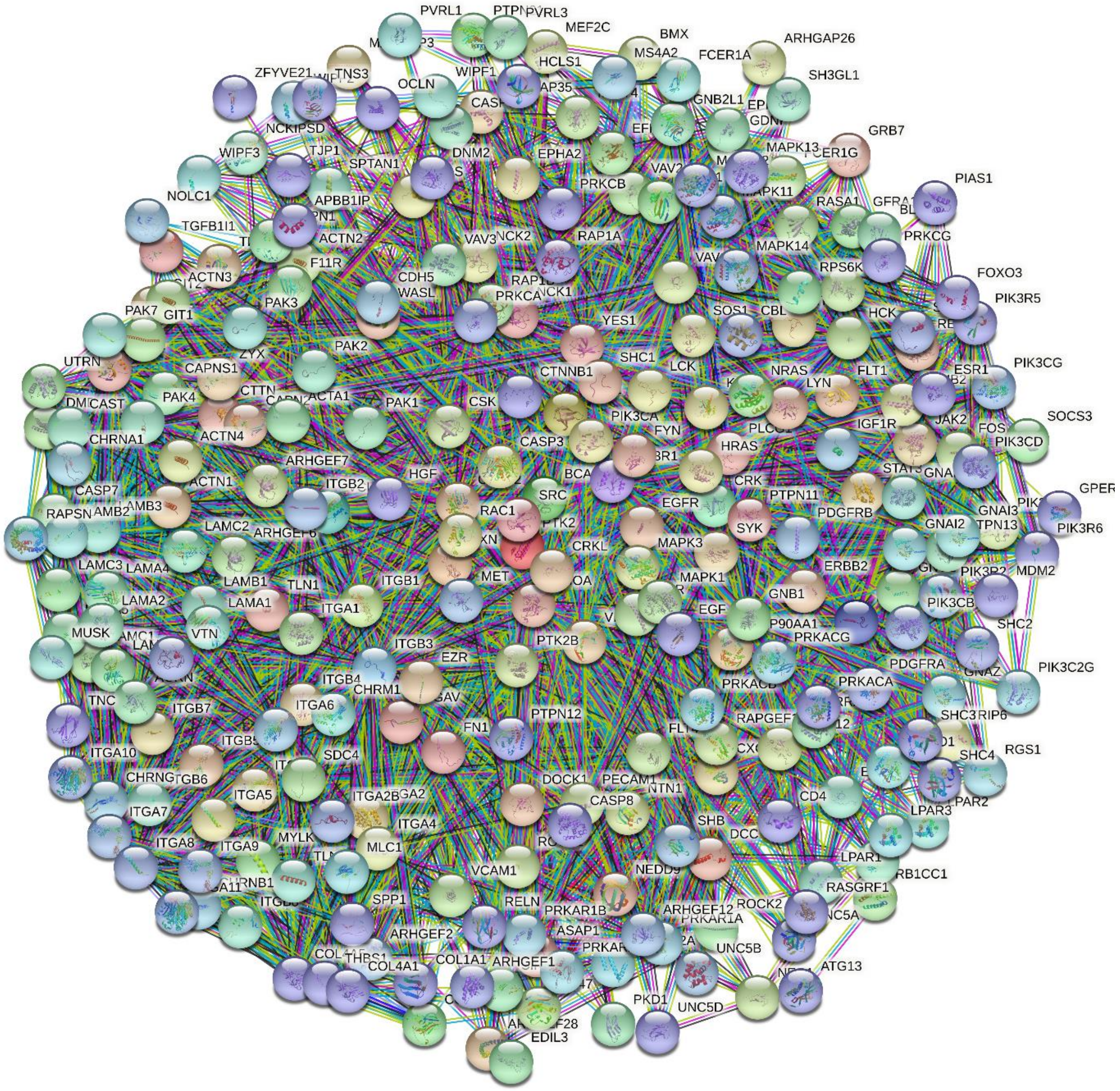

PVRL1
PVRL3
MEF2C
BMX
MS4A2
FCER1A
ARHGAP26
SH3GL1
GRB7
HCLS1
ZFYVE21
TNS3
OCLN
WIPF1
AP35
GNB2L1
NCKIPSD
WIPF3
TJP1
SPTAN1
DNM2
EPHA2
MAPK13
FCER1G
NOLC1
APBB1IP
PRKCB
VAV2
MAPK11
PIAS1
TGFB1I1
ACTN2
VAV3
NCK2
RAP1A
RASA1
PRKCG
F11R
MAPK14
RPS6K
FOXO3
ACTN3
CDH5
WASL
PRKCA
NCK1
PIK3R5
PAK7
GIT1
PAK3
YES1
SOS1
CBL
HCK
PAK2
UTRN
CAPNS1
ZYX
SHC1
LCK
NRAS
LYN
FLT1
ESR1
PIK3CG
CTNNB1
CAST
PAK4
CTTN
ACTA1
PAK1
CSK
PIK3CA
FYN
PLCG1
IGF1R
JAK2
SOCS3
CHRNA1
ACTN4
CASP3
HRAS
FOS
PIK3CD
ARHGEF7
HGF
BCAR1
STAT3
CASP7
ACTN1
ITGB2
SRC
CRK
PTPN11
GNAI3
GPER
RAPSN
LAMB2
LAMB3
RAC1
PTK2
EGFR
SYK
PDGFRB
GNAI2
PTPN13
PIK3R6
LAMC2
ARHGEF6
CRKL
MAPK3
LAMC3
LAMA4
ERBB2
PIK3R2
MDM2
LAMB1
TLN1
ITGB1
MET
MAPK1
GNB1
LAMA2
LAMA1
ITGA1
EGF
PIK3CB
MUSK
VTN
P90AA1
PRKACG
SHC2
ITGB3
PTK2B
EZR
PDGFRA
GNAZ
PIK3C2G
ITGB4
CHRM1
PRKACA
TNC
ITGA6
ITGB7
FN1
PTPN12
SHC3
ITGA10
SHC4
RGS1
CHRNG
ITGB6
SDC4
DOCK1
PECAM1
NTN1
CD4
ITGA5
ITGA2B
CASP8
LPAR3
PAR2
ITGA7
ITGA4
SHB
DCC
ITGA8
ITGA9
MYLK
MLC1
LPAR1
RB1CC1
VCAM1
NEDD9
RASGRF1
SPP1
RELN
PRKAR1B
ARHGEF12
ROCK2
PRKAR1A
ARHGEF2
ASAP1
UNC5B
COL4A1
COL1A1
ARHGEF1
ATG13
PKD1
UNC5D
EDIL3

minimum required interaction score: high confidence (0.700)

number of nodes:	263
number of edges:	5082
average node degree:	38.6
avg. local clustering coefficient:	0.605
expected number of edges:	1183
PPI enrichment p-value:	< 1.0e-16

G

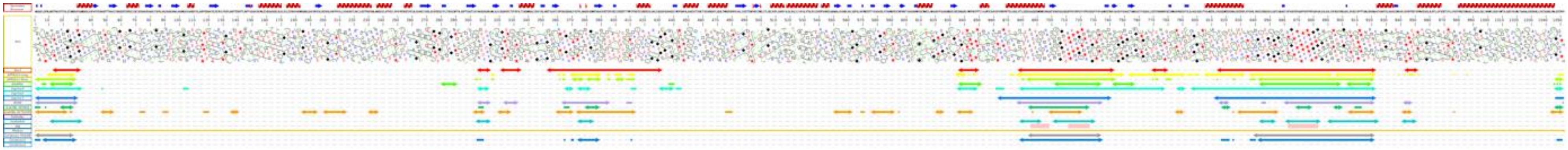

H

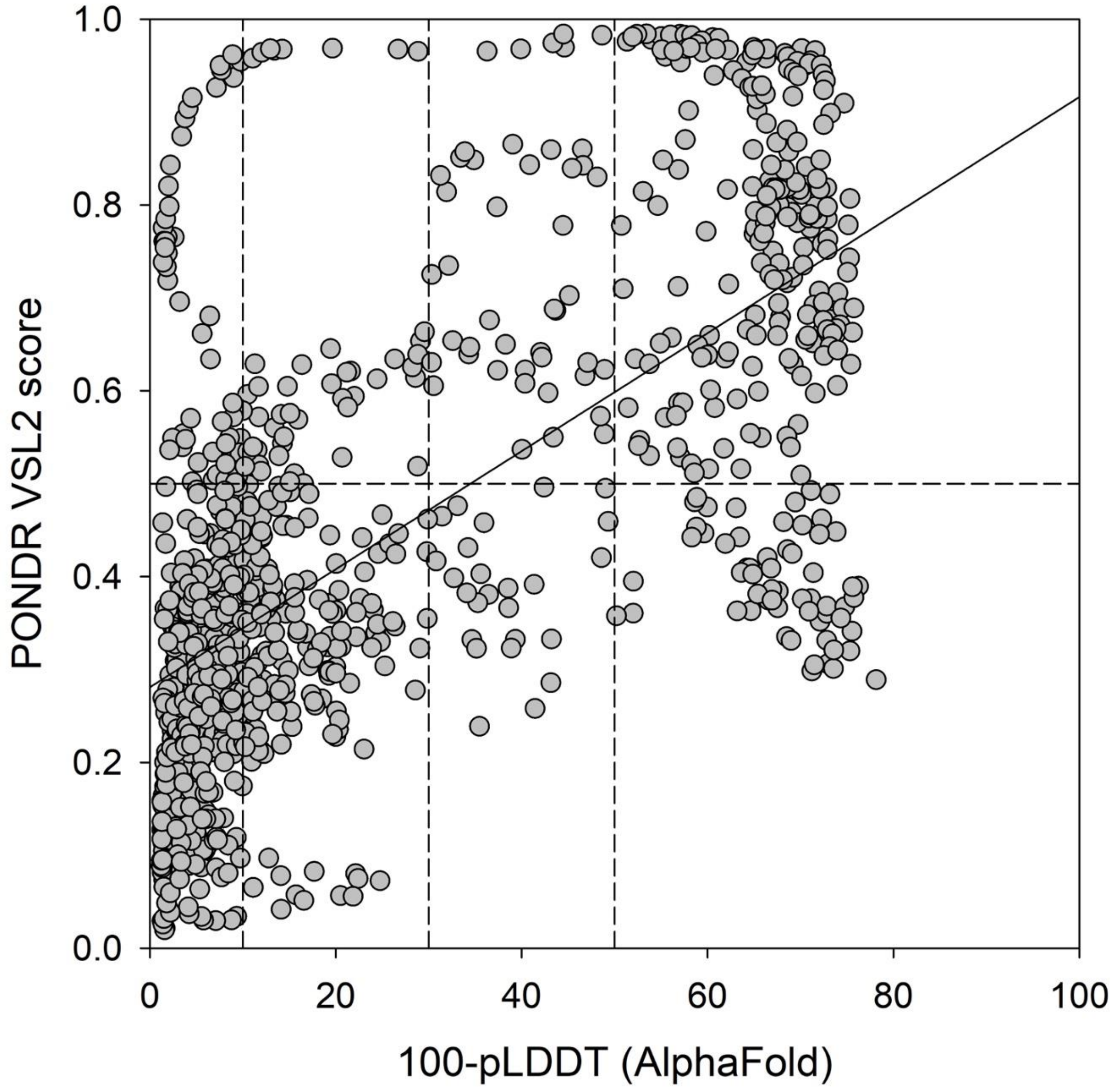

1.0
0.8
0.6
0.4
0.2
0.0
PONDR VSL2 score
0
20
40
60
80
100
100-pLDDT (AlphaFold)

Supplementary Figure S13. Functional disorder in nucleolysin TIAR. **A.** Amino acid sequence. **B.** RIDAO-generated disorder profile. **C.** D2P2-generated functional disorder profile. **D.** FuzDrop output. **E.** AlphaFold2 structure with FuzDrop annotations. **F.** STRING-generated PPI network with corresponding statistics. **G.** MeDor output. **H.** Plot of the *per* residue disorder score (as provided by PONDR® VLS2) versus (100-pLDDT), where pLDDT is the corresponding AlphaFold2 confidence score.

A

>sp|Q01085|TIAR_HUMAN Nucleolysin TIAR OS=Homo sapiens OX=9606 GN=TIAL1 PE=1 SV=1
MMEDDGQPRTLYVGNLSRDVTEVLILQLFSQIGPCKSCKMITEHTSNDPYCFVEFYEHRDAAAALAAMNGRKILGKEVKVNWATTPSSQKKDTSNHFHVFVGDLSPEITTEDIKSAFA
PFGKISDARVVKDMATGKSKGYGFVSFYNKLDAENAIVHMGGQWLGGRQIRTNWATRKPPAPKSTQENNTKQLRFEDVVNQSSPKNCTVYCGGIASGLTDQLMRQTFSPFGQIMEIRV
FPEKGYSFVRFSTHESAAHAIVSVNGTTIEGHVVKCYWGKESPDMTKNFQQVDYSQWGQWSQVYGNPQQYGQYMANGWQVPPYGVYGQPWNQQGFGVDQSPSAAWMGGFGAQPPQGQA
PPPVIPPPNQAGYGMASYQTQ

B

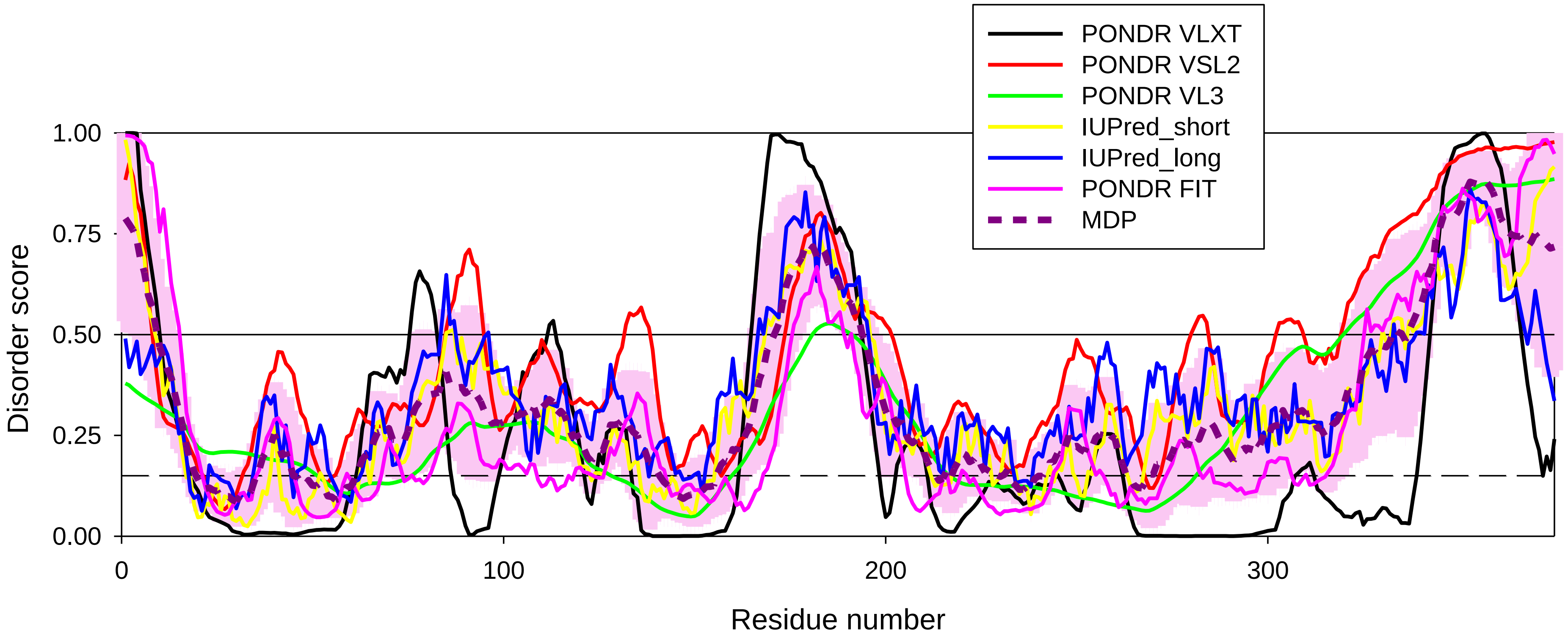

C

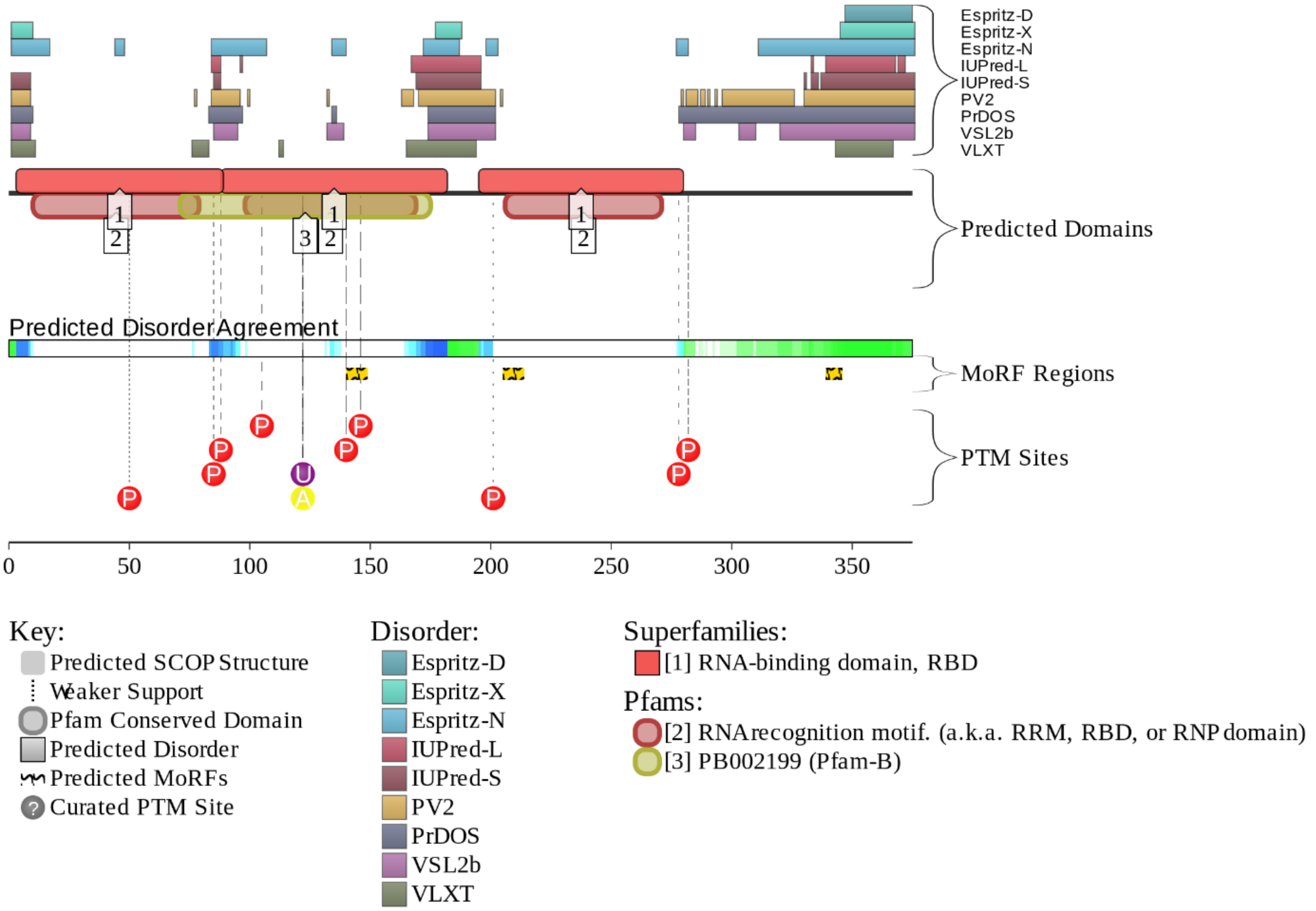
Espritz-D
Espritz-X
Espritz-N
IUPred-L
IUPred-S
PV2
PrDOS
VSL2b
VLXT
Predicted Domains
Predicted Disorder Agreement
MoRF Regions
PTM Sites
0
50
100
150
200
250
300
350
Key:
Predicted SCOP Structure
Weaker Support
Pfam Conserved Domain
Predicted Disorder
Predicted MoRFs
Curated PTM Site
Disorder:
Espritz-D
Espritz-X
Espritz-N
IUPred-L
IUPred-S
PV2
PrDOS
VSL2b
VLXT
Superfamilies:
[1] RNA-binding domain, RBD
Pfams:
[2] RNA recognition motif. (a.k.a. RRM, RBD, or RNP domain)
[3] PB002199 (Pfam-B)

D

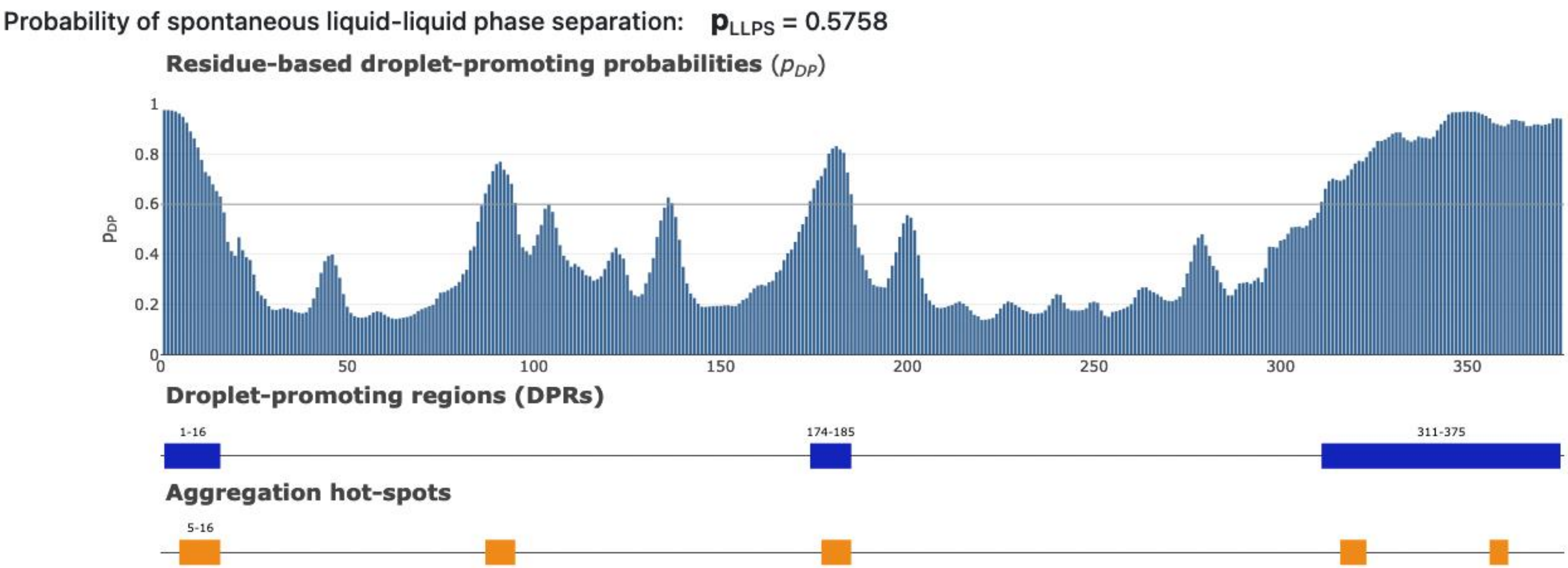
Probability of spontaneous liquid-liquid phase separation: pLLPS = 0.5758
Residue-based droplet-promoting probabilities (pDP)
pDP
1
0.8
0.6
0.4
0.2
0
0
50
100
150
200
250
300
350
Droplet-promoting regions (DPRs)
1-16
174-185
311-375
Aggregation hot-spots
5-16

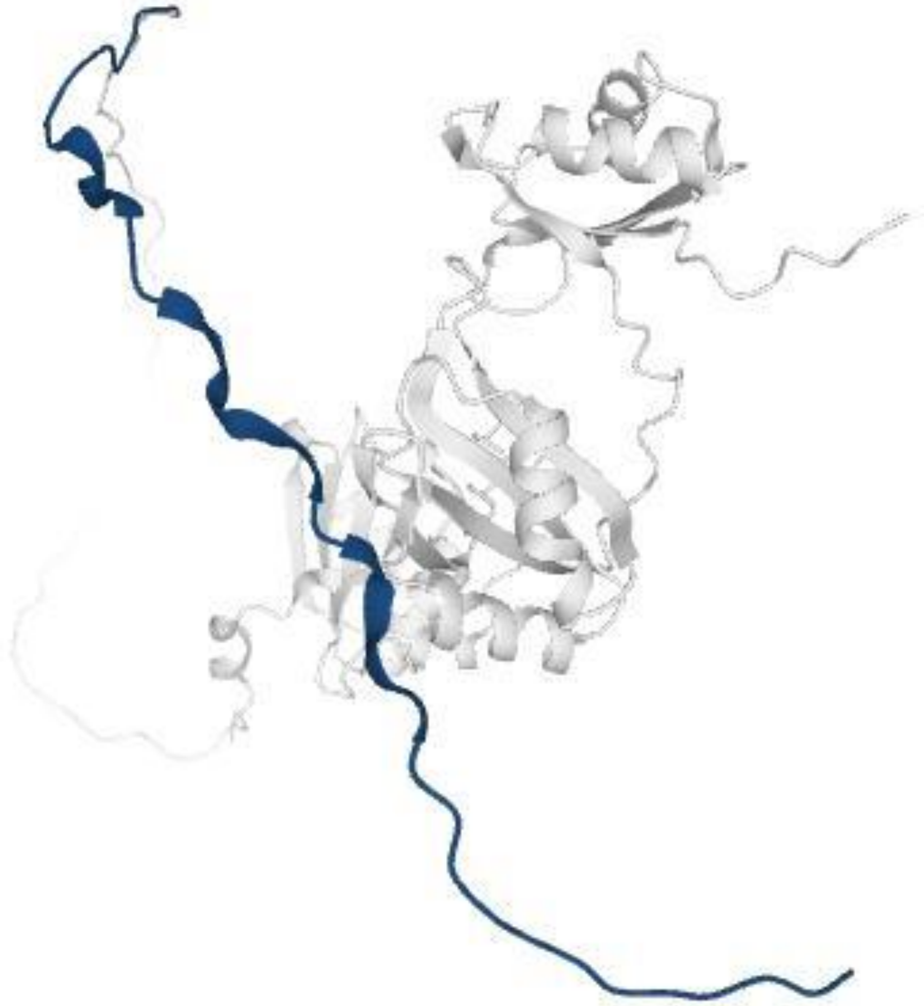

E

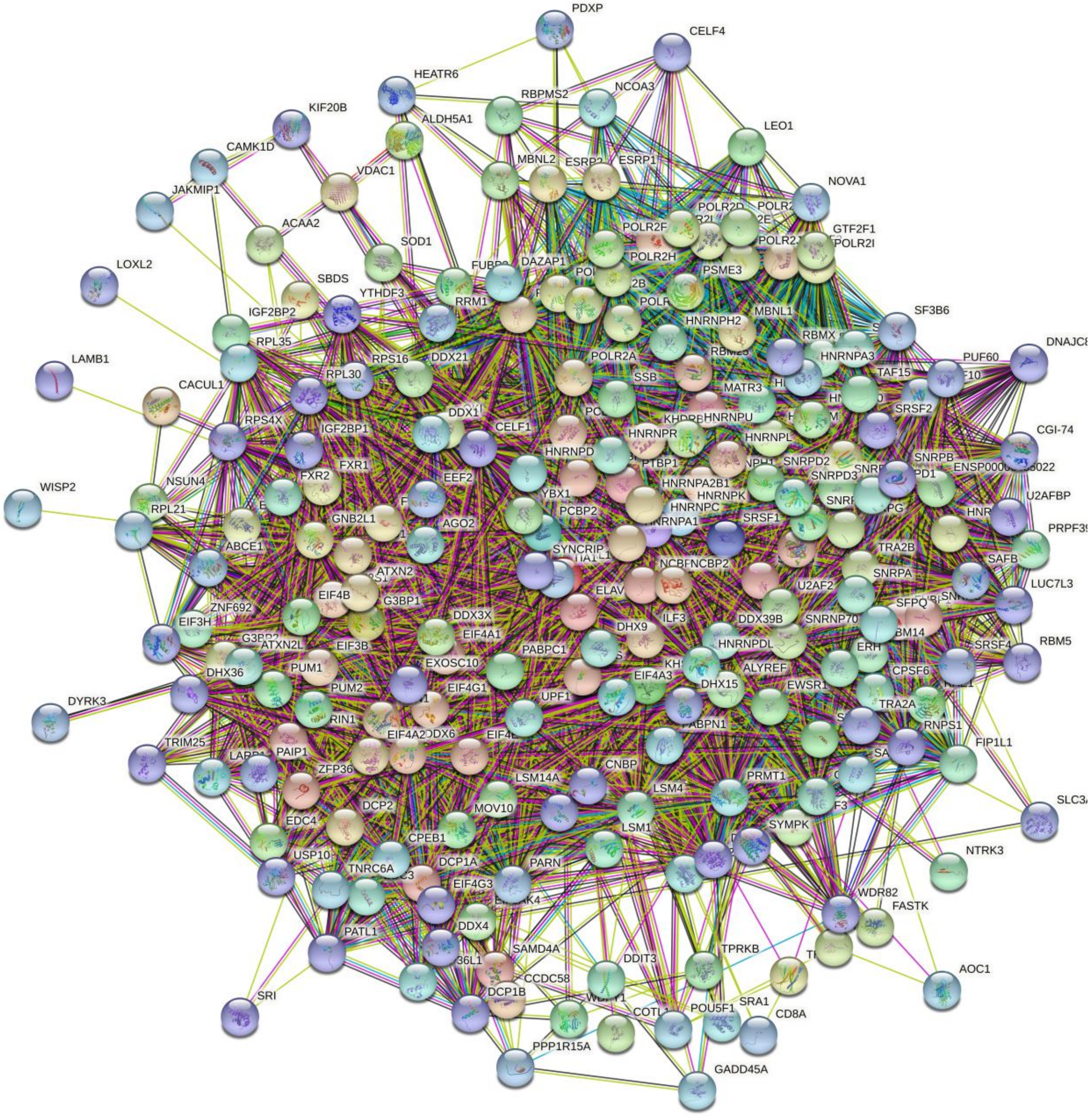
PDXP
CELF4
HEATR6
RBPMS2
NCOA3
KIF20B
ALDH5A1
LEO1
CAMK1D
VDAC1
MBNL2
ESRP2
ESRP1
NOVA1
JAKMIP1
ACAA2
SOD1
POLR2F
POLR2D
GTF2F1
POLR2I
LOXL2
SBDS
YTHDF3
DAZAP1
POLR2H
PSME3
POLR2J
IGF2BP2
RRM1
HNRNPH2
MBNL1
SF3B6
RPL35
RPS16
DDX21
LAMB1
RPL30
POLR2A
RBMX
PUF60
HNRNPA3
TAF15
CACUL1
SSB
MATR3
SRSF2
DDX1
CELF1
RPS4X
IGF2BP1
HNRNPU
CGI-74
HNRNPD
HNRNPL
FXR1
SNRPD2
SNRPB
EEF2
PTBP1
NSUN4
FXR2
SNRPD3
YBX1
HNRNPA2B1
U2AFBP
WISP2
HNRNPK
RPL21
GNB2L1
AGO2
PCBP2
HNRNPC
SRSF1
ABCE1
SYNCRIP
TRA2B
SAFB
ATXN2
NCBP2
SNRPA
LUC7L3
EIF4B
U2AF2
G3BP1
ILF3
DDX39B
SNRNP70
ZNF692
DDX3X
EIF3H
SFPQ
EIF4A1
DHX9
RBM5
ATXN2L
EIF3B
PABPC1
HNRNPDL
ERH
SRSF4
PUM1
EXOSC10
ALYREF
CPSF6
DHX36
EIF4A3
DHX15
EWSR1
PUM2
EIF4G1
DYRK3
UPF1
TRA2A
RIN1
EIF4A2
DDX6
RNPS1
FIP1L1
TRIM25
PAIP1
ZFP36
CNBP
LSM14A
PRMT1
LSM4
DCP2
MOV10
EDC4
SYMPK
LSM1
CPEB1
NTRK3
USP10
DCP1A
PARN
TNRC6A
EIF4G3
WDR82
FASTK
DDX4
PATL1
SAMD4A
TPRKB
DDIT3
CCDC58
AOC1
SRI
DCP1B
POU5F1
SRA1
CD8A
COTL1
PPP1R15A
GADD45A

F

minimum required interaction score: medium confidence (0.400)
number of nodes: 222
number of edges: 6457
average node degree: 58.2
avg. local clustering coefficient: 0.669
expected number of edges: 1131
PPI enrichment p-value: < 1.0e-16

G

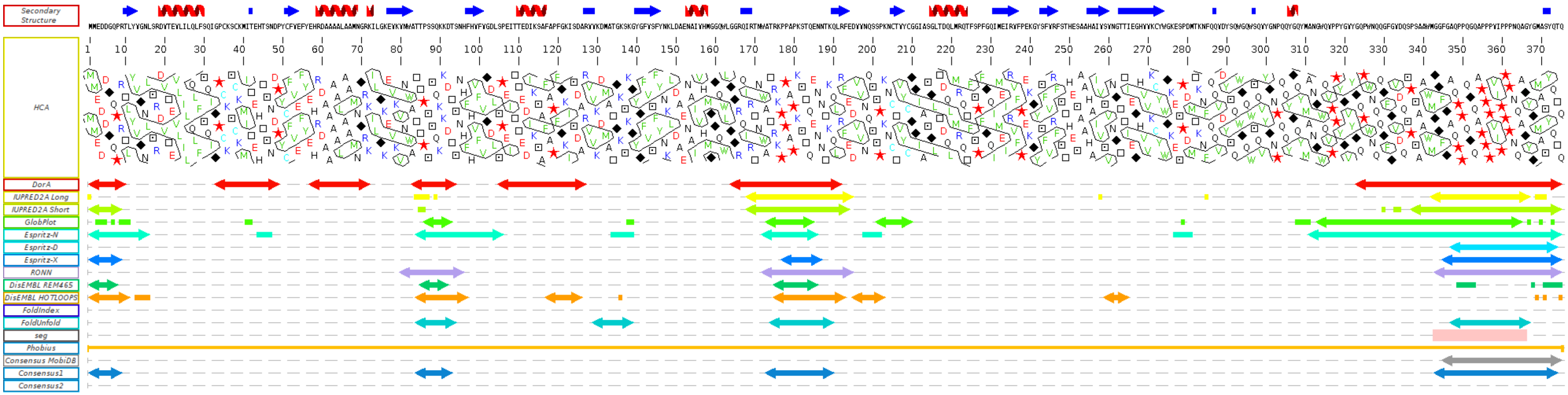

Secondary Structure
HCA
DorA
IUPRED2A Long
IUPRED2A Short
GlobPlot
Espritz-N
Espritz-D
Espritz-X
RONN
DisEMBL REM465
DisEMBL HOTLOOPS
FoldIndex
FoldUnfold
seg
Phobius
Consensus MobiDB
Consensus1
Consensus2

H

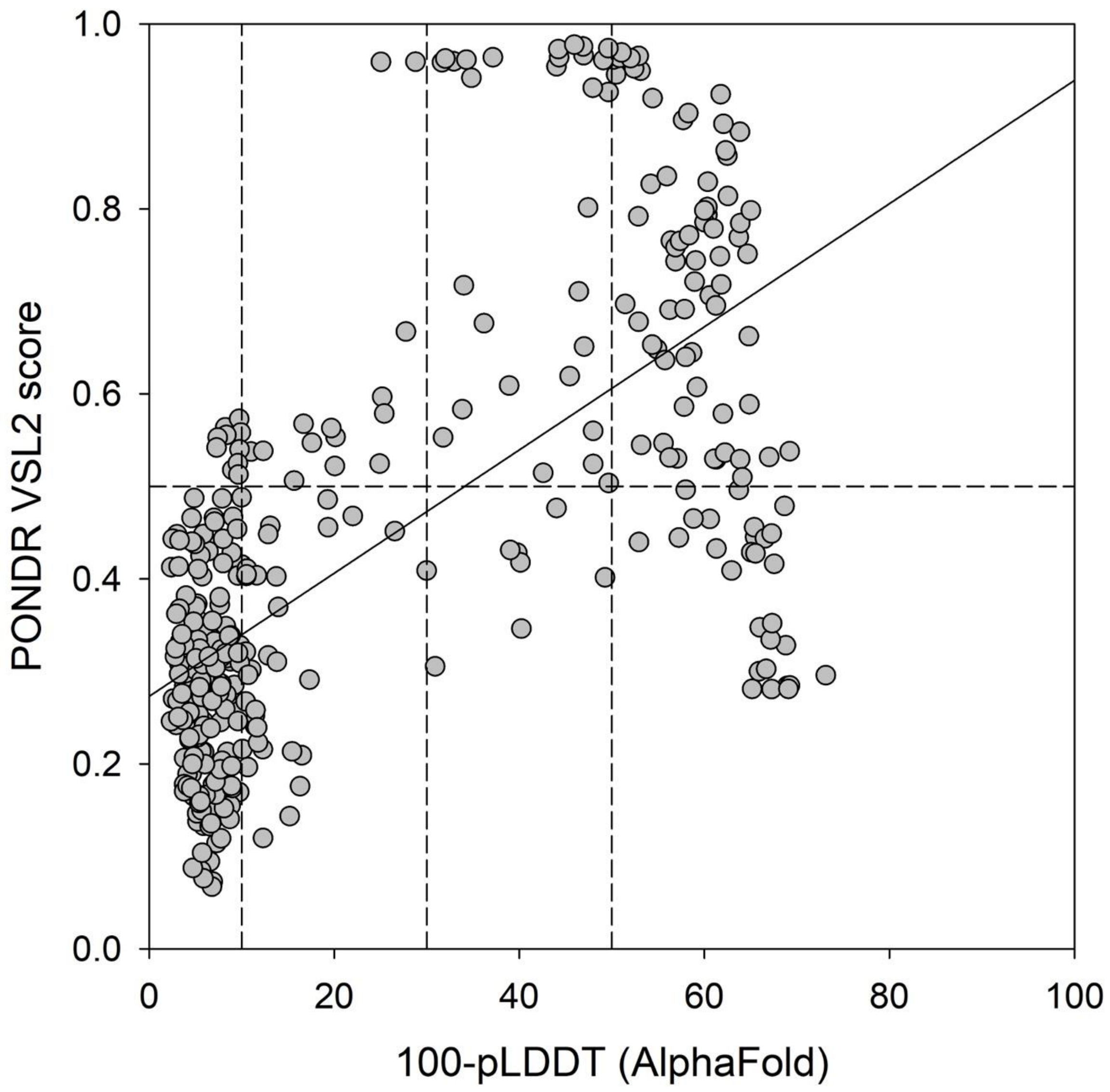
1.0
0.8
0.6
0.4
0.2
0.0
PONDR VSL2 score
0
20
40
60
80
100
100-pLDDT (AlphaFold)

Supplementary Figure S14. Functional disorder in heat shock 70 kDa protein 1A. **A.** Amino acid sequence. **B.** RIDAO-generated disorder profile. **C.** D2P2-generated functional disorder profile. **D.** FuzDrop output. **E.** AlphaFold2 structure with FuzDrop annotations. **F.** STRING-generated PPI network with corresponding statistics. **G.** MeDor output. **H.** Plot of the *per* residue disorder score (as provided by PONDR® VLS2) versus (100-pLDDT), where pLDDT is the corresponding AlphaFold2 confidence score.

A

>sp|P0DMV8|HS71A_HUMAN Heat shock 70 kDa protein 1A OS=Homo sapiens OX=9606 GN=HSPA1A PE=1 SV=1
MAKAAAIGIDLGTTYSCVGVFQHGKVEIIANDQGNRTTPSYVAFTDTERLIGDAAKNQVALNPQNTVFDAKRLIGRKFGDPVVQSDMKHWPFQVINDGDKPKVQVSYKGETKAFYPEE
ISSMVLTKMKEIAEAYLGYPVTNAVITVPAYFNDSQRQATKDAGVIAGLNVLRIINEPTAAAIAYGLDRTGKGERNVLIFDLGGGTFDVSILTIDDGIFEVKATAGDTHLGGEDFDNR
LVNHFVEEFKRKHKKDISQNKRAVRRLRTACERAKRTLSSSTQASLEIDSLFEGIDFYTSITRARFEELCSDLFRSTLEPVEKALRDAKLDKAQIHDLVLVGGSTRIPKVQKLLQDFF
NGRDLNKSINPDEAVAYGAAVQAAILMGDKSENVQDLLLLDVAPLSLGLETAGGVMTALIKRNSTIPTKQTQIFTTYSDNQPGVLIQVYEGERAMTKDNNLLGRFELSGIPPAPRGVP
QIEVTFDIDANGILNVTATDKSTGKANKITITNDKGRLSKEEIERMVQEAEKYKAEDEVQRERVSAKNALESYAFNMKSAVEDEGLKGKISEADKKKVLDKCQEVISWLDANTLAEKD
EFEHKRKELEQVCNPIISGLYQGAGGPGPGGFGAQGPKGGSGSGPTIEEVD

B

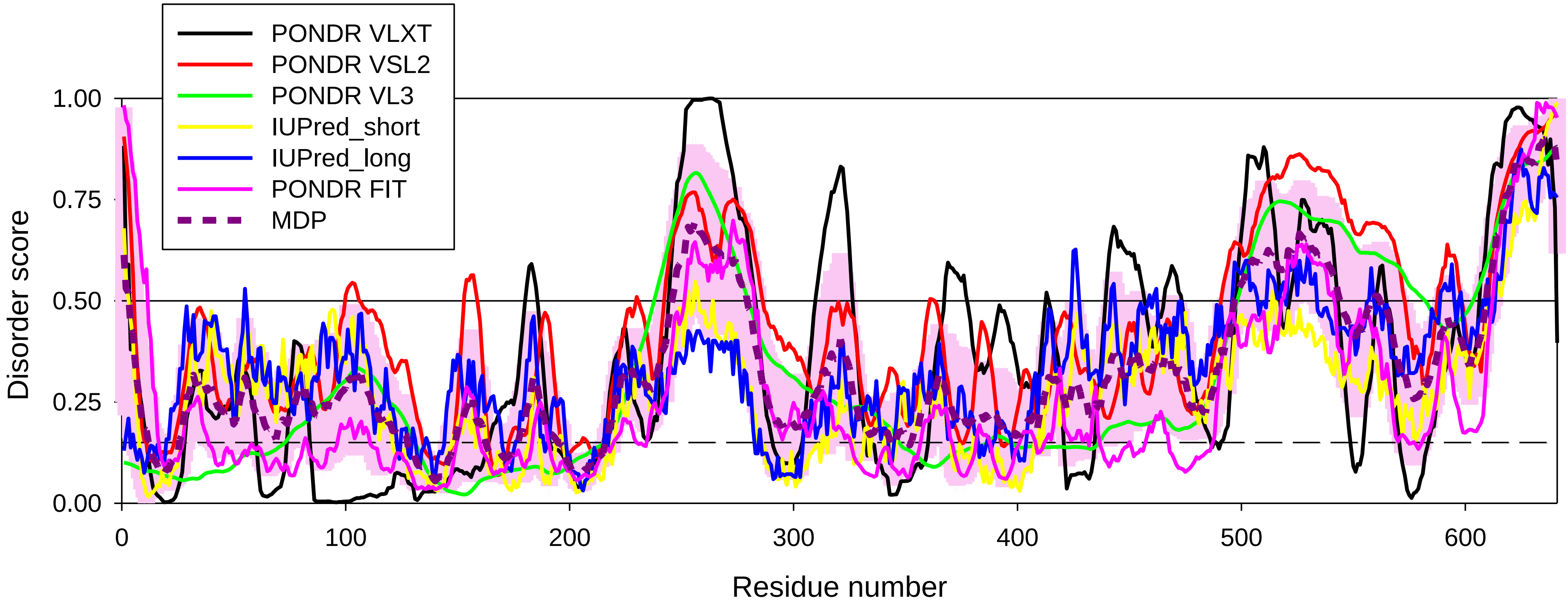

C

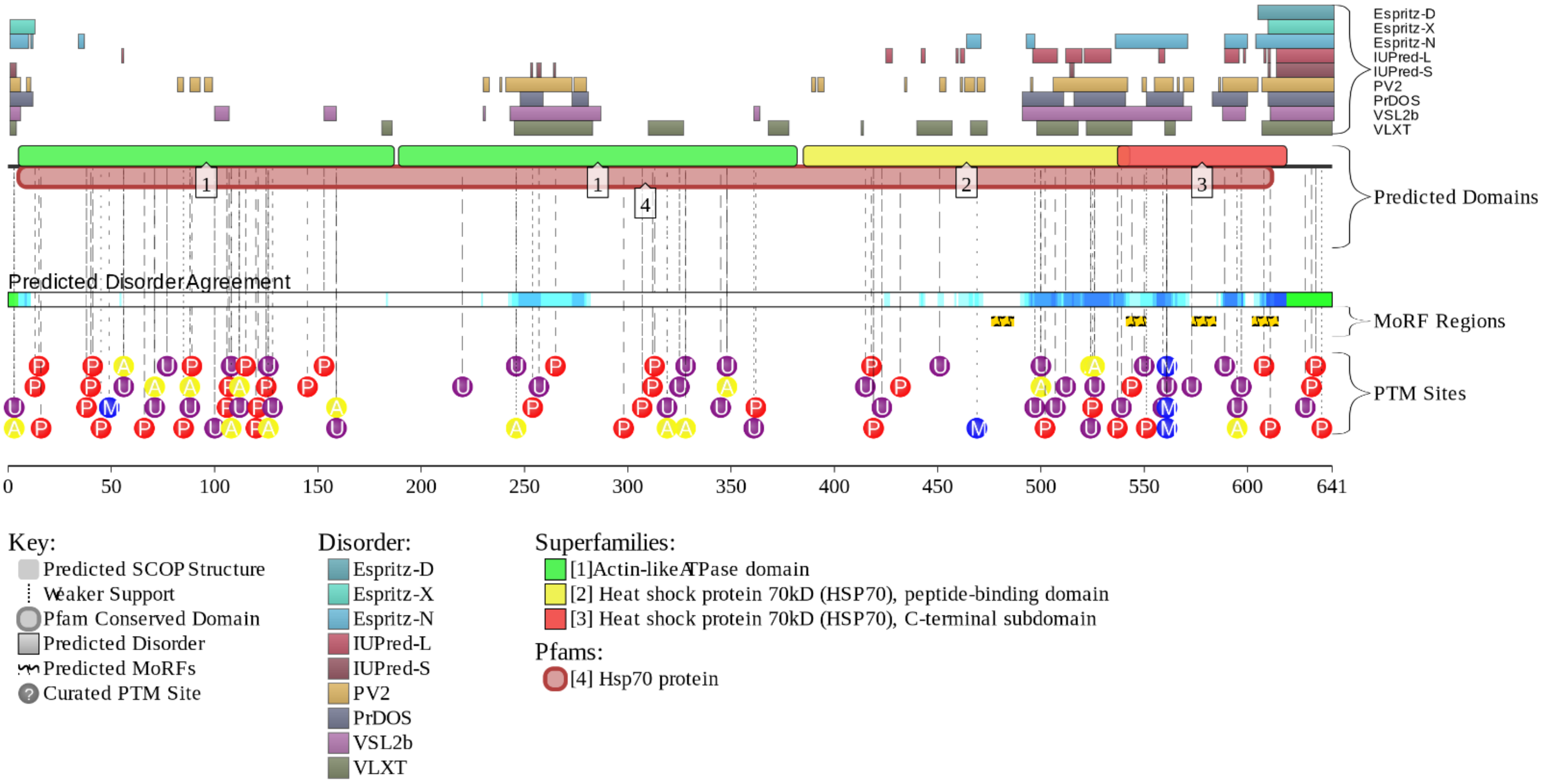
Espritz-D
Espritz-X
Espritz-N
IUPred-L
IUPred-S
PV2
PrDOS
VSL2b
VLXT
Predicted Domains
Predicted Disorder Agreement
MoRF Regions
PTM Sites
0
50
100
150
200
250
300
350
400
450
500
550
600
641
Key:
Predicted SCOP Structure
Weaker Support
Pfam Conserved Domain
Predicted Disorder
Predicted MoRFs
Curated PTM Site
Disorder:
Espritz-D
Espritz-X
Espritz-N
IUPred-L
IUPred-S
PV2
PrDOS
VSL2b
VLXT
Superfamilies:
[1] Actin-like ATPase domain
[2] Heat shock protein 70kD (HSP70), peptide-binding domain
[3] Heat shock protein 70kD (HSP70), C-terminal subdomain
Pfams:
[4] Hsp70 protein

D

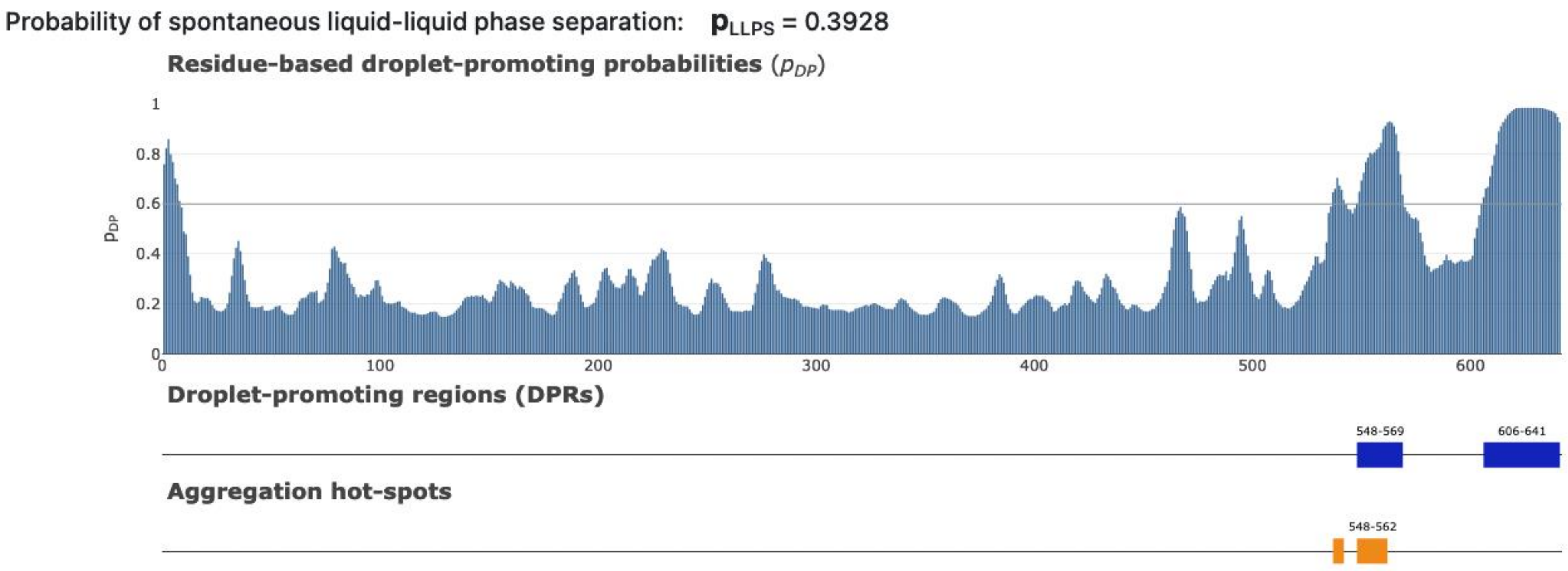

Probability of spontaneous liquid-liquid phase separation: p_{LLPS} = 0.3928
Residue-based droplet-promoting probabilities (p_{DP})
p_{DP}
1
0.8
0.6
0.4
0.2
0
0
100
200
300
400
500
600
Droplet-promoting regions (DPRs)
548-569
606-641
Aggregation hot-spots
548-562

E

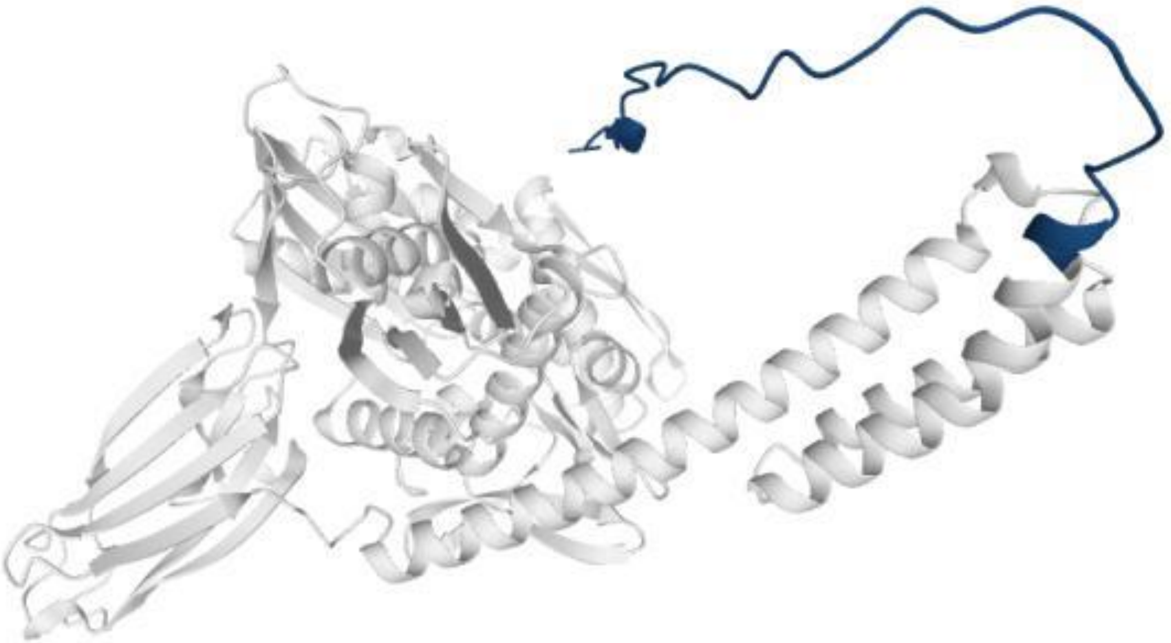

F

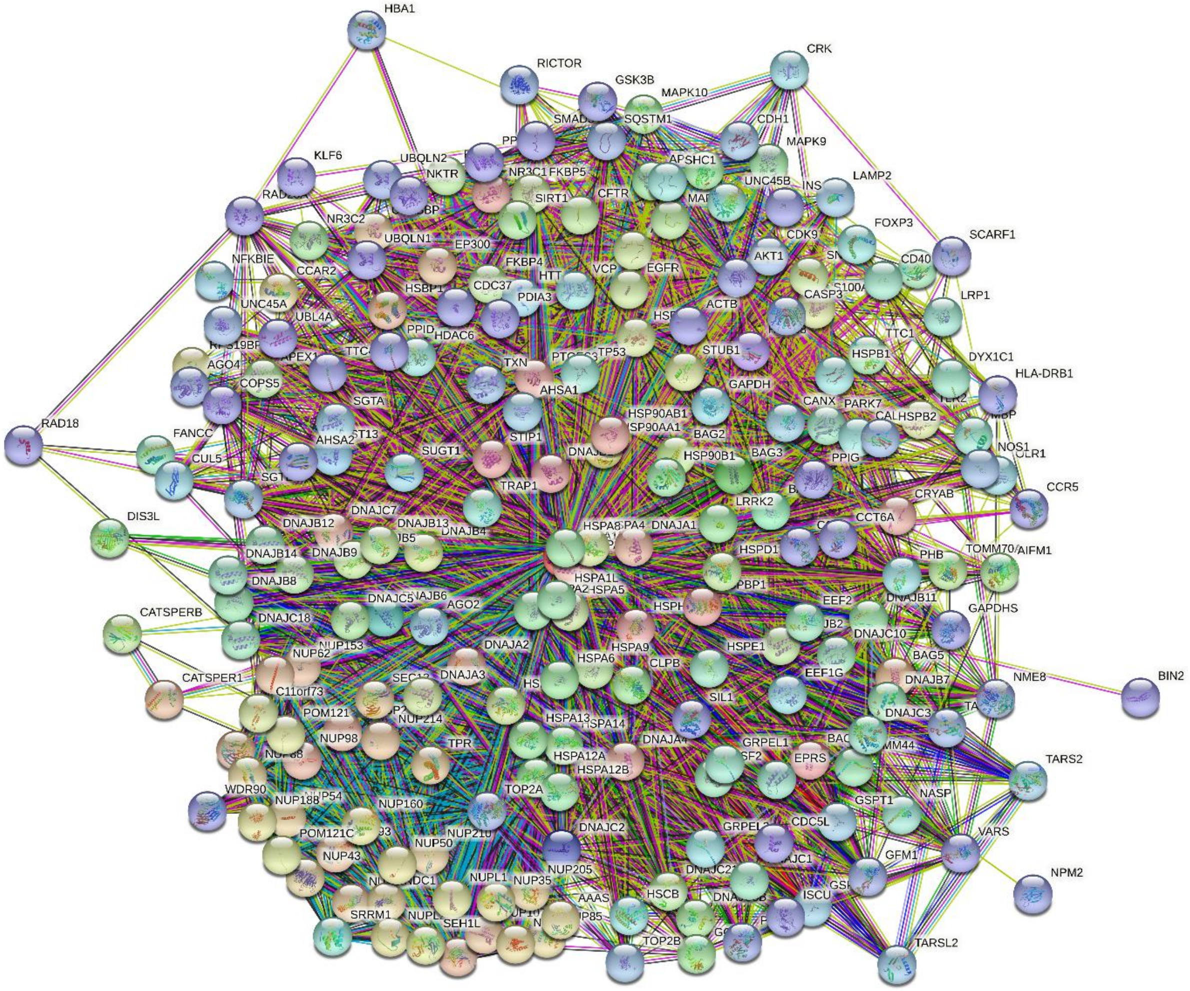

minimum required interaction score: medium confidence (0.400)
number of nodes: 215
number of edges: 4876
average node degree: 45.4

avg. local clustering coefficient: 0.697
expected number of edges: 1281
PPI enrichment p-value: < 1.0e-16

G

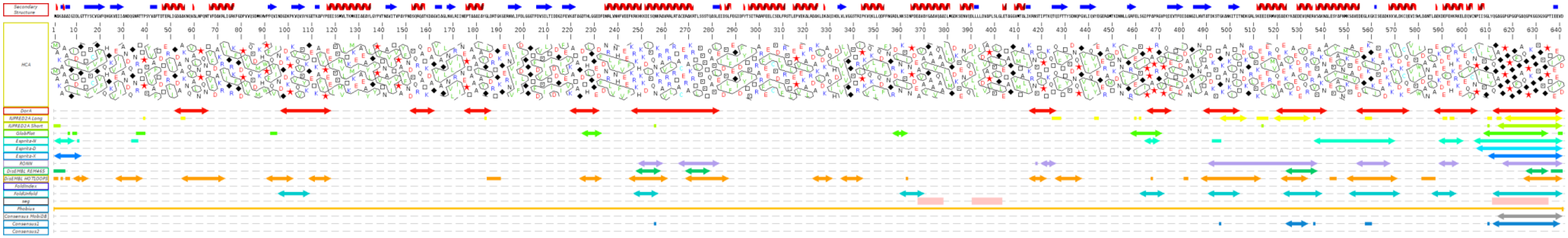

H

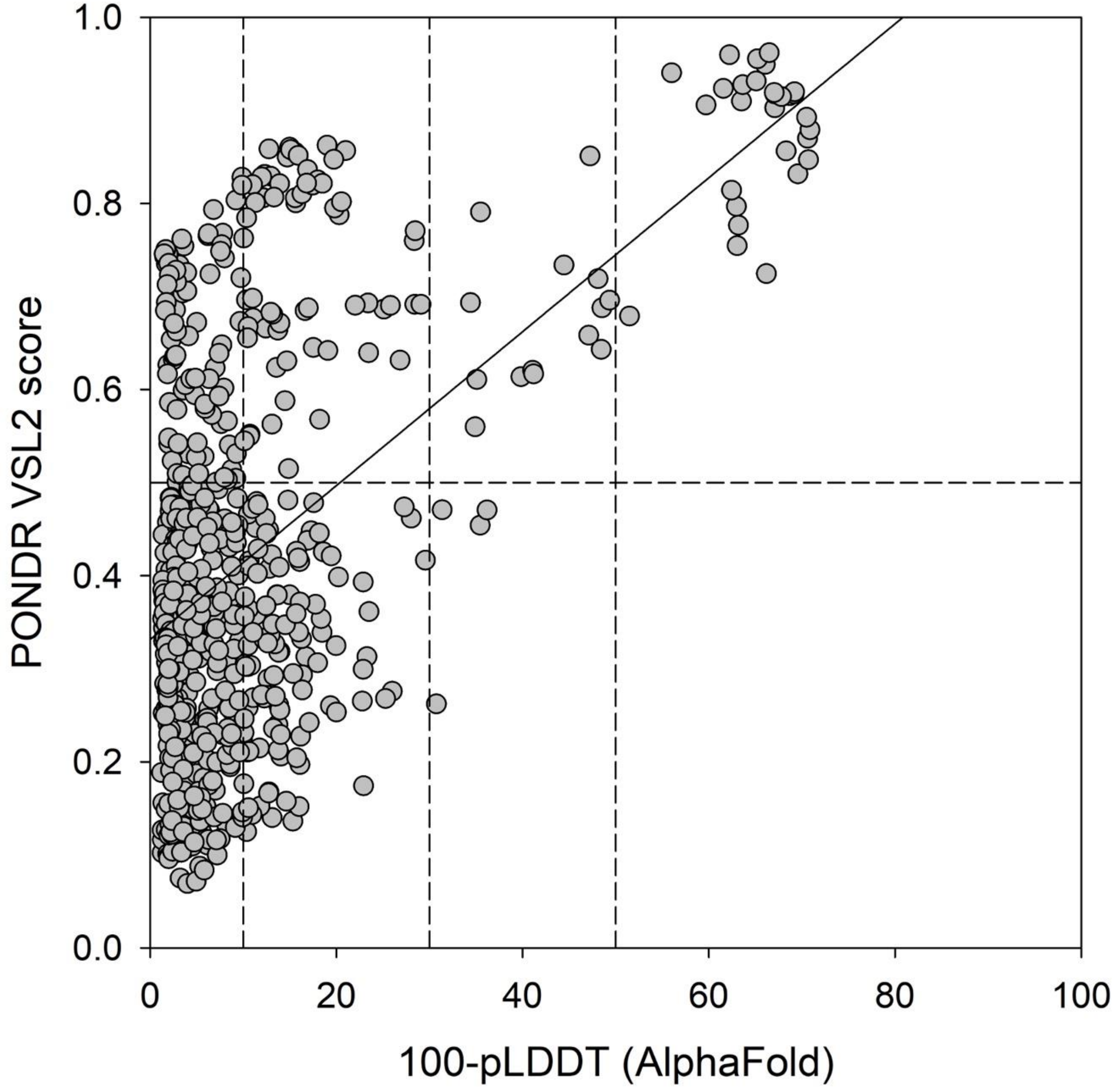

1.0
0.8
0.6
0.4
0.2
0.0
PONDR VSL2 score
0
20
40
60
80
100
100-pLDDT (AlphaFold)

Supplementary Figure S15. Functional disorder in Ras-related protein Rab-11A. **A.** Amino acid sequence. **B.** RIDAO-generated disorder profile. **C.** D2P2-generated functional disorder profile. **D.** FuzDrop output. **E.** AlphaFold2 structure with FuzDrop annotations. **F.** STRING-generated PPI network with corresponding statistics. **G.** MeDor output. **H.** Plot of the *per* residue disorder score (as provided by PONDR® VLS2) versus (100-pLDDT), where pLDDT is the corresponding AlphaFold2 confidence score.

A

>sp|P62491|RB11A_HUMAN Ras-related protein Rab-11A OS=Homo sapiens OX=9606 GN=RAB11A PE=1 SV=3
MGTRDDEYDYLFKVVLIGDSGVGKSNLLSRFTRNEFNLESKSTIGVEFATRSIQVDGKTIKAQIWDTAGQERYRAITSAYYRGAVGALLVYDIAKHLTYENVERWLKELRDHADSNIV
IMLVGNKSDLRHLRAVPTDEARAFAEKNGLSFIETSALDSTNVEAAFQTILTEIYRIVSQKQMSDRRENDMSPSNNVVPIHVPPTTENKPKVQCCQNI

B

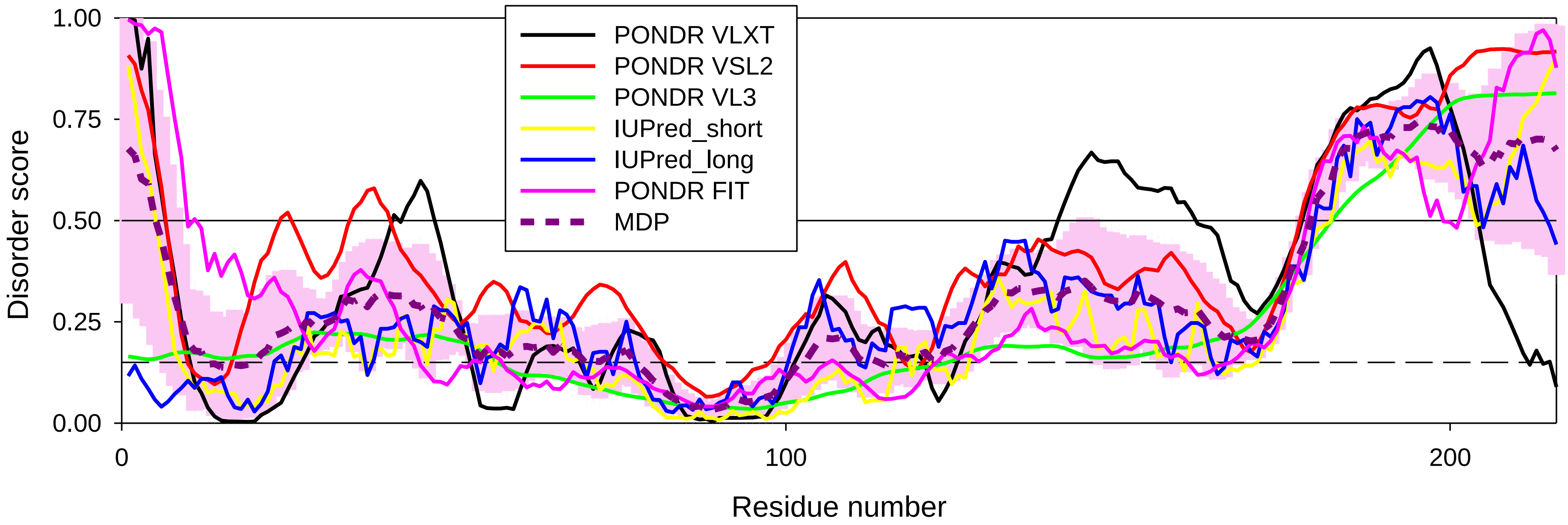

C

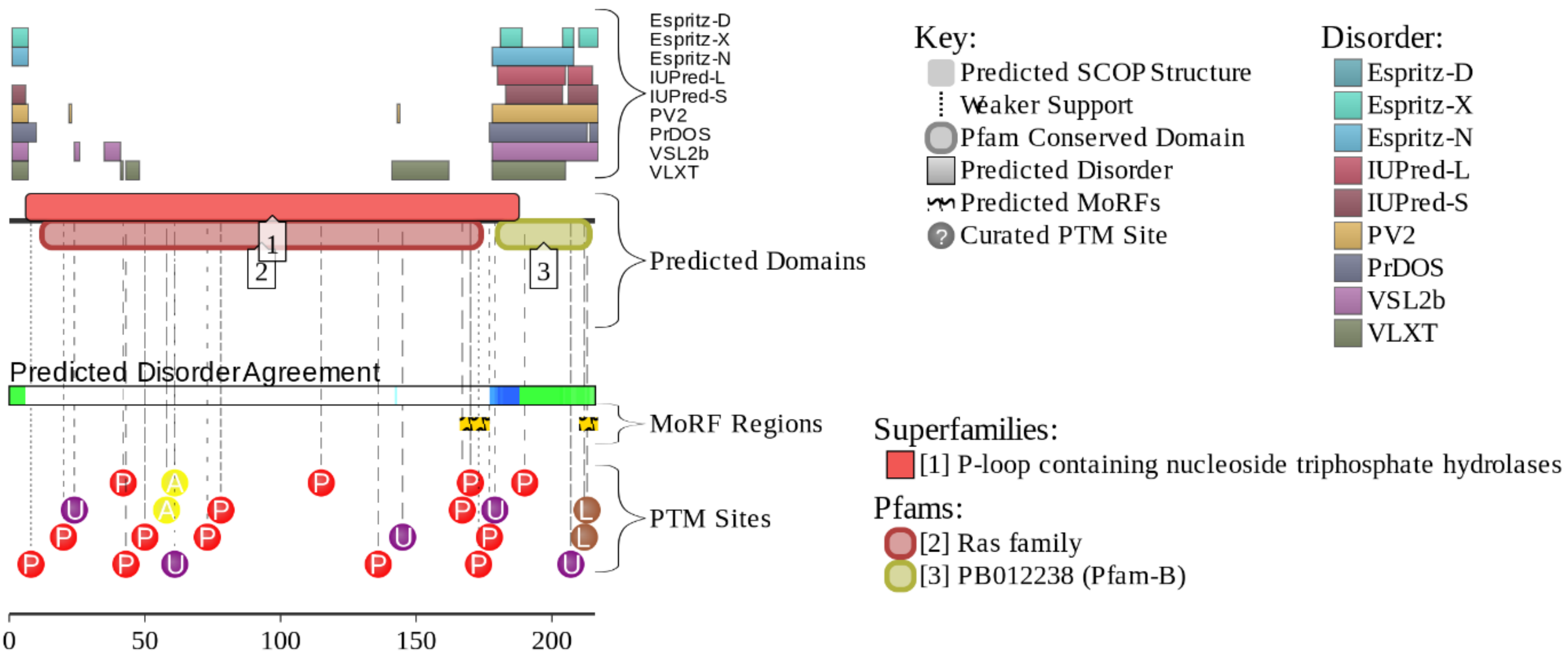

Espritz-D
Espritz-X
Espritz-N
IUPred-L
IUPred-S
PV2
PrDOS
VSL2b
VLXT
Predicted Domains
1
2
3
Predicted Disorder Agreement
MoRF Regions
PTM Sites
0
50
100
150
200
Key:
Predicted SCOP Structure
Weaker Support
Pfam Conserved Domain
Predicted Disorder
Predicted MoRFs
Curated PTM Site
Disorder:
Espritz-D
Espritz-X
Espritz-N
IUPred-L
IUPred-S
PV2
PrDOS
VSL2b
VLXT
Superfamilies:
[1] P-loop containing nucleoside triphosphate hydrolases
Pfams:
[2] Ras family
[3] PB012238 (Pfam-B)

D

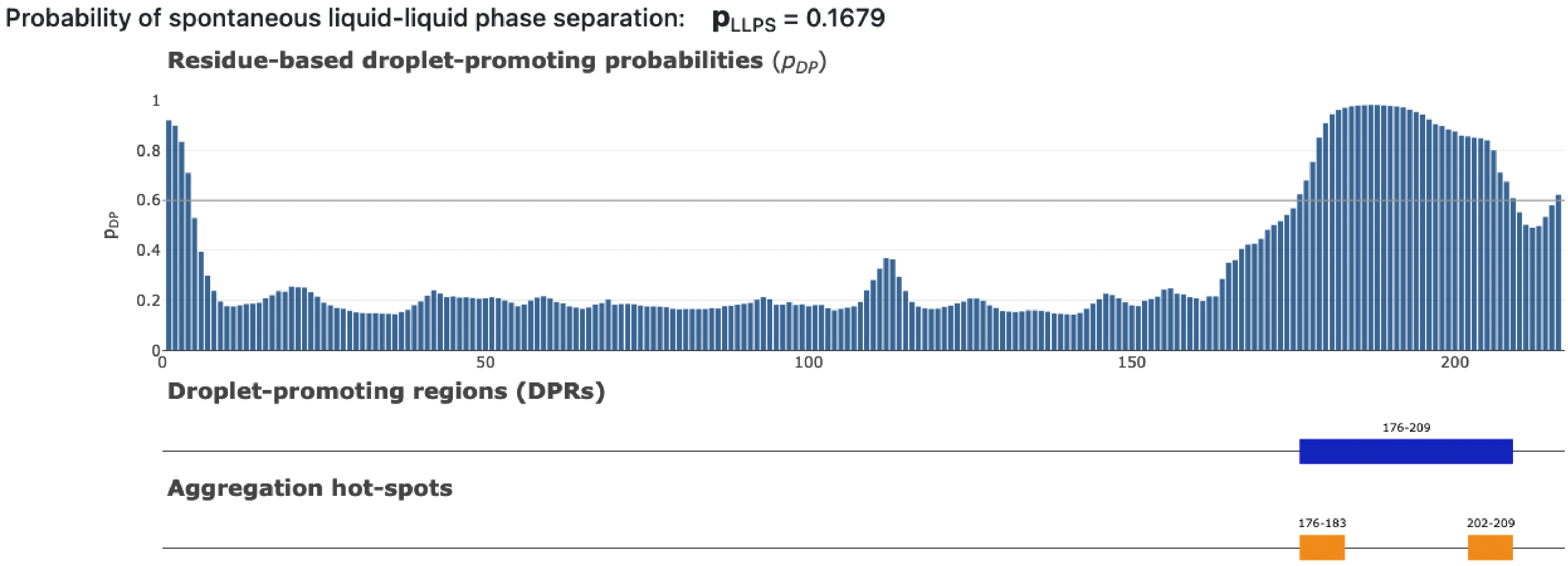
Probability of spontaneous liquid-liquid phase separation: p_{LLPS} = 0.1679
Residue-based droplet-promoting probabilities (p_{DP})
p_{DP}
1
0.8
0.6
0.4
0.2
0
0
50
100
150
200
Droplet-promoting regions (DPRs)
176-209
Aggregation hot-spots
176-183
202-209

E

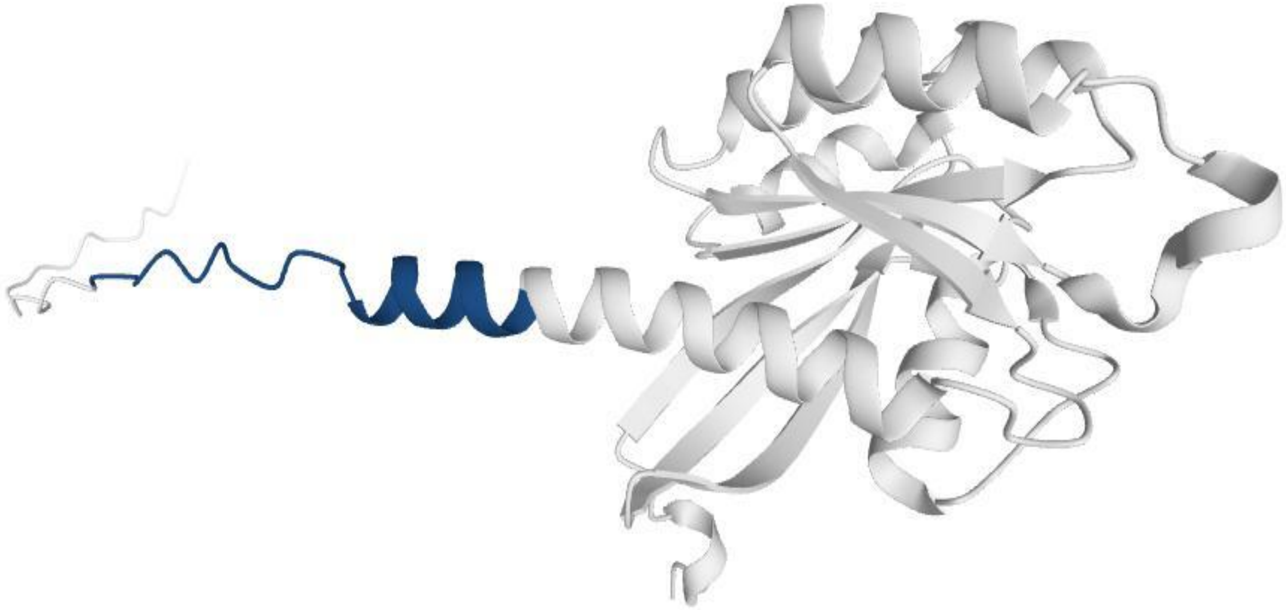

F

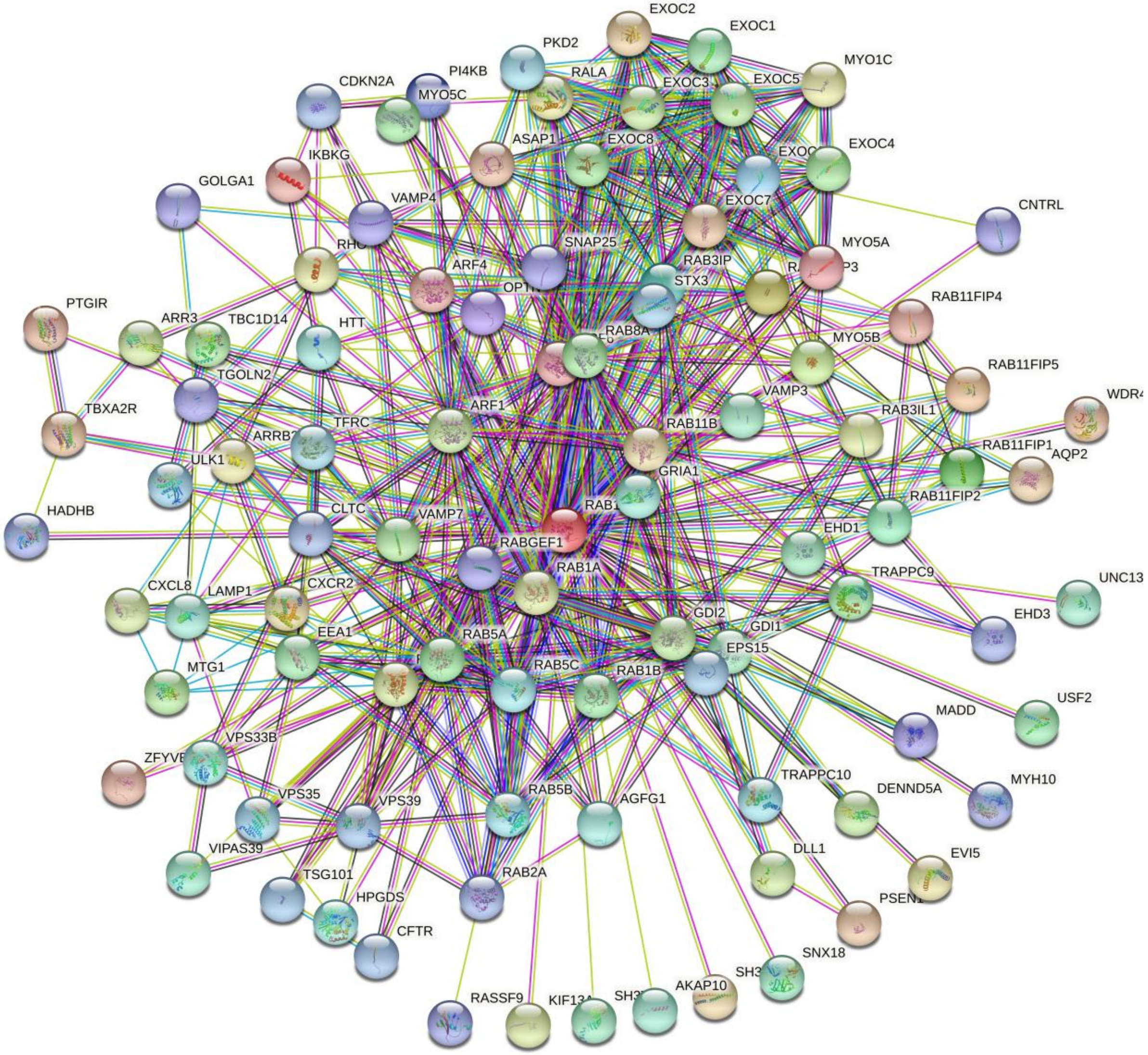

minimum required interaction score: high confidence (0.700)

number of nodes:	97
number of edges:	485
average node degree:	10
avg. local clustering coefficient:	0.699
expected number of edges:	125
PPI enrichment p-value:	< 1.0e-16

G

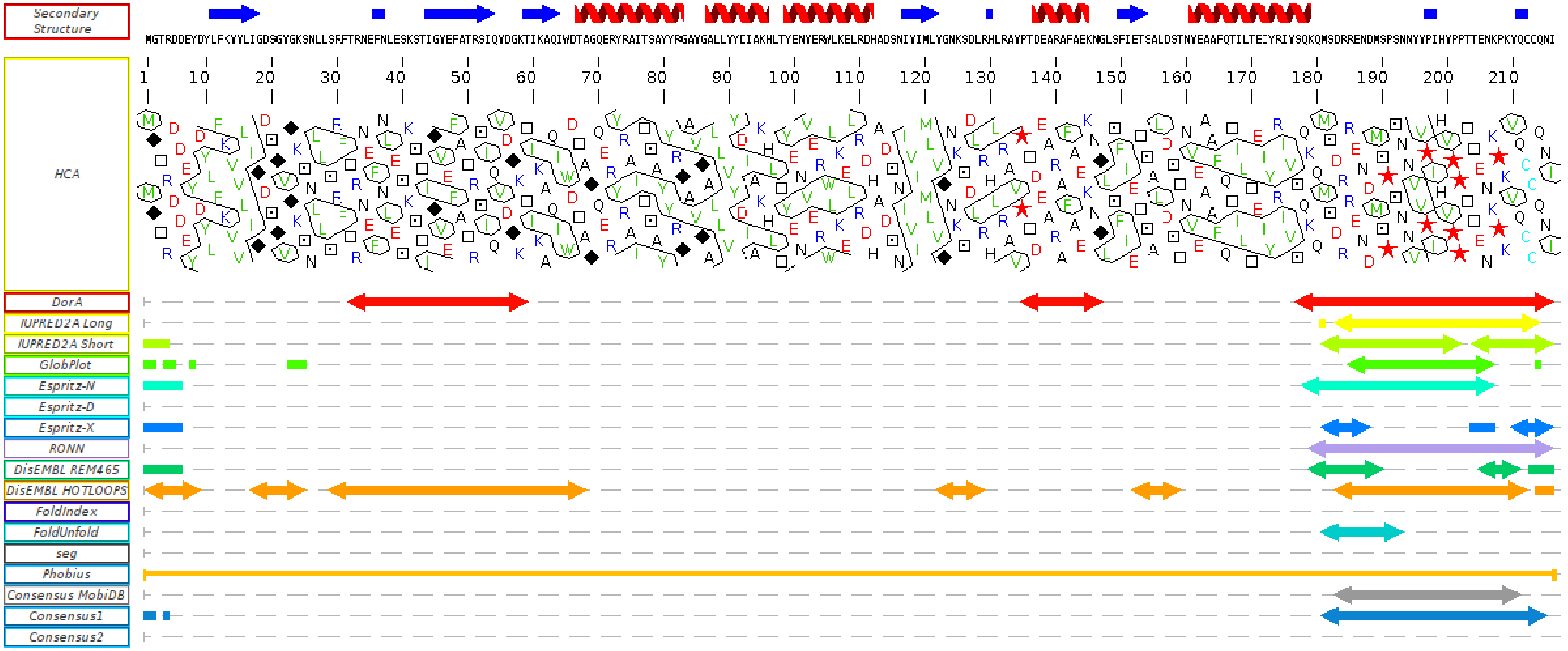

Secondary Structure
HCA
DorA
IUPRED2A Long
IUPRED2A Short
GlobPlot
Espritz-N
Espritz-D
Espritz-X
RONN
DisEMBL REM465
DisEMBL HOTLOOPS
FoldIndex
FoldUnfold
seg
Phobius
Consensus MobiDB
Consensus1
Consensus2

H

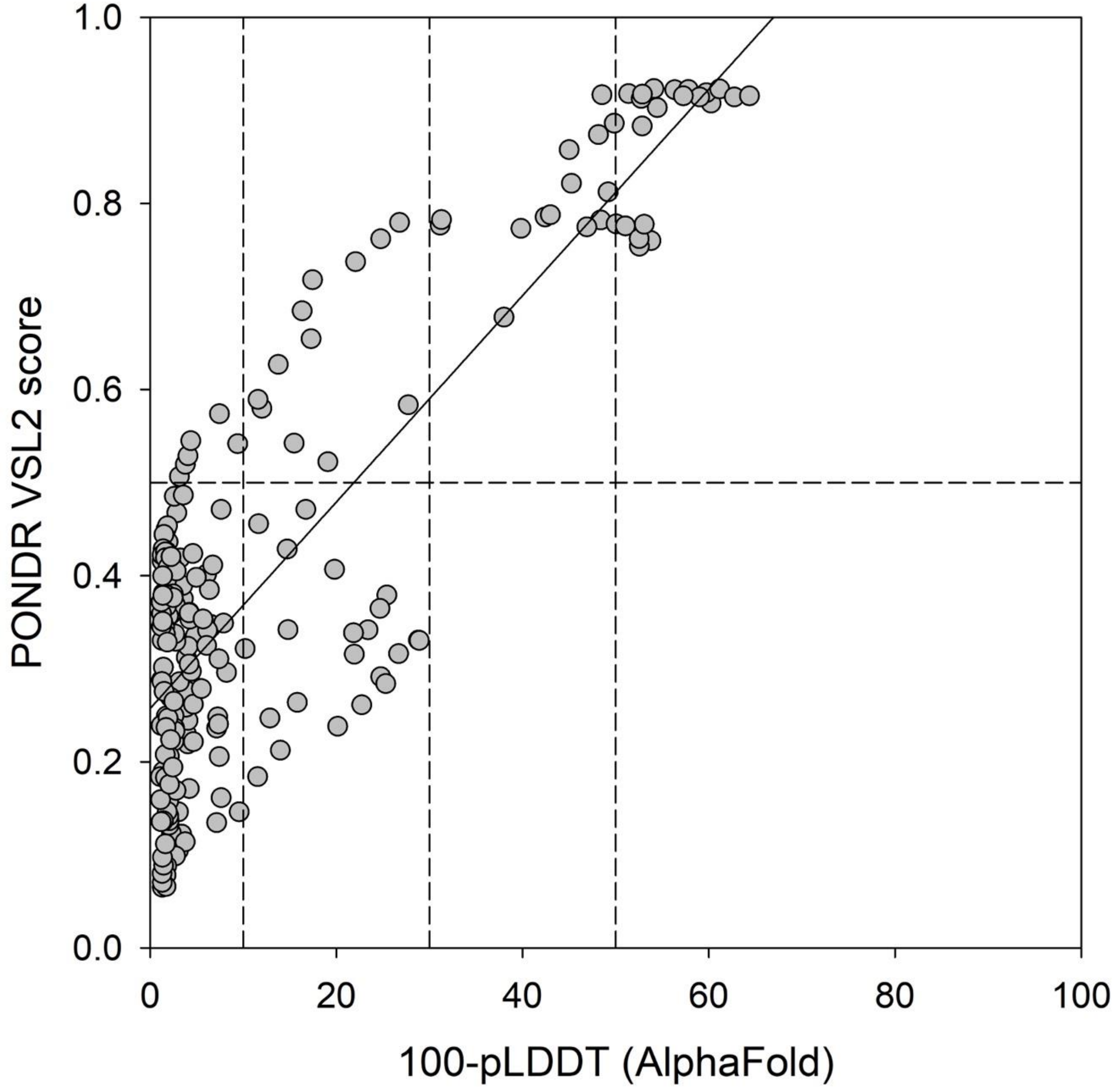
1.0
0.8
0.6
0.4
0.2
0.0
PONDR VSL2 score
0
20
40
60
80
100
100-pLDDT (AlphaFold)

Supplementary Figure S16. Functional disorder in serine/threonine-protein phosphatase PP1-alpha catalytic subunit. **A.** Amino acid sequence. **B.** RIDAO-generated disorder profile. **C.** D2P2-generated functional disorder profile. **D.** FuzDrop output. **E.** AlphaFold2 structure with FuzDrop annotations. **F.** STRING-generated PPI network with corresponding statistics. **G.** MeDor output. **H.** Plot of the *per* residue disorder score (as provided by PONDR® VLS2) versus (100-pLDDT), where pLDDT is the corresponding AlphaFold2 confidence score.

A

>sp|P62136|PP1A_HUMAN Serine/threonine-protein phosphatase PP1-alpha catalytic subunit OS=Homo sapiens OX=9606 GN=PPP1CA PE=1 SV=1
MSDSEKLNLDSIIGRLLEVQGSRPGKNVQLTENEIRGLCLKSREIFLSQPILLELEAPLKICGDIHGQYYDLLRLFEYGGFPPESNYLFLGDYVDRGKQSLETICLLLAYKIKYPENF
FLLRGNHECASINRIYGFYDECKRRYNIKLWKTFTDCFNCLPIAAIVDEKIFCCHGGLSPDLQSMEQIRRIMRPTDVPDQGLLCDLLWSDPDKDVQGWGENDRGVSFTFGAEVVAKFL
HKHDLDLICRAHQVVEDGYEFFAKRQLVTLFSAPNYCGEFDNAGAMMSVDETLMCSFQILKPADKNKGKYGQFSGLNPGGRPITPPRNSAKAKK

B

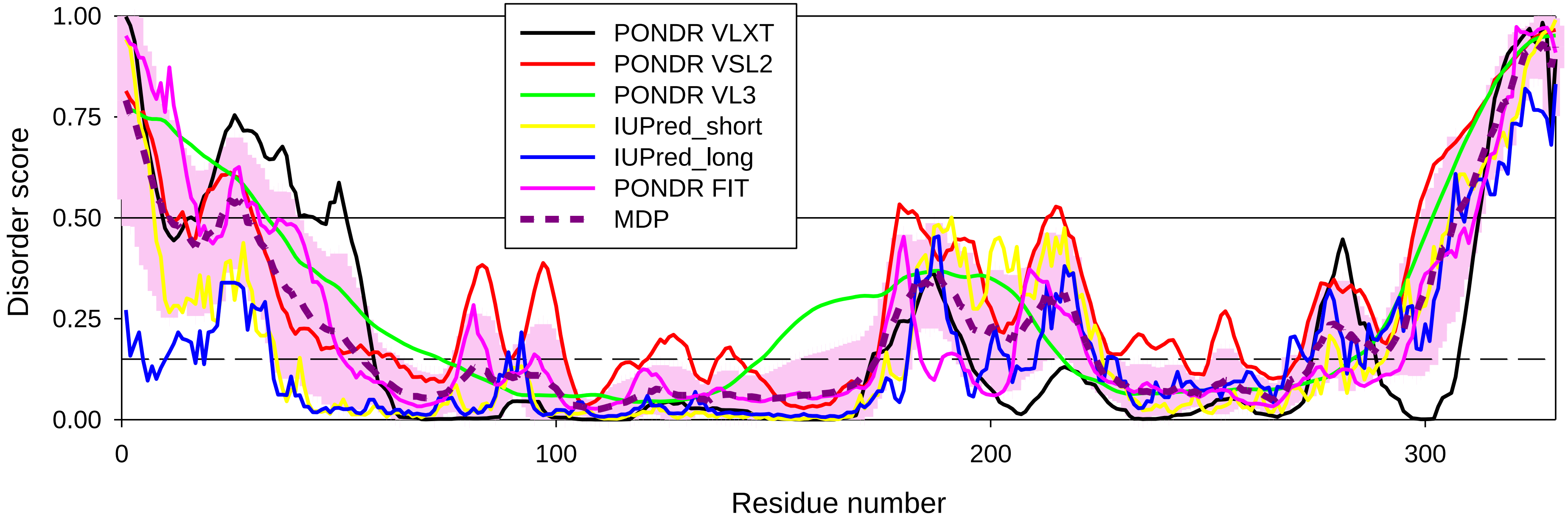

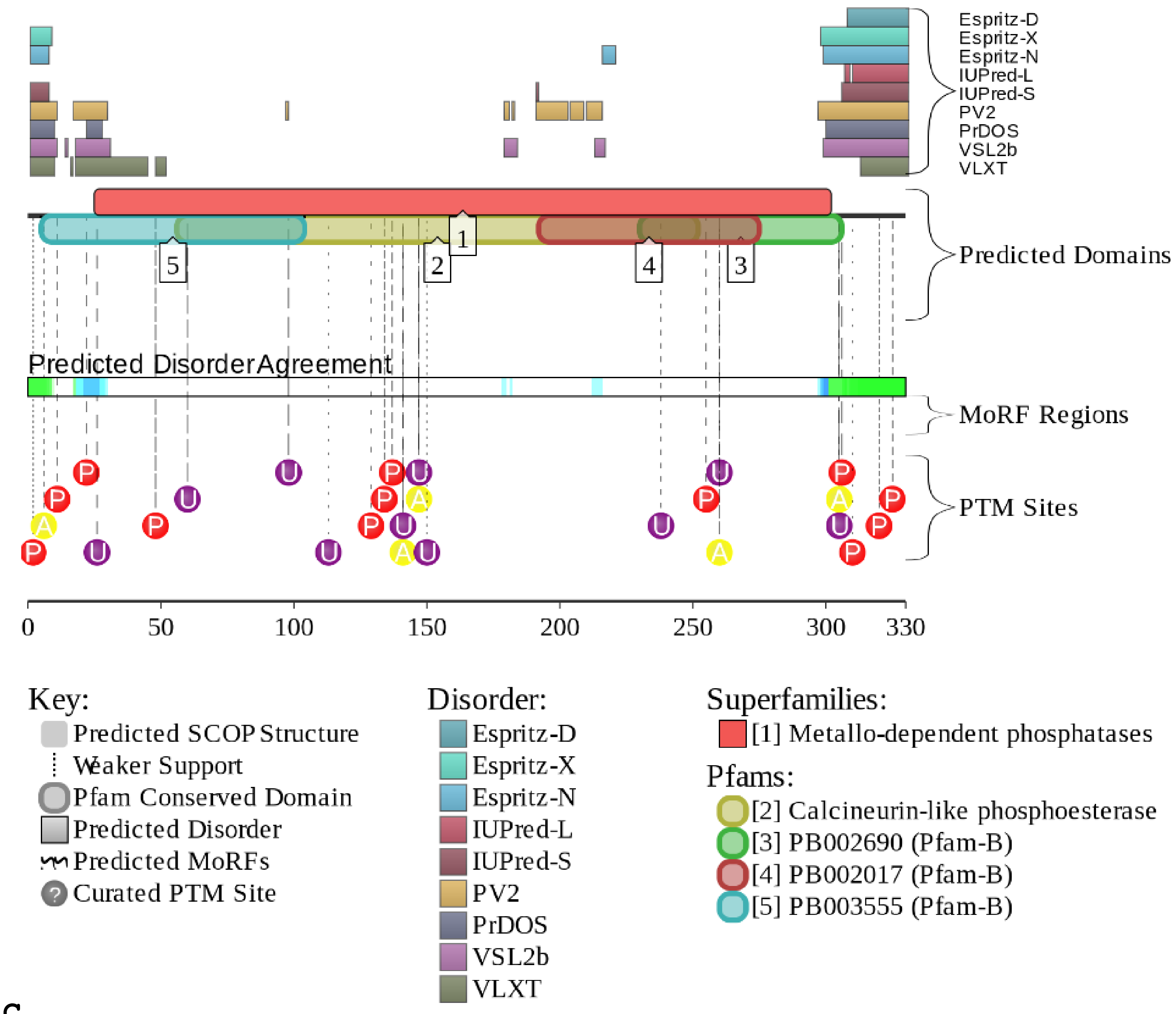

Espritz-D
Espritz-X
Espritz-N
IUPred-L
IUPred-S
PV2
PrDOS
VSL2b
VLXT
Predicted Domains
Predicted Disorder Agreement
MoRF Regions
PTM Sites
0
50
100
150
200
250
300
330
Key:
Predicted SCOP Structure
Weaker Support
Pfam Conserved Domain
Predicted Disorder
Predicted MoRFs
Curated PTM Site
Disorder:
Espritz-D
Espritz-X
Espritz-N
IUPred-L
IUPred-S
PV2
PrDOS
VSL2b
VLXT
Superfamilies:
[1] Metallo-dependent phosphatases
Pfams:
[2] Calcineurin-like phosphoesterase
[3] PB002690 (Pfam-B)
[4] PB002017 (Pfam-B)
[5] PB003555 (Pfam-B)

C

D

Probability of spontaneous liquid-liquid phase separation: $\mathbf{p}_{LLPS} = 0.1692$

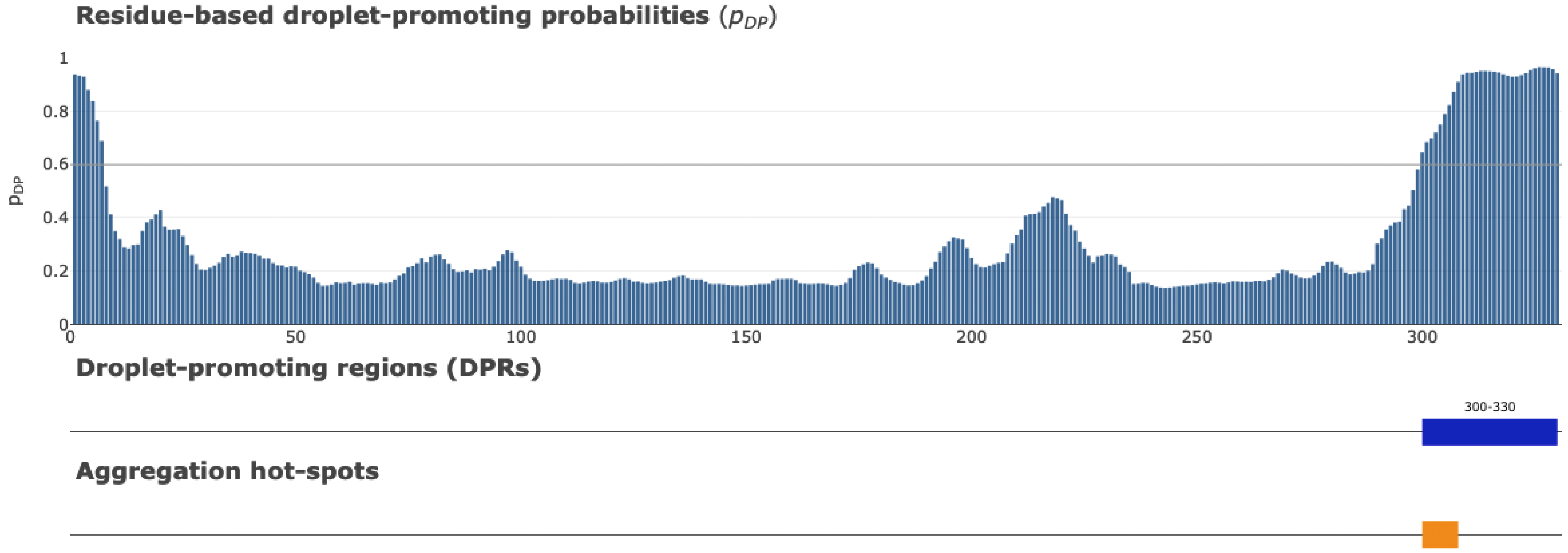

E

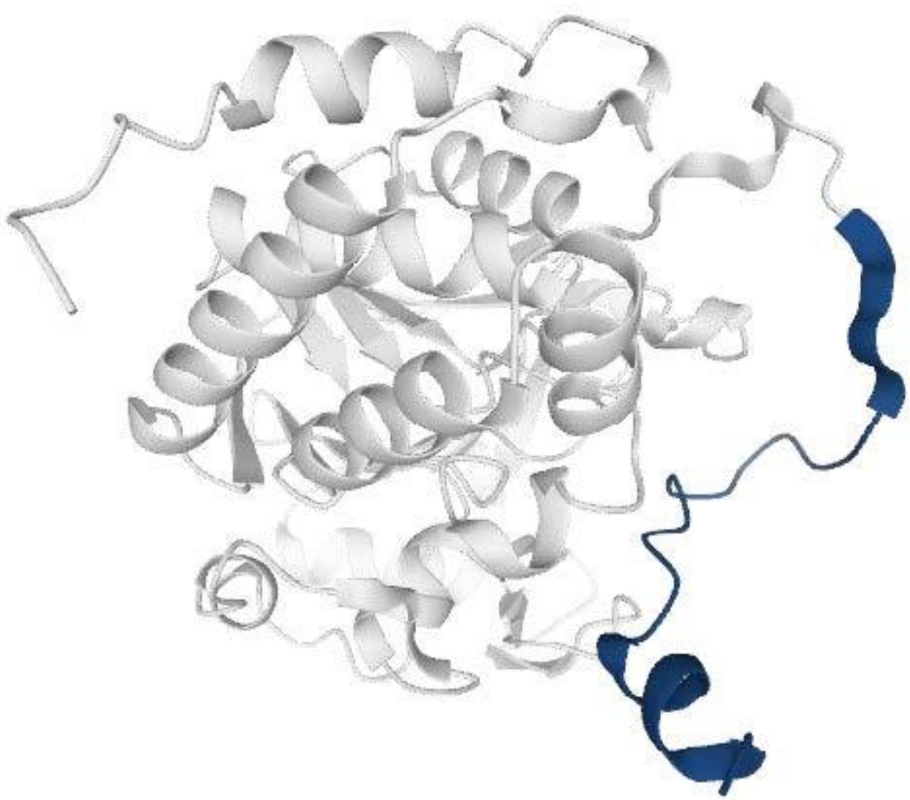

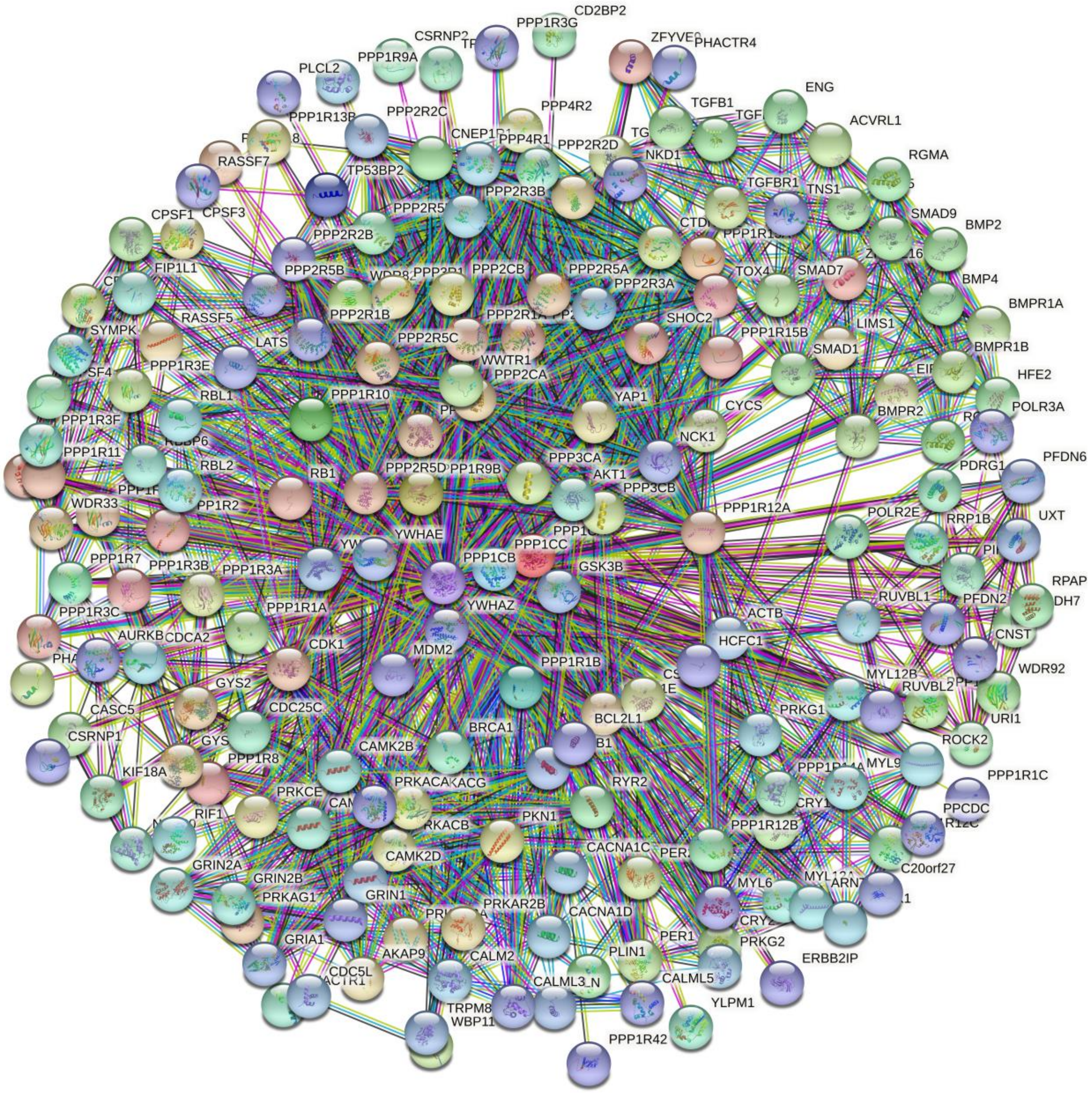
PPP1R3G
CD2BP2
CSRNP2
PPP1R9A
PLCL2
PHACTR4
ENG
TGFB1
ACVRL1
RGMA
PPP4R2
PPP2R2C
PPP1R13B
PPP4R1
PPP2R2D
NKD1
RASSF7
TP53BP2
PPP2R3B
TGFBR1
TNS1
SMAD9
BMP2
CPSF1
CPSF3
PPP2R2B
PPP2R5B
FIP1L1
PPP2CB
PPP2R5A
TOX4
SMAD7
BMP4
PPP2R3A
BMPR1A
RASSF5
PPP2R1B
SHOC2
LIMS1
SYMPK
PPP2R5C
PPP1R15B
SMAD1
BMPR1B
PPP1R3E
WWTR1
PPP2CA
HFE2
RBL1
PPP1R10
YAP1
CYCS
BMPR2
POLR3A
NCK1
PPP1R3F
PPP1R11
RBL2
PPP2R5D
PPP1R9B
PPP3CA
AKT1
PPP3CB
PDRG1
PFDN6
RB1
WDR33
PPP1R12A
POLR2E
RRP1B
UXT
YWHAE
PPP1CC
PPP1R7
PPP1R3B
PPP1R3A
PPP1CB
GSK3B
RPAP
PPP1R3C
RUVBL1
PFDN2
PPP1R1A
YWHAZ
ACTB
CNST
AURKB
CDCA2
CDK1
HCFC1
MDM2
WDR92
PPP1R1B
MYL12B
RUVBL2
URI1
GYS2
CASC5
CDC25C
BCL2L1
PRKG1
CSRNP1
BRCA1
ROCK2
CAMK2B
PPP1R8
KIF18A
RYR2
PPP1R1C
PRKCE
RIF1
PKN1
PPCDC
CAMK2D
CACNA1C
PPP1R12B
C20orf27
GRIN2A
GRIN2B
GRIN1
MYL6
PRKAG1
PRKAR2B
CACNA1D
GRIA1
PER1
PRKG2
AKAP9
CALM2
PLIN1
CDC5L
ERBB2IP
CALML5
TRPM8
YLPM1
WBP11
PPP1R42

F

minimum required interaction score: high confidence (0.700)
number of nodes: 191
number of edges: 1748
average node degree: 18.3
avg. local clustering coefficient: 0.755
expected number of edges: 411
PPI enrichment p-value: < 1.0e-16

G

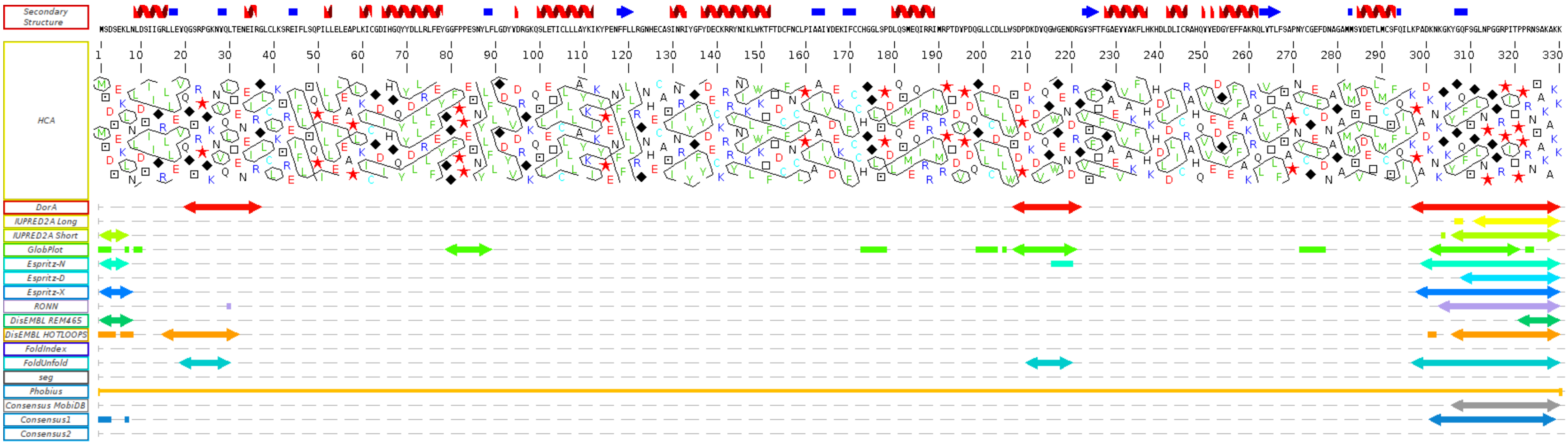
Secondary Structure
HCA
DorA
IUPRED2A Long
IUPRED2A Short
GlobPlot
Espritz-N
Espritz-D
Espritz-X
RONN
DisEMBL REM465
DisEMBL HOTLOOPS
FoldIndex
FoldUnfold
seg
Phobius
Consensus MobiDB
Consensus1
Consensus2
1 10 20 30 40 50 60 70 80 90 100 110 120 130 140 150 160 170 180 190 200 210 220 230 240 250 260 270 280 290 300 310 320 330

H

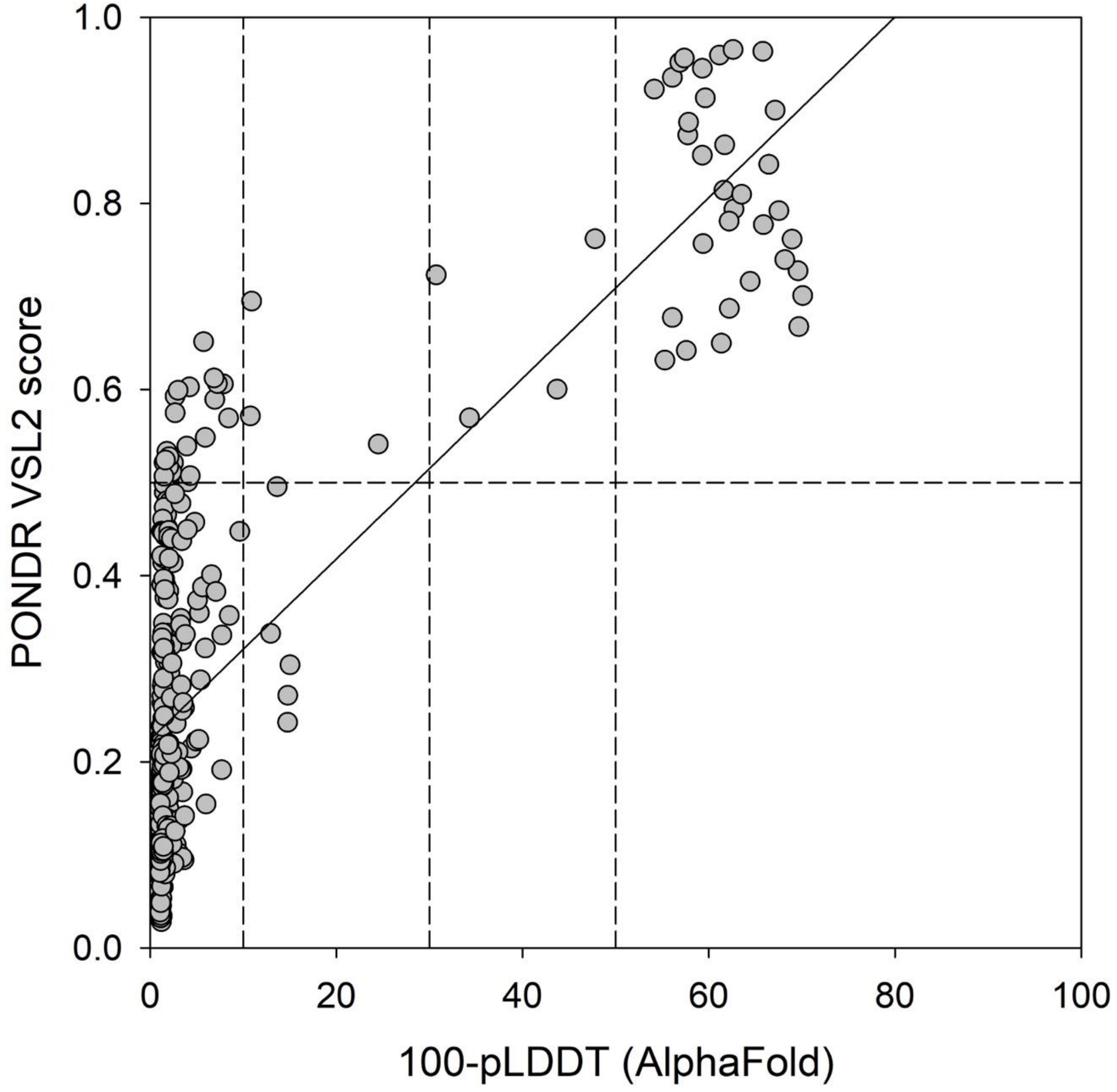
PONDR VSL2 score
1.0
0.8
0.6
0.4
0.2
0.0
0
20
40
60
80
100
100-pLDDT (AlphaFold)

Supplementary Figure S17. Functional disorder in CAD protein. **B.** RIDAO-generated disorder profile. **C.** D2P2-generated functional disorder profile. **D.** FuzDrop output. **E.** AlphaFold2 structure with FuzDrop annotations. **F.** STRING-generated PPI network with corresponding statistics. **G.** MeDor output. **H.** Plot of the *per* residue disorder score (as provided by PONDR® VLS2) versus (100-pLDDT), where pLDDT is the corresponding AlphaFold2 confidence score.

A

>sp|P27708|PYR1_HUMAN CAD protein OS=Homo sapiens OX=9606 GN=CAD PE=1 SV=3
MAALVLEDGSVLRGQPFGAAVSTAGEVVFQTGMVGYPEALTDPSYKAQILVLTYPLIGNYGIPPDEMDEFGLCKWFESSGIHVAALVVGECCPTPSHWSATRTLHEWLQQHGIPGLQG
VDTRELTKKLREQGSLLGKLVQNGTEPSSLPFLDPNARPLVPEVSIKTPRVFNTGGAPRILALDCGLKYNQIRCLCQRGAEVTVVPWDHALDSQEYEGLFLSNGPGDPASYPSVVSTL
SRVLSEPNPRPVFGICLGHQLLALAIGAKTYKMRYGNRGHNQPCLLVGSGRCFLTSQNHGFAVETDSLPADWAPLFTNANDGSNEGIVHNSLPFFSVQFHPEHQAGPSDMELLFDIFL
ETVKEATAGNPGGQTVRERLTERLCPPGIPTPGSGLPPPRKVLILGSGGLSIGQAGEFDYSGSQAIKALKEENIQTLLINPNIATVQTSQGLADKVYFLPITPHYVTQVIRNERPDGV
LLTFGGQTALNCGVELTKAGVLARYGVRVLGTPVETIELTEDRRAFAARMAEIGEHVAPSEAANSLEQAQAAAERLGYPVLVRAAFALGGLGSGFASNREELSALVAPAFAHTSQVLV
DKSLKGWKEIEYEVVRDAYGNCVTVCNMENLDPLGIHTGESIVVAPSQTLNDREYQLLRQTAIKVTQHLGIVGECNVQYALNPESEQYYIIEVNARLSRSSALASKATGYPLAYVAAK
LALGIPLPELRNSVTGGTAAFEPSVDYCVVKIPRWDLSKFLRVSTKIGSCMKSVGEVMGIGRSFEEAFQKALRMVDENCVGFDHTVKPVSDMELETPTDKRIFVVAAALWAGYSVDRL
YELTRIDRWFLHRMKRIIAHAQLLEQHRGQPLPPDLLQQAKCLGFSDKQIALAVLSTELAVRKLRQELGICPAVKQIDTVAAEWPAQTNYLYLTYWGTTHDLTFRTPHVLVLGSGVYR
IGSSVEFDWCAVGCIQQLRKMGYKTIMVNYNPETVSTDYDMCDRLYFDEISFEVVMDIYELENPEGVILSMGGQLPNNMAMALHRQQCRVLGTSPEAIDSAENRFKFSRLLDTIGISQ
PQWRELSDLESARQFCQTVGYPCVVRPSYVLSGAAMNVAYTDGDLERFLSSAAAVSKEHPVVISKFIQEAKEIDVDAVASDGVVAAIAISEHVENAGVHSGDATLVTPPQDITAKTLE
RIKAIVHAVGQELQVTGPFNLQLIAKDDQLKVIECNVRVSRSFPFVSKTLGVDLVALATRVIMGEEVEPVGLMTGSGVVGVKVPQFSFSRLAGADVVLGVEMTSTGEVAGFGESRCEA
YLKAMLSTGFKIPKKNILLTIGSYKNKSELLPTVRLLESLGYSLYASLGTADFYTEHGVKVTAVDWHFEEAVDGECPPQRSILEQLAEKNFELVINLSMRGAGGRRLSSFVTKGYRTR
RLAADFSVPLIIDIKCTKLFVEALGQIGPAPPLKVHVDCMTSQKLVRLPGLIDVHVHLREPGGTHKEDFASGTAAALAGGITMVCAMPNTRPPIIDAPALALAQKLAEAGARCDFALF
LGASSENAGTLGTVAGSAAGLKLYLNETFSELRLDSVVQWMEHFETWPSHLPIVAHAEQQTVAAVLMVAQLTQRSVHICHVARKEEILLIKAAKARGLPVTCEVAPHHLFLSHDDLER
LGPGKGEVRPELGSRQDVEALWENMAVIDCFASDHAPHTLEEKCGSRPPPGFPGLETMLPLLLTAVSEGRLSLDDLLQRLHHNPRRIFHLPPQEDTYVEVDLEHEWTIPSHMPFSKAH
WTPFEGQKVKGTVRRVVLRGEVAYIDGQVLVPPGYGQDVRKWPQGAVPQLPPSAPATSEMTTTPERPRRGIPGLPDGRFHLPPRIHRASDPGLPAEEPKEKSSRKVAEPELMGTPDGT
CYPPPPVPRQASPQNLGTPGLLHPQTSPLLHSLVGQHILSVQQFTKDQMSHLFNVAHTLRMMVQKERSLDILKGKVMASMFYEVSTRTSSSFAAAMARLGGAVLSFSEATSSVQKGES
LADSVQTMSCYADVVVLRHPQPGAVELAAKHCRRPVINAGDGVGEHPTQALLDIFTIREELGTVNGMTITMVGDLKHGRTVHSLACLLTQYRVSLRYVAPPSLRMPPTVRAFVASRGT
KQEEFESIEEALPDTDVLYMTRIQKERFGSTQEYEACFGQFILTPHIMTRAKKKMVVMHPMPRVNEISVEVDSDPRAAYFRQAENGMYIRMALLATVLGRF

B

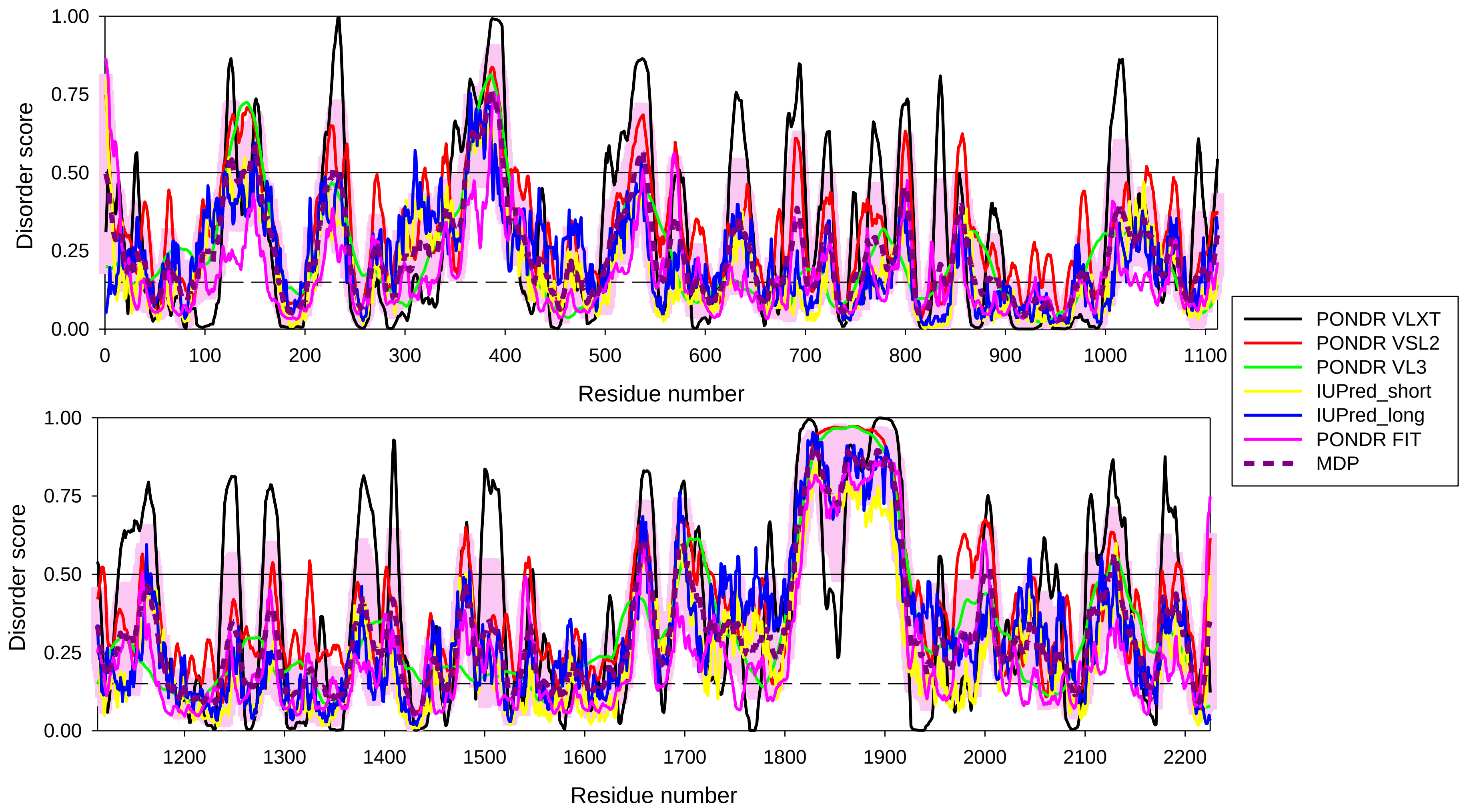

1.00
0.75
0.50
0.25
0.00
Disorder score
0
100
200
300
400
500
600
700
800
900
1000
1100
Residue number
1200
1300
1400
1500
1600
1700
1800
1900
2000
2100
2200
PONDR VLXT
PONDR VSL2
PONDR VL3
IUPred_short
IUPred_long
PONDR FIT
MDP

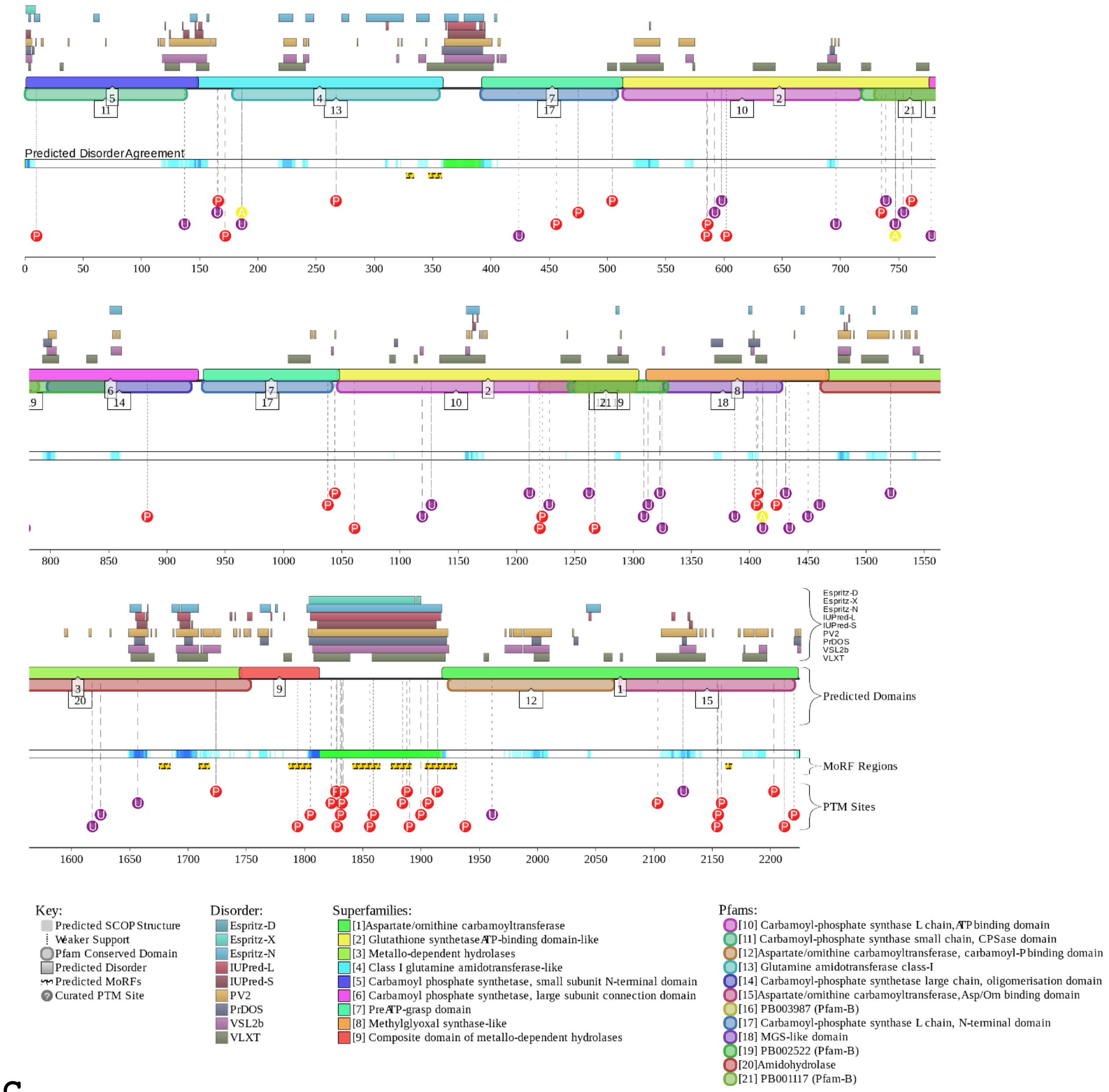
Predicted DisorderAgreement
Esprtiz-D
Esprtiz-X
Esprtiz-N
IUPred-L
IUPred-S
PV2
PrDOS
VSL2b
VLXT
Predicted Domains
MoRF Regions
PTM Sites
Key:
Predicted SCOP Structure
Weaker Support
Pfam Conserved Domain
Predicted Disorder
Predicted MoRFs
Curated PTM Site
Disorder:
Espritz-D
Espritz-X
Espritz-N
IUPred-L
IUPred-S
PV2
PrDOS
VSL2b
VLXT
Superfamilies:
[1]Aspartate/ornithine carbamoyltransferase
[2] Glutathione synthetase ATP-binding domain-like
[3] Metallo-dependent hydrolases
[4] Class I glutamine amidotransferase-like
[5] Carbamoyl phosphate synthetase, small subunit N-terminal domain
[6] Carbamoyl phosphate synthetase, large subunit connection domain
[7] PreATP-grasp domain
[8] Methylglyoxal synthase-like
[9] Composite domain of metallo-dependent hydrolases
Pfams:
[10] Carbamoyl-phosphate synthase L chain, ATP binding domain
[11] Carbamoyl-phosphate synthase small chain, CPSase domain
[12]Aspartate/ornithine carbamoyltransferase, carbamoyl-P binding domain
[13] Glutamine amidotransferase class-I
[14] Carbamoyl-phosphate synthetase large chain, oligomerisation domain
[15]Aspartate/ornithine carbamoyltransferase, Asp/Orn binding domain
[16] PB003987 (Pfam-B)
[17] Carbamoyl-phosphate synthase L chain, N-terminal domain
[18] MGS-like domain
[19] PB002522 (Pfam-B)
[20]Amidohydrolase
[21] PB001117 (Pfam-B)

C

D

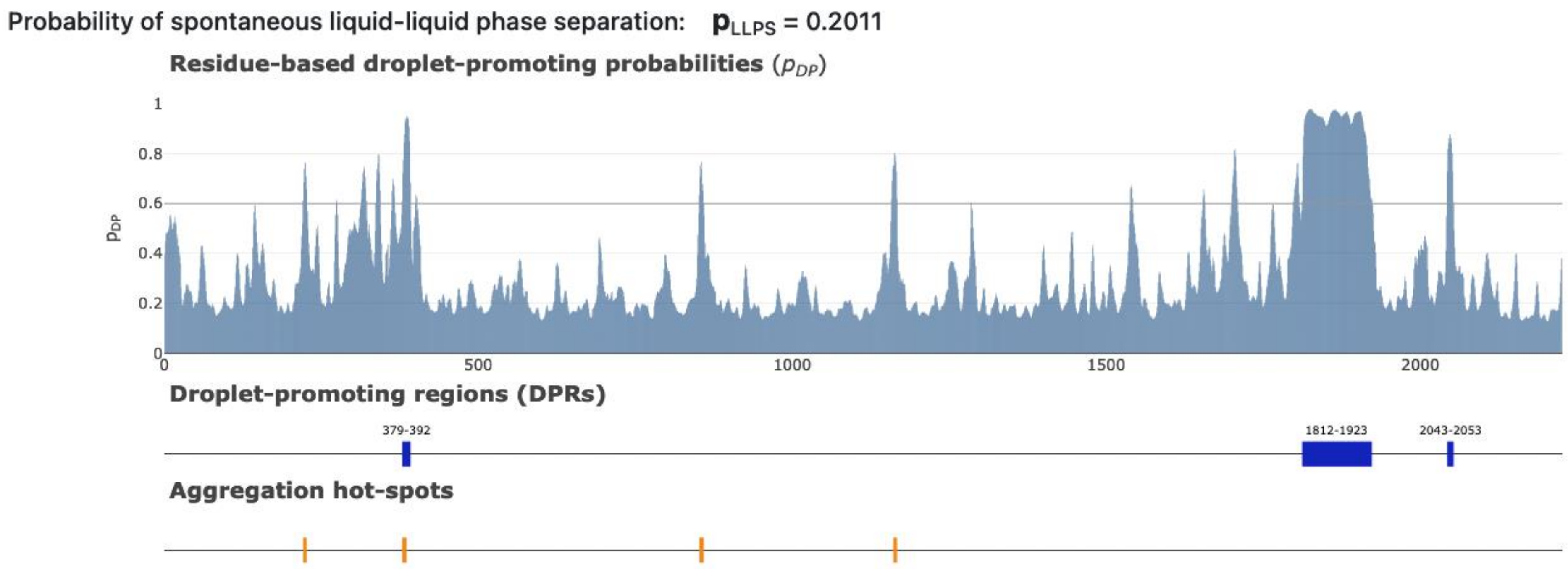
Probability of spontaneous liquid-liquid phase separation: pLLPS = 0.2011
Residue-based droplet-promoting probabilities (pDP)
pDP
1
0.8
0.6
0.4
0.2
0
0
500
1000
1500
2000
Droplet-promoting regions (DPRs)
379-392
1812-1923
2043-2053
Aggregation hot-spots

E

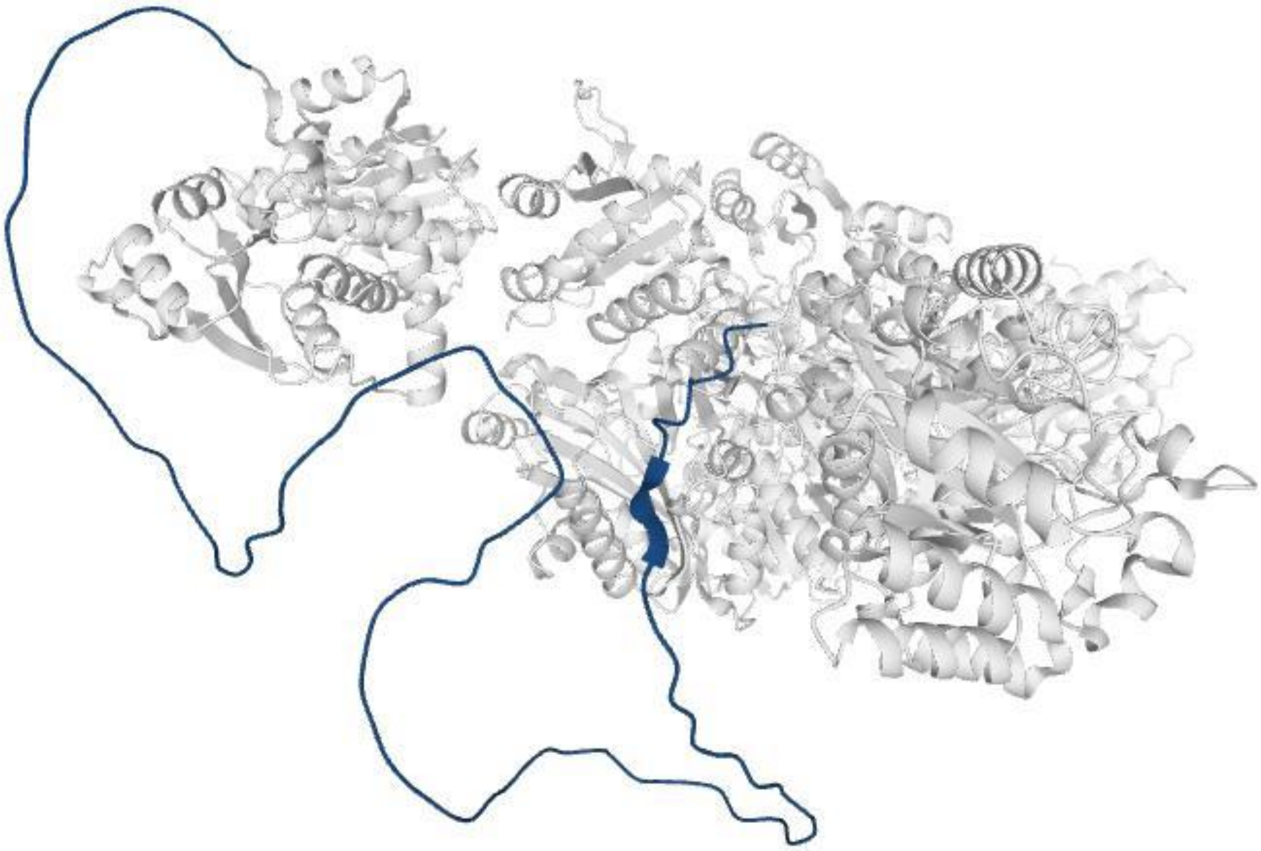

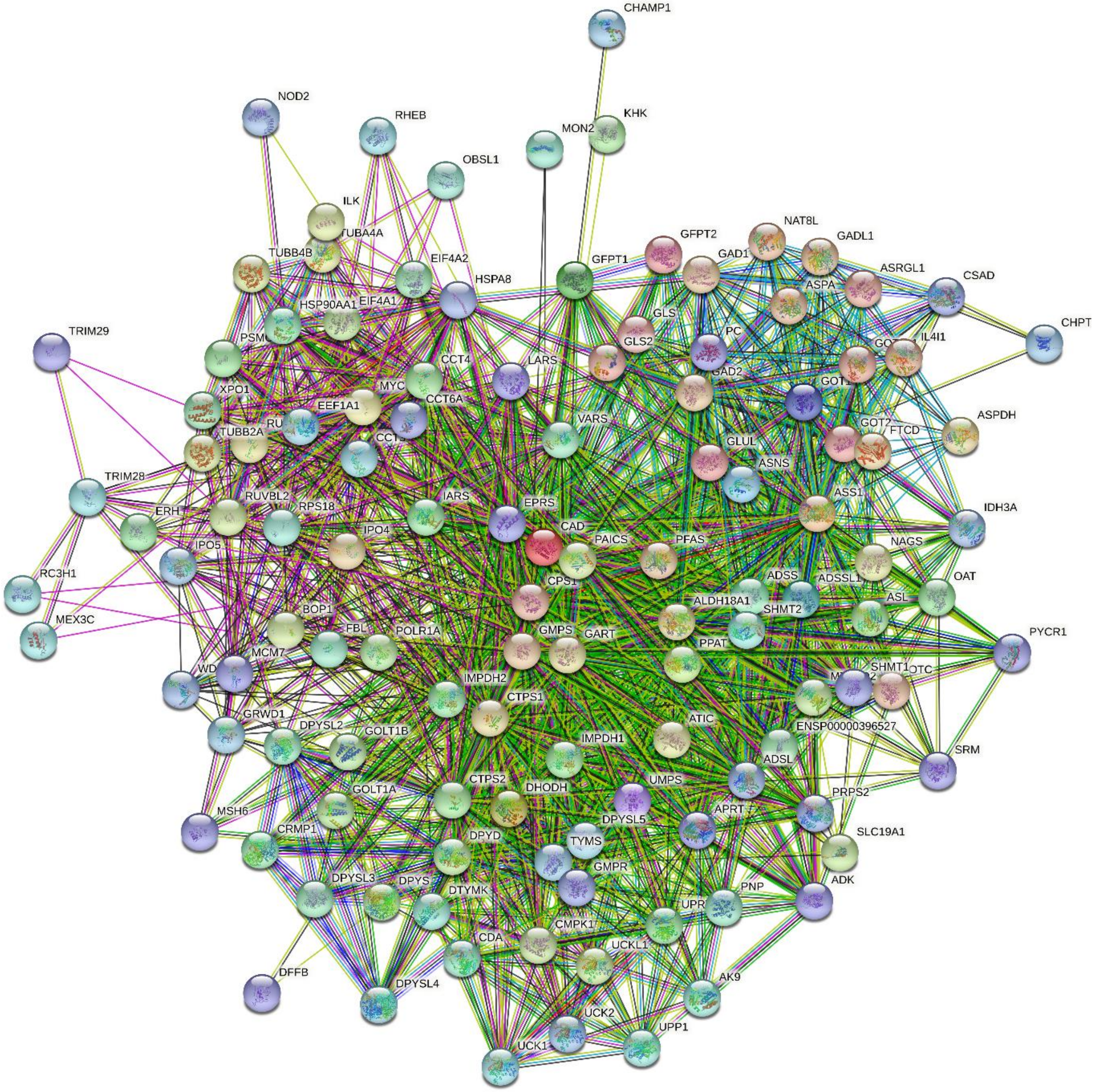
CHAMP1
NOD2
RHEB
KHK
MON2
OBSL1
ILK
TUBA4A
TUBB4B
NAT8L
GFPT2
GADL1
GFPT1
GAD1
ASRGL1
EIF4A2
HSPA8
ASPA
CSAD
HSP90AA1
EIF4A1
CHPT
TRIM29
GLS
GLS2
PC
IL4I1
CCT4
LARS
XPO1
MYC
GAD2
GOT1
CCT6A
EEF1A1
GOT2
FTCD
ASPDH
TUBB2A
VARS
GLUL
ASNS
TRIM28
RUVBL2
IARS
ASS1
ERH
RPS18
EPRS
IDH3A
CAD
IPO4
PAICS
PFAS
NAGS
IPO5
RC3H1
CPS1
ADSS
ADSSL1
OAT
ALDH18A1
ASL
MEX3C
BOP1
SHMT2
FBL
POLR1A
GMPS
GART
PYCR1
PPAT
MCM7
SHMT1
OTC
IMPDH2
CTPS1
GRWD1
ATIC
DPYSL2
ENSP00000396527
GOLT1B
IMPDH1
ADSL
SRM
GOLT1A
CTPS2
DHODH
UMPS
PRPS2
MSH6
APRT
DPYSL5
CRMP1
TYMS
SLC19A1
DPYD
GMPR
DPYSL3
DPYS
DTYMK
ADK
PNP
UPRT
CDA
CMPK1
UCKL1
DFFB
AK9
DPYSL4
UCK2
UPP1
UCK1

F

minimum required interaction score: medium confidence (0.400)

number of nodes:	119
number of edges:	1358
average node degree:	22.8
avg. local clustering coefficient:	0.655
expected number of edges:	325
PPI enrichment p-value:	< 1.0e-16

G

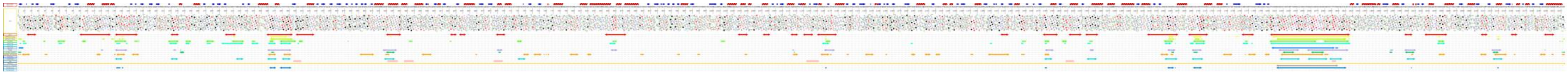

H

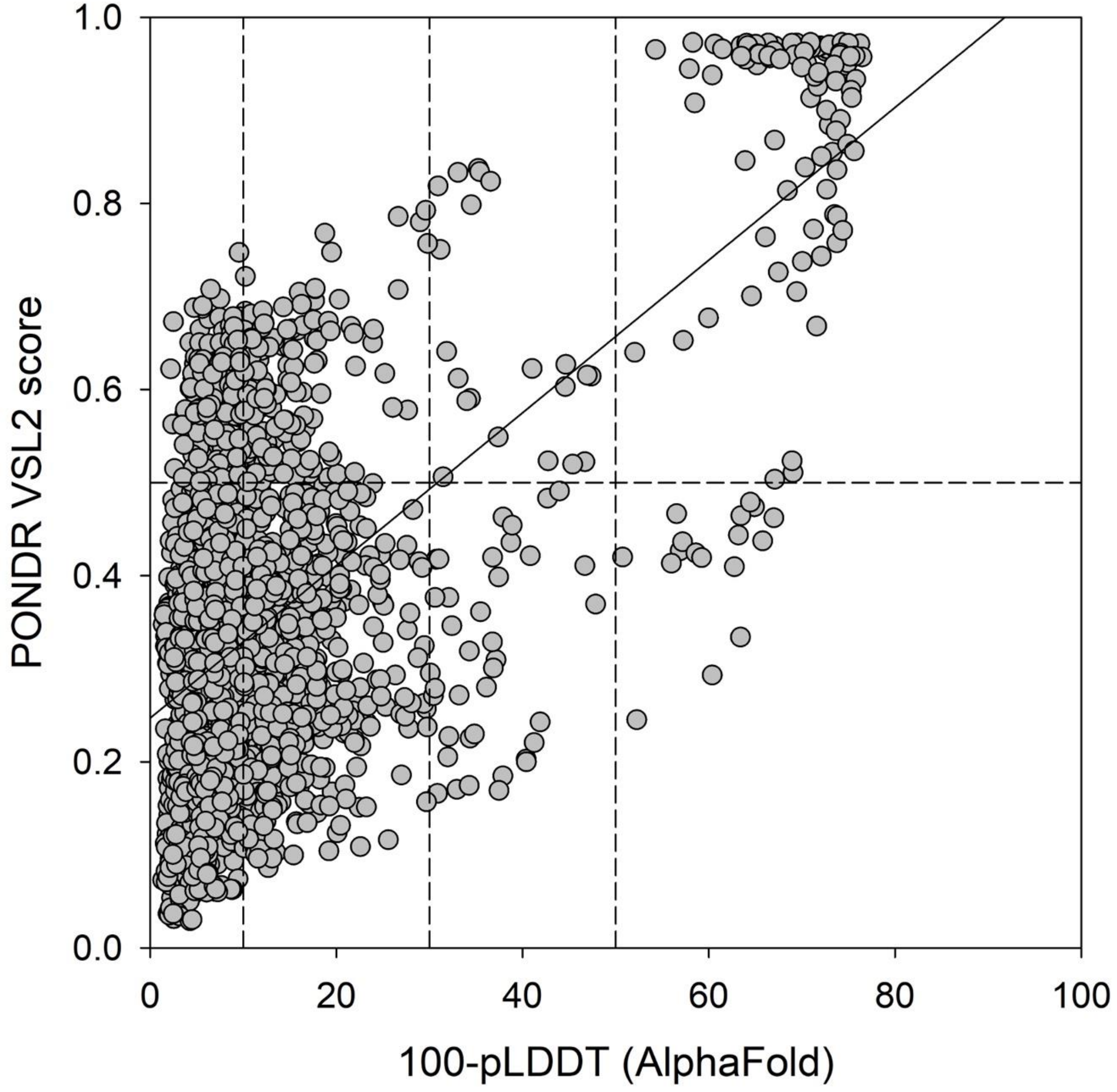

1.0
0.8
0.6
0.4
0.2
0.0
PONDR VSL2 score
0
20
40
60
80
100
100-pLDDT (AlphaFold)

Supplementary Figure S18. Functional disorder in UDP-N-acetylglucosamine--peptide N-acetylglucosaminyltransferase 110 kDa subunit. **B.** RIDAO-generated disorder profile. **C.** D2P2-generated functional disorder profile. **D.** FuzDrop output. **E.** AlphaFold2 structure with FuzDrop annotations. **F.** STRING-generated PPI network with corresponding statistics. **G.** MeDor output. **H.** Plot of the *per* residue disorder score (as provided by PONDR® VLS2) versus (100-pLDDT), where pLDDT is the corresponding AlphaFold2 confidence score.

A

>sp|O15294|OGT1_HUMAN UDP-N-acetylglucosamine--peptide N-acetylglucosaminyltransferase 110 kDa subunit OS=Homo sapiens OX=9606 GN=OGT PE=1 SV=3
MASSVGNVADSTEPTKRMLSFQGLAELAHREYQAGDFEAAERHCMQLWRQEPDNTGVLLLLSSIHFQCRRLDRSAHFSTLAIKQNPLLAEAYSNLGNVYKERGQLQEAIEHYRHALRL
KPDFIDGYINLAAALVAAGDMEGAVQAYVSALQYNPDLYCVRSDLGNLLKALGRLEEAKACYLKAIETQPNFAVAWSNLGCVFNAQGEIWLAIHHFEKAVTLDPNFLDAYINLGNVLK
EARIFDRAVAAYLRALSLSPNHAVVHGNLACVYYEQGLIDLAIDTYRRAIELQPHFPDAYCNLANALKEKGSVAEAEDCYNTALRLCPTHADSLNNLANIKREQGNIEEAVRLYRKAL
EVFPEFAAAHSNLASVLQQQGKLQEALMHYKEAIRISPTFADAYSNMGNTLKEMQDVQGALQCYTRAIQINPAFADAHSNLASIHKDSGNIPEAIASYRTALKLKPDFPDAYCNLAHC
LQIVCDWTDYDERMKKLVSIVADQLEKNRLPSVHPHHSMLYPLSHGFRKAIAERHGNLCLDKINVLHKPPYEHPKDLKLSDGRLRVGYVSSDFGNHPTSHLMQSIPGMHNPDKFEVFC
YALSPDDGTNFRVKVMAEANHFIDLSQIPCNGKAADRIHQDGIHILVNMNGYTKGARNELFALRPAPIQAMWLGYPGTSGALFMDYIITDQETSPAEVAEQYSEKLAYMPHTFFIGDH
ANMFPHLKKKAVIDFKSNGHIYDNRIVLNGIDLKAFLDSLPDVKIVKMKCPDGGDNADSSNTALNMPVIPMNTIAEAVIEMINRGQIQITINGFSISNGLATTQINNKAATGEEVPRT
IIVTTRSQYGLPEDAIVYCNFNQLYKIDPSTLQMWANILKRVPNSVLWLLRFPAVGEPNIQQYAQNMGLPQNRIIFSPVAPKEEHVRRGQLADVCLDTPLCNGHTTGMDVLWAGTPMV
TMPGETLASRVAASQLTCLGCLELIAKNRQEYEDIAVKLGTDLEYLKKVRGKVWKQRISSPLFNTKQYTMELERLYLQMWEHYAAGNKPDHMIKPVEVTESA

B

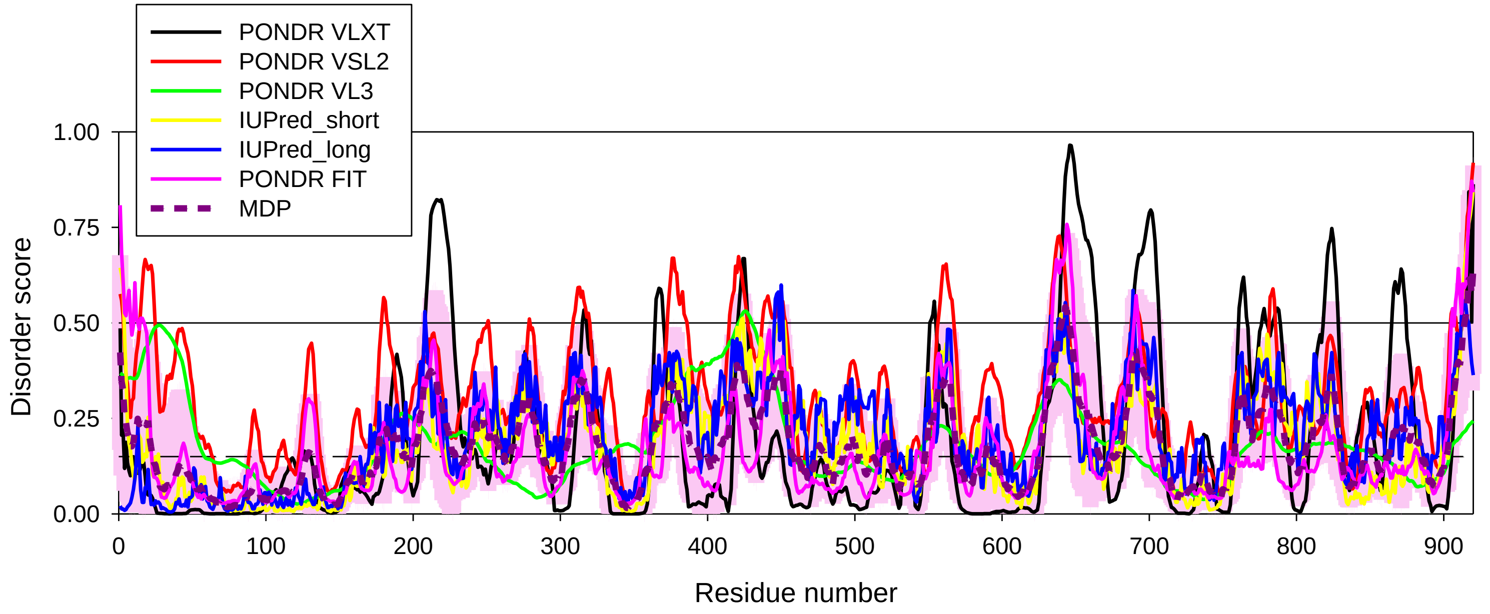

C

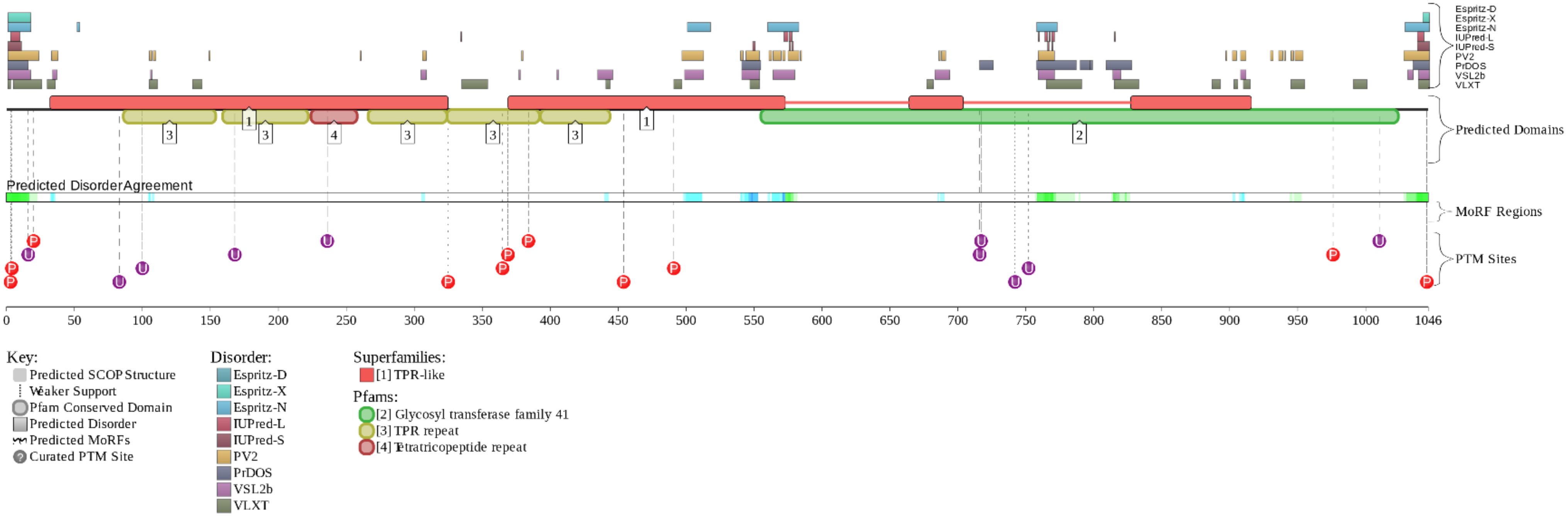

Espritz-D
Espritz-X
Espritz-N
IUPred-L
IUPred-S
PV2
PrDOS
VSL2b
VLXT
Predicted Domains
Predicted Disorder Agreement
MoRF Regions
PTM Sites
0 50 100 150 200 250 300 350 400 450 500 550 600 650 700 750 800 850 900 950 1000 1046
Key:
Predicted SCOP Structure
Weaker Support
Pfam Conserved Domain
Predicted Disorder
Predicted MoRFs
Curated PTM Site
Disorder:
Espritz-D
Espritz-X
Espritz-N
IUPred-L
IUPred-S
PV2
PrDOS
VSL2b
VLXT
Superfamilies:
[1] TPR-like
Pfams:
[2] Glycosyl transferase family 41
[3] TPR repeat
[4] Tetratricopeptide repeat

D

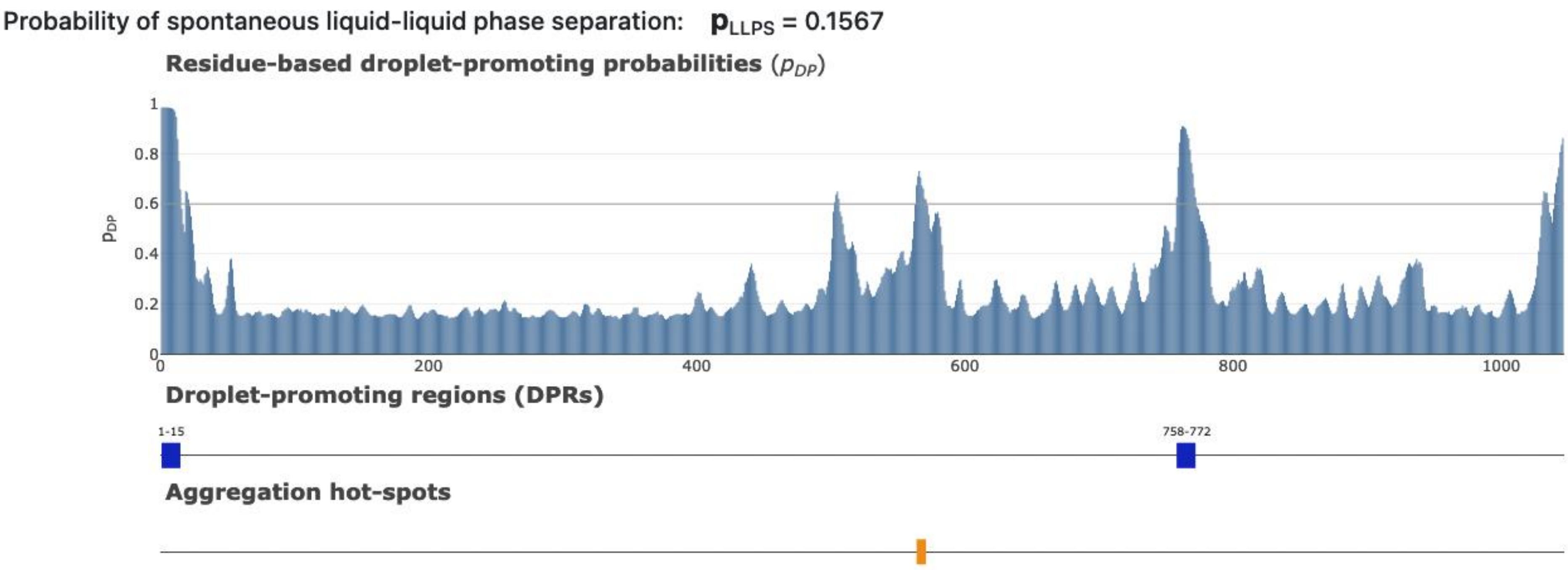

E

There is no Alpha-Fold-generated 3D structure of this protein in FuzDrop.

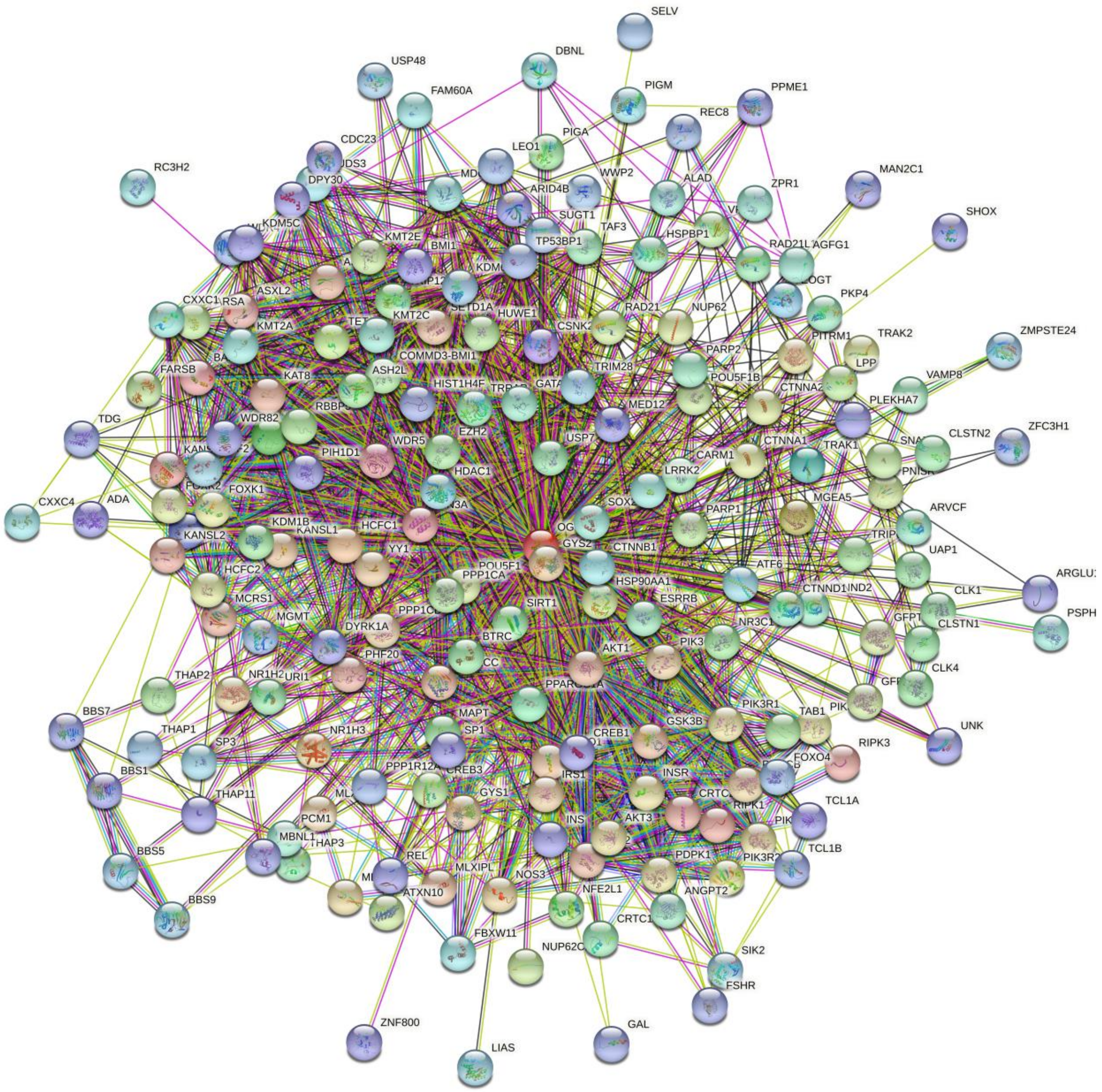
SELV
DBNL
USP48
PIGM
PPME1
FAM60A
REC8
PIGA
CDC23
LEO1
RC3H2
DPY30
WWP2
ALAD
ZPR1
MAN2C1
ARID4B
SHOX
SUGT1
TAF3
KDM5C
HSPBP1
KMT2E
BMI1
TP53BP1
AGFG1
PKP4
CXXC1
ASXL2
SETD1A
HUWE1
KMT2A
KMT2C
RAD21
NUP62
TRAK2
ZMPSTE24
PITRM1
COMMD3-BMI1
PARP2
LPP
FARSB
KAT8
ASH2L
TRIM28
POU5F1B
VAMP8
HIST1H4F
CTNNA2
PLEKHA7
TDG
WDR82
MED12
ZFC3H1
CLSTN2
EZH2
PIH1D1
WDR5
USP7
CARM1
CTNNA1
TRAK1
HDAC1
LRRK2
CXXC4
ADA
FOXK1
MGEA5
ARVCF
PARP1
KDM1B
KANSL1
HCFC1
KANSL2
YY1
CTNNB1
UAP1
HCFC2
POU5F1
PPP1CA
HSP90AA1
ATF6
CTNND1
CLK1
MCRS1
SIRT1
ESRRB
PSPH
MGMT
DYRK1A
CLSTN1
BTRC
NR3C1
PHF20
AKT1
THAP2
NR1H2
URI1
CLK4
PIK3R1
BBS7
MAPT
TAB1
UNK
THAP1
NR1H3
SP1
GSK3B
CREB1
RIPK3
SP3
FOXO4
CREB3
IRS1
INSR
BBS1
THAP11
GYS1
TCL1A
PCM1
INS
AKT3
RIPK1
MBNL1
THAP3
TCL1B
BBS5
PDPK1
REL
MLXIPL
NOS3
BBS9
ATXN10
ANGPT2
NFE2L1
CRTC1
FBXW11
SIK2
FSHR
ZNF800
GAL
LIAS

F

minimum required interaction score: medium confidence (0.400)
number of nodes: 189
number of edges: 1609
average node degree: 17
avg. local clustering coefficient: 0.617
expected number of edges: 716
PPI enrichment p-value: < 1.0e-16

G

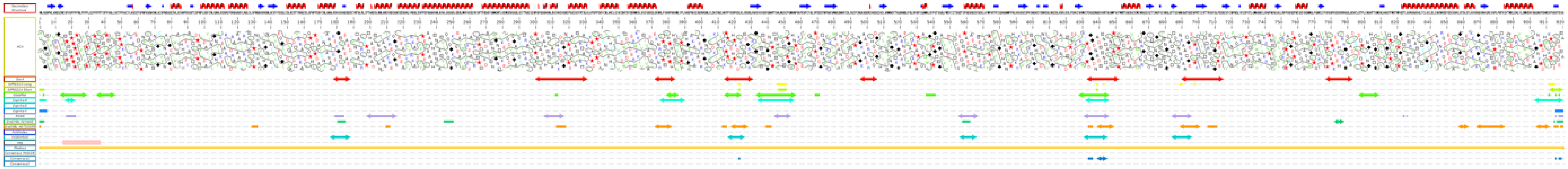

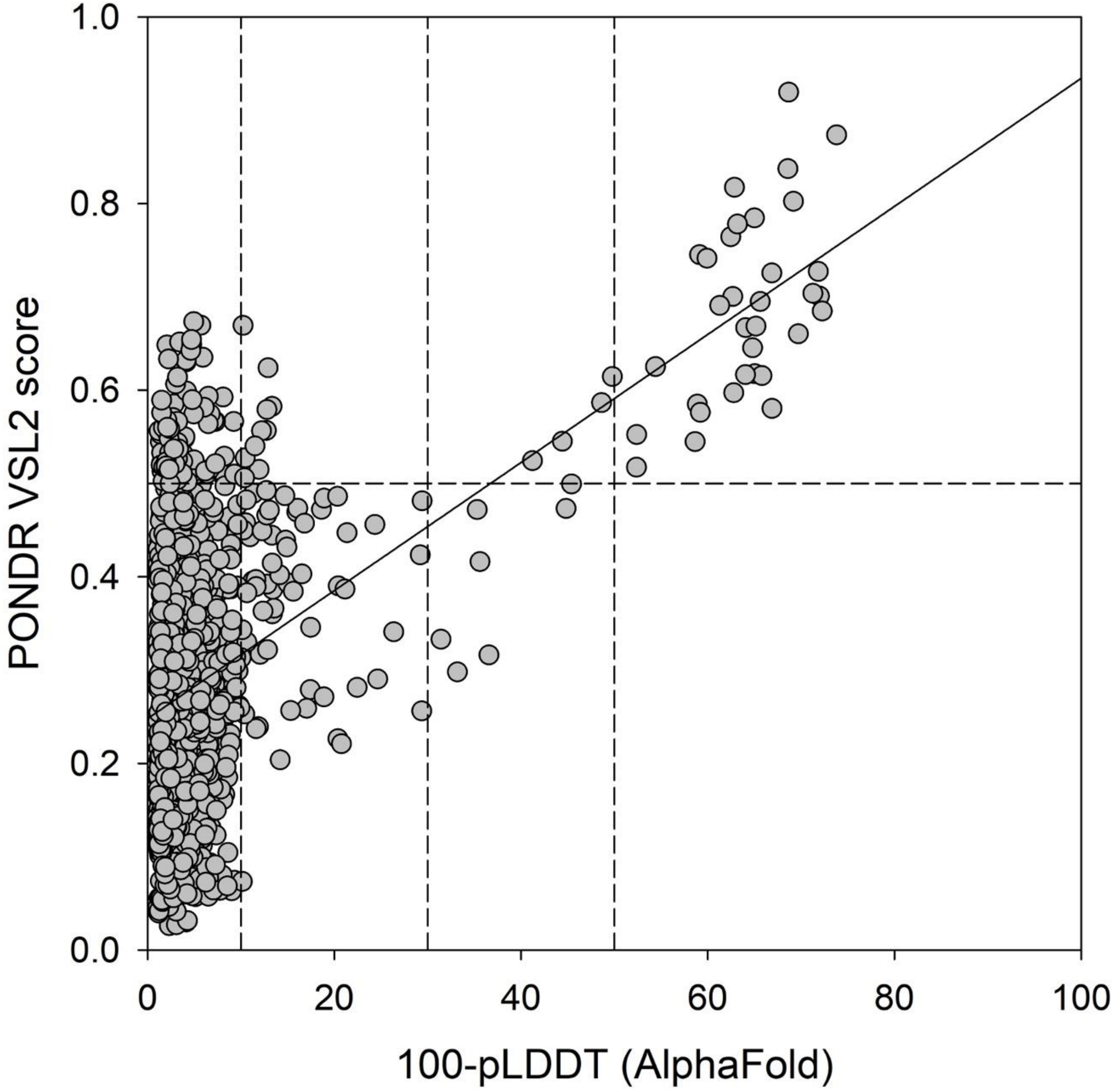

PONDR VSL2 score
100-pLDDT (AlphaFold)
1.0
0.8
0.6
0.4
0.2
0.0
0
20
40
60
80
100

H
